# Expanding the scope of native chemical ligation – templated small molecule drug synthesis *via* benzanilide formation†

**DOI:** 10.1039/d1sc00513h

**Published:** 2021-09-14

**Authors:** Richard Houska, Marvin Björn Stutz, Oliver Seitz

**Affiliations:** Department of Chemistry, Humboldt-Universität zu Berlin Brook-Taylor-Strasse 2 12489 Berlin Germany oliver.seitz@chemie.hu-berlin.de

## Abstract

We describe a reaction system that enables the synthesis of Bcr–Abl tyrosine kinase inhibitors (TKI) *via* benzanilide formation in water. The reaction is based on native chemical ligation (NCL). In contrast to previous applications, we used the NCL chemistry to establish aromatic rather than aliphatic amide bonds in coupling reactions between benzoyl and *o*-mercaptoaniline fragments. The method was applied for the synthesis of thiolated ponatinib and GZD824 derivatives. Acid treatment provided benzothiazole structures, which opens opportunities for diversification. Thiolation affected the affinity for Abl1 kinase only moderately. Of note, a ponatinib-derived benzothiazole also showed nanomolar affinity. NCL-enabled benzanilide formation may prove useful for fragment-based drug discovery. To show that benzanilide synthesis can be put under the control of a template, we connected the benzoyl and *o*-mercaptoaniline fragments to DNA and peptide nucleic acid (PNA) oligomers. Complementary RNA templates enabled adjacent binding of reactive conjugates triggering a rapid benzoyl transfer from a thioester-linked DNA conjugate to an *o*-mercaptoaniline-DNA or -PNA conjugate. We evaluated the influence of linker length and unpaired spacer nucleotides within the RNA template on the product yield. The data suggest that nucleic acid-templated benzanilide formation could find application in the establishment of DNA-encoded combinatorial libraries (DEL).

## Abstract 2

The templated native chemical ligation between benzoyl thioesters and *o*-mercaptoaniline fragments proceeds in water and provides benzanilides that have nanomolar affinity for Abl1 kinase.

## Graphical abstract

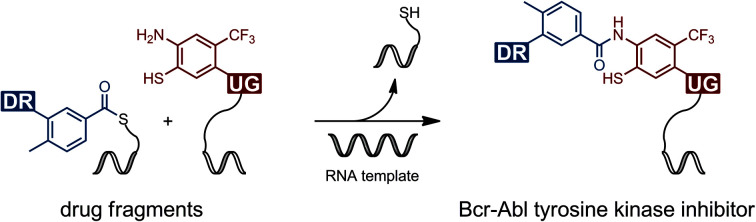

## Introduction

**Fig. 1:**
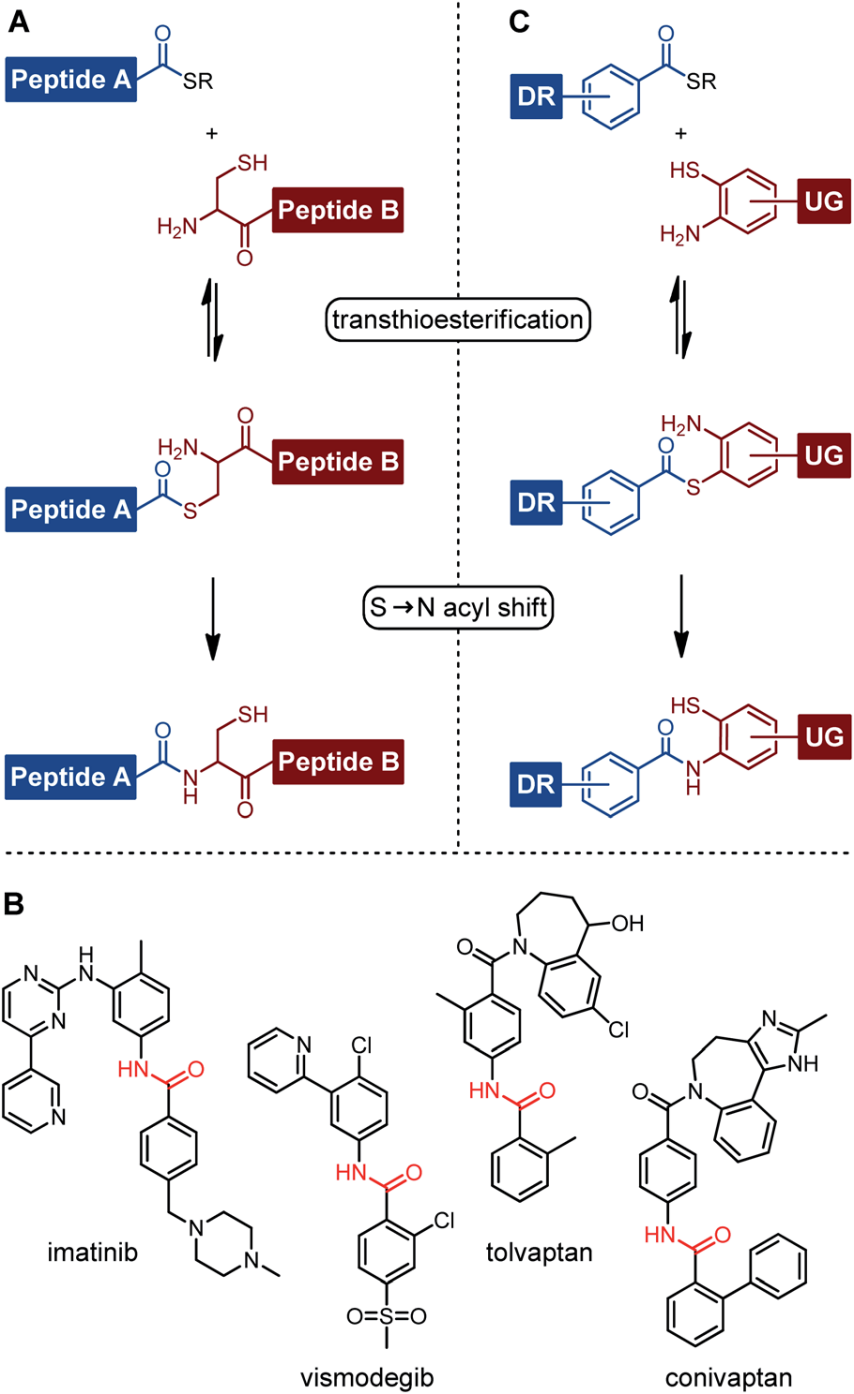
(A) Mechanism of native chemical ligation (NCL). (B) Small molecule drugs containing benzanilide units. (C) NCL-based small molecule drug synthesis *via* benzanilide formation.

The amide bond is a key structural feature of peptides and proteins, as well as natural compounds and drugs. Amongst the many methods available for amide bond formation, native chemical ligation (NCL) is unique.^1–3^ This reaction proceeds in aqueous solution and tolerates most unprotected functional groups. A two-step mechanism provides for extreme chemoselectivity. In the original form of NCL, a peptide thioester first reacts with the thiol side chain of cysteine yielding a thioester intermediate, which joins two peptide units (Fig. 1A). A subsequent S → N acyl shift then proceeds through a kinetically favored five-membered ring and establishes the amide bond. The NCL has emerged as the key enabling reaction for the total chemical synthesis of proteins, peptides, or peptidomimetic compounds.^4,5^ Cysteine has been replaced by various other 1,2-aminothiol structures to allow the formation of aliphatic amide bonds in segment coupling reactions.^3,6–11^ To our surprise, the NCL approach has not been applied in the synthesis of small molecules comprising aromatic amide bonds. However, a variety of small molecule drugs contain amides in which aromatic structures are directly linked to the amide bond. For example, imatinib, vismodegib, tolvaptan, and conivaptan include benzanilide units (Fig. 1B). We assumed that such molecules should be accessible by NCL reactions (Fig. 1C). The ability to establish benzanilide bonds under mild conditions and in aqueous solution could be an asset to fragment-based drug discovery approaches involving reactions templated by protein or nucleic acid targets. For example, DNA-templated synthesis (DTS) of small molecules is an effective method to create DNA-encoded combinatorial libraries (DEL), which allow the simultaneous screening of millions to trillions of different chemical compounds in a single experiment, leading to a substantially decreased time and cost burden compared to traditional high-throughput screenings.^12–17^ In exploratory studies, DTS has been performed within living cells or cell lysates.^18–23^ In one application scenario, reactions triggered by cell endogenous RNA lead to activation of fluorescence from non-fluorescent precursors.^24–28^ Such reactions can provide information about the presence and localization of intracellular RNA targets. It has been proposed that disease-specific mRNA could act as a template for the release or formation of drug-like molecules inside an affected cell.^21,22,29–40^

**Fig. 2:**
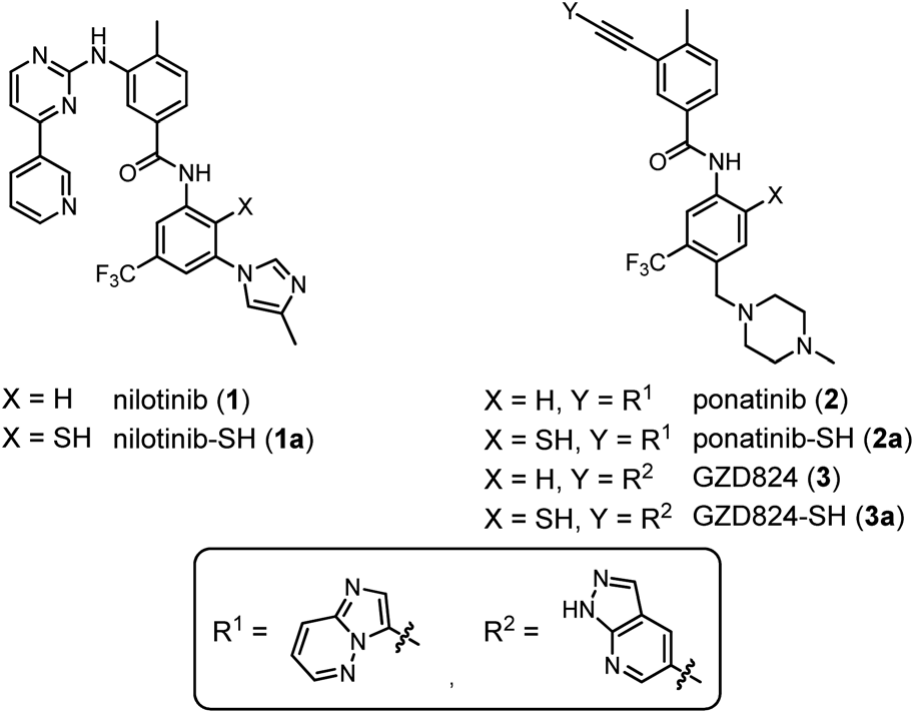
Chemical structures of nilotinib (**1**), ponatinib (**2**), and GZD824 (**3**), as well as their thiolated derivatives **1a–3a**.

In this study, we explored whether NCL chemistry can provide access to small molecule benzanilide structures found in tyrosine kinase inhibitors (TKI). Our specific interest pertained to the small molecule inhibitors of the Bcr–Abl kinase such as nilotinib (**1**) and ponatinib (**2**) (Fig. 2), which are used in the clinic for treatment of chronic myeloid leukemia (CML).^41^ We show that the formed benzanilide and unexpectedly obtained benzothiazole structures both bind the Abl1 tyrosine kinase with nanomolar activity.

Based on our long-standing interest in nucleic acid-instructed processes, the focus of the study was set to the RNA-templated synthesis of a small molecule Bcr–Abl TKI. Ideally, templated reactions for applications in the field of DEL or pro-drug approaches should provide high product yields in presence of template and within a reasonable time frame, while product formation in absence of template should remain low. To unravel the criteria for a highly efficient formation of amide bonds between a benzoate and a weakly nucleophilic aniline, we compared reactive conjugates based on DNA and peptide nucleic acid (PNA), varied the length and flexibility of linkers connecting the benzanilide fragment with the nucleic acid part, and assessed the influence of template architecture.

## Results and discussion

Nilotinib (**1**),^42^ ponatinib (**2**),^43^ and GZD824 (**3**)^44^ inhibit kinases such as Bcr–Abl with IC_50_ values in the low nanomolar range (Fig. 2). Nilotinib (**1**) and ponatinib (**2**) are approved for the therapy of CML and Bcr–Abl-positive acute lymphoblastic leukemia (ALL).^45^ GZD824 (**3**) entered phase II clinical studies.^46^

We envisioned that the benzanilide core of the three TKIs could be accessed by NCL-type chemistry, with potential applications in fragment-based and DNA-templated drug discovery. To enable the envisaged NCL-type reaction, a thiol group needs to be introduced at one of the *ortho*-positions to the anilide nitrogen. We focused on derivatives **1a–3a**, in which the thiol group is introduced in the position *para* to the trifluoromethyl residue (Fig. 2). According to crystal structure analyses, these thiolation sites are not involved in tight contacts with the kinase domain.^42–44^

**Scheme 1:**
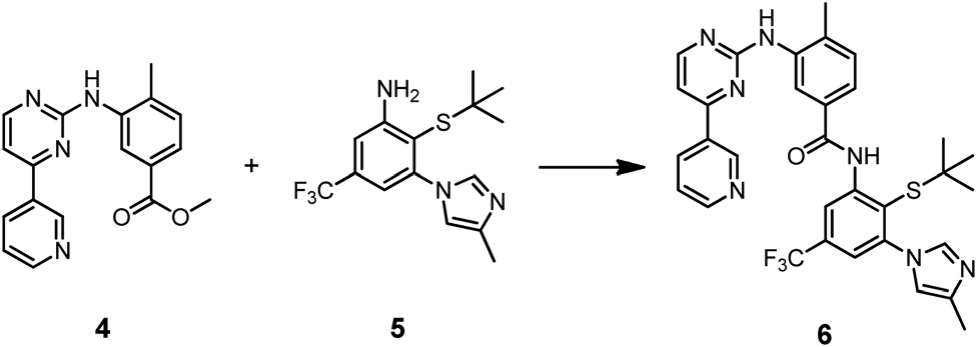
Formation of the core benzanilide structure of nilotinib-SH (**1a**). Reagents and conditions: KO*t*Bu, THF, rt, 3 h, Ar, 97%.

Prior to a potential application in templated synthesis, we had to ascertain the affinity of the thiolated compounds for the Abl1 kinase. To illustrate a typically used route, nilotinib-SH (**1a**) was accessed in a 7-step convergent synthesis, in which the core benzanilide structure was established in organic solvents upon aminolysis of the methyl benzoate **4** in presence of potassium *tert*-butoxide as a strong base (Scheme 1 and S1†).^47^

**Scheme 2:**
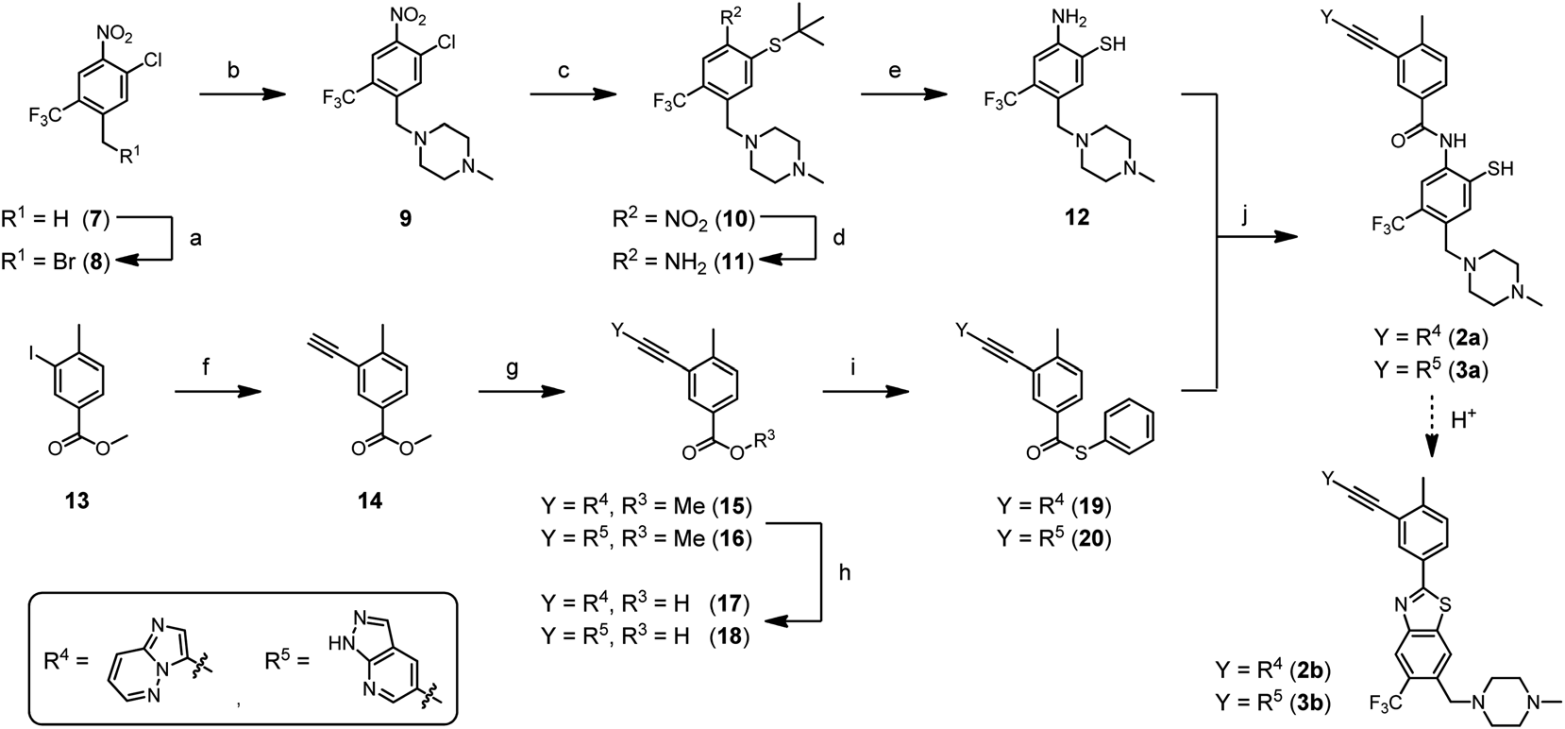
Synthesis of ponatinib-SH (**2a**) and GZD824-SH (**3a**) *via* NCL-type reaction. Reagents and conditions: (a) NBS, AIBN, AcOH, 80 °C, 26 h, 72%; (b) 1-methylpiperazine, DIPEA, DMF, rt, 1 h, 91%; (c) *t*BuSH, Cs_2_CO_3_, DMF, rt, 1 h, 83%; (d) (i) Na_2_S_2_O_4_, MeOH, H_2_O, rt, 1 h; (ii) conc. HCl, rt, 3 h, 86%; (e) TFMSA, TFA, thioanisole, rt, 30 min, 100%; (f) (i) ethynyltrimethylsilane, Pd(PPh_3_)_2_Cl_2_, CuI, NEt_3_, THF, rt, 17 h, Ar; (ii) K_2_CO_3_, MeOH, rt, 30 min, 88%; (g) 3-bromoimidazo[1,2-*b*]pyridazine or 5-bromo-1*H*-pyrazolo[3,4-*b*]pyridine, Pd(PPh_3_)_2_Cl_2_, CuI, DIPEA, DMF, 80 °C, 5 h, Ar, 99% **15**/75% **16**; (h) NaOH, MeOH, H_2_O, 60 °C, 3 h, 67% **17**/90% **18**; (i) thiophenol, HATU, DIPEA, DMF, 45 °C, 3 h, 100% **19**/56% **20**; (j) transfer buffer (4 M GnHCl, 100 mM NaH_2_PO_4_/Na_2_HPO_4_, 12.5 mM TCEP, pH 7.2), MeCN, 40 °C, 24 h, Ar.

For the synthesis of ponatinib-SH (**2a**) and GZD824-SH (**3a**), we designed a route depending on the milder NCL chemistry (Scheme 2). Nitroarene **7** was brominated at the benzylic position using *N*-bromosuccinimide, followed by the nucleophilic substitution of the bromine atom with 1-methylpiperazine. Then, the sulfur-containing moiety was introduced by nucleophilic aromatic substitution of the chlorine atom with 2-methylpropane-2-thiol, yielding thioether **10**. A subsequent reduction of the nitro group with sodium dithionite led to aniline derivative **11**. Acidolysis furnished the *o*-mercaptoaniline **12**, also termed acceptor fragment, in 47% overall yield. The thioester **19** was synthesized in four steps starting from methyl 3-iodo-4-methylbenzoate (**13**), which was submitted to the known sequence of two Sonogashira reactions for the introduction of the imidazo[1,2-*b*]pyridazine ethynyl appendage in **15**.^48^ After saponification, the benzoic acid derivative **17** was coupled with thiophenol, using HATU as a coupling agent, which furnished the desired thioester **19**, also termed donor fragment, in 58% overall yield. A similar approach was used for the synthesis of donor fragment **20**, which served as a precursor for the synthesis of GZD824-SH (**3a**).

We explored the *o*-mercaptoaniline **12** in NCL-type reactions with benzoic acid thioesters **19** and **20** in aqueous solution under neutral conditions (Scheme 2, step j). A phosphine (tris(2-carboxyethyl)phosphine, TCEP) was added to reduce potentially formed disulfides. The NCL reactions proceeded smoothly at 2.5 mM concentration of reactants and afforded the desired benzanilides **2a** and **3a** in up to 85% yield according to UPLC analysis of crude material. To our knowledge, this marks the first report of a reaction based on the NCL mechanism forming a benzanilide motif. The reaction proceeded without the need for further reagents, illustrating its potential for applications in fragment coupling reactions templated by nucleic acid or protein targets. First attempts to access nilotinib-SH (**1a**) by the NCL approach were not successful (data not shown) indicating a limited applicability of poorly reactive/soluble benzoyl thioesters. A noteworthy observation is that ponatinib-SH (**2a**) and GZD824-SH (**3a**) can be converted to the benzothiazole compounds ponatinib-BT (**2b**) and GZD824-BT (**3b**), respectively, by applying weak acidic conditions. Such diversification methods can be interesting in drug discovery processes.

Next, we assessed the affinity of the thiolated benzanilides and benzothiazoles for the Abl1 kinase using the KdELECT assay by DiscoverX (Table 1). In comparison to nilotinib (**1**) (*K*_d_ = 3.9 nM), the thiolated inhibitor nilotinib-SH (**1a**) (*K*_d_ = 881 nM) showed a markedly reduced affinity for the kinase. In stark contrast, thiolation of ponatinib (**2**) was well tolerated. The affinity of thiolated ponatinib-SH (**2a**) (*K*_d_ = 0.6 nM) was in the same range as the affinity of unmodified ponatinib (**2**) (*K*_d_ = 0.9 nM). Interestingly, the benzothiazole derivative ponatinib-BT (**2b**) (*K*_d_ = 8.4 nM) still offered a high affinity towards the Abl1 kinase. Of note, a viability assay confirmed the toxic effect of the ponatinib derivatives on the Bcr–Abl-positive cell line K562 (Table S1†). Similar results were obtained for the GZD824 derivatives, which despite thiolation showed subnanomolar *K*_d_ values too (GZD824 (**3**): *K*_d_ = 0.6 nM *vs.* thiolated GZD824-SH (**3a**): *K*_d_ = 0.9 nM). The differing effects of thiolation indicate that the Abl1 kinase binds nilotinib (**1**) in a slightly different mode than ponatinib (**2**) and GZD824 (**3**). Regardless of these differences, the data suggest that biologically active benzanilides can be formed by NCL chemistry.

**Table 1:** Dissociation constants (*K*_d_) of complexes formed with Abl1 kinase

Compound	*K*_d_ a [nM]
Nilotinib (**1**)	3.9 ± 0.3
Nilotinib-SH (**1a**)	881 ± 151
Ponatinib (**2**)	0.9 ± 0.5
Ponatinib-SH (**2a**)	0.6 ± 0.1
Ponatinib-BT (**2b**)	8.4 ± 2.3
GZD824 (**3**)	0.6 ± 0.3
GZD824-SH (**3a**)	0.9 ± 0.4

a The determination of the dissociation constants (*K*_d_) was performed with non-phosphorylated Abl1 kinase using the KdELECT assay (DiscoverX). The *K*_d_ values were calculated from quadruplicates.

**Scheme 3:**
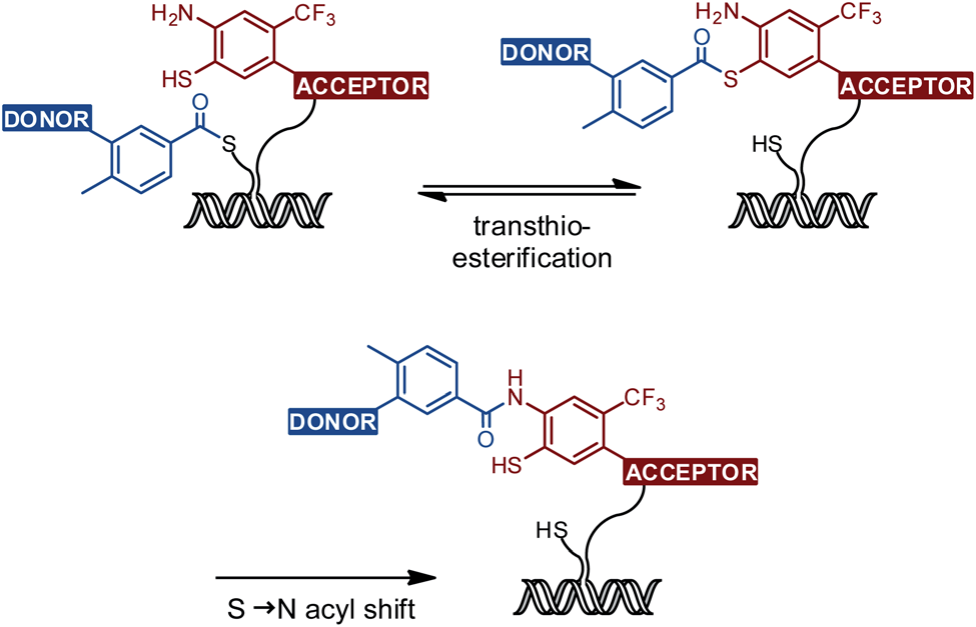
Schematic representation of the RNA-templated benzanilide formation *via* NCL-type reaction.

Encouraged by the activity of the thiolated TKI, we explored the templated benzanilide synthesis (Scheme 3). Specifically, we investigated whether RNA templates are able to instruct the synthesis of a TKI. The selected RNA template corresponded to a segment of mRNA coding for the targeted Bcr–Abl tyrosine kinase, which is a hallmark for a variety of different leukemias.^49^ This constitutively active kinase is formed due to a reciprocal translocation between the chromosomes 9 and 22 that results in the formation of the so-called Philadelphia chromosome.^50^ Various breakpoint regions within the Bcr gene exist, leading to different Bcr–Abl fusion transcripts and proteins.^49^ We envisioned a reaction system in which the recognition of the b3a2 fusion site, which is one of the two major transcript variants in CML, triggers the formation of a TKI.^51^

**Scheme 4:**
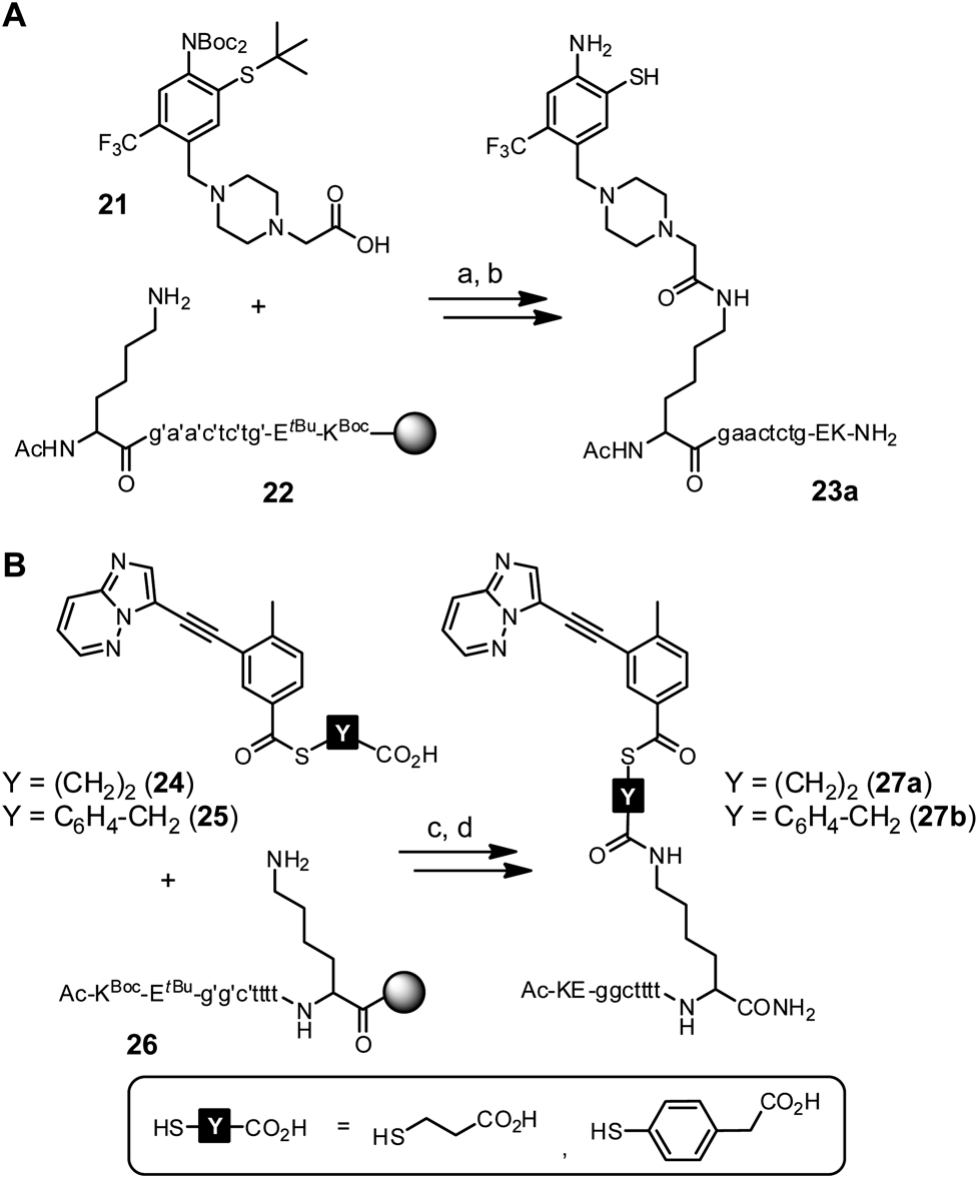
Synthesis of PNA-linked ponatinib fragments. (A) Acceptor conjugate. (B) Donor conjugates. Reagents and conditions: (a) HATU, NMM, DMF, rt, 30 min; (b) TFMSA, TFA, TIS, rt, 3 h; (c) HATU, NMM, NMP, rt, 30 min; (d) TFA, TIS, EDT, H_2_O, rt, 3 h. Apostrophe = Bhoc protecting group.

**Fig. 3:**
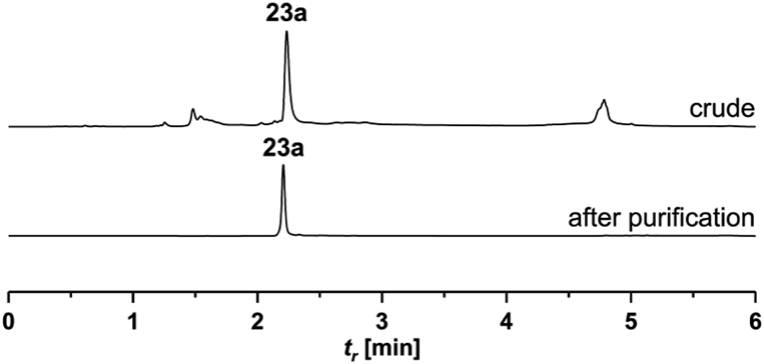
UPLC analysis of crude and HPLC-purified PNA acceptor conjugate **23a**.

First experiments towards the RNA-templated benzanilide formation were performed with PNA-linked fragments based on ponatinib (**2**) (Scheme S7†). We initially selected PNA as the recognition unit for the RNA template as it offers a high chemical and biological stability that would be advantageous for applications in live cells.^52,53^ Furthermore, the PNA-linked acceptor and donor conjugates are easily obtained *via* solid-phase synthesis (SPS). For conjugation of the *o*-mercaptoaniline fragment with PNA, the methylpiperazine residue was replaced by carboxymethylpiperazine (Scheme S4†). Crystal structure analysis shows that the methyl group is solvent-exposed when the inhibitor is bound to the kinase domain.^43^ In the last step of SPS, the carboxylic acid function of acceptor fragment **21** was coupled to the N-terminal lysine side chain of PNA resin **22** (Scheme 4A). During acid cleavage, we initially had anticipated problems due to side reactions at the electron-rich *o*-mercaptoaniline unit with benzhydryl cations generated from the Bhoc protecting groups. However, acidolysis proceeded smoothly in presence of triisopropylsilane as cation scavenger and the acceptor conjugate **23a** was obtained in 15% overall yield (Fig. 3).

Next, we explored access to PNA-linked benzoylation agents. Previously, aliphatic carboxylic acids were used for templated NCL.^11,21,36,39,40,54–67^ We expected a reduced reactivity of benzoic acid derivatives and examined the more reactive 4-mercaptophenylacetic acid (MPAA) esters in addition to 3-mercapto-propionic acid (MPA) esters (Scheme S5†).^68^ The corresponding donor fragments **24** and **25** were coupled to the C-terminal lysine side chain of PNA resin **26** using HATU as activating agent (Scheme 4B). Despite the basic conditions of the coupling reaction, no thioester hydrolysis was observed. After acidolytic cleavage, the donor conjugates **27a** and **27b** were obtained in 8–10% overall yield.

To investigate templated ponatinib synthesis, the MPA-derived benzoylation agent **27a** was incubated with *o*-mercaptoaniline segment **23a**. In the presence of equimolar amounts of RNA template (1.5 μM), neither product formation nor thioester hydrolysis was observed for the MPA-derived conjugate **27a**. In contrast, the MPAA-based thioester **27b** reacted smoothly with the PNA acceptor **23a**, yielding up to 85% of benzanilide product after 180 minutes (Fig. S20A†). Unfortunately, the non-templated reaction proceeded well too, affording 51% product.

The reaction rate in absence of template decreased at low conjugate concentration, suggesting that aggregation can likewise increase the effective molarity (Fig. S20B†). All reactions were monitored by UPLC analysis. The peak assignments were confirmed using mass spectrometry.

**Scheme 5:**
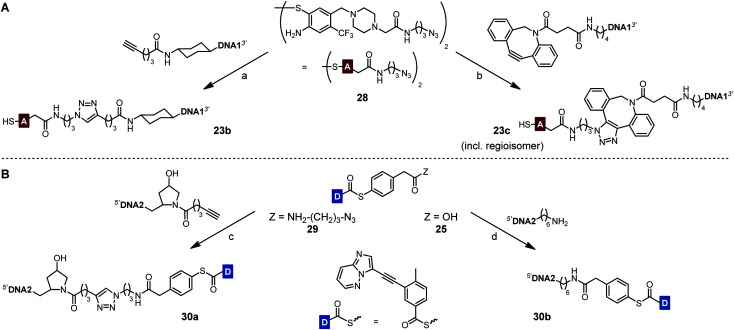
Synthesis of DNA-linked ponatinib fragments. (A) Acceptor conjugates. (B) Donor conjugates. Reagents and conditions: (a) (i) CuSO_4_, THPTA, sodium ascorbate, phosphate buffer (100 mM NaH_2_PO_4_/Na_2_HPO_4_, pH 7.4), H_2_O, DMSO, 30 °C, 3 h, Ar; (ii) TCEP, rt, 30 min, 28%; (b) (i) H_2_O, DMSO, rt, 22 h; (ii) TCEP, rt, 30 min, 32%; (c) CuSO_4_, THPTA, sodium ascorbate, phosphate buffer (100 mM NaH_2_PO_4_/Na_2_HPO_4_, pH 7.4), H_2_O, DMSO, 60 °C, 6 h, Ar, 37%; (d) HATU, DIPEA, H_2_O, DMF, rt, 4 h, 15%. DNA1 = 5′-TTGAACTCTGCTTAAATCCAG-3′. DNA2 = 5′-CCGCTGAAGGGCTT-3′.

To prevent undesired aggregation, we replaced the hydrophobic PNA part of the reactive conjugates by polyanionic DNA. Two DNA acceptor conjugates with different linker units were synthesized *via* either copper(i)-catalyzed (CuAAC) or strain-promoted azide–alkyne cycloaddition (SPAAC) using azide-modified acceptor fragment **28** and commercially available 5′-modified DNA oligonucleotides (Scheme 5A). While the linker length of the obtained conjugates **23b** and **23c** was almost identical, the azadibenzocyclooctene-based linker of **23c** was characterized by an increased hydrophobicity and rigidity compared to the triazole-derived linker of **23b**. In addition, two benzoyl donor conjugates were synthesized by coupling the benzoic acid thioesters **29** and **25** to 3′-modified DNA oligonucleotides using CuAAC or amide bond formation (Scheme 5B). The conjugates **30a** and **30b** offered different flexibilities, controlled by the length of the linker unit. Due to the lack of reactivity of the MPA-derived thioester in our previous experiments with PNA conjugates, only DNA donor conjugates based on MPAA were synthesized.

**Scheme 6:**
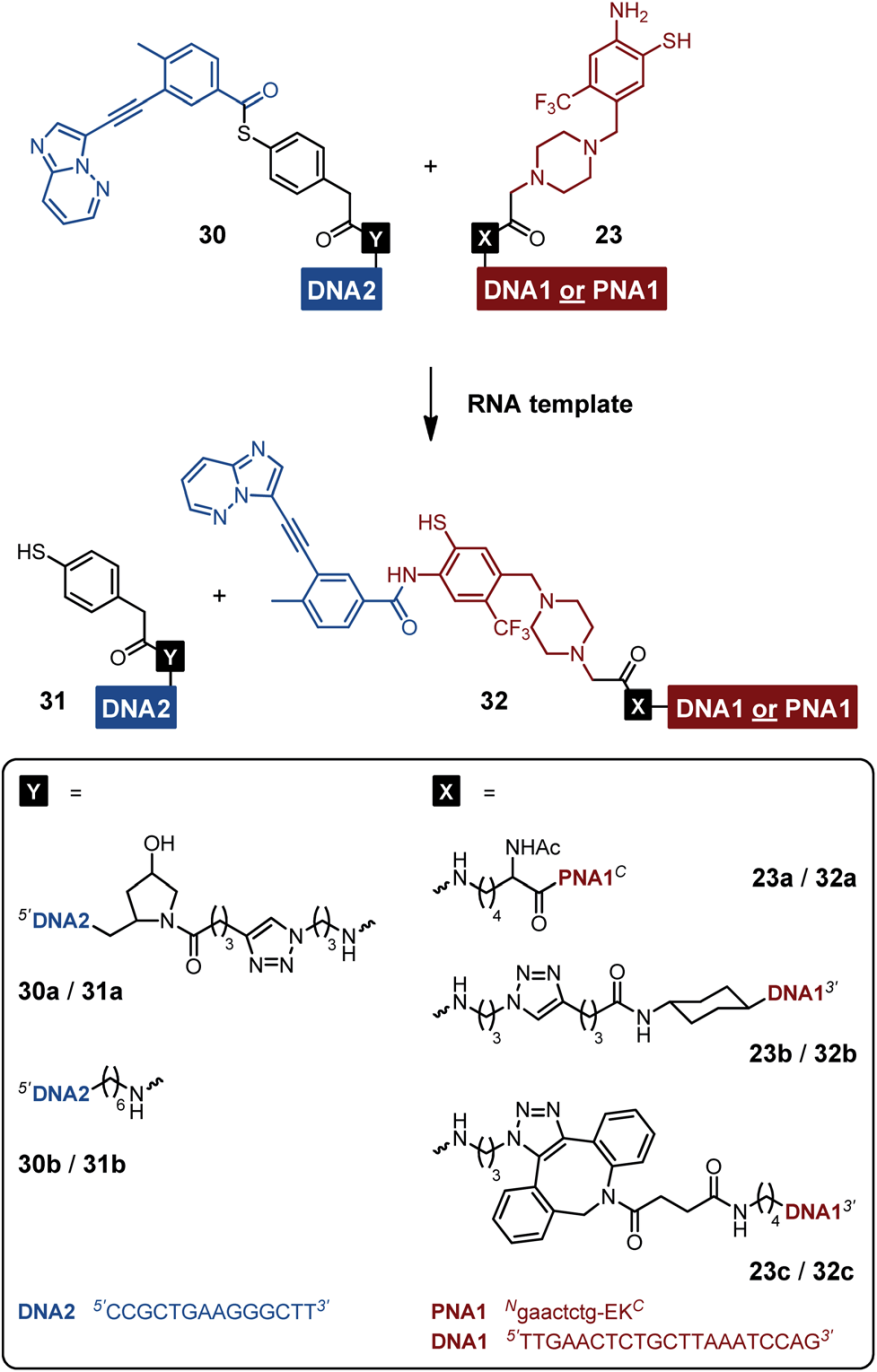
RNA-templated benzanilide formation *via* NCL-type reaction between the various acceptor and donor conjugates.

**Fig. 4:**
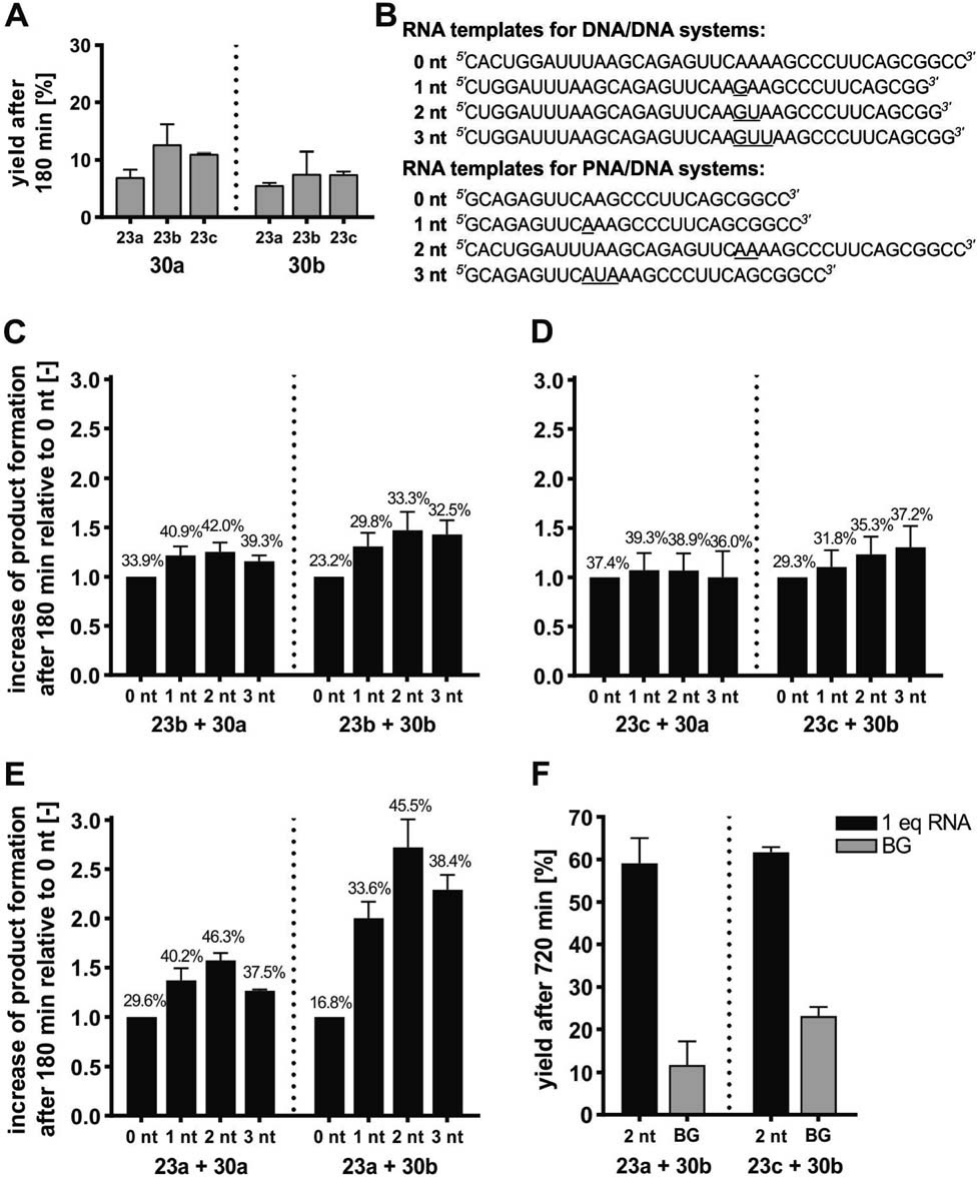
(A) Product yields after 180 min for background reactions of DNA donor conjugates **30a** or **30b** with acceptor conjugates **23a** –**23c**. (B–E) Fold change of product formation upon introduction of unpaired template nucleotides (underlined in B) relative to seamless annealing (0 nt) for transfer reactions of DNA donor conjugates **30a** or **30b** with DNA acceptor conjugate **23b** (C), DNA acceptor conjugate **23c** (D), or PNA acceptor conjugate **23a** (E). Error bars show the standard deviation of triplicate measurements. For details, see Tables S3–S6.† Mean yields given in numbers over the bars. (F) Product yields after 720 min for the indicated transfer reactions in presence and absence of RNA template. Conditions: 10 mM MOPS, 200 mM NaCl, 2 mM TCEP, pH 7.2, [A] = 1 μM, [D] = 2 μM, [RNA] = 0/1 μM, 37 °C. BG = background; nt = nucleotides.

Each of the three *o*-mercaptoaniline conjugates was incubated with one of the two DNA-linked benzoylation agents (Scheme 6). The reactions were performed as independent triplicates at 1 μM concentration of acceptor and, in anticipation of competing hydrolysis, a twofold excess of benzoylating agents **30a** or **30b**. Without template, the ponatinib conjugates were formed in 6–13% yield after 180 minutes (Fig. 4A, see also Table S2†). The comparison with the 51% product generated in reactions involving the PNA-linked benzoyl donor illustrates that the use of DNA-linked donors helped alleviate aggregation. For reactions of the DNA-linked benzoyl donor agent **30a**, background was lowest when the DNA donor conjugates were incubated with PNA-linked acceptor **23a** rather than DNA-linked acceptors **23b** or **23c**. We consider this observation as interesting given that previously described templated reactions involved DNA-only or PNA-only systems. Differences of background rates were less obvious with conjugate **30b**, in which the benzoic acid thioester was linked *via* a shorter linker than in **30a**.

In a next step, we examined the influence of the template and varied the number of unpaired template nucleotides between the aligned reactive conjugates. Upon seamless annealing a template effect was observed for each reaction system (see yields on 0 nt bars in Fig. 4C–E). Compared to 0 nt, gap sizes between 1–3 nt accelerated the templated reaction (Fig. 4C–E). However, the fold change of yields varied little (3.3–5.4% variation of the mean) when DNA-linked benzoyl donors **30a** or **30b** were co-aligned with DNA-linked acceptor conjugates **23b** or **23c** in 1–3 nt distance (Fig. 4C and D). Given the standard deviation, we considered these changes insignificant and inferred that the size of the gap had little influence. By contrast, a comparatively pronounced dependency of transfer yields on the gap size was observed for reactions involving the PNA-linked acceptor **23a** (Fig. 4E). In this case, a 2 nt gap proved optimal. Templates that allowed for seamless hybridization (gap size = 0 nt) provided the lowest reactivity (16.8 ± 1.7% for reaction between **23a** and **30b**), which was more than doubled when the reactions were allowed to proceed on a template offering a two-nucleotide gap (45.5 ± 3.0% for reaction between **23a** and **30b**). At present, we cannot explain why reactions between a PNA and a DNA conjugate show a higher distance dependency than reactions between two DNA conjugates. In previous acyl transfer reactions using PNA-linked conjugates, we also observed a pronounced dependency of rates on the number of unpaired template nucleotides between the aligned functional groups.^40,59^ Similar to the herein described templated benzoylation, comparably low reactivity was observed upon seamless alignment. We speculate that end-fraying of PNA-containing duplexes is reduced, and that additional flexibility provided by unpaired template nucleotides is required for productive encounters of the aligned reactive groups. In line with this assumption, benzoyl donor conjugate **30b** with the shorter linker proved more susceptible to variations of gap size than **30a**.

To improve the yields of templated benzanilide formation, we increased the reaction time (Fig. 4F). After 12 hours, the DNA-based reaction partners **23c** and **30b** provided 61.7 ± 1.0% product, which surpassed the yield in absence of template by 38.5% (Table S5†). A similar yield was obtained with the mixed PNA/DNA system (**23a** and **30b**). More importantly, the non-templated reaction was less productive, resulting in a desirable surplus of 47.3% on the account of the templated reaction. A comparison with previously described DNA-templated benzoyl transfer reactions is instructive. Stulz and Turberfield analyzed the templated transfer of a TAMRA group that was linked to DNA as a benzoic acid thioester.^69^ The reactions with various aliphatic amines, alkoxyamines, and hydrazides afforded less than 15% of the desired products after 48 hours. The 59% yield after only 12 hours observed with the **23a**/**30b** reaction system, involving a less reactive aromatic amine, points to the advantages provided by the NCL-type reaction. The underlying mechanism was confirmed by control experiments involving thiol-protected or thiol-free PNA acceptor conjugates (Schemes S8, S9, Fig. S24 and S25†). In these cases, no transfer product was formed within 6 or 12 hours, respectively. We conclude that the RNA-templated benzanilide formation represents a useful extension of the scope of nucleic acid-templated transfer reactions, yielding structural motifs that have eluded access by mild (and metal-free) chemistry so far.

## Conclusions

In conclusion, we developed a reaction that provides access to benzanilides and benzothiazoles under mild conditions and allows bond formation in presence of other biomolecules. The amide bond forming reaction relies on a NCL-type mechanism, which involves a benzoic acid thioester and an *o*-mercaptoaniline. We applied the reaction to the synthesis of ponatinib and GZD824 derivatives and showed that the small molecules bound/inhibited Abl1 tyrosine kinase at the 10^−1^ nanomolar range. The mildness of the fragment coupling conditions and the possibility to perform reactions in absence of activating agent should be an asset for fragment-based drug discovery approaches. It is conceivable that benzanilide formation can be put under the control of a protein target, which by means of a templated reaction would instruct the formation of its own inhibitor. We explored nucleic acid-templated synthesis of ponatinib derivatives. A comparative study revealed high rates of non-templated product formation for reactions between PNA-based reactive conjugates, which are probably caused by aggregation. Given the hydrophobic nature of the ponatinib fragments, this result points to potential pitfalls when hydrophobic PNA is used in templated synthesis. The use of two DNA-based conjugates or mixed systems comprising a PNA acceptor conjugate and a DNA donor conjugate provided a solution to the problem of high reactivity in absence of template. Under optimal conditions, the templated reaction between the DNA-linked benzoic acid thioester and a weakly nucleophilic aniline–PNA conjugate proceeded in 59% yield without side reactions other than thioester hydrolysis. These reactions were susceptible to changes of the template architecture, which afforded highest reactivity when the reactive groups were separated by two unpaired template nucleotides. Of note, the size of this gap was less important in reactions involving two DNA conjugates. Our study also confirmed results of previous reports describing that the template-induced reactivity is highest when the reactive groups are connected to DNA *via* short linkers. The presented reaction systems based on DNA conjugates could find application in drug candidate screenings applying DEL.

## Data availability

The datasets supporting this article have been uploaded as part of the ESI.†

## Author contributions

O. S. formulated the research goals and aims. R. H. and M. B. S. conducted the research and developed methodology. M. B. S. validated data as a part of replication of experiments. R. H. and M. B. S. contributed to visualization of data. O. S. and R. H. wrote the original draft. All authors contributed to reviewing and editing.

## Conflicts of interest

There are no conflicts to declare.

## Supplementary Material

SC-012-D1SC00513H-s001
