# Supplementary material for: Expanding the scope of native chemical ligation – templated small molecule drug synthesis via benzanilide formation
Source: Chem Sci. 2021 Sep 14;12(40):13450–7. doi: 10.1039/d1sc00513h (PMC8528049; doi:10.1039/d1sc00513h)
Supplement: SC-012-D1SC00513H-s001 [file SC-012-D1SC00513H-s001.pdf]

Electronic Supplementary Material (ESI) for Chemical Science.
This journal is © The Royal Society of Chemistry 2021

Supporting Information

Materials and methods 2
Synthesis of nilotinib-SH 7
Synthesis of ponatinib-SH, ponatinib-BT, GZD824-SH, and GZD824-BT 12
Synthesis of ponatinib-SH fragments 24
Synthesis of PNA conjugates 34
Synthesis of DNA conjugates 39
Determination of dissociation constants 45
Determination of half-maximal inhibitory concentrations 47
Determination of molar extinction coefficients 48
Determination of melting temperatures 49
Synthetic RNAs 50
Transfer reactions of PNA/PNA systems 51
Transfer reactions of DNA/DNA and PNA/DNA systems 53
Control experiment using a thiol-protected PNA acceptor conjugate 58
Control experiment using a thiol-free PNA acceptor conjugate 59
NMR spectra 60
Abbreviations 98
References 98

Materials and methods

Materials

Reagents for organic synthesis were purchased from abcr (Karlsruhe, Germany), Acros Organics (Geel, Belgium), Alfa Aesar (Haverhill, USA), Merck (Darmstadt, Germany), Sigma Aldrich (St. Louis, USA), TCI (Tokyo, Japan) and VWR (Radnor, USA). All reagents were used without any further purification. Dry solvents were obtained by dynamic drying using a MB SPS-800 system from MBraun (Garching, Germany). Water was purified using a Milli-Q Ultra-Pure Water Purification system from membraPure (Henningsdorf, Germany). Flash column chromatography was performed with silica gel from Machery-Nagel (Dueren, Germany) and silica gel C18 from Carl Roth (Karlsruhe, Germany). Nilotinib, ponatinib, and GZD824 were purchased from Selleck Chemicals (Houston, USA). Fmoc/Bhoc-protected PNA monomers were purchased from Link Technologies (Bellshill, Scotland) and Fmoc-protected amino acids from Iris Biotech GmbH (Marktredwitz, Germany). HATU, HCTU, and TFA were obtained from Carl Roth (Karlsruhe, Germany), HOBt from Carbolution Chemicals (St. Ingbert, Germany), DMF and acetonitrile from VWR (Radnor, USA). The resin TentaGel R RAM was purchased from Rapp Polymere (Tuebingen, Germany). Amino-, alkyne-, and DBCO-modified DNA oligonucleotides, as well as synthetic RNAs were synthesized and purified by Biomers (Ulm, Germany). The CML cell line K562 was obtained from DSMZ (Braunschweig, Germany). Dulbecco's Modified Eagle Medium (DMEM) and fetal calve serum (FCS), as well as alamarBlue™ reagent and penicillin-streptomycin mix were purchased from Thermo Fisher Scientific (Waltham, USA).

NMR spectroscopy

NMR spectra were recorded on Avance II 300 (^{1}H = 300.13 MHz, ^{19}F = 282.40 MHz), Avance II 400 (^{1}H = 400.13 MHz, ^{13}C = 100.61 MHz), and Avance III 500 (^{1}H = 500.13 MHz, ^{13}C = 125.76 MHz, ^{19}F = 470.59 MHz) spectrometers from Bruker (Billerica, USA). All measurements were carried out at room temperature. Chemical shifts (δ) are reported as parts per million (ppm) with the solvent signal as internal standard for ^{1}H ($CDCl_3$ = 7.26 ppm, DMSO-d_6 = 2.50 ppm, methanol-d_4 = 3.31 ppm) and ^{13}C NMR ($CDCl_3$ = 77.16 ppm, DMSO-d_6 = 39.52 ppm, methanol-d_4 = 49.00 ppm). Fluorine spectra were calibrated with an external standard ($CFCl_3$ = 0.00 ppm). Carbon spectra were recorded with complete proton decoupling. Signals in the ^{13}C and ^{19}F NMR represent singlets, unless stated otherwise. Data are reported as follows: chemical shift (δ) in ppm (multiplicity, coupling constants (J) in Hz, number of protons determined by integration). The multiplicity of the signals is specified with the following abbreviations: br. s (broad singlet), d (doublet), dd (doublet of doublets), ddd (doublet of doublets of doublets), m (multiplet), q (quartet), quin (quintet), s (singlet), t (triplet).

Determination of molar extinction coefficients

The molar extinction coefficients ($\varepsilon_{260,exp.}$) of inhibitor fragments and inhibitor derivatives were determined on a V-750 UV/Vis spectrometer from Jasco (Pfungstadt, Germany). For each compound, 5 stock solutions of defined concentration were prepared (1 – 5 mg in 10 mL DMF) and subsequently diluted with buffer (100 mM NaH_2PO_4/Na_2HPO_4, 0.2% w/v CHAPS, pH 7.4) and DMF, yielding a 1:20 and a 1:40 dilution of each stock solution (10% final concentration of DMF). The blank corrected absorbance at 260 nm was measured as triplicate for each dilution. Molar extinction coefficients

($\varepsilon_{260,exp.}$) were determined by linear regression after plotting the averaged absorbance at 260 nm against the concentration. The obtained data were rounded to the nearest hundred.

Solid-phase synthesis (SPS)

PNA conjugates were prepared by automated Fmoc-SPS. The syntheses were performed in 2 to 5 µmol scale with a MultiPep RS synthesizer from Intavis (Cologne, Germany) using microscale columns and rink-amide resin Tentagel R RAM (0.2 mmol · g^{-1}). Prior to conjugate synthesis, the resin was swelled in DMF for 30 min. The Fmoc protecting group was removed by treatment with piperidine in DMF (1:4, v/v; 1 x 3 min, 1 x 7 min) and the resin was washed with DMF (8 x) afterwards. PNA conjugates were synthesized by sequential coupling of Fmoc-protected PNA monomers (4 eq) in DMF (0.2 M) in the presence of HCTU (3.6 eq) and NMM (8 eq) or Fmoc-protected amino acids (4 eq) in DMF (0.2 M) in the presence of HCTU (3.6 eq), HOBt (4 eq), and NMM (8 eq) for 20 min at room temperature. Amino acids were coupled twice. The resin was washed with DMF (3 x) and unreacted amino groups were capped by treatment with a mixture of acetic anhydride/2,6-lutidine/DMF (5:6:89, v/v/v; 2 x 2 min). Prior to the next coupling cycle, the resin was washed with DMF (4 x). After the synthesis of the PNA oligomer, the inhibitor fragment was coupled to the side chain of the *N*- or *C*-terminal lysine according to the described procedure. Subsequently, the resin was washed with DCM (3 x), dried under vacuum, and treated with a cleavage cocktail of TFA/TIS/EDT/water (92.5:2.5:2.5:2.5, v/v/v/v; donor conjugate) or TFA/TIS/TFMSA (80:12:8, v/v/v; acceptor conjugate) for 3 h at room temperature. After filtration and partial evaporation of the solvent, the PNA conjugate was precipitated in ice-cold diethyl ether. The precipitate was dried under argon, dissolved in water, and purified by preparative HPLC.

HPLC purification

PNA conjugates and certain small molecules were purified using a 1100 HPLC system from Agilent (Santa Clara, USA) with a Nucleodur C18 Gravity column (110 Å, 5 µm, 10 · 250 mm) from Machery-Nagel (Dueren, Germany). A binary mixture of solvents A1 (98.9% water, 1.0% MeCN, 0.1% TFA) and B1 (98.9% MeCN, 1.0% water, 0.1% TFA) was used as mobile phase, unless stated otherwise. Chromatographic separations were performed at room temperature, with a constant flow rate of 6 mL · min^{-1} and linear gradients. The absorbance was measured at 210 nm and 260 nm.

DNA conjugates were purified using a 1105 HPLC system from Gilson (Limburg, Germany) with a XBridge BEH C18 column (130 Å, 5 µm, 10 · 100 mm) from Waters (Milford, USA). A binary mixture of solvents A2 (0.1 M triethylammonium acetate, pH 7.2) and B2 (MeCN) was used as mobile phase. Chromatographic separations were performed at 50 °C with a constant flow rate of 7 mL · min^{-1} and linear gradients. The absorbance was measured at 210 nm and 260 nm.

After HPLC purification, pure product fractions were pooled and concentrated to dryness using a VaCo2 lyophilizer from Zirbus (Bad Grund, Germany). PNA and DNA conjugates were stored at -20 °C.

UPLC analysis

UPLC-MS analysis of PNA conjugates and small molecules was performed on an ACQUITY UPLC H-Class system with ACQUITY QDA Performance detector from Waters (Milford, USA). Chromatographic separation was achieved with an ACQUITY UPLC CSH C18 column (130 Å, 1.7 µm, 2.1 · 50 mm, column 1) heated to 50 °C and a constant flow rate of 0.5 mL · min^{-1}. A binary mixture of solvents A1 (98.9% water, 1.0% MeCN, 0.1% TFA) and B1 (98.9% MeCN, 1.0% water, 0.1% TFA) was used as mobile

phase. The absorbance was measured at 260 nm. Mass spectra were obtained by electron spray ionization (ESI) using a quadrupole in positive mode. The data are reported as mass units per charge ($m \cdot z^{-1}$).

UPLC-UV/Vis analysis of DNA conjugates was performed on an ACQUITY UPLC system with ACQUITY UPLC TUV detector from Waters (Milford, USA). The chromatographic separation was achieved with an ACQUITY UPLC BEH Oligonucleotide C18 column (130 Å, 1.7 µm, 2.1 · 50 mm, column 2) heated to 70 °C and a constant flow rate of 0.5 mL · min^{-1}. A binary mixture of solvents A2 (0.1 M triethyl-ammonium acetate, pH 7.2) and B2 (MeCN) was used as mobile phase. The absorbance was measured at 260 nm.

MALDI-TOF mass spectrometry

MALDI-TOF mass spectra were measured on an AXIMA Confidence spectrometer from Shimadzu (Kyoto, Japan). A 10:1 mixture of 3-hydroxypicolinic acid (50 mg · mL^{-1} in water/MeCN, 1:1, v/v) and diammonium hydrogen citrate (100 mg · mL^{-1} in water) was used as matrix. The MALDI-TOF mass spectra were recorded in positive linear mode. The data are reported as mass units per charge ($m \cdot z^{-1}$).

Determination of PNA and DNA conjugate concentration

The concentration of PNA and DNA conjugates in aqueous solution was determined by measuring the absorbance at 260 nm with a NanoDrop ND-1000 UV/Vis spectrometer from Thermo Fischer Scientific (Waltham, USA). The molar extinction coefficients (ε_{260}) of PNA conjugates were calculated according to equation 1, for DNA conjugates equation 2 was used.

$$(1) \quad \varepsilon_{260} = \mathrm{n} \cdot \varepsilon_{260}(A) + \mathrm{m} \cdot \varepsilon_{260}(C) + \mathrm{l} \cdot \varepsilon_{260}(G) + \mathrm{k} \cdot \varepsilon_{260}(T) + \varepsilon_{260,exp.}$$

$$(2) \quad \varepsilon_{260} = \varepsilon_{260}(ON) + \varepsilon_{260,exp.}$$

$\varepsilon_{260}(A/C/G/T)$ = molar extinction coefficients of the nucleobases at 260 nm in L · mol^{-1} · cm^{-1} ($\varepsilon_{260}(A)$ = 13700, $\varepsilon_{260}(C)$ = 6600, $\varepsilon_{260}(G)$ = 11700, $\varepsilon_{260}(T)$ = 8800), $\varepsilon_{260}(ON)$ = molar extinction coefficient of the DNA oligonucleotide at 260 nm in L · mol^{-1} · cm^{-1} according to manufacturer, $\varepsilon_{260,exp.}$ = molar extinction coefficient of the attached inhibitor fragment or modified inhibitor at 260 nm in L · mol^{-1} · cm^{-1}, n/m/l/k = number of nucleobases – A is adenine, C is cytosine, G is guanine, and T is thymine.

Determination of melting temperatures

The melting curves were recorded on a V-750 UV/Vis spectrometer from Jasco (Pfungstadt, Germany). Solutions containing PNA or DNA conjugate (1 µM) and complementary RNA (1 µM) in phosphate buffer (10 mM NaH_2PO_4/Na_2HPO_4, 100 mM NaCl, pH 7.4) were prepared and the absorbance at 260 nm was measured during a thermal cycle from 25 to 90 °C using a heating rate of 0.5 °C · min^{-1}. Melting temperatures (T_m) were taken as the maximum of the first derivatives of the Boltzmann equations fitted to the acquired melting curves. Each sample was measured in triplicate.

Determination of dissociation constants

Dissociation constants (K_d) were determined by DiscoverX (Fremont, USA) with non-phosphorylated Abl1 kinase using the KdELECT® assay. K_d values were calculated from quadruplicates with an 11-point dose-response curve using the Hill equation. A detailed procedure can be found in the literature.[1]

Cell culture and cell viability assay

K562 cells were cultured in DMEM supplemented with 10% FCS, 4 mM glutamine, 100 U · mL^{-1} penicillin, and 100 µg · mL^{-1} streptomycin in a 37 °C humidified chamber with 5% CO_2. For viability assays, cells were seeded on a 96-well plate (10000 cells/well), followed by the immediate addition of the test compounds (100 µL, serial dilutions in medium). Untreated cells were used as controls and complete medium without cells was used to determine the background signal of the assay. After incubation at 37 °C, 5% CO_2 for 96 h, cells were washed with PBS and alamarBlue™ reagent (100 µL, 1:10, v:v, in PBS) was added to each well. After further incubation at 37 °C, 5% CO_2 for 2 h, the fluorescence intensity was measured (λ_{Ex} = 531 nm, λ_{Em} = 590 nm) on a Victor X5 multilabel plate reader from PerkinElmer (Waltham, USA). IC_{50} values were calculated from triplicates with a 13-point dose-response curve using the Hill equation.

RNA-templated benzanilide formation

RNA-templated transfer reactions were performed in freshly prepared, degassed, and argon-saturated buffer (10 mM MOPS, 100 mM NaCl, 2 mM TCEP, pH 7.2). For the PNA/PNA system, 0.2% w/v CHAPS were added to increase the solubility of the PNA donor conjugate. The sequences of synthetic RNAs used as templates are reported at page 50.

For transfer reactions using the PNA/PNA system, 1.7 mL SafeSeal microcentrifuge tubes (low binding polymer technology) from Sorenson BioScience (Salt Lake City, USA) were used. The acceptor conjugate and the RNA template were dissolved in buffer with twice the target concentration and left at room temperature for 10 min to reduce potentially formed disulfides prior to the reaction. In another vessel, the donor conjugate was dissolved in buffer with twice the target concentration. To start the transfer reaction, both solutions were combined and thoroughly mixed. The reaction mixture was agitated at 37 °C for 3 h. After the indicated reaction times, aliquots of 25 µL were taken and added to 0.25 µL of TFA in 250 µL polypropylene inserts from Sigma Aldrich (St. Louis, USA) to quench the reaction, followed by UPLC-MS analysis. The template-independent transfer reaction (background) was performed analogously in the absence of RNA template.

For transfer reactions using the DNA/DNA or PNA/DNA system, 200 µL glass inserts from Machery-Nagel (Dueren, Germany) were used. The acceptor conjugate and the RNA template were dissolved in buffer with the desired target concentration and left at room temperature for 10 min to 7 h to reduce potentially formed disulfides prior to the reaction. Afterwards, the required volume of a donor conjugate stock solution was added to start the transfer reaction. The reaction mixture was thoroughly mixed, placed in the sample manager of an ACQUITY UPLC system from Waters (Milford, USA), and heated to 37 °C for 3 to 12 h. After the indicated reaction times, UPLC-UV/Vis analysis was performed by injecting aliquots directly from the reaction mixture and without prior quenching. The template-independent transfer reaction (background) was performed analogously in the absence of RNA template.

To determine the reaction kinetics, aliquots were analyzed by UPLC-MS or UPLC-UV/Vis at 260 nm. The assignment of peaks was accomplished by ESI-MS or through comparison of retention times to synthetic reference compounds. The obtained chromatograms were integrated and the transfer yields were calculated according to equation 3. The experiments were performed in triplicate. The yields were plotted against the reaction time and graphs were fitted through non-linear regression using the Michaelis-Menten equation.

(3) $$yield\ [\%] = \frac{\frac{s_{product}}{\varepsilon_{product}}}{\left(\frac{s_{acceptor}}{\varepsilon_{acceptor}} + \frac{s_{product}}{\varepsilon_{product}}\right)} \cdot 100\%$$

$s_{product}$ = area of product conjugate, $s_{acceptor}$ = area of acceptor conjugate, $\varepsilon_{product}$ = molar extinction coefficient of product conjugate at 260 nm in $L \cdot mol^{-1} \cdot cm^{-1}$, $\varepsilon_{acceptor}$ = molar extinction coefficient of acceptor conjugate at 260 nm in $L \cdot mol^{-1} \cdot cm^{-1}$.

Synthesis of nilotinib-SH

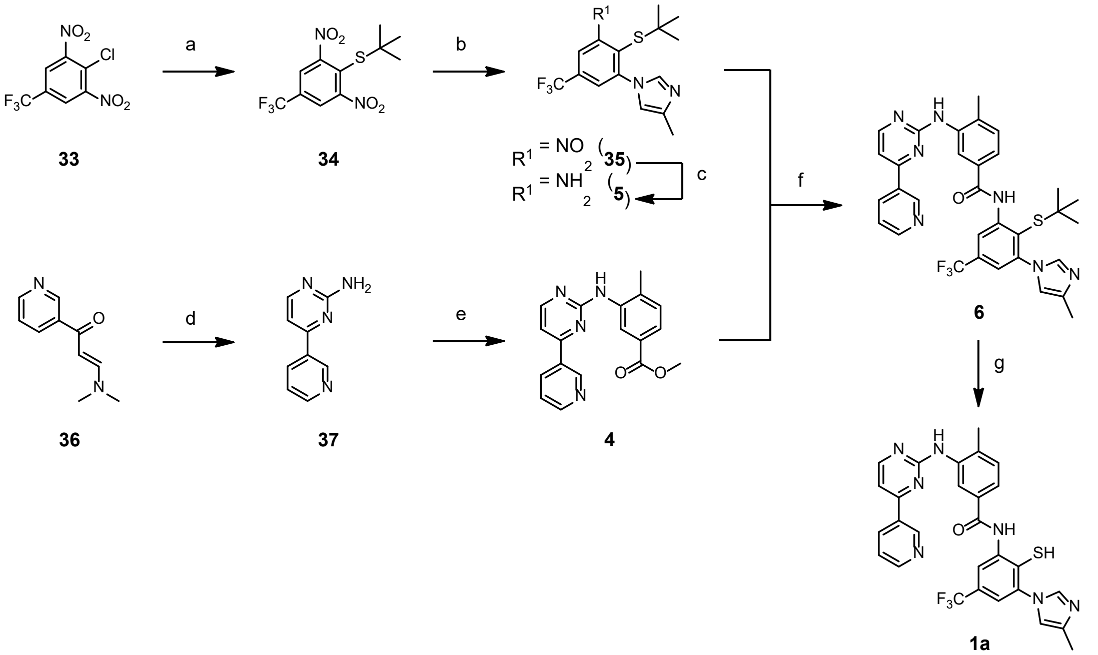

Scheme S1: Synthesis route towards nilotinib-SH (**1a**). *Reagents and conditions:* (a) *t*BuSH, Cs_2CO_3, DMF, rt, 30 min, *94%*; (b) 4-methyl-1*H*-imidazole, Cs_2CO_3, DMF, 60 °C, 3 h, *57%*; (c) $Na_2S_2O_4$, MeOH, H_2O, rt, 1 h, *36%*; (d) GnHCl, NaOH, *n*BuOH, reflux, 7 h, *95%*; (e) methyl 3-iodo-4-methylbenzoate, $Pd_2(dba)_3$, XantPhos, Cs_2CO_3, 1,4-dioxane, reflux, 3 h, Ar, *90%*; (f) KO*t*Bu, THF, rt, 3 h, Ar, *97%*; (g) TFMSA, TFA, thioanisole, rt, 30 min, *56%*.

2-(*tert*-Butylthio)-1,3-dinitro-5-(trifluoromethyl)benzene (34)

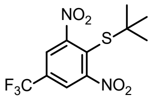

2-Methylpropane-2-thiol (4.38 mL, 38.8 mmol, 1.05 eq) and Cs_2CO_3 (24.08 g, 73.92 mmol, 2.0 eq) were added to a solution of 2-chloro-1,3-dinitro-5-(trifluoromethyl)benzene (**33**) (10.00 g, 36.96 mmol) in DMF (200 mL) under thoroughly stirring. After 30 min at room temperature, water (20 mL) was added to dissolve excess Cs_2CO_3 and the reaction mixture was extracted with EtOAc (3 x 100 mL). The combined organic extracts were washed with 1 M HCL (1 x 50 mL), water (1 x 50 mL), and brine (1 x 50 mL), dried over $MgSO_4$, and concentrated in vacuo. Following purification by flash column chromatography (silica, cyclohexane/EtOAc, 2:1) yielded the pure product as a yellow solid.

11.28 g (34.78 mmol, 94%), $C_{11}H_{11}F_3N_2O_4S$ (324.28 g · mol^{-1}).

^{1}H NMR (500 MHz, $CDCl_3$): δ [ppm] = 8.05 – 8.01 (m, 2H), 1.29 (s, 9H).

^{13}C NMR (126 MHz, $CDCl_3$): δ [ppm] = 157.4, 133.8 (q, J = 36.2 Hz), 125.3, 122.6 (q, J = 3.5 Hz), 121.6 (q, J = 273.9 Hz), 54.2, 31.7.

^{19}F NMR (282 MHz, $CDCl_3$): δ [ppm] = -63.41.

1-(2-(*tert*-Butylthio)-3-nitro-5-(trifluoromethyl)phenyl)-4-methyl-1*H*-imidazole (35)

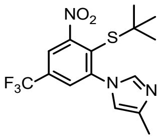

To a solution of dinitroarene **34** (10.00 g, 30.84 mmol) was added 4-methyl-1*H*-imidazole (2.78 g, 33.9 mmol, 1.1 eq) and Cs_2CO_3 (20.10 g, 61.68 mmol, 2.0 eq). After 6 h at 60 °C, water (20 mL) was added to dissolve excess Cs_2CO_3 and the reaction mixture was extracted with EtOAc (3 x 100 mL). The combined organic extracts were washed with water (3 x 50 mL) and brine (1 x 50 mL), dried over $MgSO_4$, and concentrated in vacuo. Following purification by flash column chromatography (silica, cyclohexane/EtOAc, 10:1 to 2:1 + 0.5% NEt_3) yielded the pure product as a yellow oil.

6.27 g (17.45 mmol, 57%), $C_{15}H_{16}F_3N_3O_2S$ (359.37 g · mol^{-1}).

ESI-MS: $m \cdot z^{-1}$ = 360.3 [M+H]$^+$ (calc.: 360.1).

^{1}H NMR (500 MHz, $CDCl_3$): δ [ppm] = 8.20 – 8.16 (m, 1H), 8.09 – 8.04 (m, 1H), 7.53 (d, *J* = 1.2 Hz, 1H), 6.68 – 6.62 (m, 1H), 2.29 (d, *J* = 1.0 Hz, 3H), 1.23 (s, 9H).

^{13}C NMR (126 MHz, $CDCl_3$): δ [ppm] = 148.0, 139.2, 138.0, 137.7 (q, *J* = 3.4 Hz), 137.4, 135.9, 131.4 (q, *J* = 34.5 Hz), 122.2 (q, *J* = 273.1 Hz), 121.6 (q, *J* = 3.4 Hz), 117.2, 49.6, 31.1, 13.5.

^{19}F NMR (282 MHz, $CDCl_3$): δ [ppm] = -63.28.

1-(3-Amino-2-(*tert*-butylthio)-5-(trifluoromethyl)phenyl)-4-methyl-1*H*-imidazole (5)

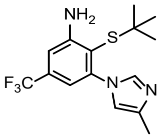

Sodium dithionite (27.02 g, 155.0 mmol, 10.0 eq) was added portionwise to a suspension of nitroarene **35** (5.58 g, 15.5 mmol) in MeOH (60 mL) and water (60 mL). After 1 h at room temperature, brine (20 mL) was added and the reaction mixture was extracted with EtOAc (4 x 50 mL). The combined organic extracts were dried over $MgSO_4$ and concentrated in vacuo. Following purification by flash column chromatography (silica, DCM/MeOH, 50:1) yielded the pure product as a colorless solid.

1.86 g (5.65 mmol, 36%), $C_{15}H_{18}F_3N_3S$ (329.38 g · mol^{-1}).

ESI-MS: $m \cdot z^{-1}$ = 330.2 [M+H]$^+$ (calc.: 330.1).

^{1}H NMR (500 MHz, $CDCl_3$): δ [ppm] = 7.45 (d, *J* = 1.2 Hz, 1H), 7.31 – 7.27 (m, 1H), 7.03 – 6.99 (m, 1H), 6.69 – 6.64 (m, 1H), 3.95 (s, 2H), 2.32 (d, *J* = 0.9 Hz, 3H), 1.21 (s, 9H).

^{13}C NMR (126 MHz, $CDCl_3$): δ [ppm] = 144.4, 138.8, 137.5, 134.2, 131.3 (q, *J* = 32.7 Hz), 128.8, 123.5 (q, *J* = 272.9 Hz), 123.4 (q, *J* = 3.7 Hz), 116.5, 112.8 (q, *J* = 3.7 Hz), 47.8, 31.3, 13.7.

^{19}F NMR (282 MHz, $CDCl_3$): δ [ppm] = -63.50.

4-(Pyridin-3-yl)pyrimidin-2-amine (37)

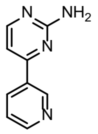

4-(Pyridin-3-yl)pyrimidin-2-amine (**37**) was synthesized according to a literature-known procedure using 3-(dimethylamino)-1-(pyridin-3-yl)prop-2-en-1-one (**36**) (2.10 g, 11.9 mmol).[2]

1.94 g (11.3 mmol, 95%), $C_9H_8N_4$ (172.19 g · mol^{-1}).

ESI-MS: $m \cdot z^{-1}$ = 173.2 [M+H]$^+$ (calc.: 173.1).

^{1}H NMR (500 MHz, DMSO-d_6): δ [ppm] = 9.23 (dd, *J* = 2.3, 0.8 Hz, 1H), 8.68 (dd, *J* = 4.8, 1.7 Hz, 1H), 8.39 (ddd, *J* = 8.0, 2.2, 1.8 Hz, 1H), 8.36 (d, *J* = 5.1 Hz, 1H), 7.53 (ddd, *J* = 8.0, 4.8, 0.8 Hz, 1H), 7.21 (d, *J* = 5.1 Hz, 1H), 6.79 (s, 2H).

^{13}C NMR (126 MHz, DMSO-d_6): δ [ppm] = 163.8, 161.6, 159.4, 151.2, 148.0, 134.2, 132.5, 123.8, 106.0.

Methyl 4-methyl-3-((4-(pyridin-3-yl)pyrimidin-2-yl)amino)benzoate (4)

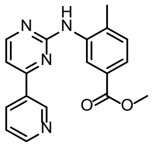

Methyl 4-methyl-3-((4-(pyridin-3-yl)pyrimidin-2-yl)amino)benzoate (**4**) was synthesized using a modified procedure of a literature-known protocol.[3]

An oven-dried 50 mL flask was flushed with argon and filled with 4-(Pyridin-3-yl)pyrimidin-2-amine (**37**) (1.50 g, 8.71 mmol), $Pd_2(dba)_3$ (0.40 g, 0.44 mmol, 0.05 eq), XantPhos (0.50 g, 0.87 mmol, 0.1 eq) and Cs_2CO_3 (3.45 g, 10.6 mmol, 1.2 eq). Then, methyl 3-iodo-4-methylbenzoate (2.65 g, 9.58 mmol, 1.1 eq) in dry 1,4-dioxane (18 mL) was added and the reaction mixture was heated to reflux for 3 h. After cooling to room temperature, water (20 mL) and brine (20 mL) were added and the reaction mixture was extracted with EtOAc (3 x 100 mL). The combined organic extracts were dried over $MgSO_4$ and concentrated in vacuo. Following purification by flash column chromatography (silica, EtOAc + 0.5% NEt_3) yielded the pure product as a yellow solid.

2.52 g (7.87 mmol, 90%), $C_{18}H_{16}N_4O_2$ (320.35 g · mol^{-1}).

ESI-MS: $m \cdot z^{-1}$ = 321.3 [M+H]$^+$ (calc.: 321.1).

^{1}H NMR (500 MHz, DMSO-d_6): δ [ppm] = 9.31 (dd, *J* = 2.2, 0.6 Hz, 1H), 9.08 (s, 1H), 8.71 (dd, *J* = 4.8, 1.6 Hz, 1H), 8.56 (d, *J* = 5.2 Hz, 1H), 8.48 – 8.43 (m, 1H), 8.41 (d, *J* = 1.0 Hz, 1H), 7.65 (dd, *J* = 7.9, 1.8 Hz, 1H), 7.55 (ddd, *J* = 8.0, 4.8, 0.7 Hz, 1H), 7.50 (d, *J* = 5.2 Hz, 1H), 7.39 (d, *J* = 8.0 Hz, 1H), 3.86 (s, 3H), 2.35 (s, 3H).

^{13}C NMR (126 MHz, DMSO-d_6): δ [ppm] = 166.2, 161.5, 160.7, 159.6, 151.5, 148.2, 138.2, 137.1, 134.2, 132.0, 130.7, 127.5, 124.6, 124.5, 123.8, 108.2, 52.1, 18.3.

Nilotinib-S(*t*Bu) (6)

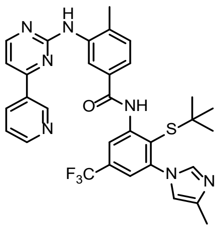

Aniline derivative **5** (0.62 g, 1.9 mmol) and methyl benzoate **4** (0.78 g, 2.4 mmol, 1.3 eq) were dissolved in dry THF (10 mL) under argon atmosphere. At 0 °C, potassium *tert*-butoxide (1.16 g, 10.3 mmol, 5.5 eq) in dry THF (10 mL) was added dropwise and the solution was allowed to warm to room temperature. After 3 h, brine (10 mL) was added and the reaction mixture was extracted with EtOAc (3 x 50 mL). The combined organic extracts were dried over $MgSO_4$ and concentrated in vacuo. Following purification by flash column chromatography (silica, DCM/MeOH, 35:1) yielded the pure product as a colorless solid.

1.14 g (1.85 mmol, 97%), $C_{32}H_{30}F_3N_7OS$ (617.69 g · mol^{-1}).

ESI-MS: $m \cdot z^{-1}$ = 618.5 [M+H]$^+$ (calc.: 618.2), 309.9 [M+2H]$^{2+}$ (calc.: 309.6).

^{1}H NMR (500 MHz, DMSO-d_6): δ [ppm] = 9.68 (s, 1H), 9.27 (dd, *J* = 2.2, 0.6 Hz, 1H), 9.11 (s, 1H), 8.68 (dd, *J* = 4.8, 1.6 Hz, 1H), 8.54 (d, *J* = 5.2 Hz, 1H), 8.46 – 8.40 (m, 1H), 8.22 (d, *J* = 2.0 Hz, 1H), 8.08 (d, *J* = 1.4 Hz, 1H), 7.89 (d, *J* = 1.7 Hz, 1H), 7.57 (d, *J* = 1.2 Hz, 1H), 7.53 (ddd, *J* = 8.0, 4.8, 0.7 Hz, 1H), 7.48 (d, *J* = 5.2 Hz, 1H), 7.43 (dd, *J* = 7.9, 1.8 Hz, 1H), 7.37 (d, *J* = 8.1 Hz, 1H), 6.95 – 6.90 (m, 1H), 2.32 (s, 3H), 2.04 (d, *J* = 0.8 Hz, 3H), 1.12 (s, 9H).

^{13}C NMR (126 MHz, DMSO-d_6): δ [ppm] = 165.9, 161.7, 161.0, 159.7, 151.5, 148.2, 139.8, 138.3, 138.2, 136.7, 136.6, 136.3, 134.4, 132.9, 132.2, 131.8 (q, *J* = 3.2 Hz), 131.5, 130.4, 128.7 (q, *J* = 32.5 Hz), 124.9 (q, *J* = 3.2 Hz), 124.2, 123.9, 123.3 (q, *J* = 273.0 Hz), 123.3, 117.2, 108.0, 47.8, 30.7, 18.3, 13.4.

^{19}F NMR (282 MHz, methanol-d_4): δ [ppm] = -63.92.

Nilotinib-SH (1a)

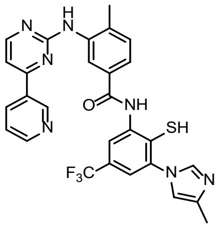

Thioanisole (0.20 mL, 1.7 mmol, 5.3 eq) and TFMSA (0.40 mL, 4.6 mmol, 14.2 eq) were added to a solution of nilotinib-S(*t*Bu) (**6**) (0.20 g, 0.32 mmol) in TFA (0.5 mL). After 30 min at room temperature, the reaction mixture was poured on crushed ice. The resulting aqueous solution was adjusted to pH 7 with 1 M NaOH and extracted with EtOAc (3 x 50 mL). The combined organic extracts were washed with brine (1 x 30 mL), dried over $MgSO_4$, and concentrated in vacuo. After purification by flash column chromatography (silica, DCM/MeOH, 10:1), the pure product was obtained as a yellow solid.

0.10 g (0.18 mmol, 56%), $C_{28}H_{22}F_3N_7OS$ (561.58 g · mol^{-1}).

ESI-MS: $m \cdot z^{-1}$ = 562.4 [M+H]$^+$ (ber.: 562.2), 281.8 [M+2H]$^{2+}$ (ber.: 281.6).

^{1}**H NMR** (500 MHz, DMSO-d_6): δ [ppm] = 9.88 (s, 1H), 9.27 (d, *J* = 2.2 Hz, 1H), 9.09 (s, 1H), 9.01 (s, 1H), 8.70 (dd, *J* = 4.8, 1.4 Hz, 1H), 8.53 (d, *J* = 5.1 Hz, 1H), 8.48 – 8.39 (m, 1H), 8.09 (d, *J* = 1.1 Hz, 1H), 7.65 – 7.51 (m, 2H), 7.51 – 7.41 (m, 2H), 7.33 (d, *J* = 8.0 Hz, 1H), 7.11 (s, 1H), 6.99 (s, 1H), 2.30 (s, 3H), 2.17 (s, 3H).

^{19}**F NMR** (282 MHz, DMSO-d_6): δ [ppm] = -61.57.

Note: The ^{13}C NMR could not be evaluated due to poor resolution and low signal intensities caused by C-F coupling.

Synthesis of ponatinib-SH, ponatinib-BT, GZD824-SH, and GZD824-BT

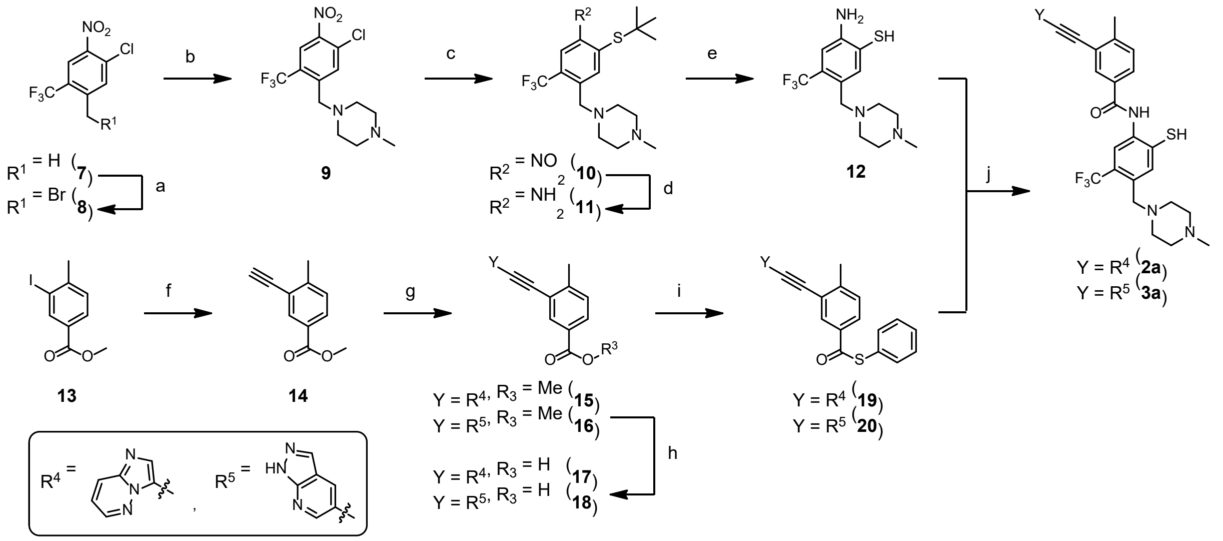

Scheme S2: Synthesis routes towards ponatinib-SH (**2a**) and GZD824-SH (**3a**) via NCL-type reaction. *Reagents and conditions:* (a) NBS, AIBN, AcOH, 80 °C, 26 h, *72%*; (b) 1-methylpiperazine, DIPEA, DMF, rt, 1 h, *91%*; (c) *t*BuSH, Cs_2CO_3, DMF, rt, 1 h, *83%*; (d) *i.* $Na_2S_2O_4$, MeOH, H_2O, rt, 1 h; *ii.* conc. HCl, rt, 3 h, *86%*; (e) TFMSA, TFA, thioanisole, rt, 30 min, *100%*; (f) *i.* ethynyltrimethylsilane, $Pd(PPh_3)_2Cl_2$, CuI, NEt_3, THF, rt, 17 h, Ar; *ii.* K_2CO_3, MeOH, rt, 30 min, *88%*; (g) 3-bromoimidazo[1,2-*b*]pyridazine or 5-bromo-1*H*-pyrazolo[3,4-*b*]pyridine, $Pd(PPh_3)_2Cl_2$, CuI, DIPEA, DMF, 80 °C, 5 h, Ar, *99%* **15**/*75%* **16**; (h) NaOH, MeOH, H_2O, 60 °C, 3 h, *67%* **17**/*90%* **18**; (i) thiophenol, HATU, DIPEA, DMF, 45 °C, 3 h, *100%* **19**/*56%* **20**; (j) transfer buffer (4 M GnHCl, 100 mM NaH_2PO_4/Na_2HPO_4, 15 mM TCEP, pH 7.2), MeCN, 40 °C, 24 h, Ar, *14%* **2a**/*35%* **3a**.

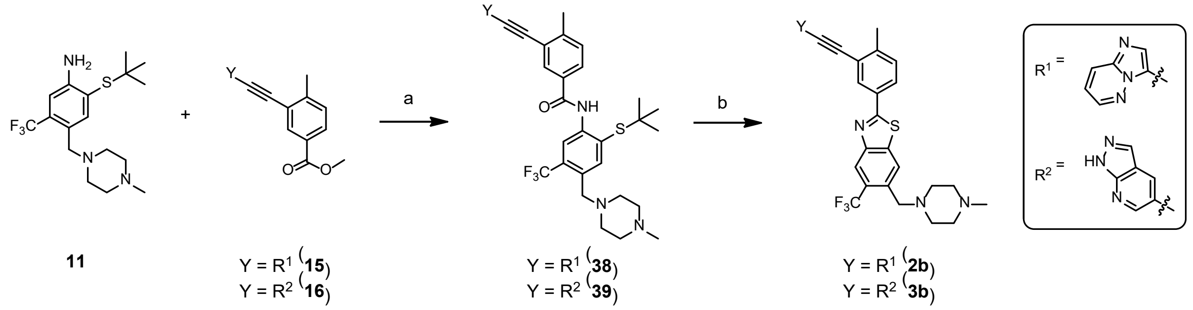

Scheme S3: Synthesis routes towards ponatinib-BT (**2b**) and GZD824-BT (**3b**). *Reagents and conditions:* (a) KO*t*Bu, THF, rt, 1 h, Ar, *83%* **38**/*93%* **39**; (b) TFMSA, TFA, thioanisole, rt, 30 min, *88%* **2b**/*59%* **3b**.

1-(Bromomethyl)-5-chloro-4-nitro-2-(trifluoromethyl)benzene (8)

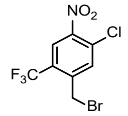

To a solution of 1-chloro-5-methyl-2-nitro-4-(trifluoromethyl)benzene (**7**) (4.90 g, 20.5 mmol) in AcOH (35 mL) were added NBS (6.37 g, 35.8 mmol, 1.8 eq) and AIBN (0.67 g, 4.1 mmol, 0.2 eq). After 26 h at 80 °C, the reaction mixture was diluted with EtOAc (200 mL). The resulting solution was washed with water (3 x 50 mL) and brine (1 x 50 mL), dried over $MgSO_4$, and concentrated in vacuo. Remaining AcOH was removed by co-evaporation with toluene. This process was repeated twice. Following purification

by flash column chromatography (silica, cyclohexane/EtOAc, 50:1 to 20:1) yielded the pure product as a yellow solid.

4.69 g (14.7 mmol, 72%), $C_8H_4BrClF_3NO_2$ (318.48 g · mol^{-1}).

^{1}H NMR (500 MHz, $CDCl_3$): *δ* [ppm] = 8.21 (s, 1H), 7.84 (s, 1H), 4.59 (s, 2H).

^{13}C NMR (126 MHz, $CDCl_3$): *δ* [ppm] = 146.8, 142.0, 136.4, 131.8, 128.0 (q, *J* = 34.2 Hz), 124.3 (q, *J* = 5.8 Hz), 122.5 (q, *J* = 274.6 Hz), 25.6 (q, *J* = 2.7 Hz).

^{19}F NMR (282 MHz, $CDCl_3$): *δ* [ppm] = -60.36.

1-(5-Chloro-4-nitro-2-(trifluoromethyl)benzyl)-4-methylpiperazine (9)

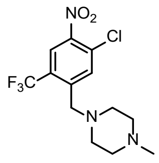

To a solution of benzyl bromide **8** (1.26 g, 3.95 mmol) and DIPEA (1.28 mL, 7.55 mmol, 1.9 eq) in DMF (40 mL) was added 1-methylpiperazine (0.76 mL, 6.9 mmol, 1.7 eq) in DMF (25 mL). The reaction mixture was stirred at room temperature for 1 h and diluted with EtOAc (200 mL) afterwards. The resulting solution was washed with water (3 x 50 mL) and brine (1 x 50 mL), dried over $MgSO_4$, and concentrated in vacuo. After flash column chromatography (silica, DCM/MeOH, 30:1 + 0.5% NEt_3), the pure product was obtained as a brown oil.

1.22 g (3.61 mmol, 91%), $C_{13}H_{15}ClF_3N_3O_2$ (337.73 g · mol^{-1}).

ESI-MS: $m \cdot z^{-1}$ = 338.2 [M(^{35}Cl)+H]$^+$ (calc.: 338.1), 340.2 [M(^{37}Cl)+H]$^+$ (calc.: 340.1).

^{1}H NMR (500 MHz, $CDCl_3$): *δ* [ppm] = 8.18 (s, 1H), 8.10 (s, 1H), 3.70 (s, 2H), 2.72 – 2.38 (m, 8H), 2.34 (s, 3H).

^{13}C NMR (126 MHz, $CDCl_3$): *δ* [ppm] = 145.9, 144.8, 133.8, 131.4, 128.2 (q, *J* = 32.6 Hz), 123.9 (q, *J* = 6.0 Hz), 122.8 (q, *J* = 274.0 Hz), 57.4, 55.1, 53.1, 46.0.

^{19}F NMR (282 MHz, $CDCl_3$): *δ* [ppm] = -60.47.

1-(5-(*tert*-Butylthio)-4-nitro-2-(trifluoromethyl)benzyl)-4-methylpiperazine (10)

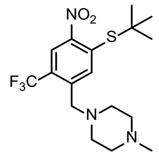

2-Methylpropane-2-thiol (0.52 mL, 4.6 mmol, 1.3 eq) and Cs_2CO_3 (2.32 g, 7.11 mmol, 2.0 eq) were added to a solution of compound **9** (1.20 g, 3.55 mmol) in DMF (17 mL) under thoroughly stirring. After 1 h at room temperature, the reaction mixture was partitioned between EtOAc (150 mL) and water (30 mL). The organic layer was washed with water (3 x 50 mL) and brine (1 x 50 mL), dried over $MgSO_4$,

and concentrated in vacuo. Following purification by flash column chromatography (silica, DCM/MeOH, 30:1 + 0.5% NEt_3) yielded the pure product as a brown solid.

1.16 g (2.96 mmol, 83%), $C_{17}H_{24}F_3N_3O_2S$ (391.45 g · mol^{-1}).

ESI-MS: $m \cdot z^{-1}$ = 392.3 $[M+H]^+$ (calc.: 392.1).

^{1}H NMR (500 MHz, $CDCl_3$): δ [ppm] = 8.31 (s, 1H), 8.08 (s, 1H), 3.69 (s, 2H), 2.76 – 2.35 (m, 8H), 2.30 (s, 3H), 1.46 (s, 9H).

^{13}C NMR (126 MHz, $CDCl_3$): δ [ppm] = 149.8, 142.0, 136.1, 136.1, 127.3 (q, *J* = 32.3 Hz), 123.2 (q, *J* = 274.0 Hz), 122.5 (q, *J* = 6.0 Hz), 57.5 (q, *J* = 1.8 Hz), 55.3, 53.3, 48.8, 46.1, 31.3.

^{19}F NMR (282 MHz, $CDCl_3$): δ [ppm] = -60.42.

1-(4-Amino-5-(*tert*-butylthio)-2-(trifluoromethyl)benzyl)-4-methylpiperazine (11)

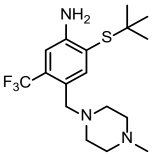

Sodium dithionite (5.60 g, 32.2 mmol, 11.0 eq) was added portionwise to a suspension of nitroarene **10** (1.14 g, 2.92 mmol) in MeOH (12 mL) and water (12 mL). The reaction mixture was stirred at room temperature for 1 h, followed by adjusting to pH 1 with concentrated HCl. After stirring for another 3 h, saturated $NaHCO_3$ solution (40 mL) was added and the reaction mixture was extracted with EtOAc (3 x 100 mL). The combined organic extracts were washed with brine (1 x 30 mL), dried over $MgSO_4$, and concentrated in vacuo. Following purification by flash column chromatography (silica, DCM/MeOH, 10:1 + 0.5% NEt_3) yielded the pure product as a yellow solid.

0.90 g (2.5 mmol, 86%), $C_{17}H_{26}F_3N_3S$ (361.47 g · mol^{-1}).

ESI-MS: $m \cdot z^{-1}$ = 362.3 $[M+H]^+$ (calc.: 362.2).

^{1}H NMR (500 MHz, $CDCl_3$): δ [ppm] = 7.67 (s, 1H), 6.96 (s, 1H), 4.57 (s, 2H), 3.50 (s, 2H), 2.67 – 2.40 (m, 8H), 2.31 (s, 3H), 1.32 (s, 9H).

^{13}C NMR (126 MHz, $CDCl_3$): δ [ppm] = 149.0, 141.7, 130.7 (q, *J* = 30.0 Hz), 125.6, 124.3 (q, *J* = 274.5 Hz), 119.5, 112.0 (q, *J* = 5.8 Hz), 57.7, 55.3, 52.8, 48.6, 46.0, 31.2.

^{19}F NMR (282 MHz, $CDCl_3$): δ [ppm] = -59.84.

2-Amino-5-((4-methylpiperazin-1-yl)methyl)-4-(trifluoromethyl)benzenethiol (12)

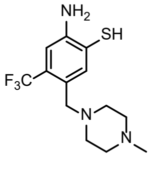

Thioanisole (0.50 mL, 4.3 mmol, 1.6 eq) and TFMSA (3.50 mL, 39.9 mmol, 14.4 eq) were added successively to a solution of aniline derivative **11** (1.00 g, 2.77 mmol) in TFA (7 mL). After 30 min at

room temperature, the reaction mixture was poured on crushed ice. The resulting aqueous solution was adjusted to pH 6 with 1 M NaOH and extracted with EtOAc (5 x 50 mL). The organic extracts were combined and re-extracted with 1 M HCl (5 x 50 mL). Next, the aqueous extracts were pooled, adjusted to pH 6 with 1 M NaOH and once more extracted with EtOAc (5 x 50 mL). The combined organic extracts were dried over $MgSO_4$ and concentrated in vacuo, yielding the pure product as a yellow solid.

0.84 g (2.8 mmol, 100%), $C_{13}H_{18}F_3N_3S$ (305.36 g · mol^{-1}).

ESI-MS: $m \cdot z^{-1}$ = 306.0 $[M+H]^+$ (calc.: 306.1).

^{1}H NMR (500 MHz, DMSO-d_6): δ [ppm] = 7.33 (s, 1H), 7.05 (s, 1H), 5.84 (s, 2H), 3.28 (s, 2H), 2.36 – 2.15 (m, 8H), 2.14 (s, 3H).

^{13}C NMR (126 MHz, DMSO-d_6): δ [ppm] = 147.6, 135.2, 128.7 (q, J = 29.3 Hz), 124.3 (q, J = 274.1 Hz), 122.8, 119.6, 111.8 (q, J = 6.0 Hz), 56.9, 54.4, 52.2, 45.3.

^{19}F NMR (471 MHz, DMSO-d_6): δ [ppm] = -58.17.

Methyl 3-ethynyl-4-methylbenzoate (14)

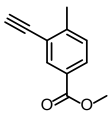

Methyl 3-ethynyl-4-methylbenzoate (**14**) was synthesized using a modified procedure of a literature-known protocol.[4]

An oven-dried 100 mL flask was flushed with argon and filled with methyl 3-iodo-4-methyl-benzoate (**13**) (4.65 g, 16.9 mmol), CuI (0.23 g, 1.2 mmol, 0.07 eq), $Pd_2(PPh_3)_2Cl_2$ (0.59 g, 0.84 mmol, 0.05 eq) and dry THF (50 mL). Then, NEt_3 (7.01 mL, 50.6 mmol, 3.0 eq) and ethynyltrimethylsilane (4.73 mL, 33.7 mmol, 2.0 eq) were added and the reaction mixture was stirred at room temperature for 17 h. After filtration through kieselguhr, the solvent was evaporated under reduced pressure and the residue was dissolved in EtOAc (200 mL). The resulting solution was washed with water (1 x 50 mL) and brine (1 x 20 mL), dried over $MgSO_4$, and concentrated in vacuo.

Without any further purification, the obtained intermediate was dissolved in MeOH (60 mL). K_2CO_3 (3.50 g, 25.3 mmol, 1.5 eq) was added and the reaction mixture was stirred at room temperature for 30 min. After evaporation of the solvent under reduced pressure, the residue was dissolved in EtOAc (200 mL) and washed with water (1 x 50 mL) and brine (1 x 20 mL). The organic layer was dried over $MgSO_4$ and concentrated under vacuo. Following purification by flash column chromatography (silica, cyclohexane/EtOAc, 15:1) yielded the pure product as a brown solid.

2.57 g (14.8 mmol, 88%), $C_{11}H_{10}O_2$ (174.20 g · mol^{-1}).

^{1}H NMR (500 MHz, $CDCl_3$): δ [ppm] = 8.13 (d, J = 1.8 Hz, 1H), 7.90 (dd, J = 8.0, 1.9 Hz, 1H), 7.28 (d, J = 8.0 Hz, 1H), 3.90 (s, 3H), 3.31 (s, 1H), 2.50 (s, 3H).

^{13}C NMR (126 MHz, $CDCl_3$): δ [ppm] = 166.5, 146.1, 133.9, 129.8, 129.8, 128.0, 122.5, 82.0, 81.6, 52.3, 21.0.

Methyl 3-((imidazo[1,2-*b*]pyridazin-3-yl)ethynyl)-4-methylbenzoate (15)

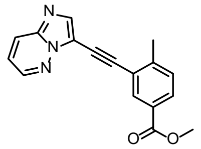

Methyl 3-((imidazo[1,2-*b*]pyridazin-3-yl)ethynyl)-4-methylbenzoate (**15**) was synthesized using a modified procedure of a literature-known protocol.[4]

An oven-dried 100 mL flask was flushed with argon and filled with methyl 3-ethynyl-4-methyl-benzoate (**14**) (1.03 g, 5.89 mmol), 3-bromoimidazo[1,2-*b*]pyridazine (1.16 g, 5.89 mmol, 1.0 eq), CuI (0.11 g, 0.59 mmol, 0.1 eq), $Pd_2(PPh_3)_2Cl_2$ (0.21 g, 0.29 mmol, 0.05 eq) and dry DMF (30 mL). Then, DIPEA (2.00 mL, 11.8 mmol, 2.0 eq) was added and the reaction mixture was heated to 80 °C for 5 h. After filtration through kieselguhr, the reaction mixture was diluted with EtOAc (200 mL). The resulting solution was washed with water (3 x 50 mL) and brine (1 x 20 mL), dried over $MgSO_4$, and concentrated in vacuo. After purification by flash column chromatography (silica, DCM/MeOH, 100:1 + 0.5% NEt_3), the pure product was obtained as a brown solid.

1.70 g (5.84 mmol, 99%), $C_{17}H_{13}N_3O_2$ (291.30 g · mol^{-1}).

ESI-MS: $m \cdot z^{-1}$ = 292.2 $[M+H]^+$ (calc.: 292.1).

^{1}H NMR (400 MHz, DMSO-*d*$_6$): δ [ppm] = 8.72 (dd, *J* = 4.4, 1.5 Hz, 1H), 8.25 (dd, *J* = 9.2, 1.5 Hz, 1H), 8.23 (s, 1H), 8.05 (d, *J* = 1.8 Hz, 1H), 7.89 (dd, *J* = 8.0, 1.8 Hz, 1H), 7.52 (d, *J* = 8.0 Hz, 1H), 7.39 (dd, *J* = 9.2, 4.5 Hz, 1H), 3.87 (s, 3H), 2.59 (s, 3H).

^{13}C NMR (101 MHz, DMSO-*d*$_6$): δ [ppm] = 165.4, 145.1, 144.9, 139.7, 138.4, 131.7, 130.4, 129.4, 127.7, 126.1, 122.2, 119.1, 111.6, 95.9, 81.3, 52.3, 20.5.

3-((Imidazo[1,2-*b*]pyridazin-3-yl)ethynyl)-4-methylbenzoic acid (17)

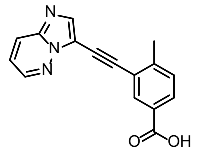

To a suspension of methyl benzoate **15** (1.70 g, 5.83 mmol) in MeOH (130 mL) was added 1 M NaOH (18.00 mL, 18.00 mmol, 3.1 eq). The reaction mixture was heated to 60 °C for 3 h, meanwhile the suspension turned into solution. The solution was reduced to half of the original volume by evaporation under reduced pressure and adjusted to pH 2 with 1 M HCl afterwards, resulting in precipitation of the product. The precipitate was filtered off, washed with ice-cold water, and dried under vacuum, yielding the pure product as a yellow solid.

1.08 g (3.89 mmol, 67%), $C_{16}H_{11}N_3O_2$ (277.28 g · mol^{-1}).

ESI-MS: $m \cdot z^{-1}$ = 278.2 $[M+H]^+$ (calc.: 278.1).

^{1}H NMR (400 MHz, DMSO-*d*$_6$): δ [ppm] = 13.12 (br. s, 1H), 8.72 (dd, *J* = 4.4, 1.4 Hz, 1H), 8.30 – 8.19 (m, 2H), 8.05 (d, *J* = 1.8 Hz, 1H), 7.88 (dd, *J* = 7.9, 1.8 Hz, 1H), 7.50 (d, *J* = 8.0 Hz, 1H), 7.39 (dd, *J* = 9.2, 4.5 Hz, 1H), 2.58 (s, 3H).

^{13}**C NMR** (101 MHz, DMSO-d_6): δ [ppm] = 166.5, 145.1, 144.4, 139.7, 138.2, 131.9, 130.2, 129.7, 128.9, 126.1, 122.0, 119.1, 111.7, 96.2, 81.1, 20.5.

Phenyl-derived thioester 19

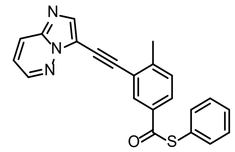

HATU (1.37 g, 3.60 mmol, 2.0 eq) was added to a solution of benzoic acid derivative **17** (0.50 g, 1.8 mmol) and DIPEA (1.20 mL, 7.20 mmol, 4.0 eq) in DMF (15 mL). After 15 min at 45 °C, thiophenol (1.80 mL, 18.0 mmol, 10.0 eq) was added and the reaction mixture was stirred for another 3 h. The solvent was evaporated under reduced pressure and the obtained residue was purified by flash column chromatography (silica, DCM/MeOH, 80:1), yielding the pure product as a yellow solid.

0.66 g (1.8 mmol, 100%), $C_{22}H_{15}N_3OS$ (369.44 g · mol^{-1}).

ESI-MS: $m \cdot z^{-1}$ = 370.3 [M+H]$^+$ (calc.: 370.1).

^{1}**H NMR** (500 MHz, $CDCl_3$): δ [ppm] = 8.49 (dd, J = 4.4, 1.0 Hz, 1H), 8.25 (d, J = 2.0 Hz, 1H), 8.09 (s, 1H), 8.02 (dd, J = 9.1, 1.0 Hz, 1H), 7.91 (dd, J = 8.0, 1.9 Hz, 1H), 7.55 – 7.50 (m, 2H), 7.50 – 7.43 (m, 3H), 7.39 (d, J = 8.1 Hz, 1H), 7.14 (dd, J = 9.1, 4.4 Hz, 1H), 2.66 (s, 3H).

^{13}**C NMR** (126 MHz, $CDCl_3$): δ [ppm] = 189.3, 146.5, 144.0, 138.6, 135.2, 134.6, 131.0, 130.2, 129.7, 129.4, 129.2, 127.7, 127.6, 127.3, 126.1, 123.3, 117.9, 96.6, 81.1, 21.3.

Ponatinib-SH (2a)

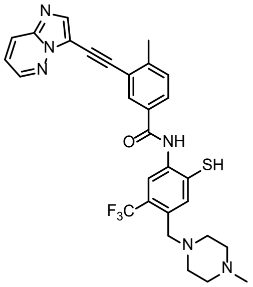

A solution of phenyl-derived thioester **19** (54 mg, 0.15 mmol, 1.0 eq) in MeCN (15 mL) was added to a solution of mercaptoaniline **12** (45 mg, 0.15 mmol) in transfer buffer (45 mL) under argon atmosphere. The reaction mixture was stirred at 40 °C for 24 h and extracted with EtOAc (5 x 50 mL) afterwards. The combined organic extracts were dried over $MgSO_4$ and concentrated in vacuo. Following purification of the residue by preparative HPLC (water/MeCN, 3% to 70% MeCN in 30 min) yielded the pure product as a pale yellow solid.

Transfer buffer (4 M GnHCl, 100 mM NaH_2PO_4/Na_2HPO_4, 12.5 mM TCEP in water, pH 7.2)

12 mg (0.021 mmol, 14%), $C_{29}H_{27}F_3N_6OS$ (564.62 g · mol^{-1}).

ESI-MS: $m \cdot z^{-1}$ = 565.3 [M+H]$^+$ (calc.: 565.2), 283.2 [M+2H]$^{2+}$ (calc.: 283.1).

^{1}**H NMR** (500 MHz, DMSO-*d*$_6$): *δ* [ppm] = 11.11 (br. s, 1H), 8.72 (dd, *J* = 4.4, 1.4 Hz, 1H), 8.50 – 7.98 (m, 4H), 7.95 (dd, *J* = 8.0, 1.7 Hz, 1H), 7.74 (s, 1H), 7.57 (d, *J* = 8.2 Hz, 1H), 7.39 (dd, *J* = 9.2, 4.4 Hz, 1H), 3.50 (s, 2H), 3.00 – 2.55 (m, 14H).

^{19}**F NMR** (471 MHz, DMSO-*d*$_6$): *δ* [ppm] = -54.92.

Note: The ^{13}C NMR could not be evaluated due to poor resolution and low signal intensities caused by C-F coupling.

Ponatinib-S(*t*Bu) (38)

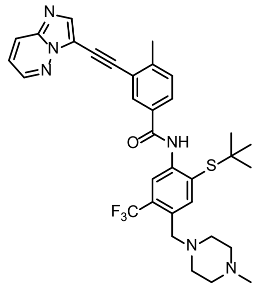

Aniline derivative **11** (0.28 g, 0.77 mmol) and methyl benzoate **15** (0.29 g, 1.0 mmol, 1.3 eq) were dissolved in dry THF (5 mL) under argon atmosphere. At 0 °C, potassium *tert*-butoxide (0.48 g, 4.2 mmol, 5.5 eq) in dry THF (5 mL) was added dropwise and the solution was allowed to warm to room temperature. After 1 h, brine (10 mL) was added and the reaction mixture was extracted with EtOAc (3 x 50 mL). The combined organic extracts were dried over $MgSO_4$ and concentrated in vacuo. Following purification by flash column chromatography (silica, DCM/MeOH, 50:1 to 30:1 + 0.5% NEt_3) yielded the pure product as a brown solid.

0.40 g (0.64 mmol, 83%), $C_{33}H_{35}F_3N_6OS$ (620.73 g · mol^{-1}).

ESI-MS: $m \cdot z^{-1}$ = 621.4 $[M+H]^+$ (calc.: 621.2), 311.4 $[M+2H]^{2+}$ (calc.: 311.1).

^{1}**H NMR** (500 MHz, methanol-*d*$_4$): *δ* [ppm] = 8.73 (s, 1H), 8.60 (dd, *J* = 4.4, 1.5 Hz, 1H), 8.10 – 8.05 (m, 2H), 8.03 (s, 1H), 8.01 (s, 1H), 7.84 (dd, *J* = 8.0, 2.0 Hz, 1H), 7.47 (d, *J* = 8.1 Hz, 1H), 7.34 (dd, *J* = 9.2, 4.4 Hz, 1H), 3.66 (s, 2H), 2.73 – 2.38 (m, 11H), 2.33 (s, 3H), 1.32 (s, 9H).

^{13}**C NMR** (126 MHz, methanol-*d*$_4$): *δ* [ppm] = 165.7, 146.1, 146.0, 141.7, 141.4, 141.2, 138.2, 133.9, 133.0, 131.5, 131.4, 131.2 (q, *J* = 30.6 Hz), 128.4, 128.1, 126.4, 125.4 (q, *J* = 273.6 Hz), 124.4, 120.5, 119.0 (q, *J* = 6.1 Hz), 114.3, 97.5, 81.8, 58.4, 56.0, 53.5, 50.0, 45.8, 31.4, 21.0.

^{19}**F NMR** (282 MHz, methanol-*d*$_4$): *δ* [ppm] = -60.18.

Ponatinib-BT (2b)

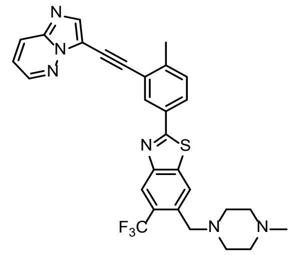

Thioanisole (75 µL, 0.64 mmol, 1.3 eq) and TFMSA (0.60 mL, 6.8 mmol, 13.6 eq) were added to a solution of ponatinib-S(*t*Bu) (**38**) (0.31 g, 0.50 mmol) in TFA (1.2 mL). After 1 h at room temperature, the reaction mixture was poured on crushed ice. The resulting aqueous solution was adjusted to pH 9 with 4 M NaOH and extracted with EtOAc (3 x 50 mL). The combined organic extracts were washed with brine (1 x 30 mL), dried over $MgSO_4$, and concentrated in vacuo. After purification by flash column chromatography (silica, DCM/MeOH, 20:1 to 2:1 + 0.5% NEt_3) the pure product was obtained as a yellow solid.

0.24 g (0.44 mmol, 88%), $C_{29}H_{25}F_3N_6S$ (546.61 g · mol^{-1}).

ESI-MS: $m \cdot z^{-1}$ = 547.4 $[M+H]^+$ (calc.: 547.2), 274.2 $[M+2H]^{2+}$ (calc.: 274.1).

^{1}H NMR (500 MHz, DMSO-d_6): δ [ppm] = 8.74 (dd, *J* = 4.4, 1.5 Hz, 1H), 8.52 (s, 1H), 8.39 (s, 1H), 8.29 – 8.25 (m, 2H), 8.24 (d, *J* = 2.0 Hz, 1H), 8.06 (dd, *J* = 7.9, 1.9 Hz, 1H), 7.60 (d, *J* = 8.1 Hz, 1H), 7.41 (dd, *J* = 9.2, 4.4 Hz, 1H), 3.83 (s, 2H), 3.46 – 3.40 (m, 2H), 3.14 – 3.06 (m, 2H), 3.03 – 2.96 (m, 2H), 2.82 (s, 3H), 2.61 (s, 3H), 2.48 – 2.42 (m, 2H).

^{13}C NMR (126 MHz, DMSO-d_6): δ [ppm] = 168.7, 152.1, 145.1, 143.5, 139.7, 138.7, 138.4, 133.0, 131.0, 130.4, 129.4, 127.8, 126.1 (q, *J* = 30.1 Hz), 126.1, 124.5, 124.3 (q, *J* = 275.2 Hz), 122.9, 120.4 (q, *J* = 5.1 Hz), 119.2, 111.6, 96.0, 81.6, 56.7, 52.8, 49.4, 42.1, 20.4.

^{19}F NMR (471 MHz, DMSO-d_6): δ [ppm] = -57.38.

Extinction coefficient: $\varepsilon_{260,exp.}$ [L · mol^{-1} · cm^{-1}] = 26200.

Note: The NMR spectra were recorded with 10 µL of TFA to increase the solubility of ponatinib-BT (**2b**) in DMSO-d_6. The signals of TFA are not listed.

Methyl 3-((1*H*-pyrazolo[3,4-*b*]pyridin-5-yl)ethynyl)-4-methylbenzoate (16)

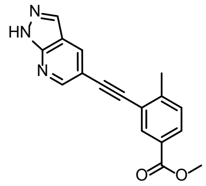

Methyl 3-((1*H*-pyrazolo[3,4-*b*]pyridin-5-yl)ethynyl)-4-methylbenzoate (**16**) was synthesized using a modified procedure of a literature-known protocol.[4]

An oven-dried 100 mL flask was flushed with argon and filled with methyl 3-ethynyl-4-methyl-benzoate (**14**) (11.12 g, 6.44 mmol), 5-bromo-1*H*-pyrazolo[3,4-*b*]pyridine (1.27 g, 6.44 mmol, 1.0 eq), CuI (0.12 g, 0.64 mmol, 0.1 eq), $Pd_2(PPh_3)_2Cl_2$ (0.23 g, 0.32 mmol, 0.05 eq) and dry DMF (35 mL). Then,

DIPEA (2.19 mL, 12.88 mmol, 2.0 eq) was added and the reaction mixture was heated to 80 °C for 5 h. After filtration through kieselguhr, the reaction mixture was diluted with EtOAc (200 mL). The resulting solution was washed with water (3 x 50 mL) and brine (1 x 20 mL), dried over $MgSO_4$, and concentrated in vacuo. After purification by flash column chromatography (silica, DCM/MeOH, 100:1 + 0.5% NEt_3), the pure product was obtained as a brown solid.

1.40 g (4.81 mmol, 75%), $C_{17}H_{13}N_3O_2$ (291.30 g · mol^{-1}).

ESI-MS: $m \cdot z^{-1}$ = 292.4 $[M+H]^+$ (calc.: 292.1).

^{1}H NMR (500 MHz, DMSO-d_6): δ [ppm] = 13.93 (s, 1H), 8.73 (d, J = 2.0 Hz, 1H), 8.52 (d, J = 1.7 Hz, 1H), 8.21 (d, J = 1.3 Hz, 1H), 8.06 (d, J = 1.8 Hz, 1H), 7.87 (dd, J = 8.0, 1.9 Hz, 1H), 7.49 (d, J = 8.0 Hz, 1H), 3.86 (s, 3H), 2.56 (s, 3H).

^{13}C NMR (126 MHz, DMSO-d_6): δ [ppm] = 165.5, 151.1, 150.5, 145.2, 133.8, 133.1, 132.0, 130.3, 129.2, 127.6, 122.6, 114.0, 111.7, 92.1, 87.8, 52.2, 20.6.

3-((1*H*-pyrazolo[3,4-*b*]pyridin-5-yl)ethynyl)-4-methylbenzoic acid (18)

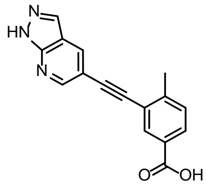

To a suspension of methyl benzoate **16** (1.40 g, 4.82 mmol) in MeOH (55 mL) was added 1 M NaOH (15.00 mL, 15.00 mmol, 3.1 eq). The reaction mixture was heated to 60 °C for 3 h, meanwhile the suspension turned into solution. The solution was reduced to half of the original volume by evaporation under reduced pressure and adjusted to pH 2 with 1 M HCl afterwards, resulting in precipitation of the product. The precipitate was filtered off, washed with ice-cold water, and dried under vacuum, yielding the pure product as a cream solid.

1.21 g (4.36 mmol, 90%), $C_{16}H_{11}N_3O_2$ (277.28 g · mol^{-1}).

ESI-MS: $m \cdot z^{-1}$ = 278.1 $[M+H]^+$ (calc.: 278.1).

^{1}H NMR (400 MHz, DMSO-d_6): δ [ppm] = 13.93 (br. s, 1H), 13.09 (br. s, 1H), 8.73 (d, J = 1.9 Hz, 1H), 8.52 (d, J = 2.0 Hz, 1H), 8.21 (s, 1H), 8.06 (d, J = 1.7 Hz, 1H), 7.86 (dd, J = 7.9, 1.8 Hz, 1H), 7.47 (d, J = 8.1 Hz, 1H), 2.56 (s, 3H).

^{13}C NMR (101 MHz, DMSO-d_6): δ [ppm] = 166.6, 151.2, 150.6, 144.7, 133.7, 133.1, 132.3, 130.1, 129.4, 128.8, 122.4, 114.0, 111.8, 91.9, 88.0, 20.6.

Phenyl-derived thioester 20

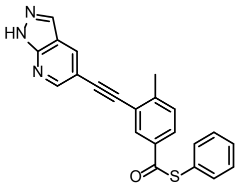

HATU (1.92 g, 5.1 mmol, 2.0 eq) was added to a solution of benzoic acid derivative **18** (0.70 g, 2.5 mmol) and DIPEA (1.70 mL, 10.1 mmol, 4.0 eq) in DMF (21 mL). After 15 min at 45 °C, thiophenol (2.60 mL, 25.2 mmol, 10.0 eq) was added and the reaction mixture was stirred for another 3 h. The solvent was evaporated under reduced pressure and the obtained residue was purified by flash column chromatography (silica, DCM/MeOH, 100:1), yielding the pure product as a yellow solid.

0.53 g (1.4 mmol, 56%), $C_{22}H_{15}N_3OS$ (369.44 g · mol^{-1}).

ESI-MS: $m \cdot z^{-1}$ = 370.3 $[M+H]^+$ (calc.: 370.1).

^{1}H NMR (500 MHz, $CDCl_3$): δ [ppm] = 13.94 (s, 1H), 8.75 (d, J = 1.9 Hz, 1H), 8.53 (d, J = 1.9 Hz, 1H), 8.21 (d, J = 1.1 Hz, 1H), 8.06 (d, J = 2.0 Hz, 1H), 7.90 (dd, J = 8.0, 2.0 Hz, 1H), 7.60 – 7.44 (m, 6H), 2.58 (s, 3H).

^{13}C NMR (126 MHz, $CDCl_3$): δ [ppm] = 188.1, 151.2, 150.5, 146.3, 135.0, 133.9, 133.2, 130.7, 129.8, 129.7, 129.5, 127.1, 126.5, 123.1, 114.0, 111.6, 92.7, 87.6, 20.7.

GZD824-SH (3a)

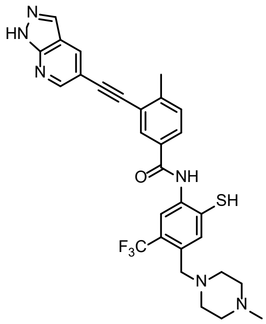

A solution of phenyl-derived thioester **20** (54 mg, 0.15 mmol, 1.0 eq) in MeCN (15 mL) was added to a solution of mercaptoaniline **12** (45 mg, 0.15 mmol) in transfer buffer (45 mL) under argon atmosphere. The reaction mixture was stirred at 40 °C for 24 h and extracted with EtOAc (5 x 50 mL) afterwards. The combined organic extracts were dried over $MgSO_4$ and concentrated in vacuo. Following purification of the residue by preparative HPLC (water/MeCN, 3% to 80% MeCN in 30 min) yielded the pure product as a pale yellow solid.

Transfer buffer (4 M GnHCl, 100 mM NaH_2PO_4/Na_2HPO_4, 12.5 mM TCEP in water, pH 7.2)

30 mg (0.053 mmol, 35%), $C_{29}H_{27}F_3N_6OS$ (564.62 g · mol^{-1}).

ESI-MS: $m \cdot z^{-1}$ = 565.3 $[M+H]^+$ (calc.: 565.2), 283.2 $[M+2H]^{2+}$ (calc.: 283.1).

^{1}H NMR (500 MHz, DMSO-d_6): δ [ppm] = 13.93 (s, 1H), 11.35 (s, 1H), 8.75 (d, J = 2.0 Hz, 1H), 8.55 (d, J = 2.0 Hz, 1H), 8.46 (s, 1H), 8.22 (s, 1H), 8.11 (d, J = 1.7 Hz, 1H), 7.92 (dd, J = 8.0, 1.8 Hz, 1H), 7.64 – 7.50 (m, 2H), 3.49 (s, 2H), 3.15 – 2.95 (m, 4H), 2.79 – 2.51 (m, 10H).

^{19}**F NMR** (471 MHz, DMSO-d_6): δ [ppm] = -54.96.

Note: The ^{13}C NMR could not be evaluated due to poor resolution and low signal intensities caused by C-F coupling.

GZD824-S(*t*Bu) (39)

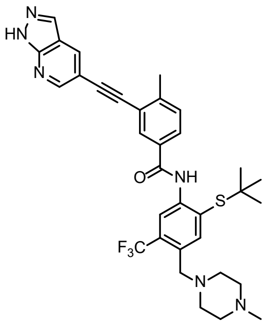

Aniline derivative **11** (0.26 g, 0.73 mmol) and methyl benzoate **16** (0.28 g, 0.9 mmol, 1.3 eq) were dissolved in dry THF (10 mL) under argon atmosphere. At 0 °C, potassium *tert*-butoxide (0.45 g, 4.0 mmol, 5.5 eq) in dry THF (5 mL) was added dropwise and the solution was allowed to warm to room temperature. After 1 h, brine (10 mL) was added and the reaction mixture was extracted with EtOAc (4 x 50 mL). The combined organic extracts were dried over $MgSO_4$ and concentrated in vacuo. Following purification by flash column chromatography (silica, DCM/MeOH, 50:1 to 20:1 + 0.5% NEt_3) yielded the pure product as a colorless solid.

0.42 g (0.68 mmol, 93%), $C_{33}H_{35}F_3N_6OS$ (620.73 g · mol^{-1}).

ESI-MS: $m \cdot z^{-1}$ = 621.4 $[M+H]^+$ (calc.: 621.3), 311.2 $[M+2H]^{2+}$ (calc.: 311.1).

^{1}**H NMR** (400 MHz, DMSO-d_6): δ [ppm] = 13.93 (s, 1H), 10.08 (s, 1H), 8.73 (d, *J* = 1.9 Hz, 1H), 8.52 (d, *J* = 1.9 Hz, 1H), 8.40 (s, 1H), 8.22 (s, 1H), 8.15 (d, *J* = 1.7 Hz, 1H), 7.98 (s, 1H), 7.91 (dd, *J* = 8.0, 1.8 Hz, 1H), 7.57 (d, *J* = 8.2 Hz, 1H), 3.75 (s, 2H), 3.46 – 3.36 (m, 2H), 3.07 – 2.99 (m, 2H), 2.98 – 2.88 (m, 2H), 2.79 (s, 3H), 2.60 (s, 3H), 2.55 – 2.50 (m, 2H), 1.25 (s, 9H).

^{13}**C NMR** (126 MHz, DMSO-d_6): δ [ppm] = 164.0, 151.1, 150.6, 144.1, 140.5, 140.4, 133.7, 133.1, 132.0, 131.6, 130.5, 130.3, 129.4, 128.5 (q, *J* = 30.5 Hz), 127.5, 123.9 (q, *J* = 274.2 Hz), 122.6, 120.5 (q, *J* = 6.9 Hz), 114.0, 111.7, 92.1, 88.1, 56.1, 52.5, 49.3, 48.5, 42.0, 30.6, 20.5.

^{19}**F NMR** (471 MHz, DMSO-d_6): δ [ppm] = -57.90.

Note: The NMR spectra were recorded with 10 µL of TFA to increase the solubility of GZD824-S(*t*Bu) (**39**) in DMSO-d_6. The signals of TFA are not listed.

GZD824-BT (3b)

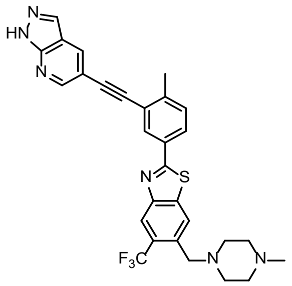

Thioanisole (0.15 mL, 1.3 mmol, 4.8 eq) and TFMSA (0.60 mL, 6.8 mmol, 25.2 eq) were added to a solution of GZD824-S(*t*Bu) (**39**) (0.17 g, 0.27 mmol) in TFA (1.6 mL). After 1 h at room temperature, the reaction mixture was poured on crushed ice. The resulting aqueous solution was adjusted to pH 9 with 4 M NaOH and extracted with EtOAc (3 x 50 mL). The combined organic extracts were washed with brine (1 x 30 mL), dried over $MgSO_4$, and concentrated in vacuo. After purification by flash column chromatography (silica, DCM/MeOH, 20:1 to 2:1 + 0.5% NEt_3), the pure product was obtained as a pale yellow solid.

90 mg (0.16 mmol, 59%), $C_{29}H_{25}F_3N_6S$ (546.61 g · mol^{-1}).

ESI-MS: $m \cdot z^{-1}$ = 547.4 $[M+H]^+$ (calc.: 547.2), 274.2 $[M+2H]^{2+}$ (calc.: 274.1).

^{1}H NMR (500 MHz, DMSO-*d*$_6$): *δ* [ppm] = 13.98 (s, 1H), 8.73 (d, *J* = 2.0 Hz, 1H), 8.51 (d, *J* = 1.9 Hz, 1H), 8.45 (s, 1H), 8.28 (s, 1H), 8.21 (s, 1H), 8.17 (d, *J* = 2.0 Hz, 1H), 7.97 (dd, *J* = 8.0, 1.9 Hz, 1H), 7.51 (d, *J* = 8.2 Hz, 1H), 3.69 (s, 2H), 2.55 (s, 3H), 2.54 – 2.46 (m, 8H), 2.27 (s, 3H).

^{13}C NMR (126 MHz, DMSO-*d*$_6$): *δ* [ppm] = 168.6, 151.9, 151.1, 150.6, 143.6, 138.6, 133.8, 133.6, 133.1, 130.8, 130.3, 129.7, 127.4, 126.0 (q, *J* = 30.2 Hz), 124.3 (q, *J* = 273.8 Hz), 124.2, 123.2, 120.1 (q, *J* = 6.3 Hz), 114.0, 111.7, 92.3, 87.8, 57.5, 54.2, 52.0, 44.9, 20.4.

^{19}F NMR (471 MHz, DMSO-*d*$_6$): *δ* [ppm] = -57.44.

Synthesis of ponatinib-SH fragments

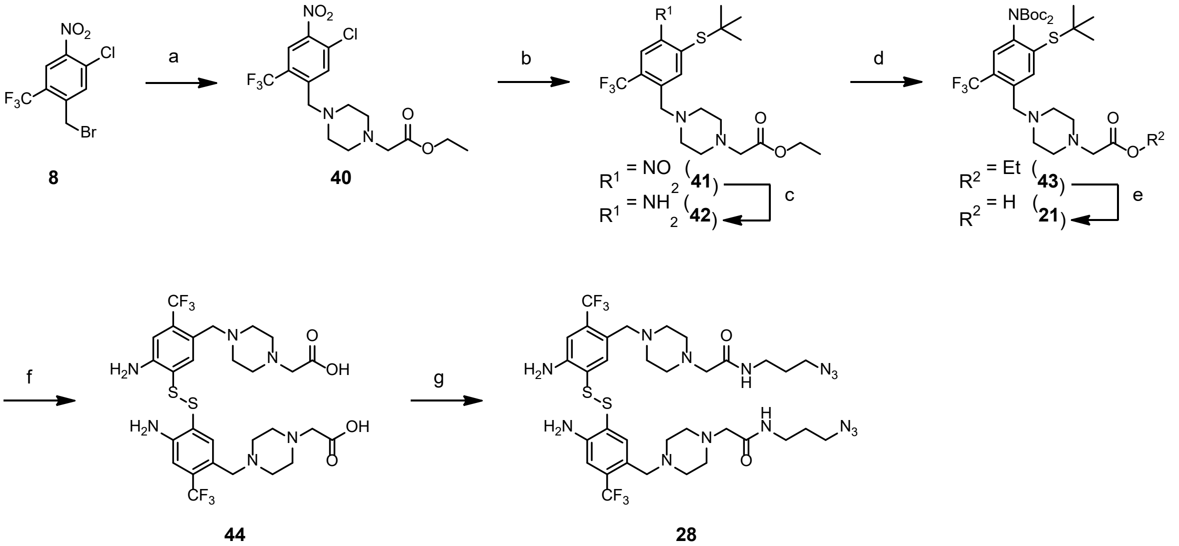

Scheme S4: Synthesis route towards ponatinib-SH acceptor fragments. *Reagents and conditions:* (a) ethyl 1-piperazineacetate, DIPEA, DCM, rt, 2 h, *72%*; (b) *t*BuSH, Cs_2CO_3, DMF, rt, 1 h, *81%*; (c) *i.* $Na_2S_2O_4$, MeOH, H_2O, rt, 1 h; *ii.* conc. HCl, rt, 4 h, *70%*; (d) Boc_2O, DMAP, toluene, 100 °C, 2 h, *90%*; (e) LiOH, THF, H_2O, rt, 1 h, *57%.*; (f) TFMSA, TFA, thioanisole, rt, 30 min, *88%*; (g) 3-azido-1-propanamine, HATU, DIPEA, DMF, rt, 30 min, *79%*.

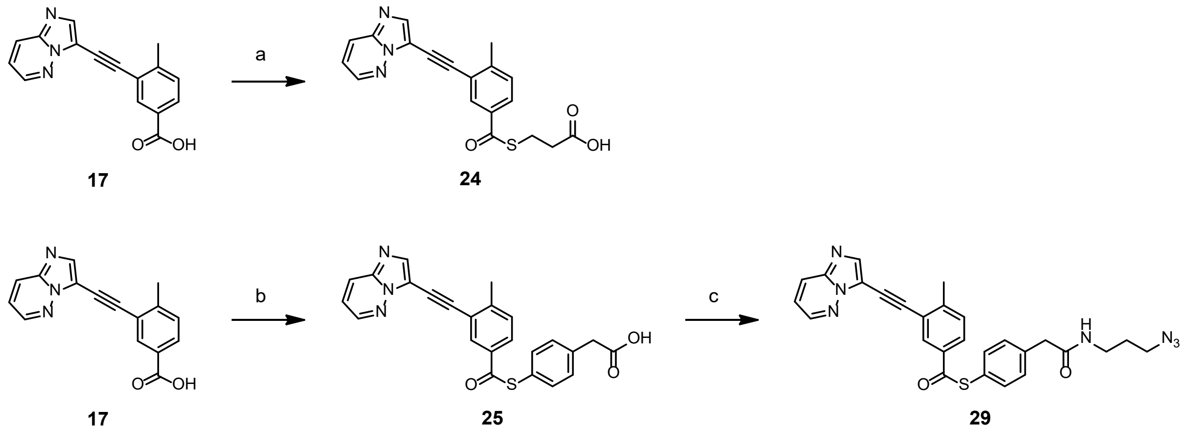

Scheme S5: Synthesis routes towards ponatinib-SH donor fragments. *Reagents and conditions:* (a) MPA, HATU, DIPEA, DMF, 45 °C, 3 h, *62%*; (b) MPAA, HATU, DIPEA, DMF, 45 °C, 3 h, *78%*; (c) 3-azido-1-propanamine, HATU, DIPEA, DMF, rt, 30 min, *83%*.

Ethyl 2-(4-(5-chloro-4-nitro-2-(trifluoromethyl)benzyl)piperazin-1-yl)acetate (40)

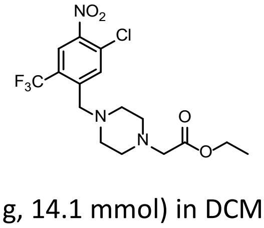

To a solution of benzyl bromide **8** (4.50 g, 14.1 mmol) in DCM (35 mL) were added ethyl 2-(piperazin-1-yl)acetate (2.78 mL, 16.9 mmol, 1.2 eq) and DIPEA (2.88 mL, 16.9 mmol, 1.2 eq). The reaction mixture was stirred at room temperature for 2 h and diluted with DCM (200 mL) afterwards. The resulting

solution was washed with water (1 x 30 mL) and brine (1 x 30 mL), dried over $MgSO_4$, and concentrated in vacuo. After purification by flash column chromatography (silica, DCM/MeOH, 50:1 to 20:1), the pure product was obtained as a brown oil.

4.16 g (10.15 mmol, 72%), $C_{16}H_{19}ClF_3N_3O_4$ (409.79 g · mol^{-1}).

ESI-MS: $m \cdot z^{-1}$ = 410.2 [M(^{35}Cl)+H]$^+$ (calc.: 410.1), 412.25 [M(^{37}Cl)+H]$^+$ (calc.: 412.1).

^{1}H NMR (400 MHz, $CDCl_3$): δ [ppm] = 8.17 (s, 1H), 8.11 (s, 1H), 4.20 (q, *J* = 7.1 Hz, 2H), 3.70 (s, 2H), 3.27 (s, 2H), 2.81 – 2.44 (m, 8H), 1.28 (t, *J* = 7.1 Hz, 3H).

^{13}C NMR (101 MHz, $CDCl_3$): δ [ppm] = 170.1, 145.9, 144.6, 133.9, 131.4, 128.2 (q, *J* = 32.9 Hz), 123.9 (q, *J* = 6.1 Hz), 122.8 (q, *J* = 274.2 Hz), 60.9, 59.2, 57.3, 53.0, 52.8, 14.4.

^{19}F NMR (282 MHz, $CDCl_3$): δ [ppm] = -60.39.

Ethyl 2-(4-(5-(*tert*-butylthio)-4-nitro-2-(trifluoromethyl)benzyl)piperazin-1-yl)acetate (41)

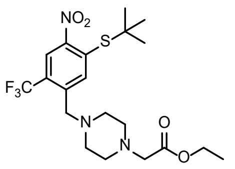

2-Methylpropane-2-thiol (1.38 mL, 12.2 mmol, 1.3 eq) and Cs_2CO_3 (6.13 g, 18.8 mmol, 2.0 eq) were added to a solution of compound **40** (3.85 g, 9.41 mmol) in DMF (50 mL) under thoroughly stirring. After 1 h at room temperature, water (50 mL) was added and the reaction mixture was extracted with EtOAc (4 x 100 mL). The combined organic extracts were dried over $MgSO_4$ and concentrated in vacuo. Following purification by flash column chromatography (silica, DCM/MeOH, 50:1 to 10:1) yielded the pure product as a brown oil.

3.54 g (7.64 mmol, 81%), $C_{20}H_{28}F_3N_3O_4S$ (463.51 g · mol^{-1}).

ESI-MS: $m \cdot z^{-1}$ = 464.4 [M+H]$^+$ (calc.: 464.2).

^{1}H NMR (400 MHz, $CDCl_3$): δ [ppm] = 8.31 (s, 1H), 8.08 (s, 1H), 4.18 (q, *J* = 7.1 Hz, 2H), 3.70 (s, 2H), 3.23 (s, 2H), 2.74 – 2.53 (m, 8H), 1.45 (s, 9H), 1.27 (t, *J* = 7.1 Hz, 3H).

^{13}C NMR (101 MHz, $CDCl_3$): δ [ppm] = 170.3, 149.8, 141.9, 136.1, 136.1, 127.3 (q, *J* = 32.4 Hz), 123.1 (q, *J* = 274.1 Hz), 122.5 (q, *J* = 5.8 Hz), 60.8, 59.4, 57.5, 53.2, 53.1, 48.8, 31.3, 14.4.

^{19}F NMR (282 MHz, $CDCl_3$): δ [ppm] = -60.40.

Ethyl 2-(4-(4-amino-5-(*tert*-butylthio)-2-(trifluoromethyl)benzyl)piperazin-1-yl)acetate (42)

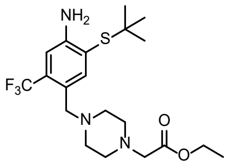

Sodium dithionite (13.00 g, 74.65 mmol, 10.0 eq) was added portionwise to a suspension of nitroarene **41** (3.46 g, 7.47 mmol) in MeOH (50 mL) and water (25 mL). The reaction mixture was stirred at room temperature for 1 h, followed by adjusting to pH 1 with concentrated HCl. After stirring for another 3 h, saturated $NaHCO_3$ solution (20 mL) was added and the reaction mixture was extracted with EtOAc (3 x 100 mL). The combined organic extracts were washed with brine (1 x 30 mL), dried over $MgSO_4$, and concentrated in vacuo. Following purification by flash column chromatography (silica, DCM/MeOH, 70:1 to 40:1 + 0.5% NEt_3) yielded the pure product as a yellow solid.

2.26 g (5.21 mmol, 70%), $C_{20}H_{30}F_3N_3O_2S$ (433.53 g · mol^{-1}).

ESI-MS: $m \cdot z^{-1}$ = 434.4 $[M+H]^+$ (calc.: 434.2).

^{1}H NMR (400 MHz, $CDCl_3$): δ [ppm] = 7.69 (s, 1H), 6.96 (s, 1H), 4.58 (s, 2H), 4.18 (q, *J* = 7.1 Hz, 2H), 3.51 (s, 2H), 3.20 (s, 2H), 2.79 – 2.35 (m, 8H), 1.31 (s, 9H), 1.26 (t, *J* = 7.1 Hz, 3H).

^{13}C NMR (101 MHz, $CDCl_3$): δ [ppm] = 170.4, 149.0, 141.8, 130.7 (q, *J* = 28.1 Hz), 125.6, 124.2 (q, *J* = 274.1 Hz), 119.5, 111.9 (q, *J* = 5.9 Hz), 60.7, 59.6, 57.6, 53.2, 52.8, 48.6, 31.2, 14.4.

^{19}F NMR (282 MHz, $CDCl_3$): δ [ppm] = -59.74.

Ethyl 2-(4-(4-(di(*tert*-butyloxycarbonyl)amino)-5-(*tert*-butylthio)-2-(trifluoromethyl)benzyl)-piperazin-1-yl)acetate (43)

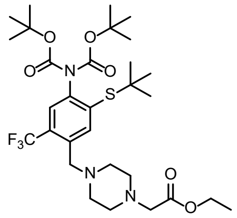

Di-*tert*-butyl dicarbonate (2.68 mL, 12.5 mmol, 6.0 eq) and catalytic amounts of DMAP were added to a solution of aniline derivate **42** (0.90 g, 2.1 mmol) in toluene (10 mL). The reaction mixture was heated to 100 °C for 2 h. After removal of the solvent under reduced pressure, the residue was purified by flash column chromatography (silica, DCM/MeOH, 50:1 to 10:1), yielding the pure product as a yellow oil.

1.19 g (1.88 mmol, 90%), $C_{30}H_{46}F_3N_3O_6S$ (633.76 g · mol^{-1}).

ESI-MS: $m \cdot z^{-1}$ = 634.4 $[M+H]^+$ (calc.: 634.3).

^{1}H NMR (500 MHz, $CDCl_3$): δ [ppm] = 8.05 (s, 1H), 7.41 (s, 1H), 4.18 (q, *J* = 7.1 Hz, 2H), 3.65 (s, 2H), 3.21 (s, 2H), 2.77 – 2.45 (m, 8H), 1.41 (s, 18H), 1.35 (s, 9H), 1.27 (t, *J* = 7.1 Hz, 3H).

^{13}C NMR (126 MHz, $CDCl_3$): δ [ppm] = 170.3, 151.1, 141.0, 138.4, 137.3, 136.6, 128.54 (q, *J* = 31.3 Hz), 126.9 (q, *J* = 5.5 Hz), 123.9 (q, *J* = 273.9 Hz), 83.1, 60.8, 59.5, 57.6, 53.2, 52.9, 48.0, 31.7, 28.0, 14.4.

^{19}F NMR (471 MHz, $CDCl_3$): δ [ppm] = -59.84.

2-(4-(4-(Di(*tert*-butyloxycarbonyl)amino)-5-(*tert*-butylthio)-2-(trifluoromethyl)benzyl)piperazin-1-yl)acetic acid (21)

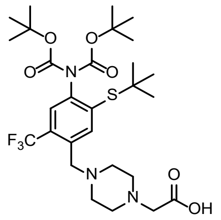

A solution of LiOH (0.22, 9.2 mmol, 4.1 eq) in water (13 mL) was added to a solution of compound **43** (1.42 g, 2.24 mmol) in THF (13 mL). After 1 h at room temperature, the reaction mixture was adjusted to pH 5 with $KHSO_4$ solution and extracted with EtOAc (3 x 100 mL). The combined organic extracts were washed with brine (1 x 30 mL), dried over $MgSO_4$, and concentrated in vacuo. After purification by flash column chromatography (silica C18, water/MeOH, 1:1 to 1:6), the pure product was obtained as a pale yellow solid.

0.77 g (1.27 mmol, 57%), $C_{28}H_{42}F_3N_3O_6S$ (605.71 g · mol^{-1}).

ESI-MS: $m \cdot z^{-1}$ = 606.5 $[M+H]^+$ (calc.: 606.3).

^{1}H NMR (500 MHz, DMSO-d_6): δ [ppm] = 8.02 (s, 1H), 7.63 (s, 1H), 3.65 (s, 2H), 3.14 (s, 2H), 2.68 – 2.53 (m, 4H), 2.49 – 2.37 (m, 4H), 1.35 (s, 18H), 1.32 (s, 9H).

^{13}C NMR (126 MHz, DMSO-d_6): δ [ppm] = 170.7, 150.3, 140.7, 137.7, 137.1, 136.4, 127.52 – 126.53 (m), 123.8 (q, J = 274.2 Hz), 82.3, 58.5, 56.7, 52.2, 52.0, 47.8, 31.2, 27.4.

^{19}F NMR (471 MHz, DMSO-d_6): δ [ppm] = -57.94.

Disulfide 44

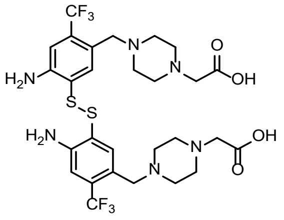

Thioanisole (0.23 mL, 1.9 mmol, 7.6 eq) and TFMSA (0.38 mL, 4.3 mmol, 17.2 eq) were added to a solution of compound **21** (0.15 g, 0.25 mmol) in TFA (1.5 mL). After 30 min at room temperature, the reaction mixture was poured on crushed ice. The resulting aqueous solution was adjusted to pH 10 with 1 M NaOH, washed with diethyl ether (2 x 5 mL), and kept under air over night. After adjusting to pH 7 with 1 M HCl, the residue was purified by flash column chromatography (silica C18, water/MeCN, 1:0 to 3:2), yielding the pure product as a yellow oil.

77 mg (0.11 mmol, 88%), $C_{28}H_{34}F_6N_6O_4S_2$ (696.73 g · mol^{-1}).

ESI-MS: $m \cdot z^{-1}$ = 697.3 $[M+H]^+$ (calc.: 697.2), 349.3 $[M+2H]^{2+}$ (calc.: 349.1).

^{1}H NMR (500 MHz, DMSO-d_6): δ [ppm] = 7.39 (s, 2H), 7.03 (s, 2H), 5.79 (s, 4H), 3.29 (s, 4H), 2.80 (s, 4H), 2.47 – 2.15 (m, 16H).

^{13}C NMR (126 MHz, DMSO-d_6): δ [ppm] = 172.9, 147.8, 135.5, 128.8 (q, J = 30.5 Hz), 124.3 (q, J = 274.4 Hz), 123.1, 119.7, 112.0, 62.2, 57.3, 52.6, 52.2.

^{19}F NMR (471 MHz, DMSO-d_6): δ [ppm] = -58.12.

Extinction coefficient: $\varepsilon_{260,exp.}$ [L · mol^{-1} · cm^{-1}] = 5100 (reduced form).

Diazide 28

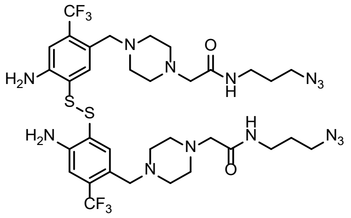

HATU (0.13 g, 0.34 mmol, 2.4 eq), DIPEA (96 µL, 0.56 mmol, 4.0 eq), and 3-azido-1-propanamine (66 µL, 0.68 mmol, 4.9 eq) were added to a solution of disulfide **44** (98 mg, 0.14 mmol) in DMF (4 mL). After 30 min at room temperature, the reaction mixture was diluted with EtOAc (150 mL). The resulting solution was washed with 1 M NaOH (3 x 20 mL) and brine (1 x 20 mL), dried over $MgSO_4$, and concentrated in vacuo. Following purification by flash column chromatography (silica, DCM/MeOH, 50:1 + 0.5% NEt_3) yielded the pure product as a yellow solid.

91 mg (0.11 mmol, 79%), $C_{34}H_{46}F_6N_{14}O_2S_2$ (860.94 g · mol^{-1}).

ESI-MS: $m \cdot z^{-1}$ = 861.4 [M+H]$^+$ (calc.: 861.3), 431.5 [M+2H]$^{2+}$ (calc.: 431.2).

^{1}H NMR (500 MHz, $CDCl_3$): δ [ppm] = 7.42 (s, 2H), 7.35 (t, J = 6.0 Hz, 2H), 6.94 (s, 2H), 4.51 (s, 4H), 3.41 – 3.33 (m, 12H), 2.98 (s, 4H), 2.63 – 2.29 (m, 16H), 1.80 (quin, J = 6.7 Hz, 4H).

^{13}C NMR (126 MHz, $CDCl_3$): δ [ppm] = 170.5, 147.0, 138.1, 131.3 (q, J = 30.3 Hz), 128.9, 124.0 (q, J = 274.7 Hz), 121.2, 112.6 (q, J = 5.8 Hz), 61.5, 57.4, 53.6, 53.1, 49.6, 36.7, 29.0.

^{19}F NMR (471 MHz, $CDCl_3$): δ [ppm] = -59.86.

MPA-derived thioester 24

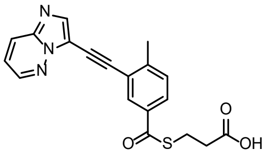

HATU (0.38 g, 1.0 mmol, 0.95 eq) was added to a solution of benzoic acid derivative **17** (0.30 g, 1.1 mmol) and DIPEA (0.36 mL, 2.2 mmol, 2.0 eq) in DMF (13 mL). After 15 min at 45 °C, 3-mercapto-propionic acid (MPA) (0.19 mL, 2.2 mmol, 2.0 eq) was added and the reaction mixture was stirred for another 3 h. The solvent was removed under reduced pressure and ice-cold ethanol (5 mL) was added to the residue. The formed precipitate was filtered off, washed with ice-cold ethanol (2 x 2 mL), and dried under vacuum, yielding the pure product as a yellow solid.

0.25 g (0.68 mmol, 62%), $C_{19}H_{15}N_3O_3S$ (365.41 g · mol^{-1}).

ESI-MS: $m \cdot z^{-1}$ = 366.4 [M+H]$^+$ (calc.: 366.1).

^{1}H NMR (400 MHz, DMSO-d_6): δ [ppm] = 12.57 (br. s, 1H), 8.73 (dd, J = 4.4, 1.6 Hz, 1H), 8.30 – 8.14 (m, 2H), 7.98 (d, J = 1.9 Hz, 1H), 7.86 (dd, J = 7.9, 1.9 Hz, 1H), 7.55 (d, J = 8.1 Hz, 1H), 7.39 (dd, J = 9.2, 4.5 Hz, 1H), 3.22 (t, J = 6.9 Hz, 2H), 2.64 (t, J = 6.9 Hz, 2H), 2.59 (s, 3H).

^{13}C NMR (101 MHz, DMSO-d_6): δ [ppm] = 189.9, 172.8, 145.7, 145.1, 139.7, 138.5, 134.3, 130.7, 129.1, 127.1, 126.1, 122.5, 119.2, 111.6, 95.8, 81.7, 33.8, 24.0, 20.6.

Extinction coefficient: $\varepsilon_{260,exp.}$ [L · mol^{-1} · cm^{-1}] = 22400.

MPAA-derived thioester 25

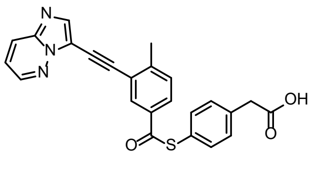

HATU (0.47 g, 1.2 mmol, 1.0 eq) was added to a solution of benzoic acid derivative **17** (0.34 g, 1.2 mmol) and DIPEA (0.63 mL, 3.6 mmol, 3.0 eq) in DMF (15 mL). After 15 min at 45 °C, 4-mercapto-phenylacetic acid (MPAA) (0.42 g, 2.4 mmol, 2.0 eq) was added and the reaction mixture was stirred for another 3 h. The solvent was removed under reduced pressure and ice-cold ethanol (5 mL) was added to the residue. The formed precipitate was filtered off, washed with ice-cold ethanol (2 x 2 mL), and dried under vacuum, yielding the pure product as a yellow solid.

0.40 g (0.94 mmol, 78%), $C_{24}H_{17}N_3O_3S$ (427.48 g · mol^{-1}).

ESI-MS: $m \cdot z^{-1}$ = 428.3 [M+H]$^+$ (calc.: 428.1).

^{1}H NMR (400 MHz, DMSO-d_6): δ [ppm] = 12.46 (br. s, 1H), 8.73 (dd, J = 4.4, 1.5 Hz, 1H), 8.30 – 8.23 (m, 2H), 8.05 (d, J = 1.9 Hz, 1H), 7.93 (dd, J = 8.0, 2.0 Hz, 1H), 7.59 (d, J = 8.2 Hz, 1H), 7.48 (d, J = 8.2 Hz, 2H), 7.44 – 7.36 (m, 3H), 3.67 (s, 2H), 2.62 (s, 3H).

^{13}C NMR (101 MHz, DMSO-d_6): δ [ppm] = 188.2, 172.4, 146.1, 145.1, 139.8, 138.5, 137.1, 134.9, 133.9, 130.8, 130.6, 129.3, 127.4, 126.1, 124.4, 122.7, 119.2, 111.6, 95.8, 81.9, 40.3, 20.7.

Extinction coefficient: $\varepsilon_{260,exp.}$ [L · mol^{-1} · cm^{-1}] = 22000.

Azide-modified MPAA-derived thioester 29

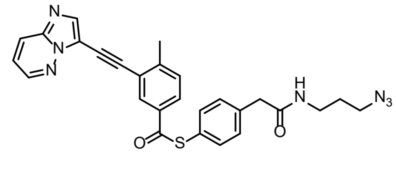

HATU (0.10 g, 0.25 mmol, 1.1 eq), DIPEA (80 µL, 0.47 mmol, 2.0 eq), and 3-azido-1-propanamine (30 µL, 0.30 mmol, 1.3 eq) were added to a solution of MPAA-derived thioester **25** (0.10 g, 0.23 mmol) in DMF (5 mL). After 30 min at room temperature, the reaction mixture was diluted with EtOAc (100 mL). The resulting solution was washed with water (4 x 20 mL) and saturated NH_4Cl solution (1 x 20 mL), dried over $MgSO_4$, and concentrated in vacuo. Following purification by flash column chromatography (silica, DCM/MeOH, 1:0 to 10:1) yielded the pure product as a yellow solid.

98 mg (0.19 mmol, 83%), $C_{27}H_{23}N_7O_2S$ (509.58 g · mol^{-1}).

ESI-MS: $m \cdot z^{-1}$ = 510.4 $[M+H]^+$ (calc.: 510.2).

^{1}H NMR (500 MHz, $CDCl_3$): δ [ppm] = 8.48 (dd, J = 4.4, 1.4 Hz, 1H), 8.23 (d, J = 1.9 Hz, 1H), 8.07 (s, 1H), 8.00 (dd, J = 9.2, 1.5 Hz, 1H), 7.89 (dd, J = 8.0, 1.9 Hz, 1H), 7.54 – 7.45 (m, 2H), 7.41 – 7.33 (m, 3H), 7.14 (dd, J = 9.2, 4.4 Hz, 1H), 5.77 (s, 1H), 3.61 (s, 2H), 3.36 – 3.22 (m, 4H), 2.65 (s, 3H), 1.75 (quin, J = 6.6 Hz, 2H).

^{13}C NMR (126 MHz, $CDCl_3$): δ [ppm] = 189.2, 170.6, 146.6, 144.0, 139.9, 138.6, 136.5, 135.8, 134.4, 131.0, 130.4, 130.2, 127.6, 126.5, 126.1, 123.3, 117.9, 113.1, 96.5, 81.2, 49.6, 43.7, 37.7, 28.7, 21.3.

Synthesis of a thiol-free ponatinib acceptor fragment

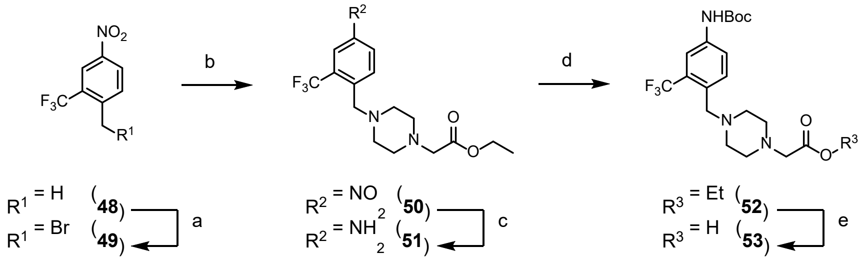

Scheme S6: Synthesis route towards a thiol-free ponatinib acceptor fragment. *Reagents and conditions*: (a) NBS, AIBN, AcOH, 80 °C, 18 h, *56%*; (b) Ethyl 2-(piperazine-1-yl)acetate, DIPEA, DCM, 23 °C, 20 h, *96%*; (c) H_2, Pd/C, EtOH, AcOH, 23 °C, 80 min, *81%*; (d) Boc_2O, DMAP, toluene, 100 °C, 12 h, *crude*; (e) LiOH, H_2O, THF, 23 °C, 1 h, *17% (over 2 steps)*.

1-(Bromomethyl)-4-nitro-2-(trifluoromethyl)benzene (49)

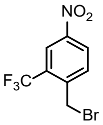

The toluene derivative **48** (1.51 g, 7.35 mmol) was dissolved in AcOH (12.5 mL) at 23 °C and NBS (2.35 g, 13.2 mmol, 1.8 eq) as well as AIBN (0.24 g, 1.47 mmol, 0.2 eq) were added subsequently. The resulting suspension was heated to 80 °C for 18 h while it became a brown solution. Afterwards, the mixture was cooled to rt and diluted with EtOAc (100 mL). The organic layer was washed with water (3 x 100 mL) and brine (100 mL) and afterwards filtered over cotton wool. The solvent was removed under reduced pressure. Following purification by flash column chromatography (silica, pentane/Et_2O, 99:1) yielded the pure product as a pale yellow oil, which solidifies upon freezing.

1.17 g (4.12 mmol, 56%), $C_8H_5BrF_3NO_2$ (284.03 g · mol^{-1})

^{1}H-NMR (500 MHz, $CDCl_3$): δ [ppm] = 8.53 (d, J = 2.4 Hz, 1H), 8.41 (dd, J = 8.5, 2.4 Hz, 1H), 7.84 (d, J = 8.5 Hz, 1H), 4.66 (s, 2H).

^{13}C-NMR (126 MHz, $CDCl_3$): δ [ppm] = 147.4, 143.2, 134.3, 129.9 (q, J = 32.7 Hz), 127.2, 122.9 (q, J = 273.4 Hz), 122.0 (q, J = 5.9 Hz), 26.4 (q, J = 2.8 Hz).

^{19}F-NMR (471 MHz, $CDCl_3$): δ [ppm] = -60.24.

Ethyl 2-(4-(4-nitro-2-(trifluoromethyl)benzyl)piperazin-1-yl)acetate (50)

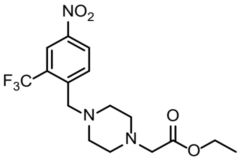

The benzyl bromide **49** (1.17 g, 4.12 mmol) was dissolved in DCM (10 mL). Ethyl 2-(piperazine-1-yl)acetate (812 µL, 4.94 mmol, 1.2 eq) and DIPEA (861 µL, 4.94 mmol, 1.2 eq) were added at rt. The reaction was stirred for 20 h. Then, it was diluted with DCM (75 mL) and washed with water (2 x 50 ml) and brine (50 mL). The organic phase was filtered over cotton wool and the solvent was removed in vacuo. The combined aqueous phases were extracted with DCM (2 x 20 mL) again. These organic

extracts were combined, filtered over cotton wool, added to the former organic phase and the solvent was removed under reduced pressure. After purification by flash column chromatography (silica, DCM/MeOH, 100:1) the pure product was obtained as yellow oil.

1.49 g (3.97 mmol, 96%), $C_{16}H_{20}F_3N_3O_4$ (375.35 g · mol^{-1}).

ESI-MS: $m \cdot z^{-1}$ = 376.5 [M+H]$^+$ (calc.: 376.4).

^{1}H-NMR (500 MHz, $CDCl_3$): *δ* [ppm] = 8.49 (d, *J* = 2.4 Hz, 1H), 8.36 (dd, *J* = 8.6, 2.4 Hz, 1H), 8.16 – 8.05 (m, 1H), 4.18 (q, *J* = 7.2 Hz, 2H), 3.76 (s, 2H), 3.26 (s, 2H), 2.76 – 2.52 (m, 8H), 1.27 (t, *J* = 7.2 Hz, 3H).

^{13}C-NMR (126 MHz, $CDCl_3$): *δ* [ppm] = 167.0, 146.7, 145.7, 131.8, 130.0 (q, *J* = 31.9 Hz), 126.6, 124.3 (q, *J* = 274.5 Hz), 121.6 (q, *J* = 6.0 Hz), 60.9, 59.1, 57.7, 52.9, 52.8, 14.3.

^{19}F-NMR (282 MHz, $CDCl_3$): *δ* [ppm] = -59.91.

Ethyl 2-(4-(4-amino-2-(trifluoromethyl)benzyl)piperazin-1-yl)acetate (51)

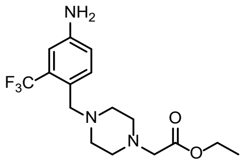

Nitroarene **50** (1.35 g, 3.60 mmol) was dissolved in EtOH/AcOH (10:1, 120 mL). Pd/C (10%, 55% wet, 135 mg) was added and the air was replaced by hydrogen by evaporation and backflushing for five times. After 80 min, the mixture was filtered over celite and rinsed with EtOH. The solvent was removed under reduced pressure. Excess AcOH was removed by co-evaporation with toluene. The residue was purified by flash column chromatography (silica, DCM/MeOH + 1% Et_3N, 100:1) yielding the pure product as yellow oil.

1.10 g (2.93 mmol, 81%), $C_{16}H_{22}F_3N_3O_2$ (345.37 g · mol^{-1})

ESI-MS: $m \cdot z^{-1}$ = 346.4 [M+H]$^+$ (calc.: 346.4).

^{1}H-NMR (500 MHz, $CDCl_3$): *δ* [ppm] = 7.44 (d, *J* = 8.3 Hz, 1H), 6.89 (d, *J* = 2.5 Hz, 1H), 6.76 (dd, *J* = 8.3, 2.5 Hz, 1H), 4.16 (q, *J* = 7.1 Hz, 2H), 3.51 (s, 2H), 3.18 (s, 2H), 2.58 (s, 4H), 2.50 (s, 4H), 1.24 (t, *J* = 7.1 Hz, 3H).

^{13}C-NMR (126 MHz, $CDCl_3$): *δ* [ppm] = 170.4, 145.3, 132.0, 129.6 (q, *J* = 29.9 Hz), 126.6, 124.4 (q, *J* = 273.5 Hz), 117.9, 112.1 (q, *J* = 6.0 Hz), 60.6, 59.6, 57.9, 53.2, 52.8, 14.3.

^{19}F-NMR (471 MHz, $CDCl_3$): *δ* [ppm] = -59.26.

Ethyl 2-(4-(4-((*tert*-butoxycarbonyl)amino)-2-(trifluoromethyl)benzyl)piperazin-1-yl)acetate (52)

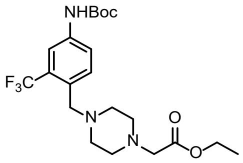

The aniline derivative **51** (1.03 g, 2.97 mmol) was suspended in toluene (14 mL). Boc_2O (3.89 g, 17.8 mmol, 6.0 eq) and DMAP (0.01 g, 0.12 mmol, 0.1 eq) were added subsequently and the whole mixture was heated to 100 °C while it became a solution. After 12 h, the reaction was cooled to rt and the solvent was removed under reduced pressure. The residue was redissolved in toluene and filtered through a plug of silica (elution: toluene/THF + 1% DMEA, 7:1) yielding a brown oil. The oil was used without further purification and without further analytics.

0.51 g, $C_{21}H_{30}F_3N_3O_4$ (445.48 g · mol^{-1})

2-(4-(4-((*tert*-butoxycarbonyl)amino)-2-(trifluoromethyl)benzyl)piperazin-1-yl)acetic acid (53)

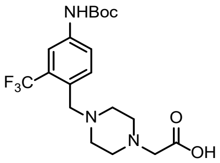

The ester derivative **52** (108 mg, 0.24 mmol) was dissolved in THF (950 µL) and a solution of LiOH (24 mg, 0.99 mmol, 4.1 eq) in water (950 µL) was added. After 90 min, the reaction was purified by semi-preparative RP-HPLC (ACN in water: 15% to 50% in 30 min).

46 mg (0.11 mmol, 17% over two steps), $C_{19}H_{26}F_3N_3O_4$ (417.43 g · mol^{-1}).

ESI-MS: $m \cdot z^{-1}$ = 418.4 $[M+H]^+$ (calc.: 418.2).

^{1}H-NMR (400 MHz, CD_3CN/CD_3OD): δ [ppm] = 7.79 (d, J = 2.0 Hz, 1H), 7.61 – 7.53 (m, 2H), 3.65 (d, J = 1.4 Hz, 2H), 3.51 (s, 2H), 3.30 – 3.19 (m, 4H), 2.74 – 2.67 (m, 4H), 1.47 (s, 9H).

^{13}C-NMR (101 MHz, CD_3CN/CD_3OD): δ [ppm] = 168.5, 154.1, 139.9, 132.5, 130.6, 129.5 (q, J = 30.3 Hz), 125.4 (q, J = 273.3 Hz), 122.3, 116.6 – 116.2 (m), 81.0, 59.3, 57.7, 53.5, 50.3, 28.3.

^{19}F-NMR (471 MHz, CD_3CN/CD_3OD): δ [ppm] = -59.89.

Synthesis of PNA conjugates

PNA acceptor conjugate 23a

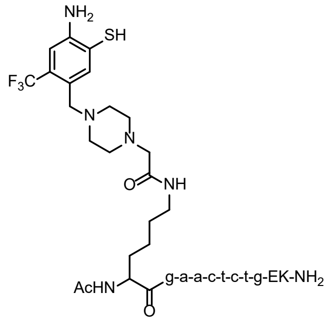

PNA acceptor conjugate **23a** was synthesized by Fmoc-SPS according to the general procedure (scale: 5 µmol). Ac-Lys(Fmoc)-OH was used to introduce the *N*-terminal amino acid unit. After Fmoc removal from the lysine side chain with piperidine in DMF (1:4, v/v; 1 x 3 min, 1 x 7 min), compound **21** (0.2 M in DMF, 4 eq) was coupled in presence of HATU (3.6 eq) and NMM (8 eq) for 30 min at room temperature. This procedure was repeated once. Global deprotection, cleavage from the resin, and HPLC purification were performed as previously described.

Formula: $C_{119}H_{158}F_3N_{55}O_{31}S$ (2943.94 g · mol^{-1}). UPLC: t_r [min] = 2.21 (3 to 30% B1 in 4 min, column 1). ESI-MS: $m \cdot z^{-1}$ = 982.2 $[M+3H]^{3+}$ (calc.: 981.8), 736.9 $[M+4H]^{4+}$ (calc.: 736.6). Extinction coefficient: ε_{260} [L · mol^{-1} · cm^{-1}] = 86700. Yield: 0.76 µmol, 15%.

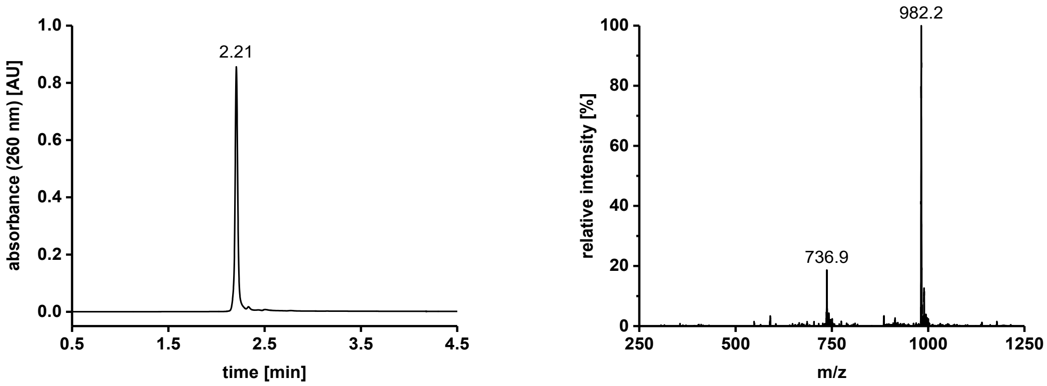

Figure S1: UPLC chromatogram (left) and ESI-MS spectrum (right) of purified PNA acceptor conjugate **23a**.

MPA-derived PNA donor conjugate 27a

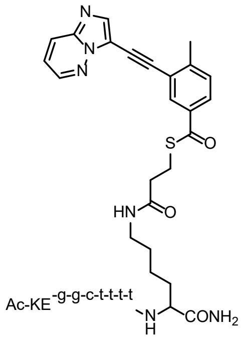

MPA-derived PNA donor conjugate **27a** was synthesized by Fmoc-SPS according to the general procedure (scale: 2 µmol). Fmoc-Lys(Mmt)-OH was used to introduce the *C*-terminal amino acid unit. After synthesis of the PNA oligomer, the Mmt protecting group of the lysine side chain was removed with TFA in DCM (1:99, v/v; 4 x 2 min, 1 x 30 min). Subsequently, MPA-derived thioester **24** (0.1 M in NMP, 4 eq) was coupled in presence of HATU (3.6 eq) and NMM (8 eq) for 30 min at room temperature. This procedure was repeated once. Global deprotection, cleavage from the resin, and HPLC purification were performed as previously described.

Formula: $C_{114}H_{144}N_{44}O_{33}S$ (2690.70 g · mol^{-1}). UPLC: t_r [min] = 3.68 (3 to 30% B1 in 4 min, column 1). ESI-MS: $m \cdot z^{-1}$ = 897.8 $[M+3H]^{3+}$ (calc.: 897.4), 673.6 $[M+4H]^{4+}$ (calc.: 673.3). Extinction coefficient: ε_{260} [L · mol^{-1} · cm^{-1}] = 87600. Yield: 0.15 µmol, 8%.

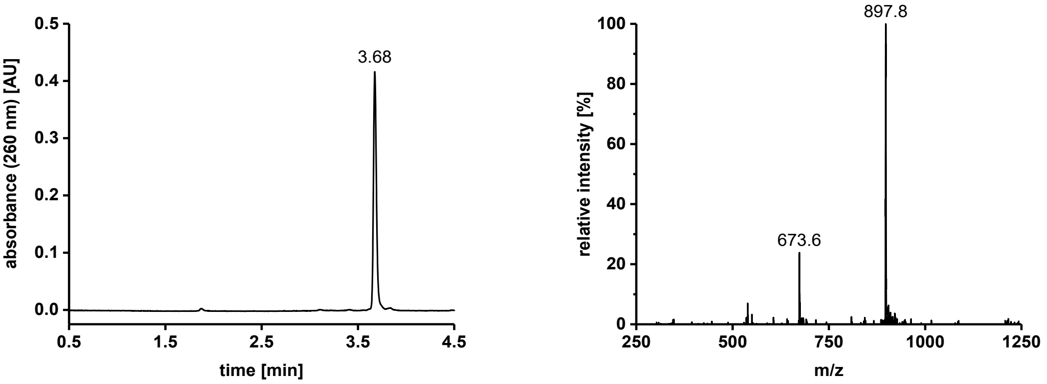

Figure S2: UPLC chromatogram (left) and ESI-MS spectrum (right) of purified PNA donor conjugate **27a**.

MPAA-derived PNA donor conjugate 27b

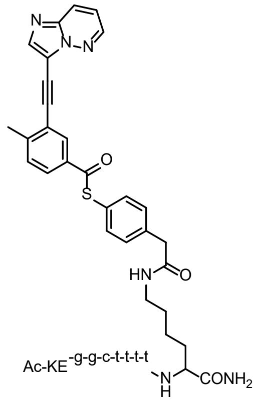

MPAA-derived PNA donor conjugate **27b** was synthesized by Fmoc-SPS according to the general procedure (scale: 2 µmol). Fmoc-Lys(Mmt)-OH was used to introduce the *C*-terminal amino acid unit. After synthesis of the PNA oligomer, the Mmt protecting group of the lysine side chain was removed with TFA in DCM (1:99, v/v; 4 x 2 min, 1 x 30 min). Subsequently, MPAA-derived thioester **25** (0.1 M in NMP, 4 eq) was coupled in presence of HATU (3.6 eq) and NMM (8 eq) for 30 min at room temperature. This procedure was repeated once. Global deprotection, cleavage from the resin, and HPLC purification were performed as previously described.

Formula: $C_{119}H_{146}N_{44}O_{33}S$ (2752.77 g · mol^{-1}). UPLC: t_r [min] = 3.62 (3 to 40% B1 in 4 min, column 1). ESI-MS: $m \cdot z^{-1}$ = 918.5 $[M+3H]^{3+}$ (calc.: 918.0), 689.1 $[M+4H]^{4+}$ (calc.: 688.8). Extinction coefficient: ε_{260} [L · mol^{-1} · cm^{-1}] = 87100. Yield: 0.20 µmol, 10%.

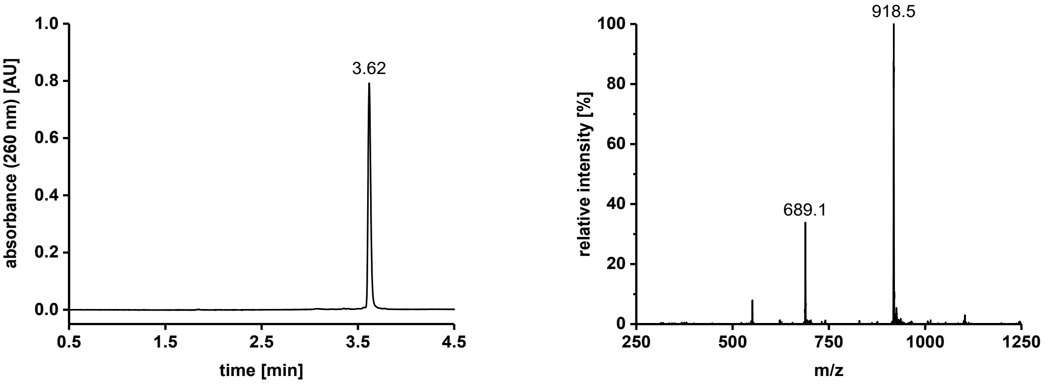

Figure S3: UPLC chromatogram (left) and ESI-MS spectrum (right) of purified PNA donor conjugate **27b**.

PNA inhibitor conjugate 32a

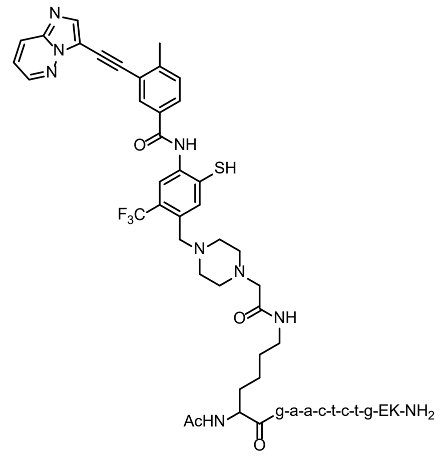

To a solution of PNA acceptor conjugate **23a** (150 nmol) in TCEP buffer 1 (100 mM NaH_2PO_4/Na_2HPO_4, 10 mM TCEP, pH 7.2, 150 µL) and MeCN (150 µL) was added MPAA-derived thioester **25** (15000 nmol, 100 eq, 100 mM in DMSO, 150 µL). The reaction mixture was stirred at room temperature for 24 h, followed by the addition of TCEP buffer 2 (100 mM NaH_2PO_4/Na_2HPO_4, 1 M TCEP, pH 7.2, 150 µL). After another 30 min at room temperature, the product was purified by HPLC as previously described.

Formula: $C_{135}H_{167}F_3N_{58}O_{32}S$ (3203.20 g · mol^{-1}). UPLC: t_r [min] = 3.21 (10 to 40% B2 in 4 min, column 2). ESI-MS: $m \cdot z^{-1}$ = 1068.3 $[M+3H]^{3+}$ (calc.: 1068.1), 802.1 $[M+4H]^{4+}$ (calc.: 801.3). Extinction coefficient: ε_{260} [L · mol^{-1} · cm^{-1}] = 107800. Yield: 28 nmol, 19%.

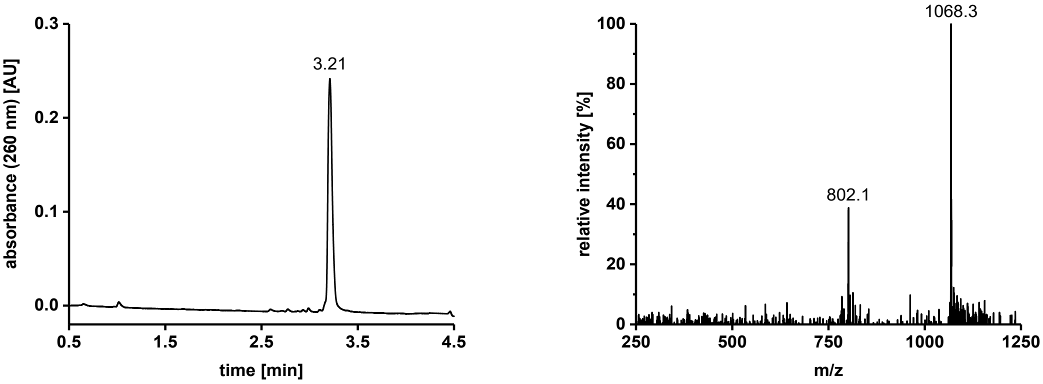

Figure S4: UPLC chromatogram (left) and ESI-MS spectrum (right) of purified PNA inhibitor conjugate **32a**.

Thiol-protected PNA acceptor conjugate 46

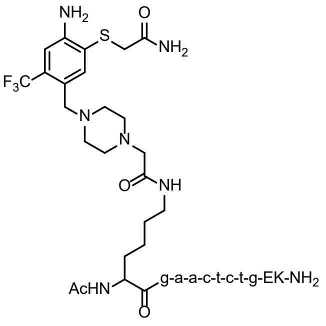

To a solution of PNA acceptor conjugate **23a** (100 nmol) in water (400 µL) and aq. $(NH_4)_2CO_3$ (0.1 M, 200 µL) was added DTT (4000 nmol, 40 eq, 1 M in 0.1 M aq. $(NH_4)_2CO_3$, 4 µL). After 30 min at 50 °C, the reaction mixture was cooled to room temperature and iodoacetamide (11200 nmol, 112 eq, 200 mM in 0.1 M aq. $(NH_4)_2CO_3$, 56 µL) was added. The obtained solution was agitated for 20 min at room temperature, followed by the addition of DTT solution (4250 nmol, 43 eq, 1 M in 0.1 M aq. $(NH_4)_2CO_3$, 4.25 µl). After another 10 min at room temperature, the product was purified by HPLC as previously described. Due to the acidic conditions during HPLC purification, a thiazine side product was formed which confirmed the selective alkylation of the thiol group.

Compound 46: Formula: $C_{121}H_{161}F_3N_{56}O_{32}S$ (3000.99 g · mol^{-1}). UPLC: t_r [min] = 2.54 (3 to 20% B1 in 4 min, column 1). ESI-MS: $m \cdot z^{-1}$ = 1001.3 $[M+3H]^{3+}$ (calc.: 1000.8), 751.2 $[M+4H]^{4+}$ (calc.: 750.8). Extinction coefficient: ε_{260} [L · mol^{-1} · cm^{-1}] = 86700. Yield: 20 nmol, 20%.

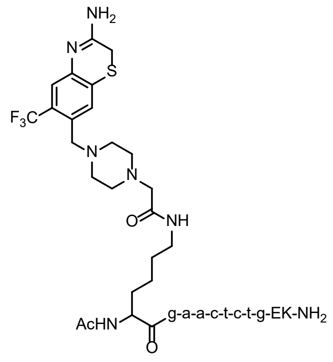

46-TA

<u>Compound **46-TA**</u>: Formula: $C_{121}H_{159}F_3N_{56}O_{31}S$ (2982.97 g · mol^{-1}). UPLC: t_r [min] = 2.83 (3 to 20% B1 in 4 min, column 1). ESI-MS: $m \cdot z^{-1}$ = 995.3 $[M+3H]^{3+}$ (calc.: 994.8), 746.9 $[M+4H]^{4+}$ (calc.: 746.3). Extinction coefficient: ε_{260} [L · mol^{-1} · cm^{-1}] = 86700. Yield: 7 nmol, 7%.

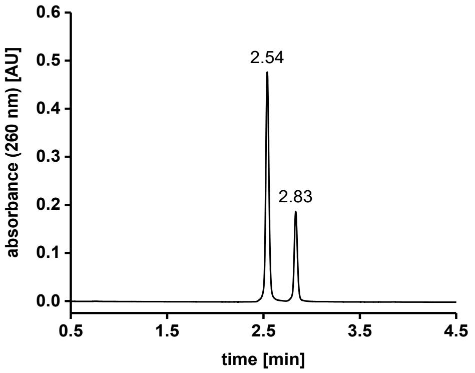

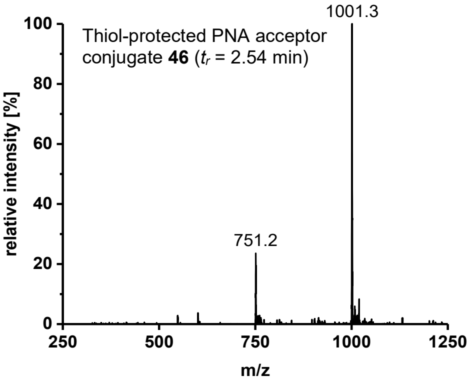

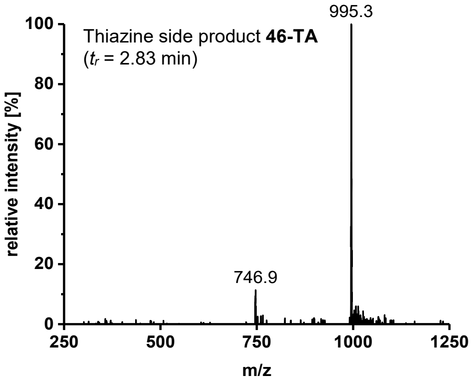

Figure S5: UPLC chromatogram (top left) and ESI-MS spectra (top right & bottom) of purified thiol-protected PNA acceptor conjugate **46** (t_r = 2.54 min) with thiazine side product **46-TA** (t_r = 2.83 min).

Thiol-free PNA acceptor conjugate 54

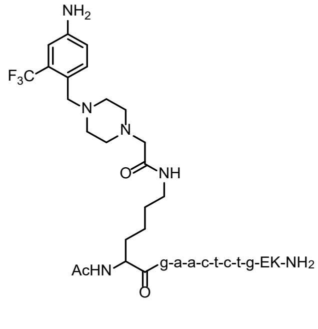

PNA acceptor conjugate **54** was synthesized by Fmoc-SPS according to the general procedure (scale: 5 µmol). Ac-Lys(Fmoc)-OH was used to introduce the *N*-terminal amino acid unit. After Fmoc removal from the lysine side chain with piperidine in DMF (1:4, v/v; 1 x 3 min, 1 x 7 min), compound **53** (0.2 M in DMF, 4 eq) was coupled in presence of HCTU (3.6 eq) and NMM (8 eq) for 30 min at room temperature. This procedure was repeated once. Global deprotection, cleavage from the resin, and HPLC purification were performed as previously described.

Formula: $C_{119}H_{158}F_3N_{55}O_{31}$ (2911.92 g · mol^{-1}). UPLC: t_r [min] = 2.81 (3 to 30% B1 in 4 min, column 1). ESI-MS: $m \cdot z^{-1}$ = 971.7 $[M+3H]^{3+}$ (calc.: 971.6), 729.2 $[M+4H]^{4+}$ (calc.: 729.0). Extinction coefficient: ε_{260} [L · mol^{-1} · cm^{-1}] = 86700. Yield: 0.34 µmol, 7%.

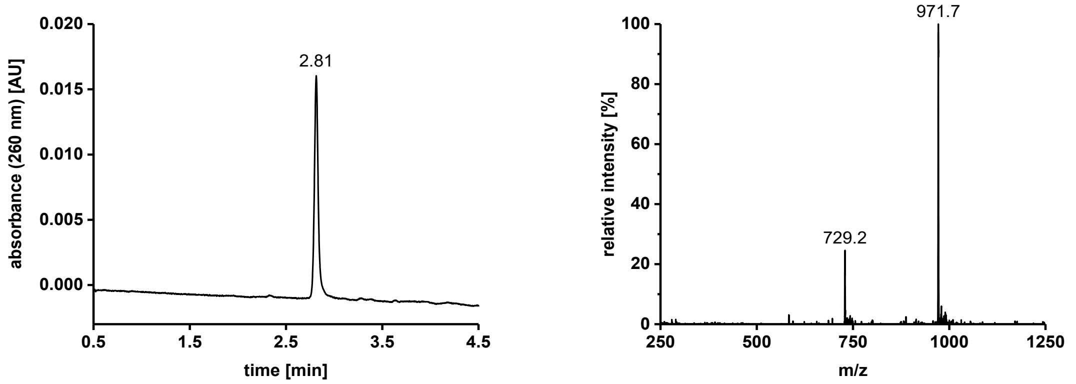

Figure S6: UPLC chromatogram (left) and ESI-MS spectrum (right) of purified thiol-free PNA acceptor conjugate **54**.

DNA acceptor conjugate 23b

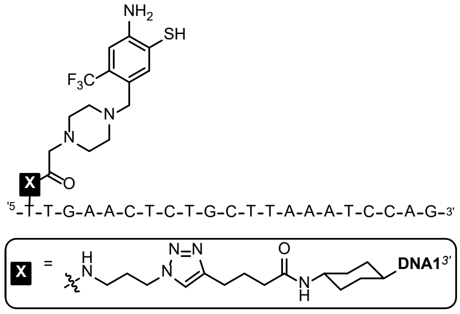

Diazide **28** (450 nmol, 5 eq, 5 mM in DMSO, 90 µL) was added to a solution of the alkyne-modified DNA oligonucleotide (90 nmol) in phosphate buffer (100 mM NaH_2PO_4/Na_2HPO_4, pH 7.4, 650 µL). Next, a freshly prepared mixture of $CuSO_4$ (450 nmol, 5 eq, 20 mM in water, 22.5 µL) and THPTA (2250 nmol, 25 eq, 50 mM in water, 45 µL) was added, followed by sodium ascorbate (9000 nmol, 100 eq, 100 mM in phosphate buffer, 90 µL). The reaction vessel was flushed with argon and the reaction mixture was heated to 30 °C for 3 h. Then, sodium hydrosulfide was added (2.5 mg) and the solution was agitated for another 15 min. The formed precipitate was removed by centrifugation (10 min, 16900 rcf, 4 °C) prior to HPLC purification of the DNA conjugate. After freeze-drying, product-containing fractions were dissolved in water (300 µL). Ammonium acetate (0.3 M in water, 300 µL) and isopropanol (1000 µL) were added successively, leading to DNA conjugate precipitation. The obtained precipitate was centrifuged (10 min, 16900 rcf, 4 °C), dried under vacuum, and dissolved in nuclease-free water.

Formula: $C_{234}H_{302}F_3N_{82}O_{130}P_{21}S$ (7082.88 g · mol^{-1}). UPLC: t_r [min] = 2.43 (disulfide, 10 to 40% B2 in 4 min, column 2). MALDI-TOF: $m \cdot z^{-1}$ = 7086 $[M+H]^+$ (calc.: 7084), 3544 $[M+2H]^{2+}$ (calc.: 3542). Extinction coefficient: ε_{260} [L · mol^{-1} · cm^{-1}] = 234500. Yield: 25 nmol, 28%.

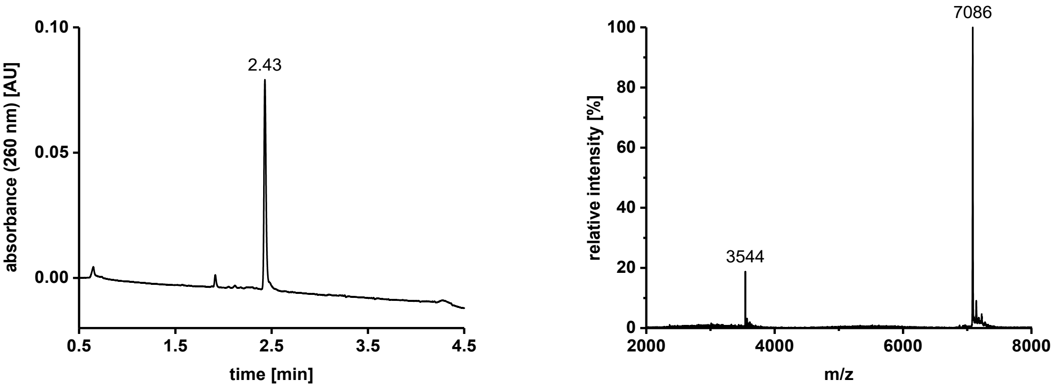

Figure S7: UPLC chromatogram (left) and MALDI-TOF mass spectrum (right) of purified DNA acceptor conjugate **23b**.

DNA acceptor conjugate 23c

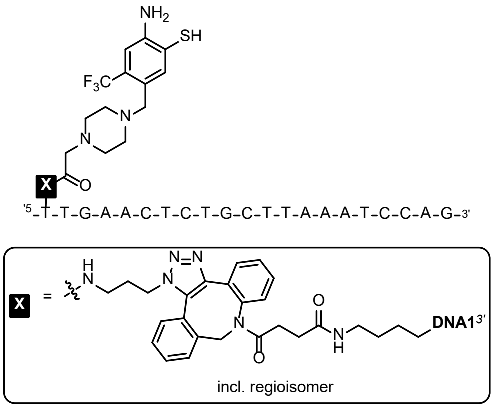

Diazide **28** (195 nmol, 3 eq, 5 mM in DMSO, 40 µL) was added to a solution of the DBCO-modified DNA oligonucleotide (65 nmol) in water (160 µL) and acetonitrile (120 µL). The reaction mixture was agitated at room temperature for 22 h, followed by HPLC purification of the formed DNA conjugate. After freeze-drying, product-containing fractions were dissolved in water (300 µL). Ammonium acetate (0.3 M in water, 300 µL) and isopropanol (1000 µL) were added successively, leading to DNA conjugate precipitation. The obtained precipitate was centrifuged (10 min, 16900 rcf, 4 °C), dried under vacuum, and dissolved in nuclease-free water.

Formula: $C_{246}H_{309}F_3N_{83}O_{131}P_{21}S$ (7264.07 g · mol^{-1}). UPLC: t_r [min] = 2.74 (disulfide, 10 to 50% B2 in 4 min, column 2). MALDI-TOF: $m \cdot z^{-1}$ = 7267 $[M+H]^+$ (calc.: 7265), 3632 $[M+2H]^{2+}$ (calc.: 3633). Extinction coefficient: ε_{260} [L · mol^{-1} · cm^{-1}] = 241500. Yield: 21 nmol, 32%.

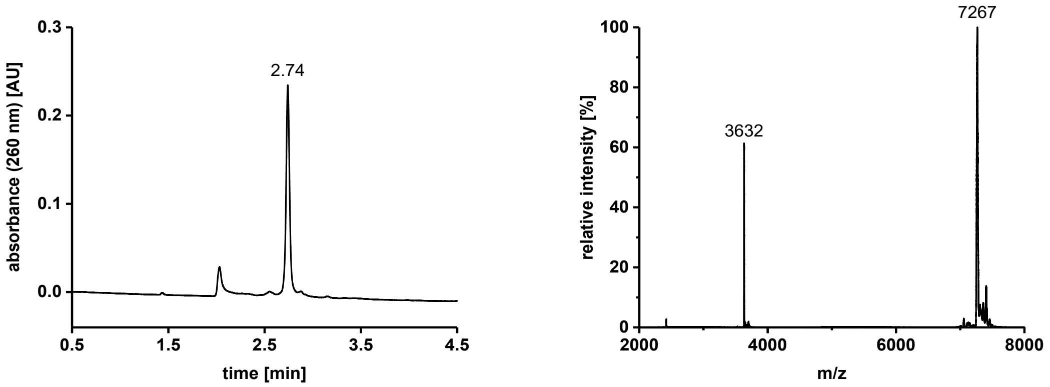

Figure S8: UPLC chromatogram (left) and MALDI-TOF mass spectrum (right) of purified DNA acceptor conjugate **23c**.

DNA donor conjugate 30a

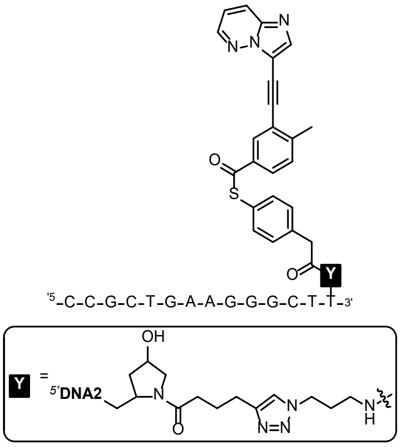

Compound **29** (750 nmol, 5 eq, 5 mM in DMSO, 150 µL) was added to a solution of the alkyne-modified DNA oligonucleotide (150 nmol) in phosphate buffer (100 mM NaH_2PO_4/Na_2HPO_4, pH 7.4, 337.5 µL) and DMSO (750 µL). Next, a freshly prepared mixture of $CuSO_4$ (750 nmol, 5 eq, 20 mM in water, 37.5 µL) and THPTA (3750 nmol, 25 eq, 50 mM in water, 75 µL) was added, followed by sodium ascorbate (15000 nmol, 100 eq, 100 mM in phosphate buffer, 150 µL). The reaction vessel was flushed with argon and the reaction mixture was heated to 60 °C for 6 h. Then, the formed DNA conjugate was precipitated by adding sodium acetate (3 M in water, 500 µL). The suspension was centrifuged (10 min, 16900 rcf, 4 °C) and the obtained pellet was purified by preparative HPLC. After freeze-drying, product-containing fractions were dissolved in water (300 µL). Ammonium acetate (0.3 M in water, 300 µL) and isopropanol (1000 µL) were added successively, leading to DNA conjugate precipitation. The obtained precipitate was centrifuged (10 min, 16900 rcf, 4 °C), dried under vacuum, and dissolved in nuclease-free water.

Formula: $C_{174}H_{211}N_{61}O_{90}P_{14}S$ (5062.59 g · mol^{-1}). UPLC: t_r [min] = 3.21 (10 to 40% B2 in 4 min, column 2). MALDI-TOF: $m \cdot z^{-1}$ = 5065 $[M+H]^+$ (calc.: 5064). Extinction coefficient: ε_{260} [L · mol^{-1} · cm^{-1}] = 168800. Yield: 56 nmol, 37%.

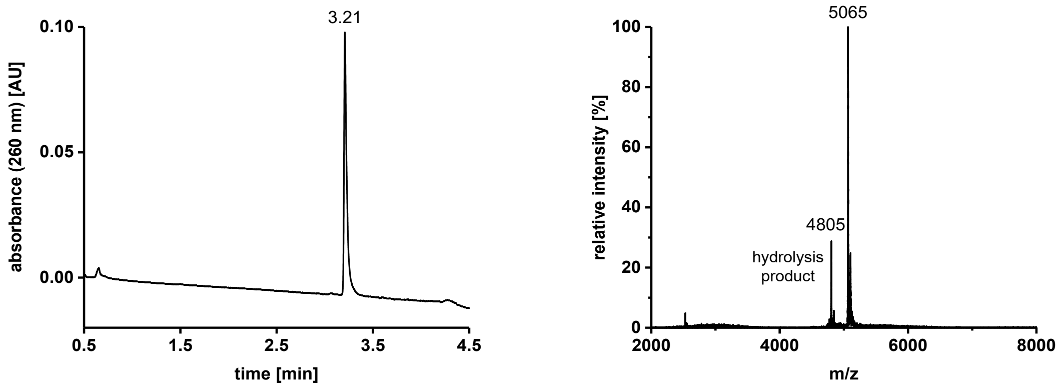

Figure S9: UPLC chromatogram (left) and MALDI-TOF mass spectrum (right) of purified DNA donor conjugate **30a**.

DNA donor conjugate 30b

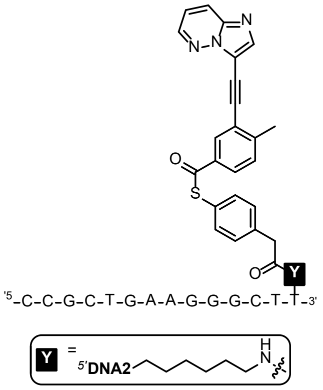

A mixture of MPAA-derived thioester **25** (10000 nmol, 100 eq, 100 mM in DMF, 100 µL), HATU (10000 nmol, 100 eq, 400 mM in DMF, 25 µL), and DIPEA (30000 nmol, 300 eq, 1200 mM in DMF, 25 µL) was added to a solution of the amino-modified DNA oligonucleotide (100 nmol) in water (200 µL). The reaction mixture was agitated at room temperature for 4 h. Then, water (650 µL) was added, leading to precipitation of excess amounts of MPAA-derived thioester **25**. The precipitate was removed by centrifugation (10 min, 16900 rcf, 4 °C) and the formed DNA conjugate was purified by preparative HPLC. After freeze-drying, product-containing fractions were dissolved in water (300 µL). Ammonium acetate (0.3 M in water, 300 µL) and isopropanol (1000 µL) were added successively, leading to DNA conjugate precipitation. The obtained precipitate was centrifuged (10 min, 16900 rcf, 4 °C), dried under vacuum, and dissolved in nuclease-free water.

Formula: $C_{166}H_{201}N_{57}O_{88}P_{14}S$ (4868.40 g · mol^{-1}). UPLC: t_r [min] = 2.92 (10 to 40% B2 in 4 min, column 2). MALDI-TOF: $m \cdot z^{-1}$ = 4868 [M+H]$^+$ (calc.: 4869). Extinction coefficient: ε_{260} [L · mol^{-1} · cm^{-1}] = 168800. Yield: 15 nmol, 15%.

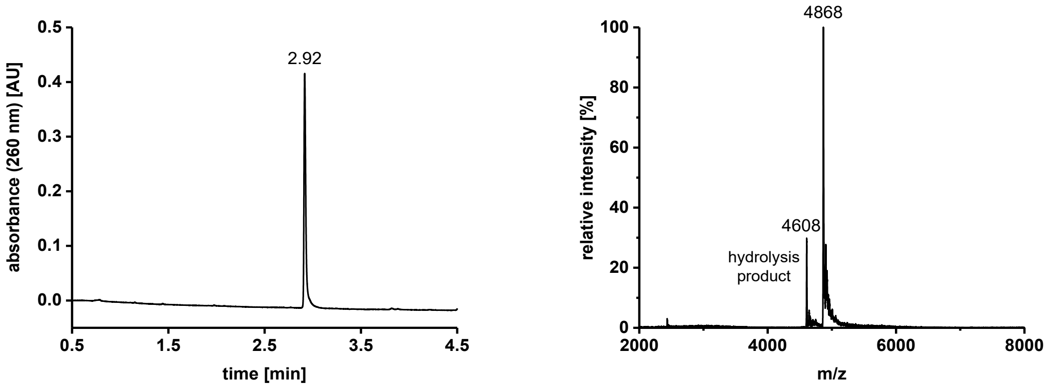

Figure S10: UPLC chromatogram (left) and MALDI-TOF mass spectrum (right) of purified DNA donor conjugate **30b**.

DNA inhibitor conjugate 32b

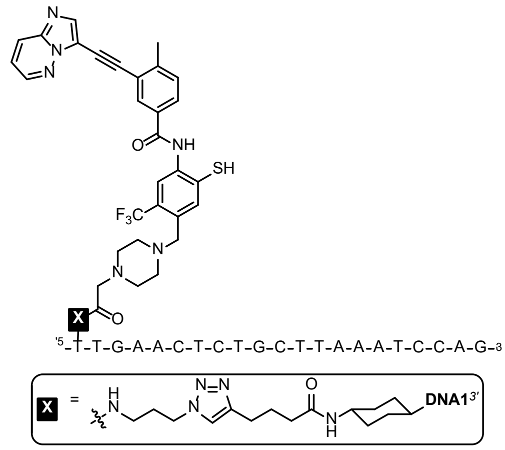

DNA acceptor conjugate **23b** (10 nmol) was dissolved in water (150 µL) and TCEP buffer 1 (100 mM NaH_2PO_4/Na_2HPO_4, 10 mM TCEP, pH 7.4, 100 µL). After 45 min at room temperature, DMSO (40 µL) and MPAA-derived thioester **25** (1000 nmol, 100 eq, 100 mM in DMSO, 10 µL) were added and the reaction mixture was agitated for further 21 h. The formed DNA conjugate was precipitated using sodium acetate (3 M in water, 30 µL) and isopropanol (1000 µL). The precipitate was centrifuged (10 min, 16900 rcf, 4 °C), dried under vacuum, and dissolved in nuclease-free water.

Formula: $C_{250}H_{311}F_3N_{85}O_{131}P_{21}S$ (7342.14 g · mol^{-1}). UPLC: t_r [min] = 3.18 (10 to 40% B2 in 4 min, column 2). MALDI-TOF: $m \cdot z^{-1}$ = 7344 $[M+H]^+$ (calc.: 7343). Extinction coefficient: ε_{260} [L · mol^{-1} · cm^{-1}] = 255600. Yield: 8.7 nmol, 87%.

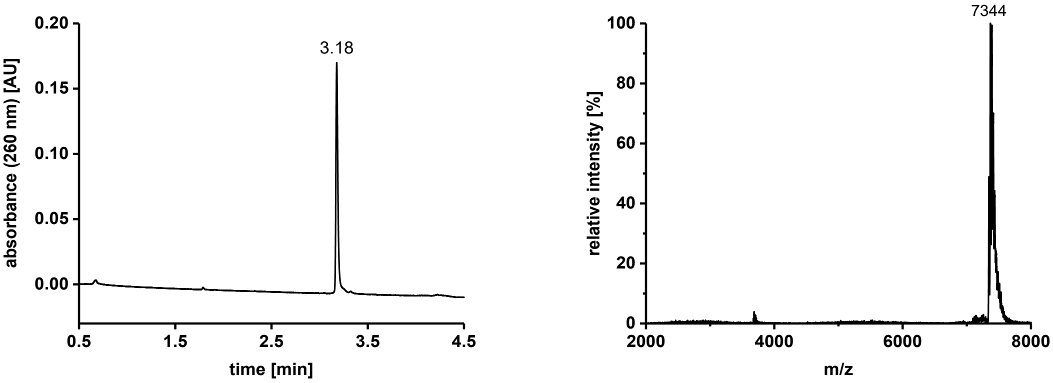

Figure S11: UPLC chromatogram (left) and MALDI-TOF mass spectrum (right) of purified DNA inhibitor conjugate **32b**.

DNA inhibitor conjugate 32c

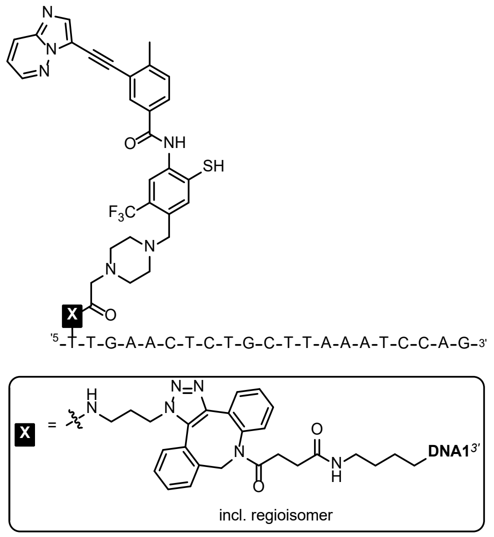

Diazide **28** (40 nmol, 2 eq, 5 mM in DMSO, 8 µL) was added to a solution of the DBCO-modified DNA oligonucleotide (20 nmol) in water (50 µL) and acetonitrile (42 µL). The reaction mixture was agitated at room temperature for 23 h, followed by the addition of TCEP buffer 1 (100 mM NaH_2PO_4/Na_2HPO_4, 10 mM TCEP, pH 7.4, 20 µL). After 45 min at room temperature, MPAA-derived thioester **25** (2000 nmol, 100 eq, 100 mM in DMSO, 20 µL) was added and the reaction mixture was agitated for further 23 h. Next, TCEP buffer 2 (100 mM NaH_2PO_4/Na_2HPO_4, 1 M TCEP, pH 7.2, 20 µL) was added and the reaction vessel was heated to 37 °C for 5 h. The formed DNA conjugate was purified by preparative HPLC. After freeze-drying, product-containing fractions were dissolved in water (300 µL). Ammonium acetate (0.3 M in water, 300 µL) and isopropanol (1000 µL) were added successively, leading to DNA conjugate precipitation. The obtained precipitate was centrifuged (10 min, 16900 rcf, 4 °C), dried under vacuum, and dissolved in nuclease-free water.

Formula: $C_{262}H_{318}F_3N_{86}O_{132}P_{21}S$ (7523.33 g · mol^{-1}). UPLC: t_r [min] = 3.07 (10 to 50% B2 in 4 min, column 2). MALDI-TOF: $m \cdot z^{-1}$ = 7526 $[M+H]^+$ (calc.: 7524), 3765 $[M+2H]^{2+}$ (calc.: 3763). Extinction coefficient: ε_{260} [L · mol^{-1} · cm^{-1}] = 262600. Yield: 3.6 nmol, 18%.

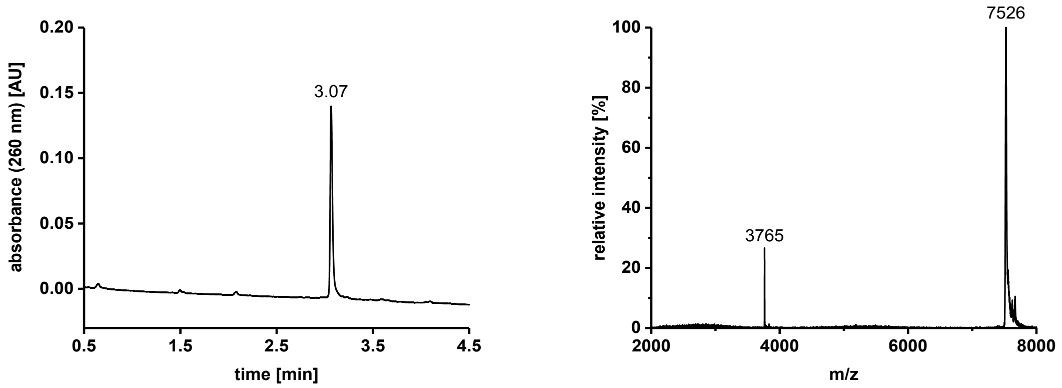

Figure S12: UPLC chromatogram (left) and MALDI-TOF mass spectrum (right) of purified DNA inhibitor conjugate **32c**.

Determination of dissociation constants

Nilotinib (1)

Replicate 1, K_d = 3.8 nM

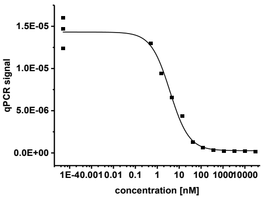

Replicate 2, K_d = 3.9 nM

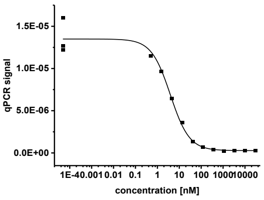

Replicate 3, K_d = 4.2 nM

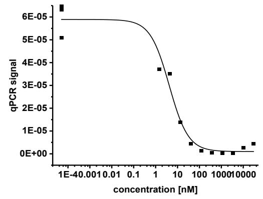

Replicate 4, K_d = 3.5 nM

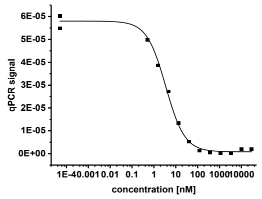

Nilotinib-SH (1a)

Replicate 1, K_d = 1142 nM

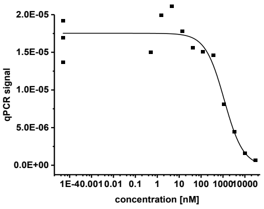

Replicate 2, K_d = 793 nM

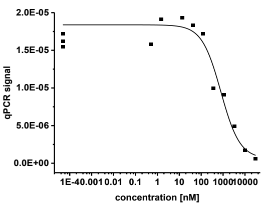

Replicate 3, K_d = 791 nM

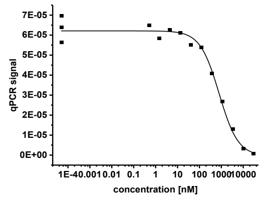

Replicate 4, K_d = 797 nM

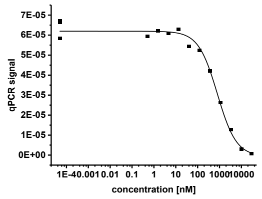

Ponatinib (2)

Replicate 1, K_d = 1.3 nM

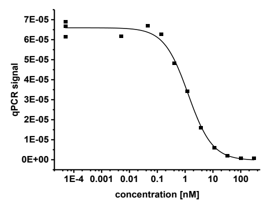

Replicate 2, K_d = 1.4 nM

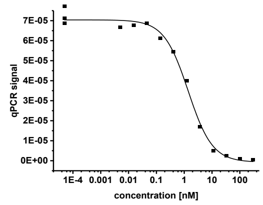

Replicate 3, K_d = 0.4 nM

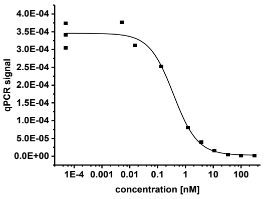

Replicate 4, K_d = 0.5 nM

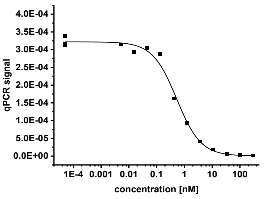

Ponatinib-SH (2a)

Replicate 1, K_d = 0.5 nM

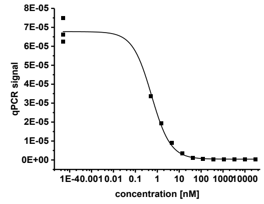

Replicate 2, K_d = 0.7 nM

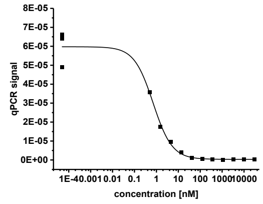

Replicate 3, K_d = 0.6 nM

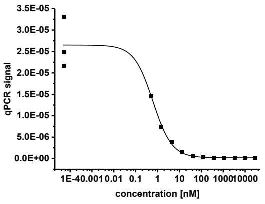

Replicate 4, K_d = 0.6 nM

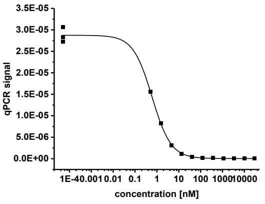

Ponatinib-BT (2b)

Replicate 1, K_d = 11.6 nM

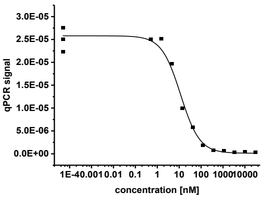

Replicate 2, K_d = 9.5 nM

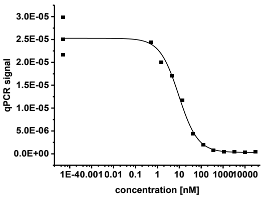

Replicate 3, K_d = 6.4 nM

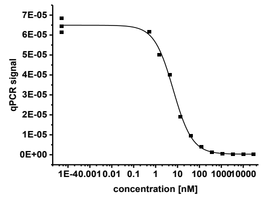

Replicate 4, K_d = 6.2 nM

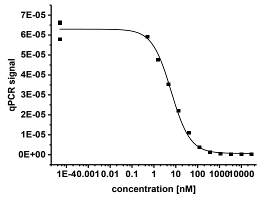

GZD824 (3)

Replicate 1, K_d = 1.0 nM Replicate 2, K_d = 0.7 nM Replicate 3, K_d = 0.3 nM Replicate 4, K_d = 0.4 nM

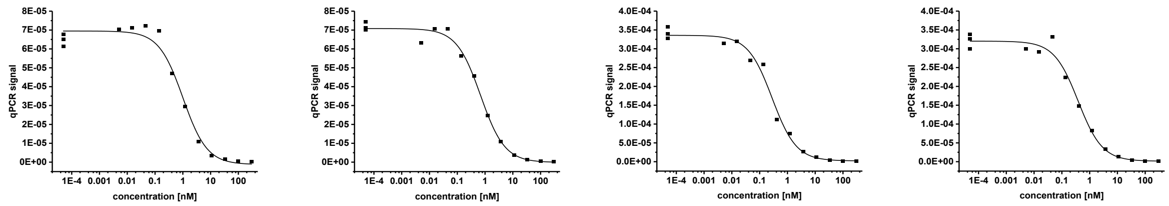

GZD824-SH (3a)

Replicate 1, K_d = 1.2 nM Replicate 2, K_d = 1.4 nM Replicate 3, K_d = 0.5 nM Replicate 4, K_d = 0.5 nM

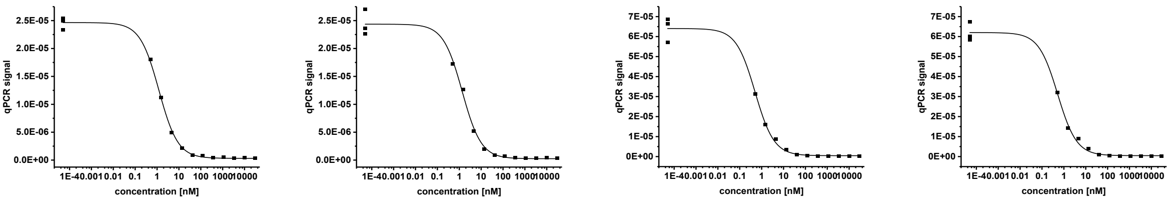

Figure S13: Dose-response curves of literature-known Bcr-Abl TKI and thiolated inhibitor derivatives against the non-phosphorylated Abl1 kinase obtained with the KdELECT® assay of DiscoverX. K_d values were determined using the Hill equation.

Determination of half-maximal inhibitory concentrations

**Table S1:** *IC_{50}* values of ponatinib (**2**), ponatinib-SH (**2a**) and ponatinib-BT (**2b**) on K562 cells, determined by alamarBlue[TM] assay.	
	IC_{50} [nM]
ponatinib (**2**)	3.4 ± 0.2
ponatinib-SH (**2a**)	354 ± 28
ponatinib-BT (**2b**)	120 ± 5

Ponatinib (2)

Replicate 1, *IC_{50}* = 3.5 nM

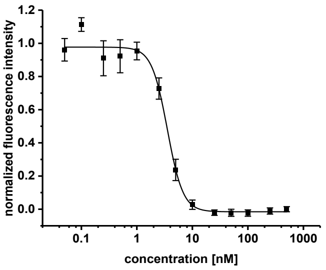

Replicate 2, *IC_{50}* = 3.6 nM

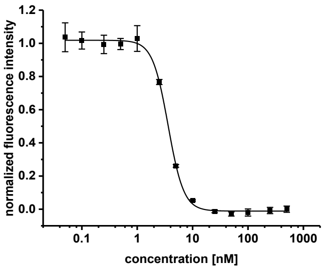

Replicate 3, *IC_{50}* = 3.1 nM

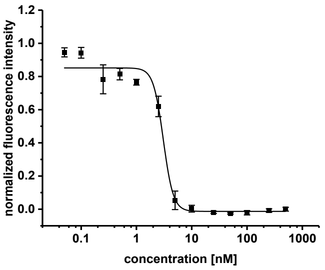

Ponatinib-SH (2a)

Replicate 1, *IC_{50}* = 392 nM

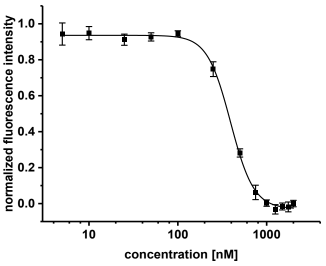

Replicate 2, *IC_{50}* = 343 nM

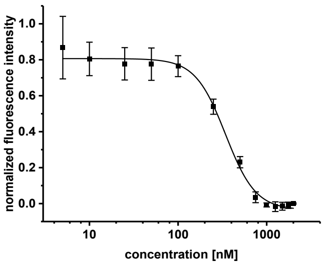

Replicate 3, *IC_{50}* = 326 nM

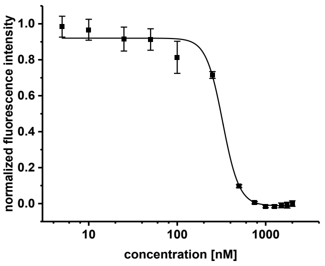

Ponatinib-BT (2b)

Replicate 1, *IC_{50}* = 118 nM

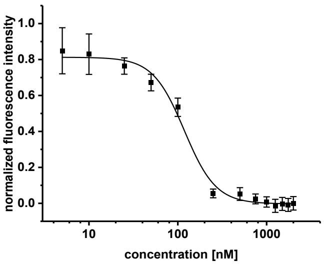

Replicate 2, *IC_{50}* = 115 nM

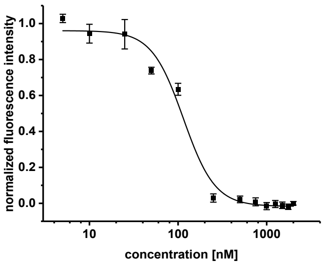

Replicate 3, *IC_{50}* = 126 nM

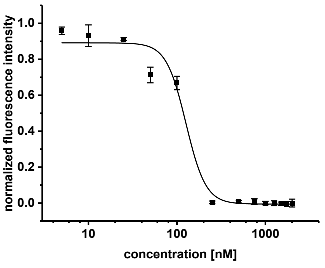

Figure S14: Dose-response curves of ponatinib (**2**), ponatinib-SH (**2a**), and ponatinib-BT (**2b**) on K562 cells. Cells were treated with serial dilutions of test compounds for 96 h, followed by viability analysis with alamarBlue[TM] reagent. *IC_{50}* values were determined using the Hill equation.

Determination of molar extinction coefficients

Disulfide 44 (reduced form)

$\varepsilon_{260,exp.}$ = 5100 L · mol^{-1} · cm^{-1}

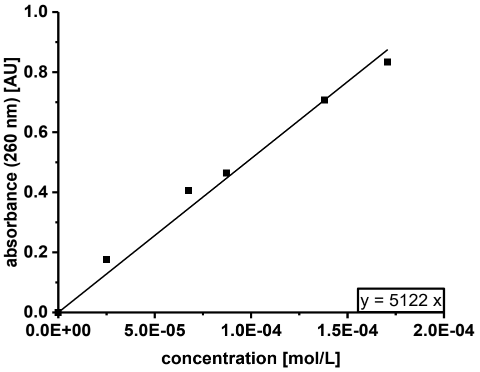

MPA-derived thioester 24

$\varepsilon_{260,exp.}$ = 22400 L · mol^{-1} · cm^{-1}

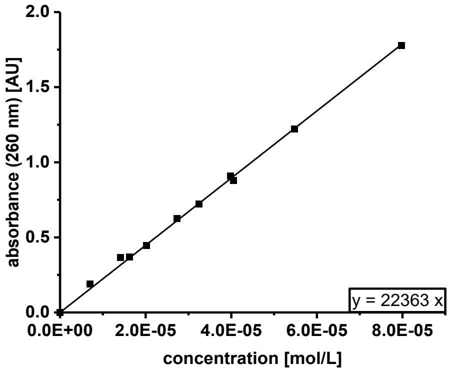

MPAA-derived thioester 25

$\varepsilon_{260,exp.}$ = 22000 L · mol^{-1} · cm^{-1}

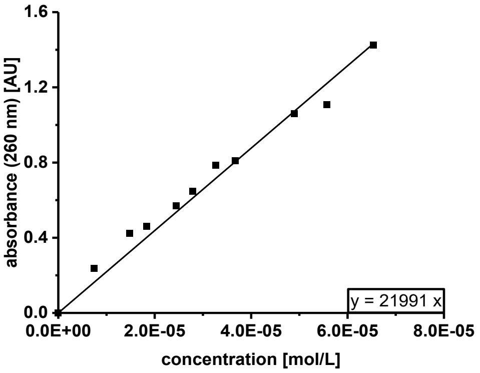

Ponatinib-BT (2b)

$\varepsilon_{260,exp.}$ = 26200 L · mol^{-1} · cm^{-1}

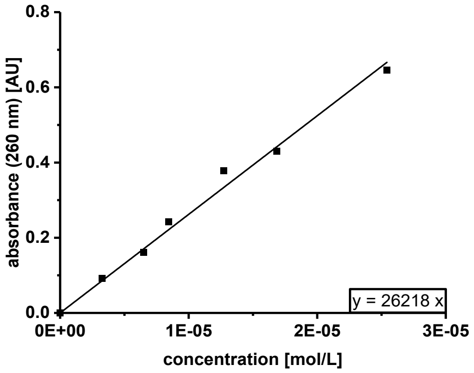

Figure S15: Plots of absorbance at 260 nm against concentration of the test compound. Molar extinction coefficients ($\varepsilon_{260,exp.}$) were taken as the slope of the linear regression line.

Determination of melting temperatures

PNA acceptor conjugate 23a

T_m = 48.7 °C

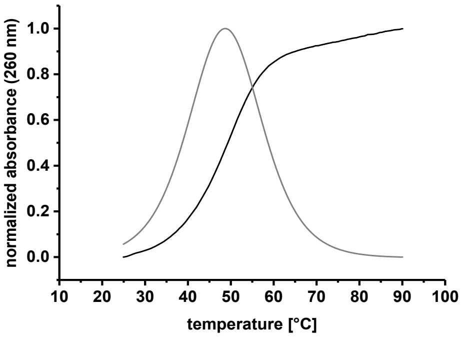

MPA-derived PNA donor conjugate 27a

T_m = 43.0 °C

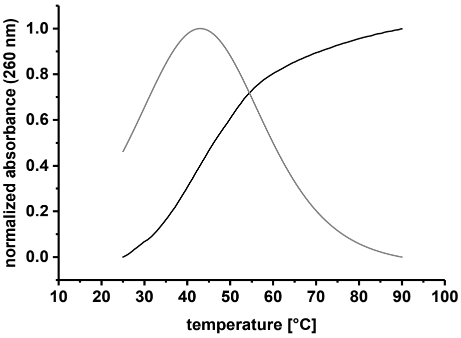

MPAA-derived PNA donor conjugate 27b

T_m = 43.1 °C

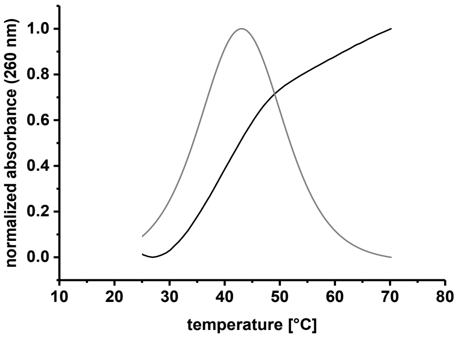

Figure S16: Normalized melting curves of PNA conjugates (black) and first derivatives of the corresponding Boltzmann fit (grey). Melting temperatures (T_m) were taken as the maximum of the first derivatives. *Conditions:* 10 mM NaH_2PO_4/Na_2HPO_4, 100 mM NaCl, pH 7.4, [PNA conjugate] = 1 µM, [RNA] = 1 µM, 25 – 90 °C.

DNA acceptor conjugate 23b

T_m = 60.3 °C

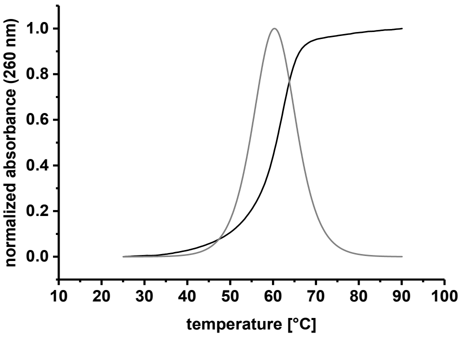

DNA acceptor conjugate 23c

T_m = 59.6 °C

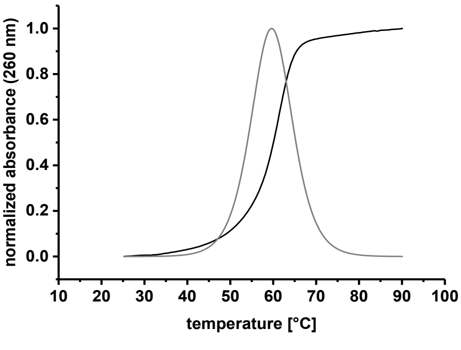

DNA donor conjugate 30a

T_m = 60.0 °C

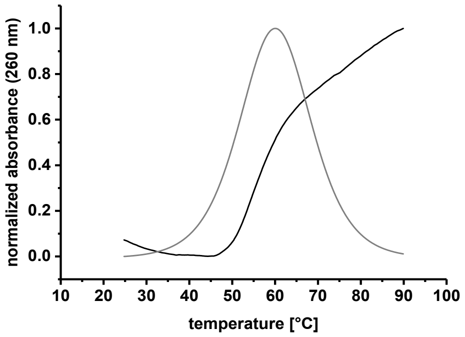

DNA donor conjugate 30b

T_m = 60.3 °C

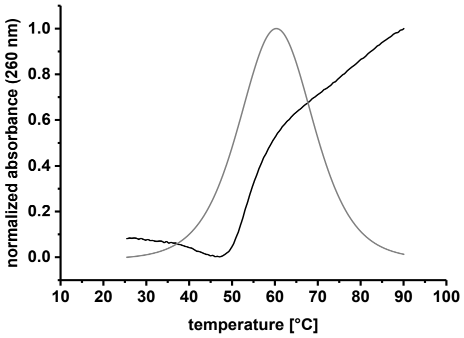

Figure S17: Normalized melting curves of DNA conjugates (black) and first derivatives of the corresponding Boltzmann fit (grey). Melting temperatures (T_m) were taken as the maximum of the first derivatives. *Conditions:* 10 mM NaH_2PO_4/Na_2HPO_4, 100 mM NaCl, pH 7.4, [DNA conjugate] = 1 µM, [RNA] = 1 µM, 25 – 90 °C.

Synthetic RNAs

BCR-ABL RNA:	PNA/PNA	5'-GCAGAGUUCAAAAGCCCU-3'
	DNA/DNA	5'-CACUGGAUUUAAGCAGAGUUCAAAAGCCCUUCAGCGGCC-3'
	PNA/DNA	5'-CACUGGAUUUAAGCAGAGUUCAAAAGCCCUUCAGCGGCC-3'

For the spacer nucleotide screening, the following modified RNA sequences were used (unpaired spacer nucleotides are underlined):

DNA/DNA:	0 nt	5'-CACUGGAUUUAAGCAGAGUUCAAAAGCCCUUCAGCGGCC-3'
	1 nt	5'-CUGGAUUUAAGCAGAGUUCAAGAAGCCCUUCAGCGG-3'
	2 nt	5'-CUGGAUUUAAGCAGAGUUCAAGUAAGCCCUUCAGCGG-3'
	3 nt	5'-CUGGAUUUAAGCAGAGUUCAAGUUAAGCCCUUCAGCGG-3'
PNA/DNA:	0 nt	5'-GCAGAGUUCAAGCCCUUCAGCGGCC-3'
	1 nt	5'-GCAGAGUUCAAAGCCCUUCAGCGGCC-3'
	2 nt	5'-CACUGGAUUUAAGCAGAGUUCAAAAGCCCUUCAGCGGCC-3'
	3 nt	5'-GCAGAGUUCAUAAAGCCCUUCAGCGGCC-3'

Transfer reactions of PNA/PNA systems

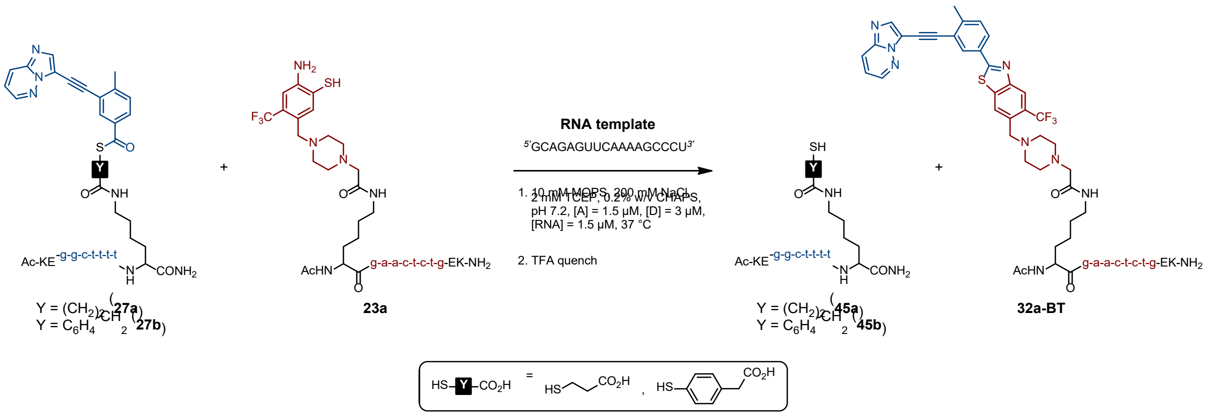

Scheme S7: Transfer reactions between PNA-based acceptor (A) and donor (D) conjugates. The benzothiazole derivative was formed due to the addition of TFA to quench the reaction prior to UPLC analysis.

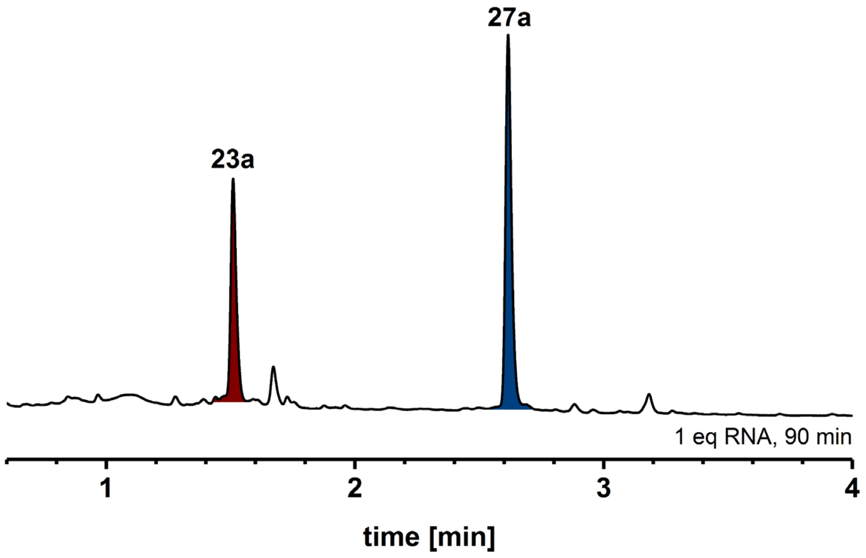

Figure S18: Representative UPLC trace at 260 nm for the attempted transfer reaction between PNA acceptor conjugate **23a** (A) and PNA donor conjugate **27a** (D) after 90 min in the presence of 1.5 µM RNA template (5'-GCAGAGUUCAAAAGCCCU-3'). Neither product formation nor thioester hydrolysis were observed. *Conditions:* 10 mM MOPS, 200 mM NaCl, 2 mM TCEP, 0.2% w/v CHAPS, pH 7.2, [A] = 1.5 µM, [D] = 3 µM, [RNA] = 1.5 µM, 37 °C. *Gradient:* 3 to 40% B1 in 4 min, column 1 (UPLC-MS).

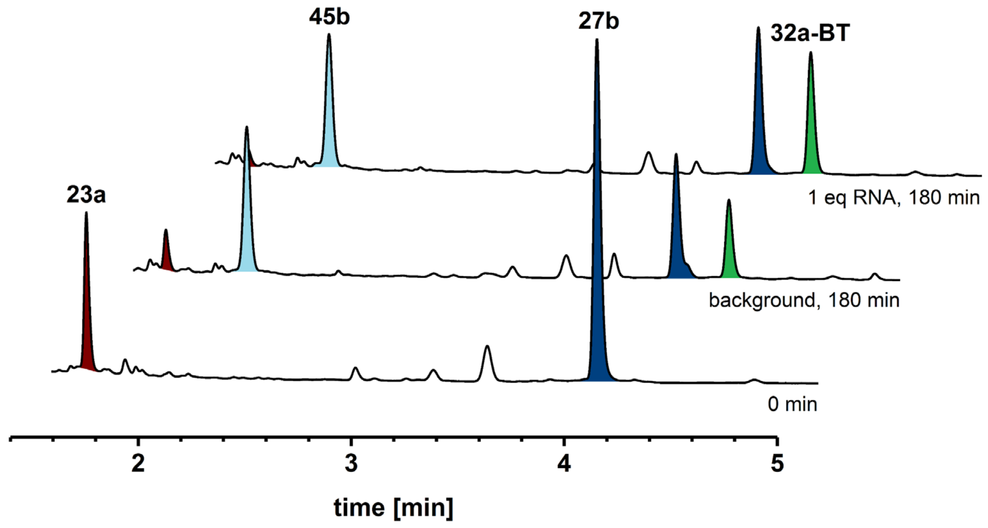

Figure S19: Representative UPLC traces at 260 nm for the formation of transfer product **32a-BT** upon reaction between PNA acceptor conjugate **23a** (A) and PNA donor conjugate **27b** (D) at t = 0 min (bottom) and after 180 min in the absence (middle) or presence (top) of 1.5 µM RNA template (5'-GCAGAGUUCAAAAGCCCU-3'). *Conditions:* 10 mM MOPS, 200 mM NaCl, 2 mM TCEP, 0.2% w/v CHAPS, pH 7.2, [A] = 1.5 µM, [D] = 3 µM, [RNA] = 0 / 1.5 µM, 37 °C. *Gradient:* 3 to 30% B1 in 5 min, column 1 (UPLC-MS).

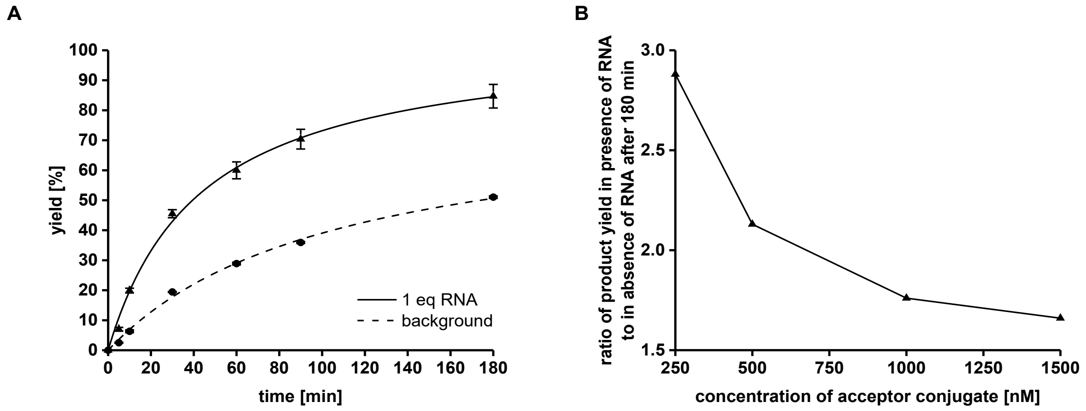

Figure S20: A) Time course of product formation upon reaction between PNA acceptor conjugate **23a** (A) and PNA donor conjugate **27b** (D) in the absence (dotted) and presence (solid) of RNA template ([A] = 1.5 µM). B) Ratio of product yield in presence of RNA template to in absence of RNA template after 180 min with different concentrations of PNA acceptor conjugate **23a**. *Conditions:* 10 mM MOPS, 200 mM NaCl, 2 mM TCEP, 0.2% w/v CHAPS, pH 7.2, [D] = 2 · [A], [RNA] = [A], 37 °C, 180 min. RNA = 5'-GCAGAGUUCAAAAGCCCU-3'.

Transfer reactions of DNA/DNA and PNA/DNA systems

Table S2: Product yields after 180 min for the indicated transfer reactions in absence of RNA template. *Conditions:* 10 mM MOPS, 200 mM NaCl, 2 mM TCEP, pH 7.2, [A] = 1 µM, [D] = 2 µM, 37 °C.

	DNA donor **30a**				
	Replicate 1	Replicate 2	Replicate 3	Mean value	Standard deviation
PNA acceptor **23a**	7.00	8.30	5.58	**6.96**	**1.11**
DNA acceptor **23b**	14.27	15.08	8.58	**12.64**	**2.89**
DNA acceptor **23c**	11.05	10.94	11.16	**11.05**	**0.09**
	DNA donor **30b**				
	Replicate 1	Replicate 2	Replicate 3	Mean value	Standard deviation
PNA acceptor **23a**	5.18	6.06	5.45	**5.56**	**0.37**
DNA acceptor **23b**	9.53	10.06	3.11	**7.57**	**3.16**
DNA acceptor **23c**	6.93	7.69	7.89	**7.50**	**0.41**

Table S3: Product yields after 180 min for the indicated transfer reactions in presence of RNA template. *Conditions:* 10 mM MOPS, 200 mM NaCl, 2 mM TCEP, pH 7.2, [A] = 1 µM, [D] = 2 µM, [RNA] = 1 µM, 37 °C.

	PNA acceptor **23a** + DNA donor **30a**				
	Replicate 1	Replicate 2	Replicate 3	Mean value	Standard deviation
0 nt	32.12	31.57	25.08	**29.59**	**3.20**
1 nt	42.33	40.31	37.86	**40.17**	**1.83**
2 nt	48.15	49.41	41.43	**46.33**	**3.50**
3 nt	40.62	40.50	31.24	**37.45**	**4.39**
	DNA acceptor **23b** + DNA donor **30a**				
	Replicate 1	Replicate 2	Replicate 3	Mean value	Standard deviation
0 nt	34.01	38.85	28.79	**33.88**	**4.11**
1 nt	40.29	44.45	37.86	**40.87**	**2.72**
2 nt	40.05	46.90	39.02	**41.99**	**3.50**
3 nt	40.42	46.35	31.24	**39.34**	**6.22**
	DNA acceptor **23c** + DNA donor **30a**				
	Replicate 1	Replicate 2	Replicate 3	Mean value	Standard deviation
0 nt	46.37	29.64	36.03	**37.35**	**6.89**
1 nt	45.41	37.67	34.67	**39.25**	**4.52**
2 nt	41.78	37.06	37.71	**38.85**	**2.09**
3 nt	36.98	38.50	32.41	**35.96**	**2.59**

Table S4: Product yields after 180 min for the indicated transfer reactions in presence of RNA template. *Conditions:* 10 mM MOPS, 200 mM NaCl, 2 mM TCEP, pH 7.2, [A] = 1 µM, [D] = 2 µM, [RNA] = 1 µM, 37 °C.

	PNA acceptor **23a** + DNA donor **30b**				
	Replicate 1	Replicate 2	Replicate 3	Mean value	Standard deviation
0 nt	15.54	19.29	15.71	**16.85**	**1.73**
1 nt	30.77	35.66	34.24	**33.56**	**2.05**
2 nt	47.22	47.98	41.37	**45.52**	**2.95**
3 nt	38.18	41.80	35.13	**38.37**	**2.73**
	DNA acceptor **23b** + DNA donor **30b**				
	Replicate 1	Replicate 2	Replicate 3	Mean value	Standard deviation
0 nt	21.02	30.30	18.26	**23.19**	**5.15**
1 nt	30.05	35.06	24.21	**29.77**	**4.43**
2 nt	31.55	38.34	29.89	**33.26**	**3.66**
3 nt	30.58	38.36	28.56	**32.50**	**4.22**
	DNA acceptor **23c** + DNA donor **30b**				
	Replicate 1	Replicate 2	Replicate 3	Mean value	Standard deviation
0 nt	36.98	23.51	27.44	**29.31**	**5.66**
1 nt	37.74	30.46	27.06	**31.75**	**4.45**
2 nt	39.30	33.38	33.28	**35.32**	**2.81**
3 nt	39.24	34.69	37.53	**37.15**	**1.88**

Table S5: Product yields after 720 min for the indicated transfer reactions in presence or absence of RNA template. *Conditions:* 10 mM MOPS, 200 mM NaCl, 2 mM TCEP, pH 7.2, [A] = 1 µM, [D] = 2 µM, [RNA] = 0 / 1 µM, 37 °C.

	PNA acceptor **23a** + DNA donor **30b**				
	Replicate 1	Replicate 2	Replicate 3	Mean value	Standard deviation
no template	13.20	16.42	5.54	**11.72**	**4.56**
2 nt	65.90	56.57	54.70	**59.06**	**4.90**
	DNA acceptor **23c** + DNA donor **30b**				
	Replicate 1	Replicate 2	Replicate 3	Mean value	Standard deviation
no template	21.20	25.34	22.94	**23.16**	**1.70**
2 nt	63.10	60.96	61.05	**61.70**	**0.99**

To determine the increase of product formation relative to seamless annealing, we used the increase factor which was calculated according to equation 4. The yield of a certain experiment (0 nt – 3 nt) was divided by the corresponding yield of the 0 nt experiment within the indicated replicate (replicate 1 – 3).

$$(4) \qquad increase\ factor\ [-] = \frac{yield_{Replicate\ i}}{yield\ (0\ nt)_{Replicate\ i}}$$

i = replicate index (1-3)

Table S6: Increase factors after 180 min for the indicated transfer reactions in presence of RNA template. *Conditions:* 10 mM MOPS, 200 mM NaCl, 2 mM TCEP, pH 7.2, [A] = 1 µM, [D] = 2 µM, [RNA] = 1 µM, 37 °C.

	PNA acceptor **23a** + DNA donor **30a**				
	Replicate 1	Replicate 2	Replicate 3	Mean value	Standard deviation
0 nt	1.00	1.00	1.00	**1.00**	**0.00**
1 nt	1.32	1.28	1.51	**1.37**	**0.10**
2 nt	1.50	1.57	1.65	**1.57**	**0.06**
3 nt	1.26	1.28	1.25	**1.26**	**0.02**
	DNA acceptor **23b** + DNA donor **30a**				
	Replicate 1	Replicate 2	Replicate 3	Mean value	Standard deviation
0 nt	1.00	1.00	1.00	**1.00**	**0.00**
1 nt	1.18	1.14	1.32	**1.21**	**0.07**
2 nt	1.18	1.21	1.36	**1.25**	**0.08**
3 nt	1.19	1.19	1.09	**1.16**	**0.05**
	DNA acceptor **23c** + DNA donor **30a**				
	Replicate 1	Replicate 2	Replicate 3	Mean value	Standard deviation
0 nt	1.00	1.00	1.00	**1.00**	**0.00**
1 nt	0.98	1.27	0.96	**1.07**	**0.14**
2 nt	0.90	1.25	1.05	**1.07**	**0.14**
3 nt	0.80	1.30	0.90	**1.00**	**0.22**

Table S7: Increase factors after 180 min for the indicated transfer reactions in presence of RNA template. *Conditions:* 10 mM MOPS. 200 mM NaCl. 2 mM TCEP. pH 7.2. [A] = 1 µM. [D] = 2 µM. [RNA] = 1 µM. 37 °C.

	PNA acceptor **23a** + DNA donor **30b**				
	Replicate 1	Replicate 2	Replicate 3	Mean value	Standard deviation
0 nt	1.00	1.00	1.00	**1.00**	**0.00**
1 nt	1.98	1.85	2.18	**2.00**	**0.14**
2 nt	3.04	2.49	2.63	**2.72**	**0.23**
3 nt	2.46	2.17	2.24	**2.29**	**0.12**
	DNA acceptor **23b** + DNA donor **30b**				
	Replicate 1	Replicate 2	Replicate 3	Mean value	Standard deviation
0 nt	1.00	1.00	1.00	**1.00**	**0.00**
1 nt	1.43	1.16	1.33	**1.30**	**0.11**
2 nt	1.50	1.27	1.64	**1.47**	**0.15**
3 nt	1.45	1.27	1.56	**1.43**	**0.12**
	DNA acceptor **23c** + DNA donor **30b**				
	Replicate 1	Replicate 2	Replicate 3	Mean value	Standard deviation
0 nt	1.00	1.00	1.00	**1.00**	**0.00**
1 nt	1.02	1.30	0.99	**1.10**	**0.14**
2 nt	1.06	1.42	1.21	**1.23**	**0.15**
3 nt	1.06	1.48	1.37	**1.30**	**0.18**

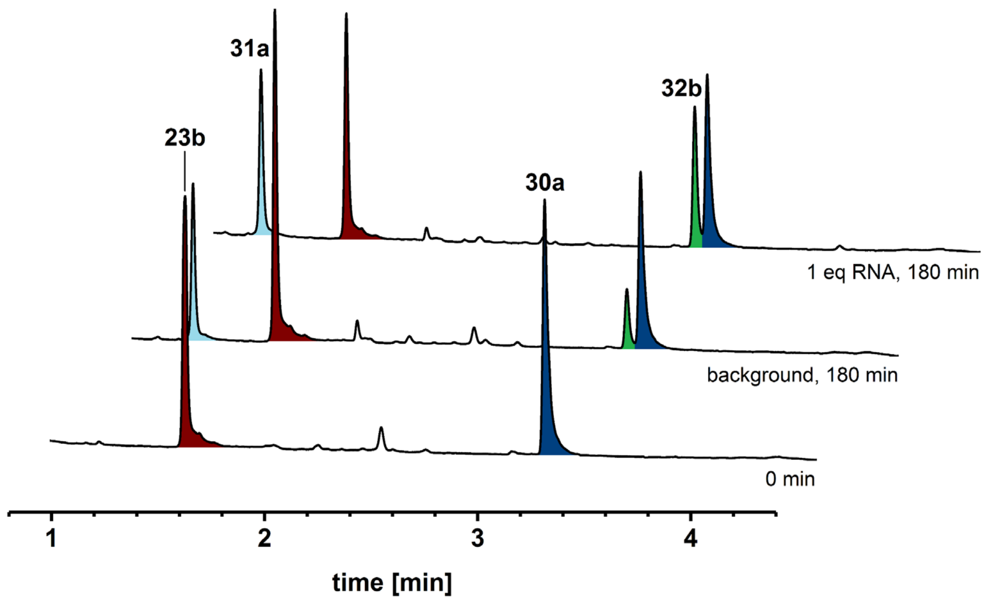

Figure S21: Representative UPLC traces at 260 nm for the formation of transfer product **32b** upon reaction between DNA acceptor conjugate **23b** (A) and DNA donor conjugate **30a** (D) at t = 0 min (bottom) and after 180 min in the absence (middle) or presence (top) of 1 µM RNA template (5'-CACUGGAUUUAAGCAGAGUUCAA-AAGCCCUUCAGCGGCC-3'). *Conditions:* 10 mM MOPS, 200 mM NaCl, 2 mM TCEP, pH 7.2, [A] = 1 µM, [D] = 2 µM, [RNA] = 0 / 1 µM, 37 °C. *Gradient:* 10 to 40% B2 in 4 min, column 2 (UPLC-UV/Vis).

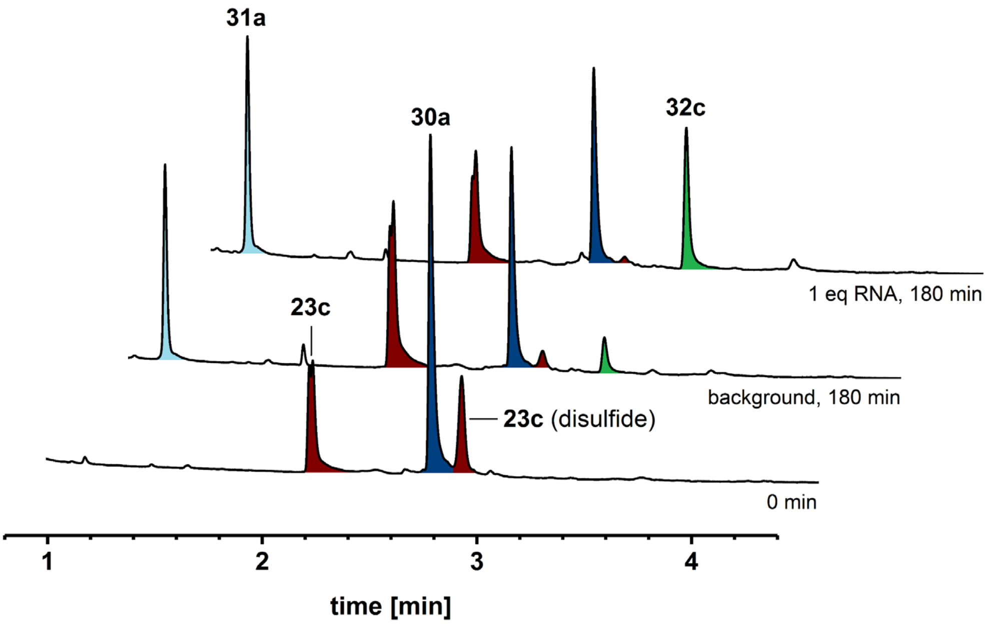

Figure S22: Representative UPLC traces at 260 nm for the formation of transfer product **32c** upon reaction between DNA acceptor conjugate **23c** (A) and DNA donor conjugate **30a** (D) at t = 0 min (bottom) and after 180 min in the absence (middle) or presence (top) of 1 µM RNA template (5'-CACUGGAUUUAAGCAGAGUUCAA-AAGCCCUUCAGCGGCC-3'). *Conditions:* 10 mM MOPS, 200 mM NaCl, 2 mM TCEP, pH 7.2, [A] = 1 µM, [D] = 2 µM, [RNA] = 0 / 1 µM, 37 °C. *Gradient:* 10 to 50% B2 in 4 min, column 2 (UPLC-UV/Vis).

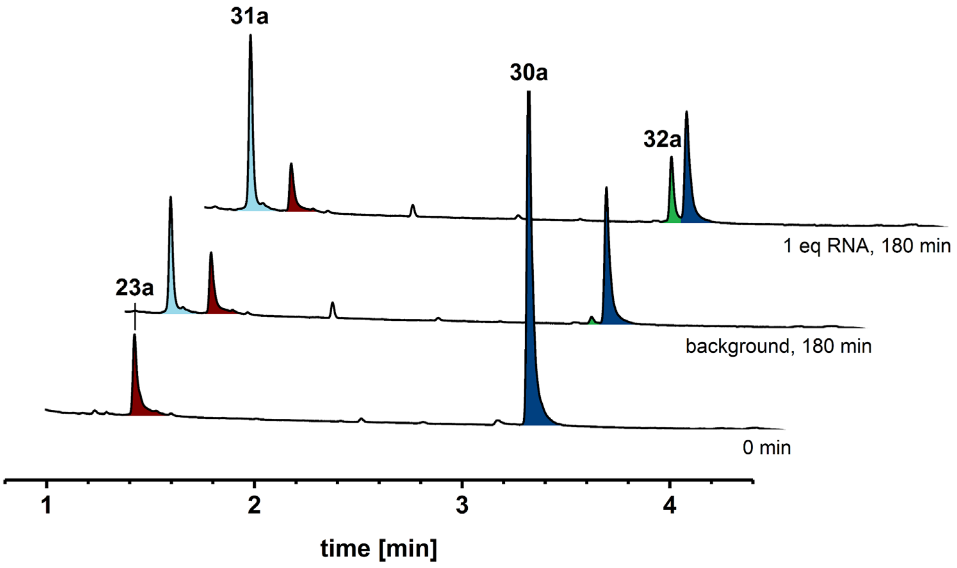

Figure S23: Representative UPLC traces at 260 nm for the formation of transfer product **32a** upon reaction between PNA acceptor conjugate **23a** (A) and DNA donor conjugate **30a** (D) at t = 0 min (bottom) and after 180 min in the absence (middle) or presence (top) of 1 µM RNA template (5'-CACUGGAUUUAAGCAGAGUUCAA-AAGCCCUUCAGCGGCC-3'). *Conditions:* 10 mM MOPS, 200 mM NaCl, 2 mM TCEP, pH 7.2, [A] = 1 µM, [D] = 2 µM, [RNA] = 0 / 1 µM, 37 °C. *Gradient:* 10 to 40% B2 in 4 min, column 2 (UPLC-UV/Vis).

Control experiment using a thiol-protected PNA acceptor conjugate

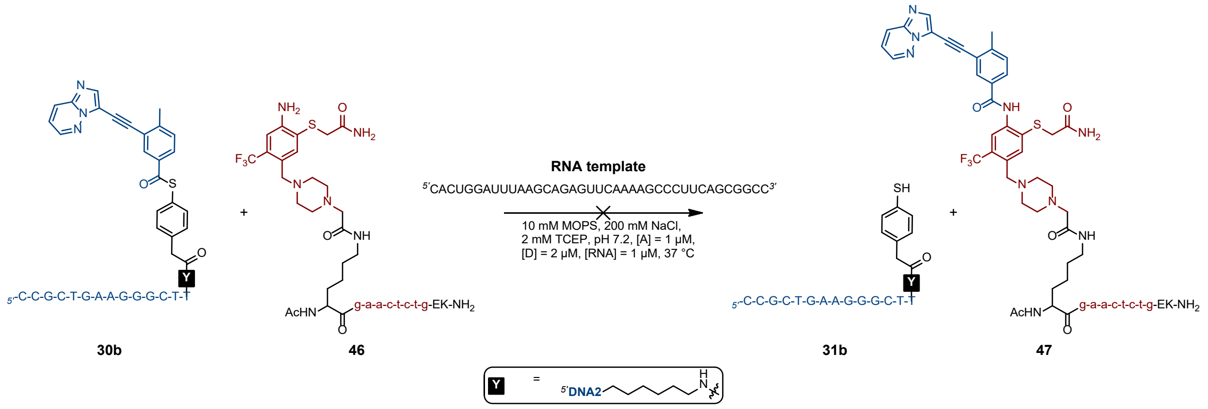

Scheme S8: Attempted transfer reaction between thiol-protected PNA acceptor conjugate **46** (A) and DNA donor conjugate **30b** (D).

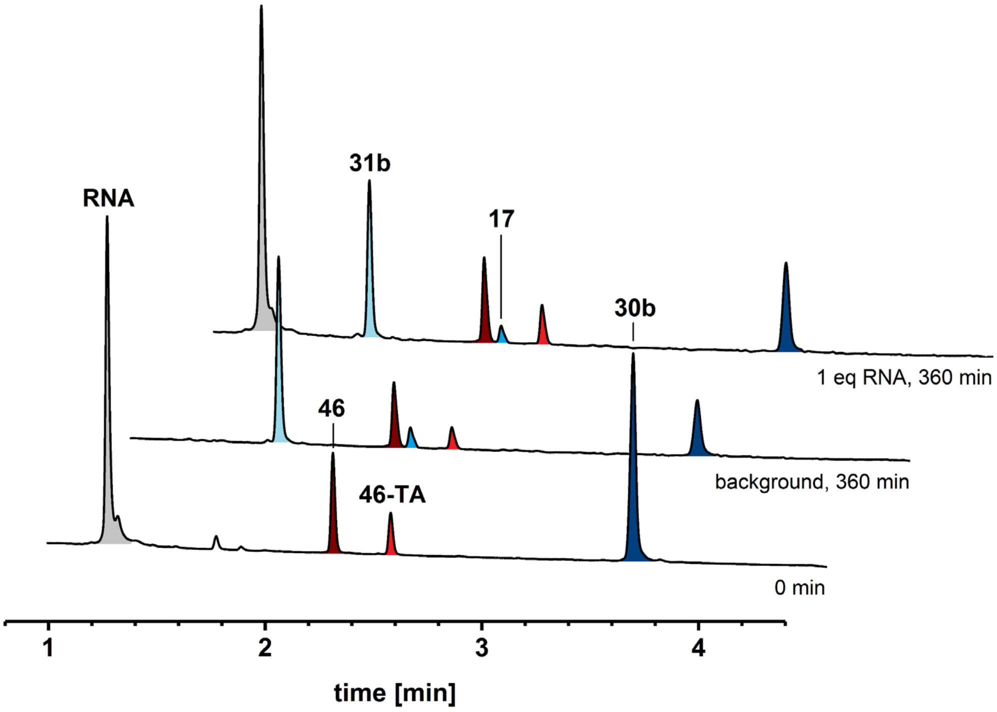

Figure S24: UPLC traces at 260 nm for the attempted transfer reaction between thiol-protected PNA acceptor conjugate **46** (A) and DNA donor conjugate **30b** (D) at t = 0 min (bottom) and after 360 min in the absence (middle) or presence (top) of 1 µM RNA template (5'-CACUGGAUUUAAGCAGAGUUCAAAAGCCCUUCAGCGGCC-3'). Only thioester hydrolysis, but no product formation was observed (confirmed by UPLC-MS). The thiazine compound **46-TA** was present due to partial cyclization of the thiol-protected PNA acceptor conjugate **46** during HPLC purification as described earlier (page 36f.) Compound **17** corresponds to the hydrolyzed donor fragment (page 16). *Conditions:* 10 mM MOPS, 200 mM NaCl, 2 mM TCEP, pH 7.2, [A] = 1 µM, [D] = 2 µM, [RNA] = 1 µM, 37 °C. *Gradient:* 10 to 40% B2 in 4 min, column 2 (UPLC-UV/Vis).

Control experiment using a thiol-free PNA acceptor conjugate

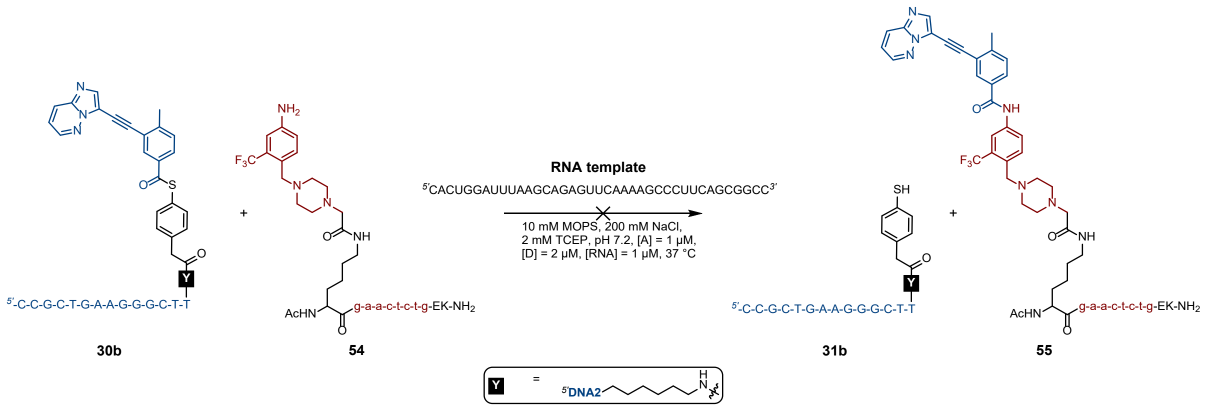

Scheme S9: Attempted transfer reaction between thiol-free PNA acceptor conjugate **54** (A) and DNA donor conjugate **30b** (D).

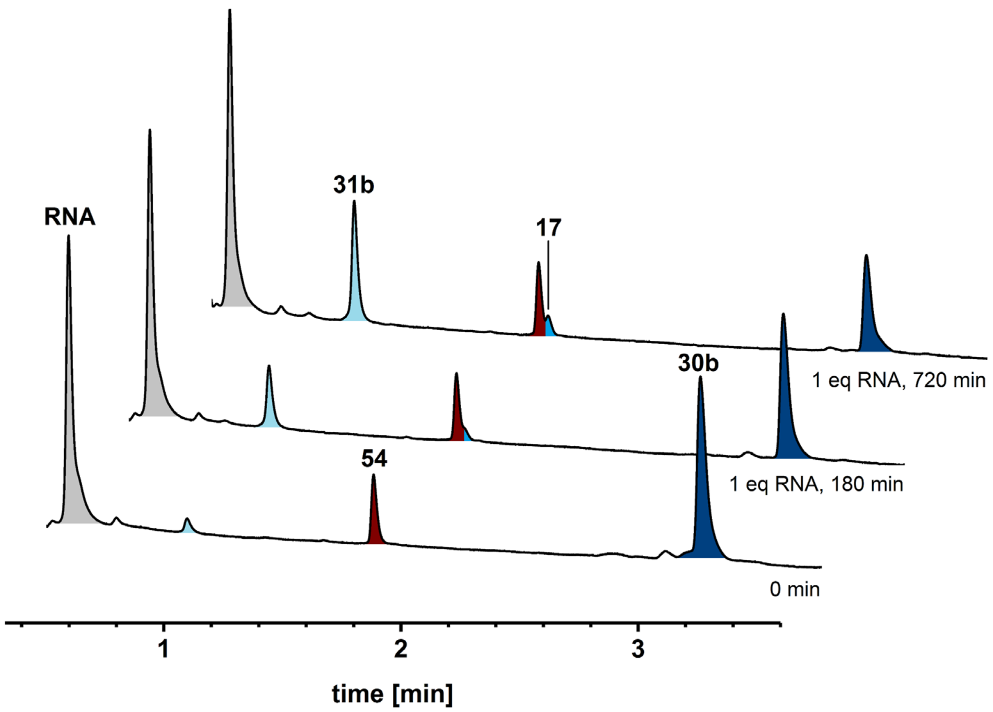

Figure S25: UPLC traces at 260 nm for the attempted transfer reaction between thiol-free PNA acceptor conjugate **54** (A) and DNA donor conjugate **30b** (D) at t = 0 min (bottom), after 180 min (middle) and after 720 min (top) in the presence of 1 µM RNA template (5'-CACUGGAUUUAAGCAGAGUUCAAAAGCCCUUCAGCGGCC-3'). Only thioester hydrolysis, but no product formation was observed (confirmed by UPLC-MS). *Conditions:* 10 mM MOPS, 200 mM NaCl, 2 mM TCEP, pH 7.2, [A] = 1 µM, [D] = 2 µM, [RNA] = 1 µM, 37 °C. *Gradient:* 10 to 40% B2 in 4 min, column 2 (UPLC-UV/Vis).

NMR spectra

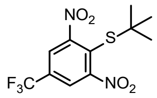

2-(*tert*-Butylthio)-1,3-dinitro-5-(trifluoromethyl)benzene (34)

^{1}H NMR (500 MHz, $CDCl_3$):

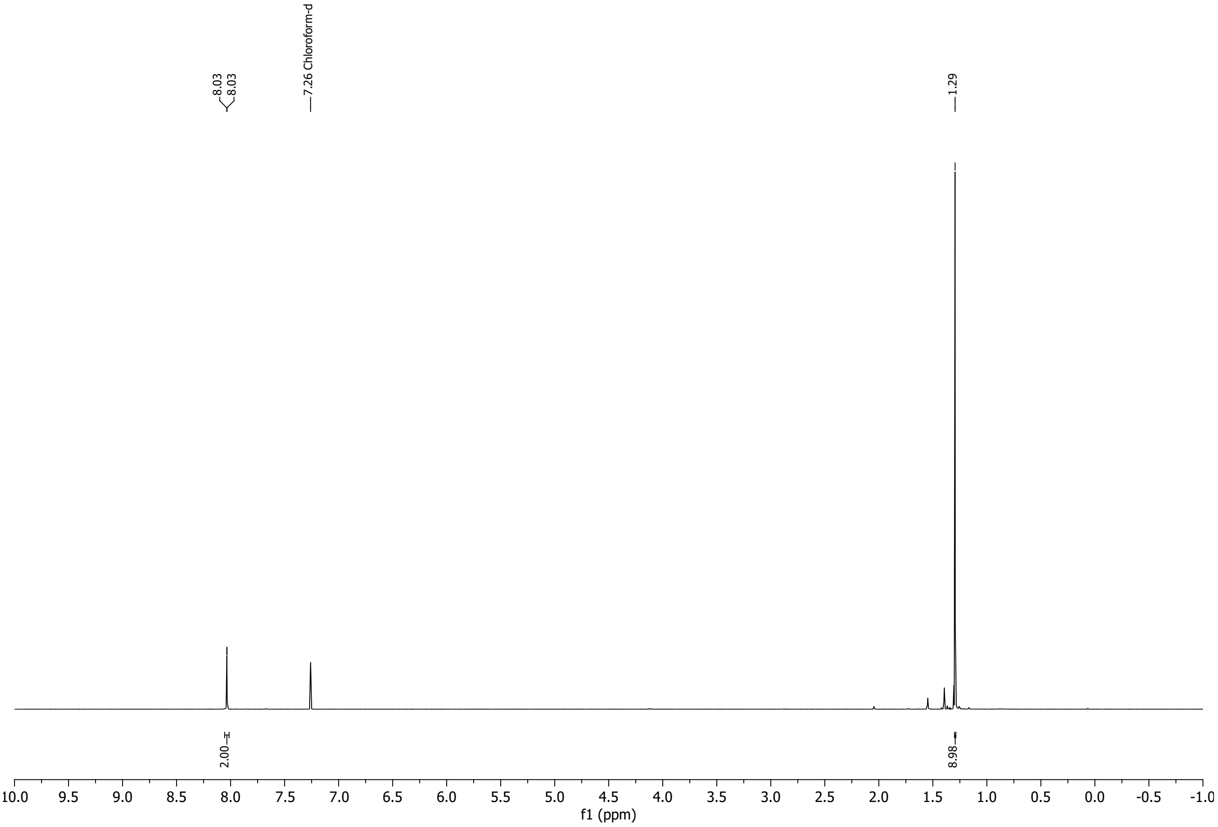

^{13}C NMR (126 MHz, $CDCl_3$):

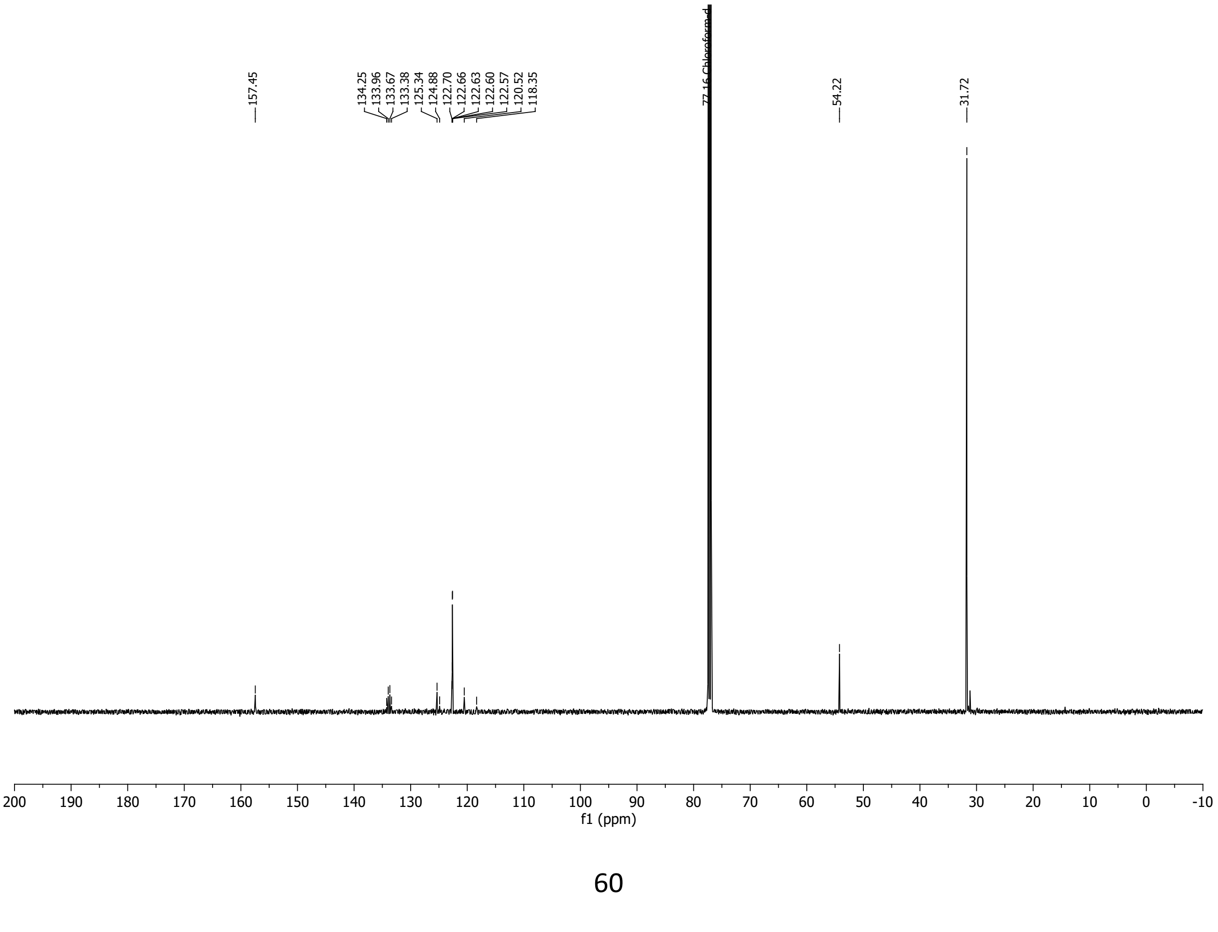

1-(2-(*tert*-Butylthio)-3-nitro-5-(trifluoromethyl)phenyl)-4-methyl-1*H*-imidazole (35)

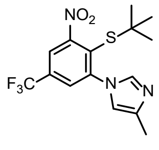

^{1}H NMR (500 MHz, $CDCl_3$):

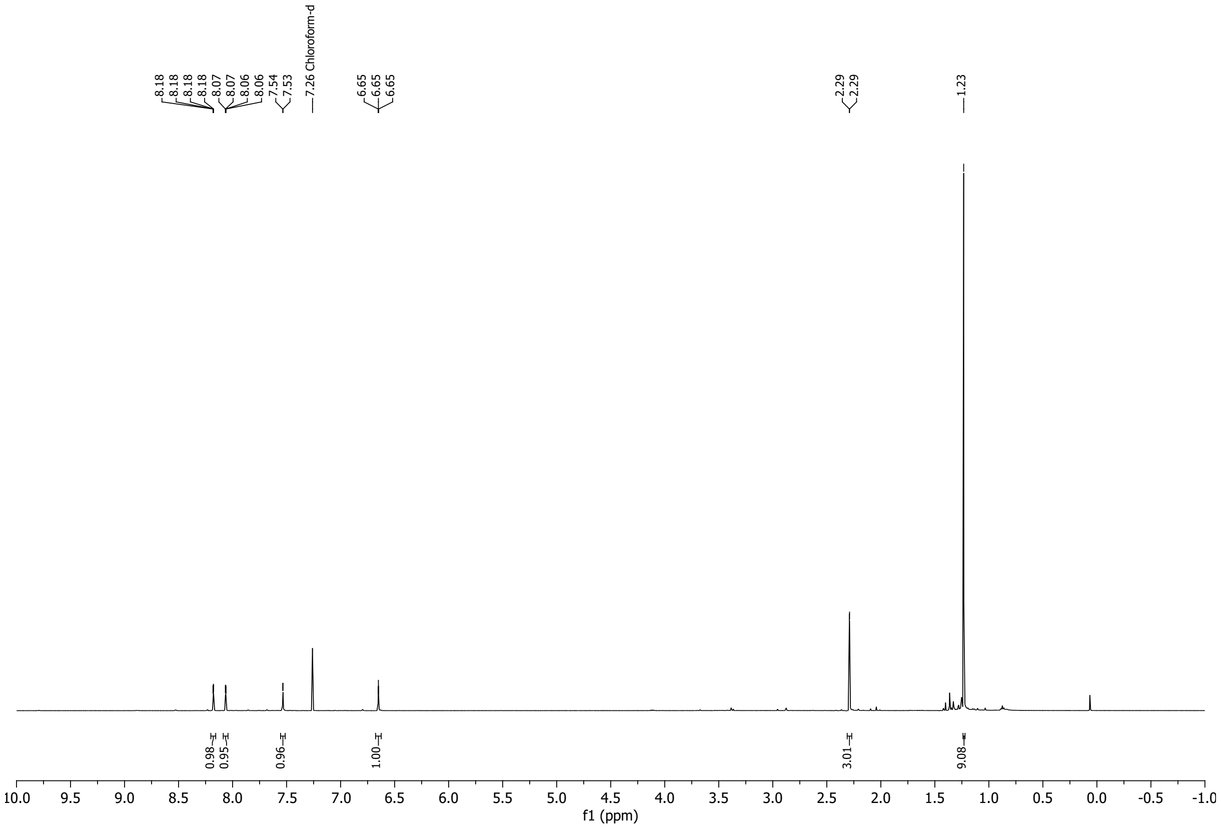

^{13}C NMR (126 MHz, $CDCl_3$):

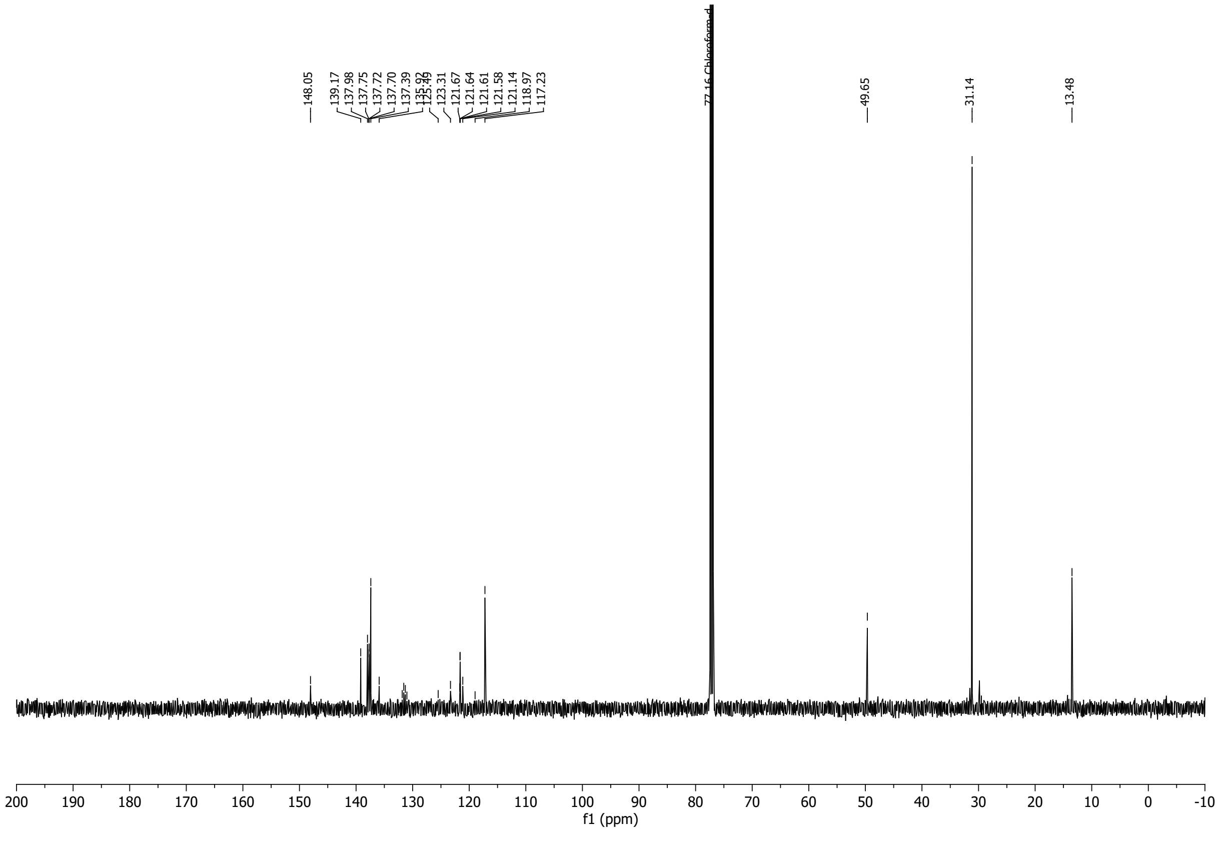

1-(3-Amino-2-(*tert*-butylthio)-5-(trifluoromethyl)phenyl)-4-methyl-1*H*-imidazole (5)

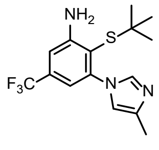

^{1}H NMR (500 MHz, $CDCl_3$):

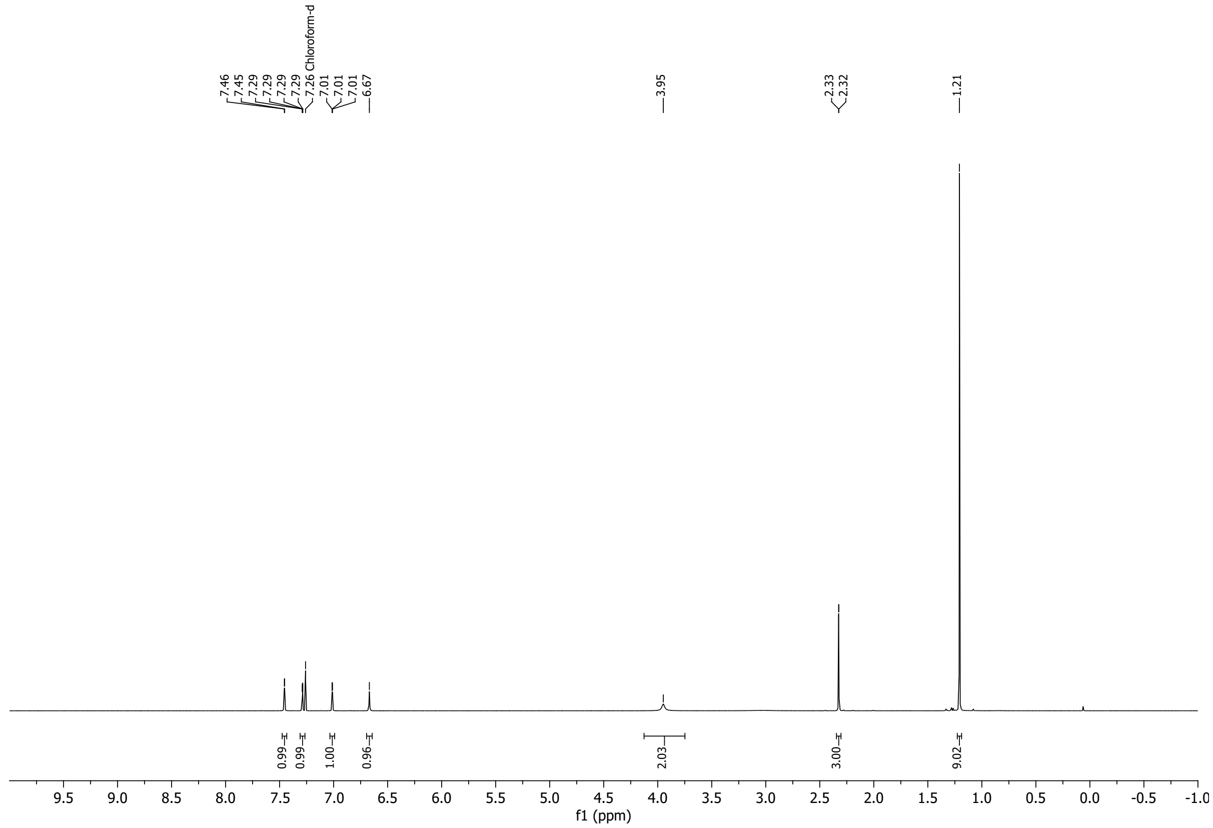

^{13}C NMR (126 MHz, $CDCl_3$):

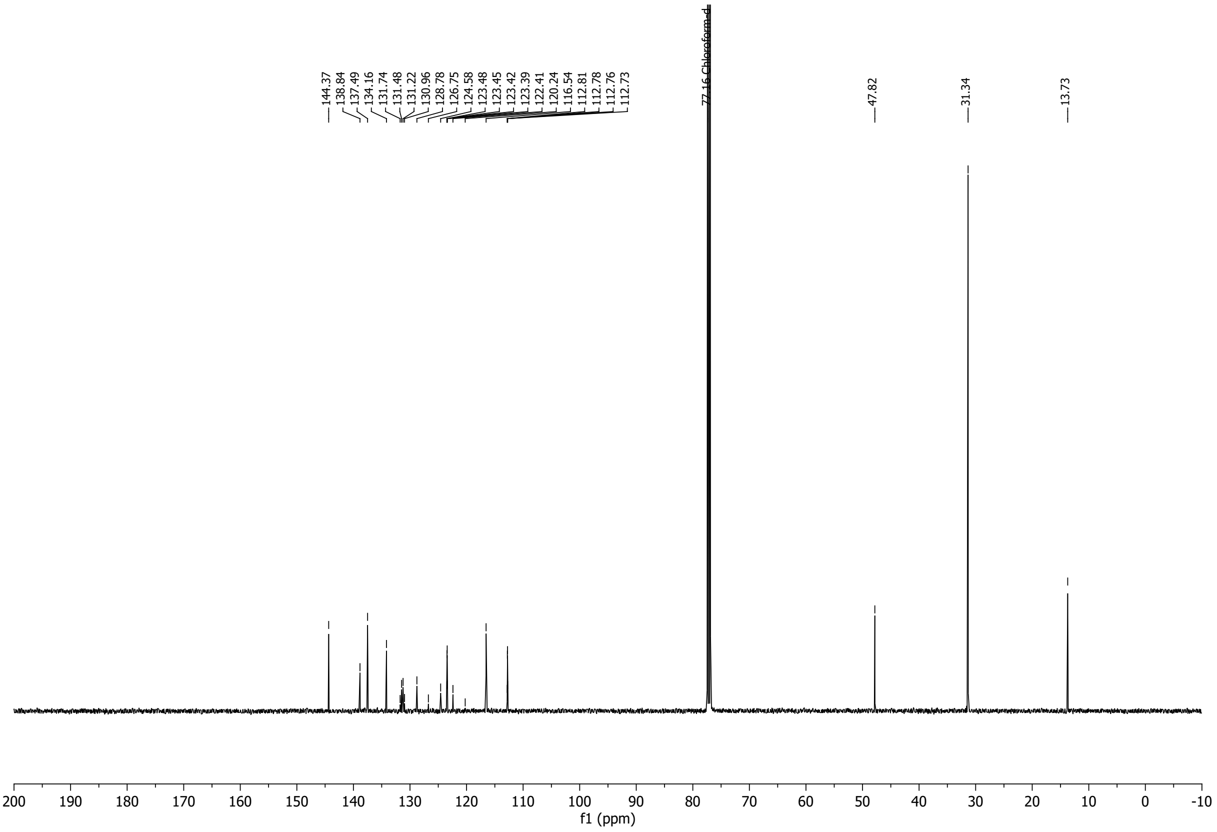

4-(Pyridin-3-yl)pyrimidin-2-amine (37)

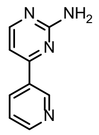

^{1}H NMR (500 MHz, DMSO-d_6):

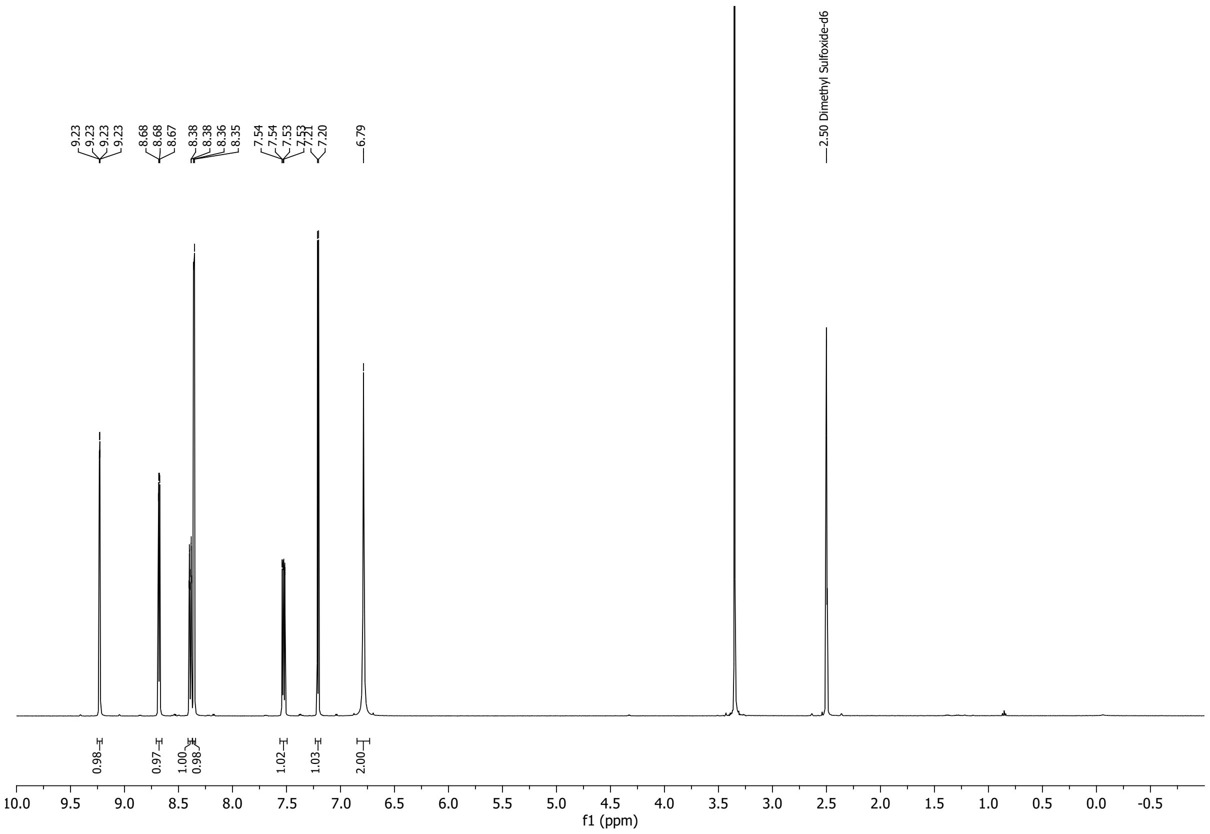

^{13}C NMR (126 MHz, DMSO-d_6):

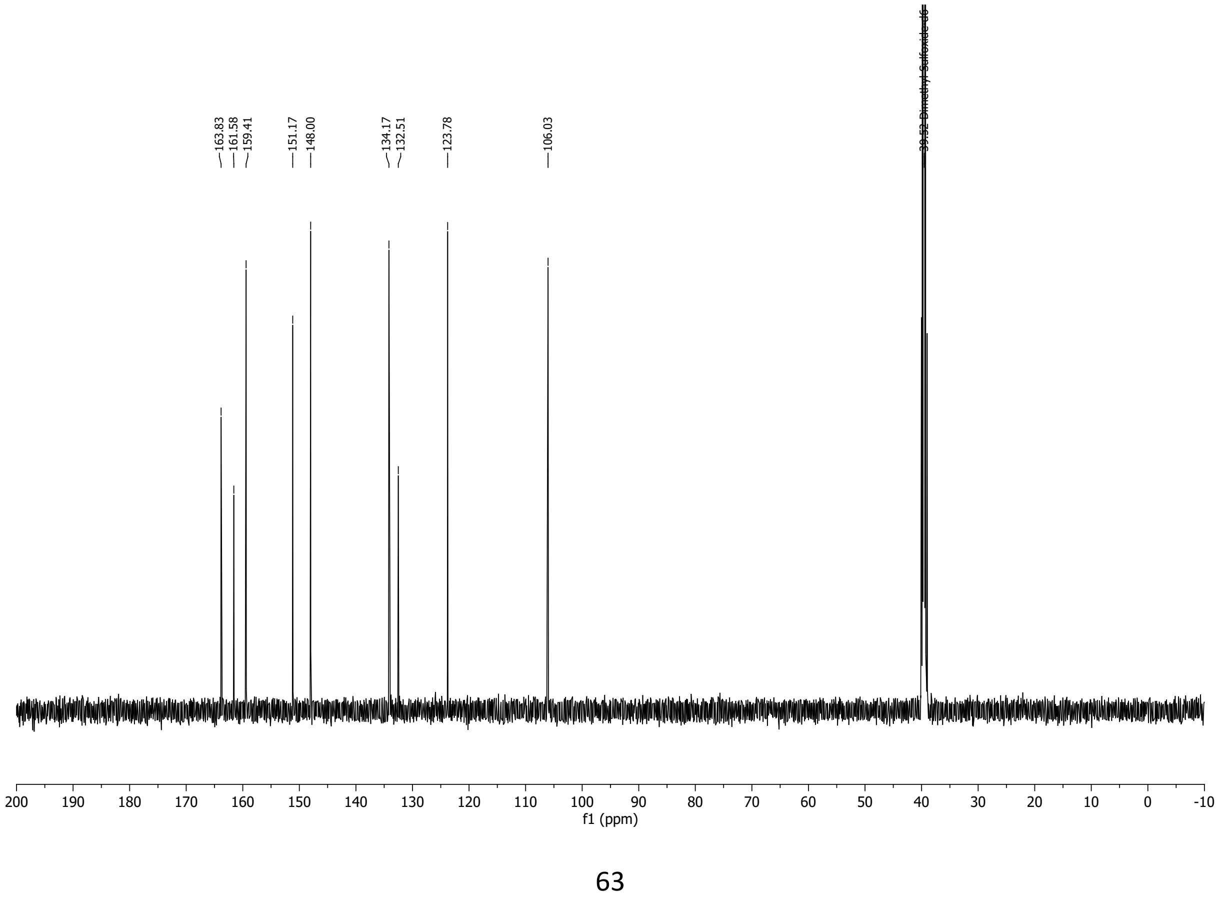

Methyl 4-methyl-3-((4-(pyridin-3-yl)pyrimidin-2-yl)amino)benzoate (4)

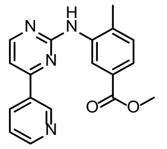

^{1}H NMR (500 MHz, DMSO-d_6):

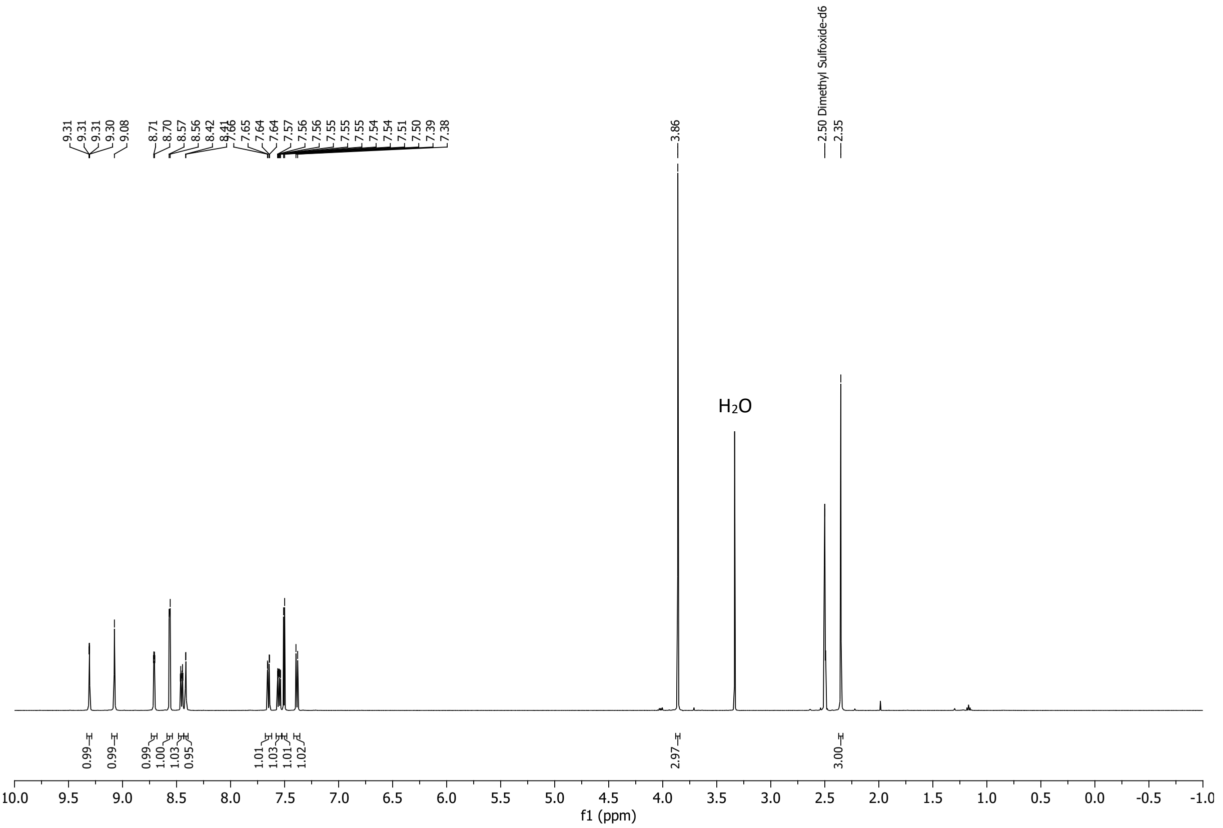

^{13}C NMR (126 MHz, DMSO-d_6):

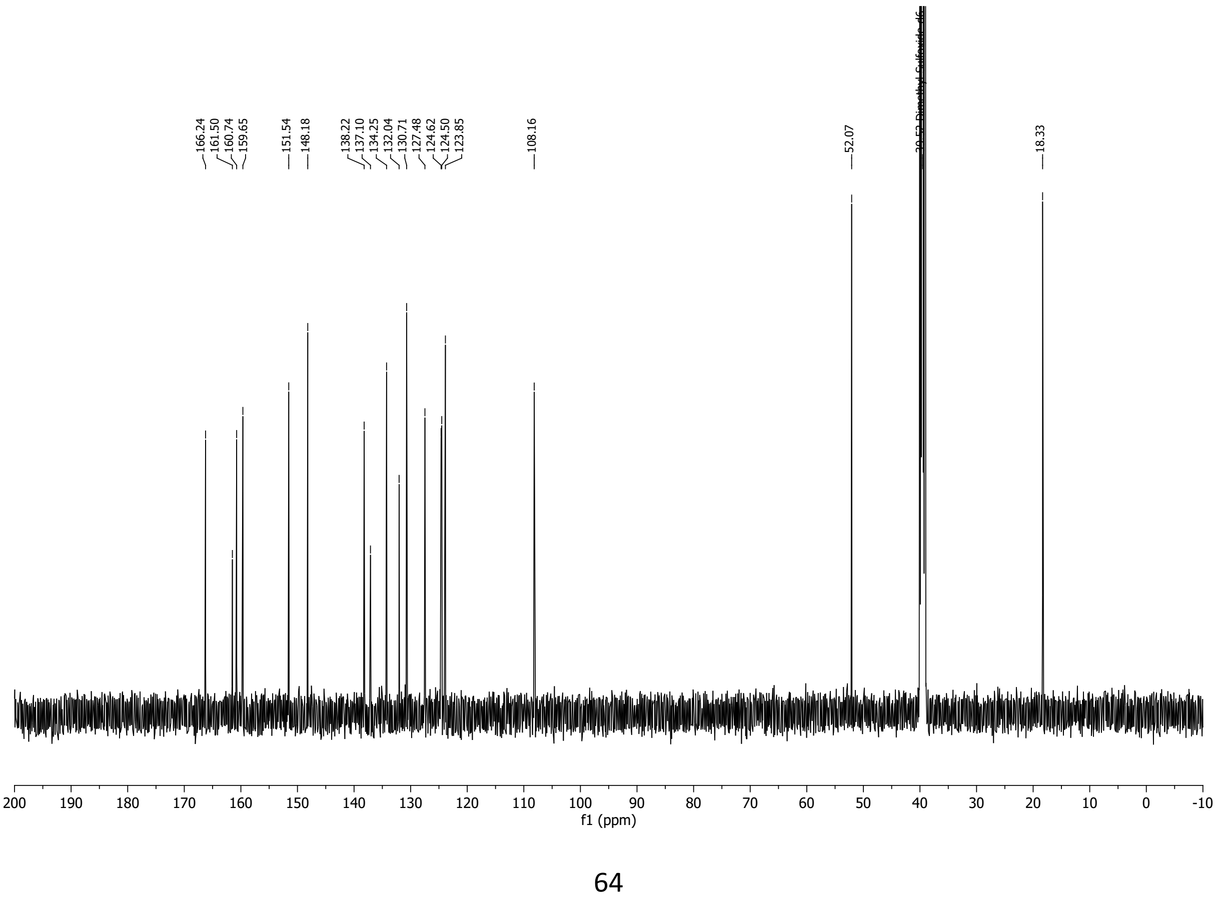

Nilotinib-S(*t*Bu) (6)

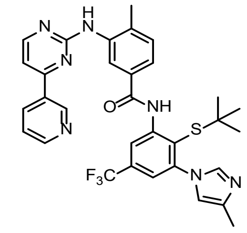

^{1}H NMR (500 MHz, DMSO-d_6):

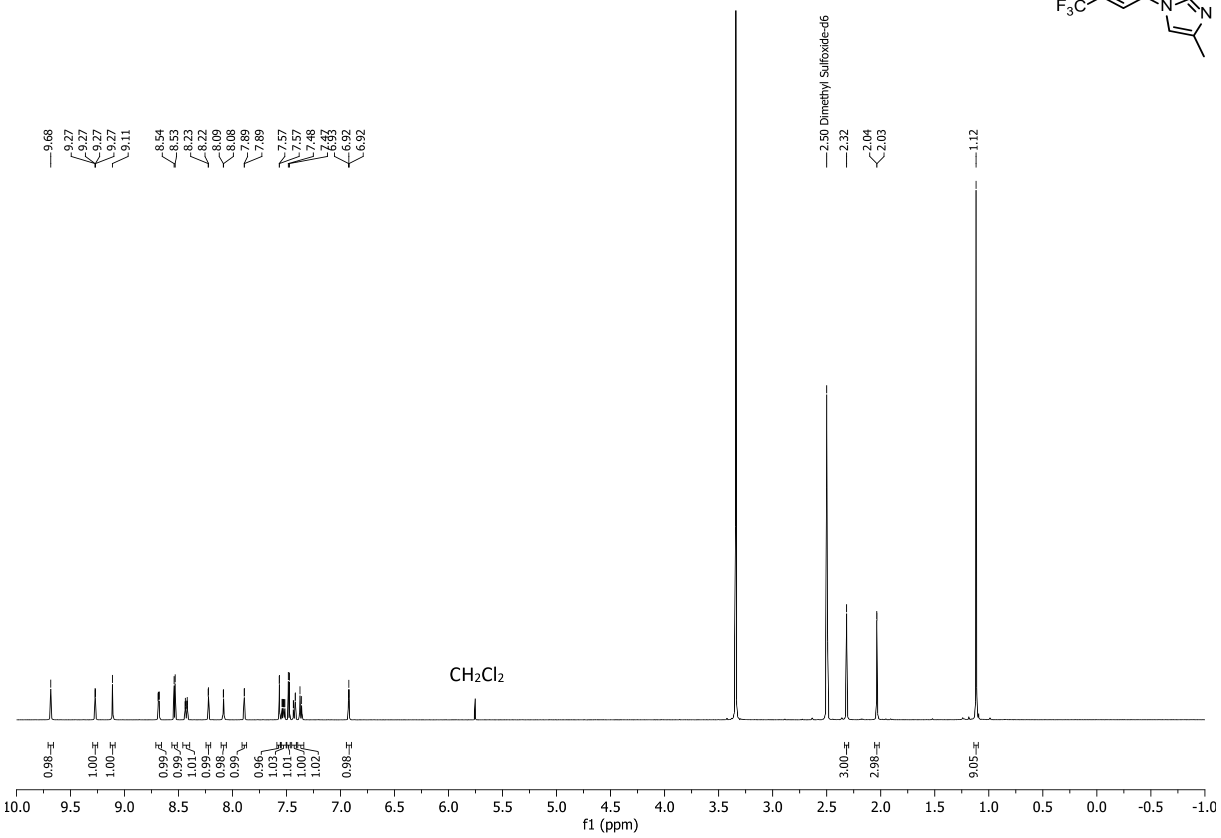

^{13}C NMR (126 MHz, DMSO-d_6):

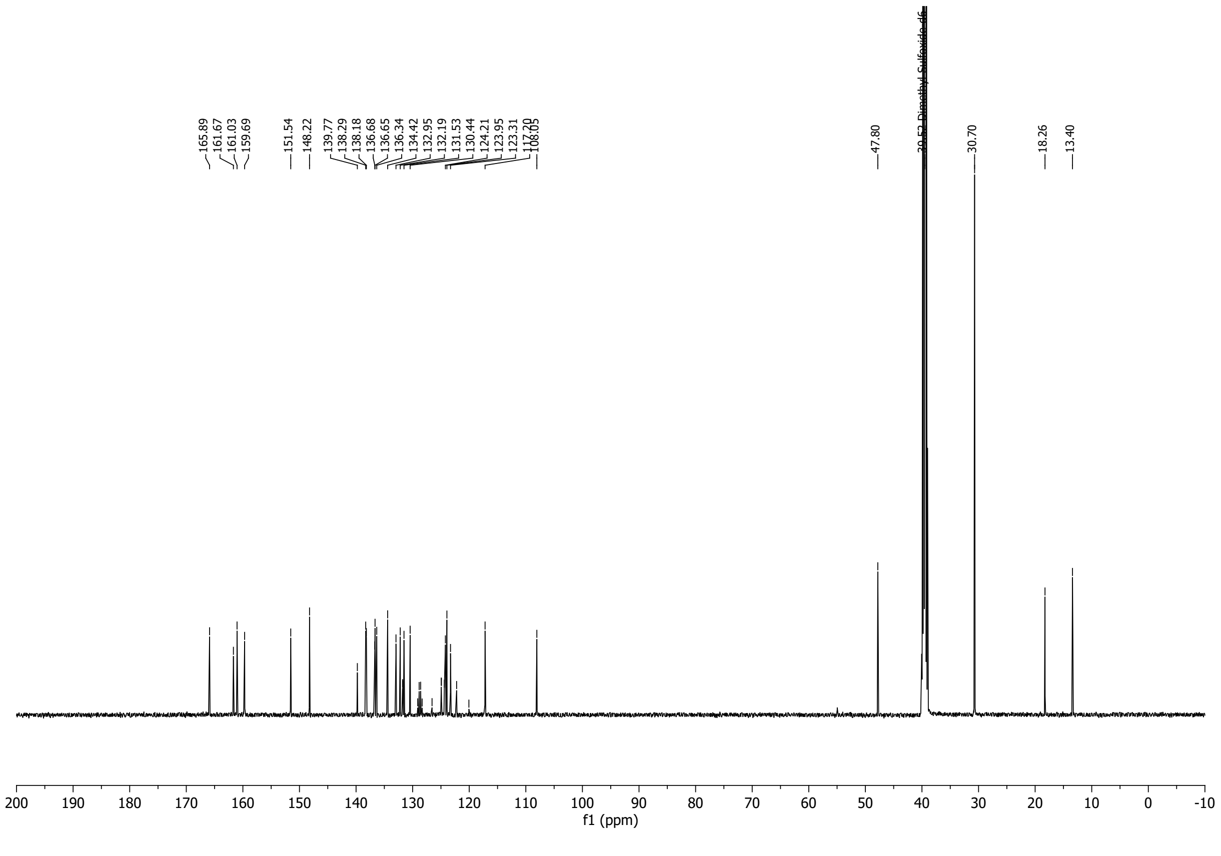

Nilotinib-SH (1a)

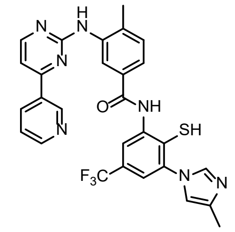

^{1}H NMR (500 MHz, DMSO-d_6):

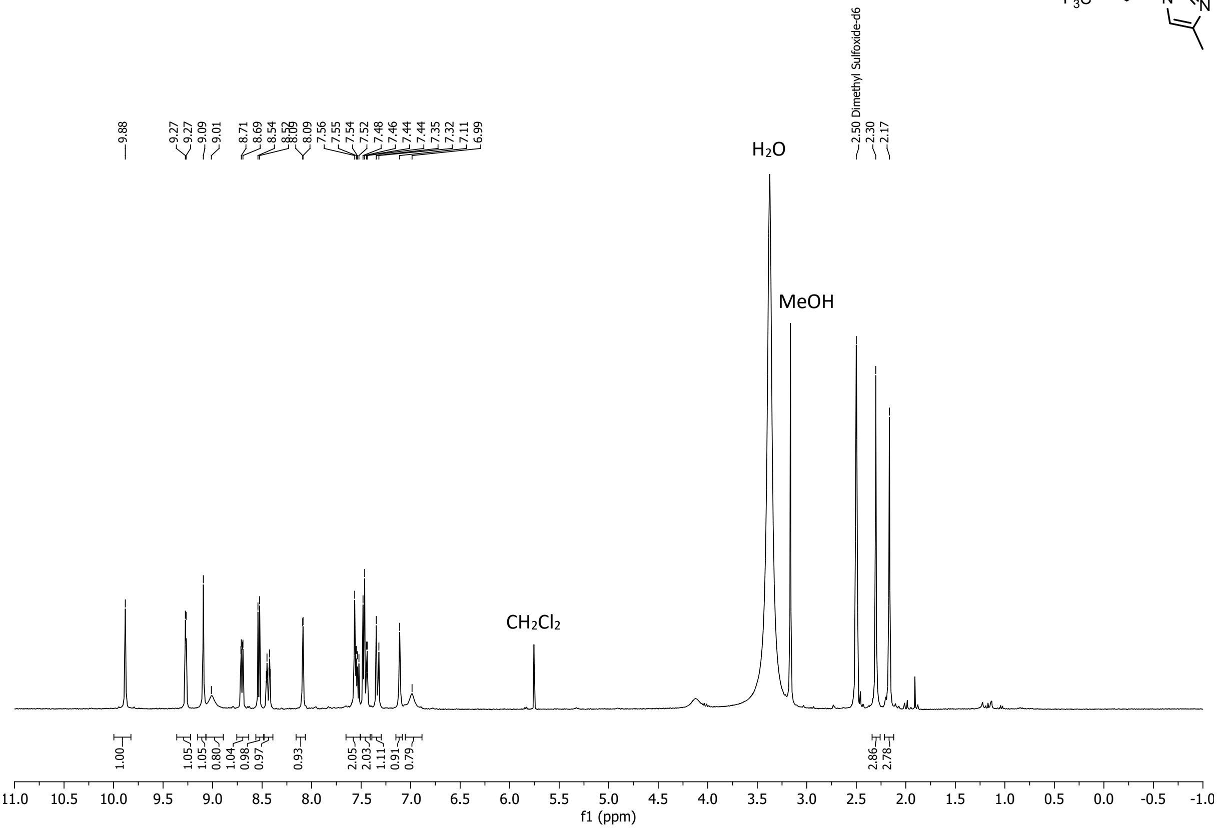

1-(Bromomethyl)-5-chloro-4-nitro-2-(trifluoromethyl)benzene (8)

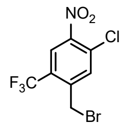

^{1}H NMR (500 MHz, $CDCl_3$):

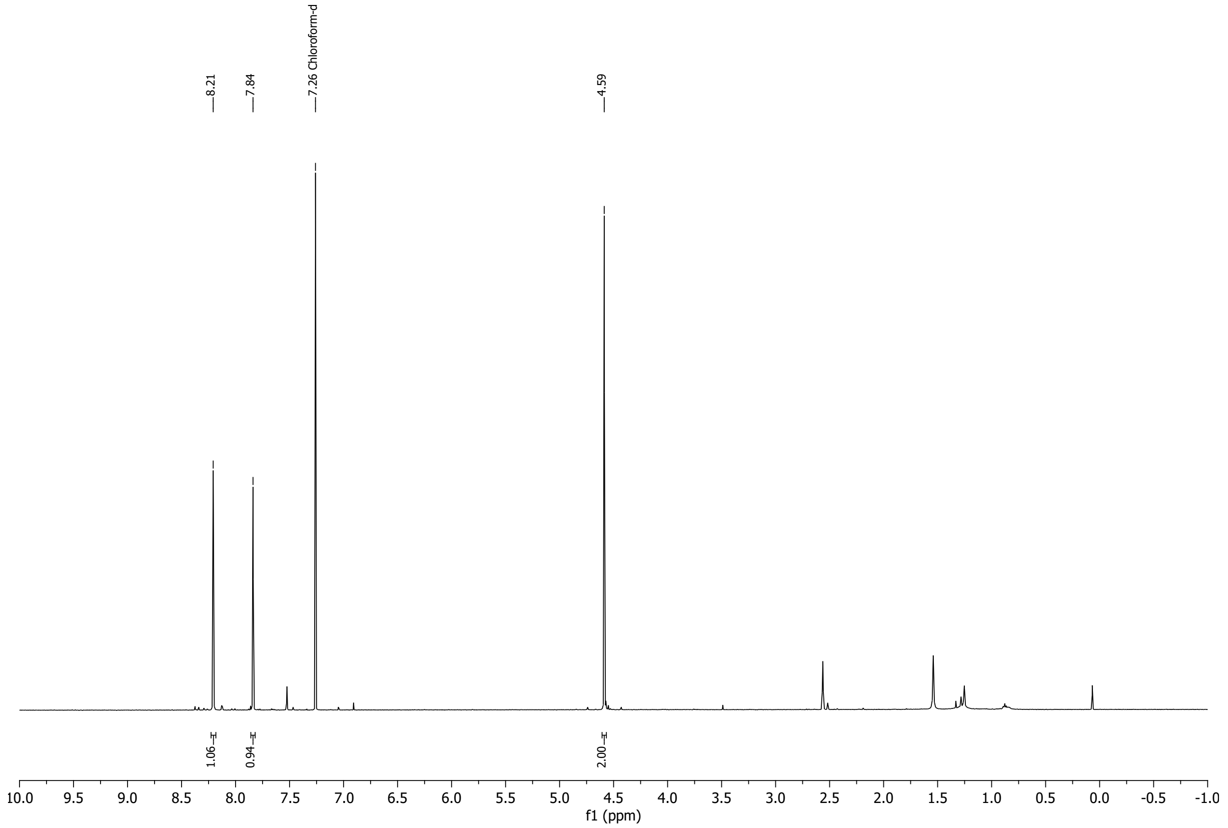

^{13}C NMR (126 MHz, $CDCl_3$):

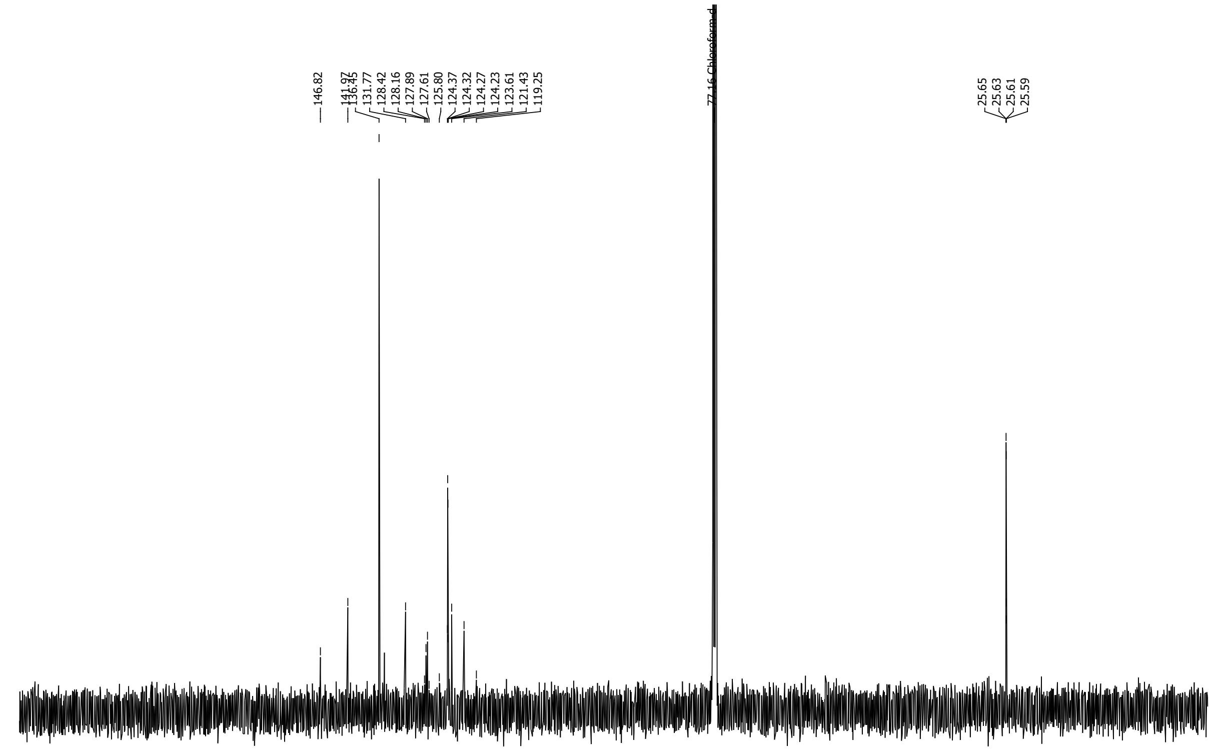

1-(5-Chloro-4-nitro-2-(trifluoromethyl)benzyl)-4-methylpiperazine (9)

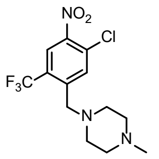

^{1}H NMR (500 MHz, $CDCl_3$):

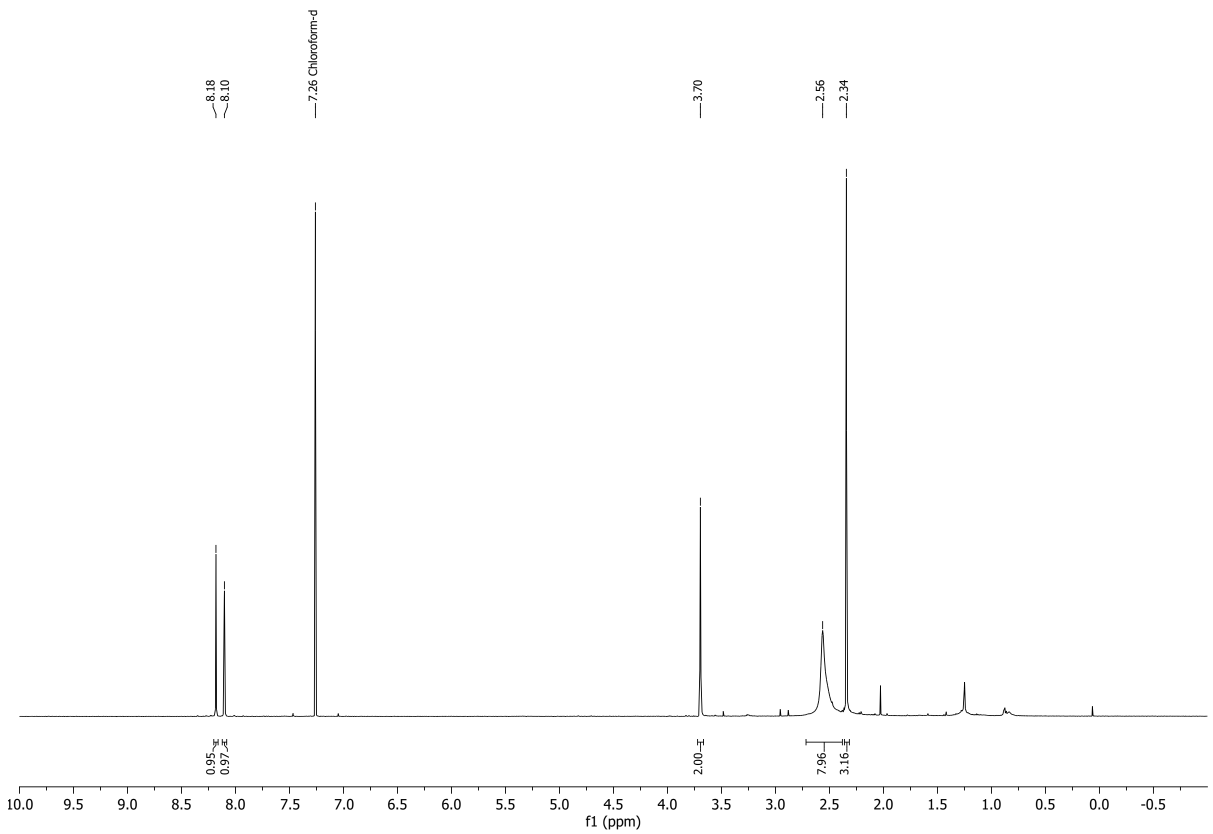

^{13}C NMR (126 MHz, $CDCl_3$):

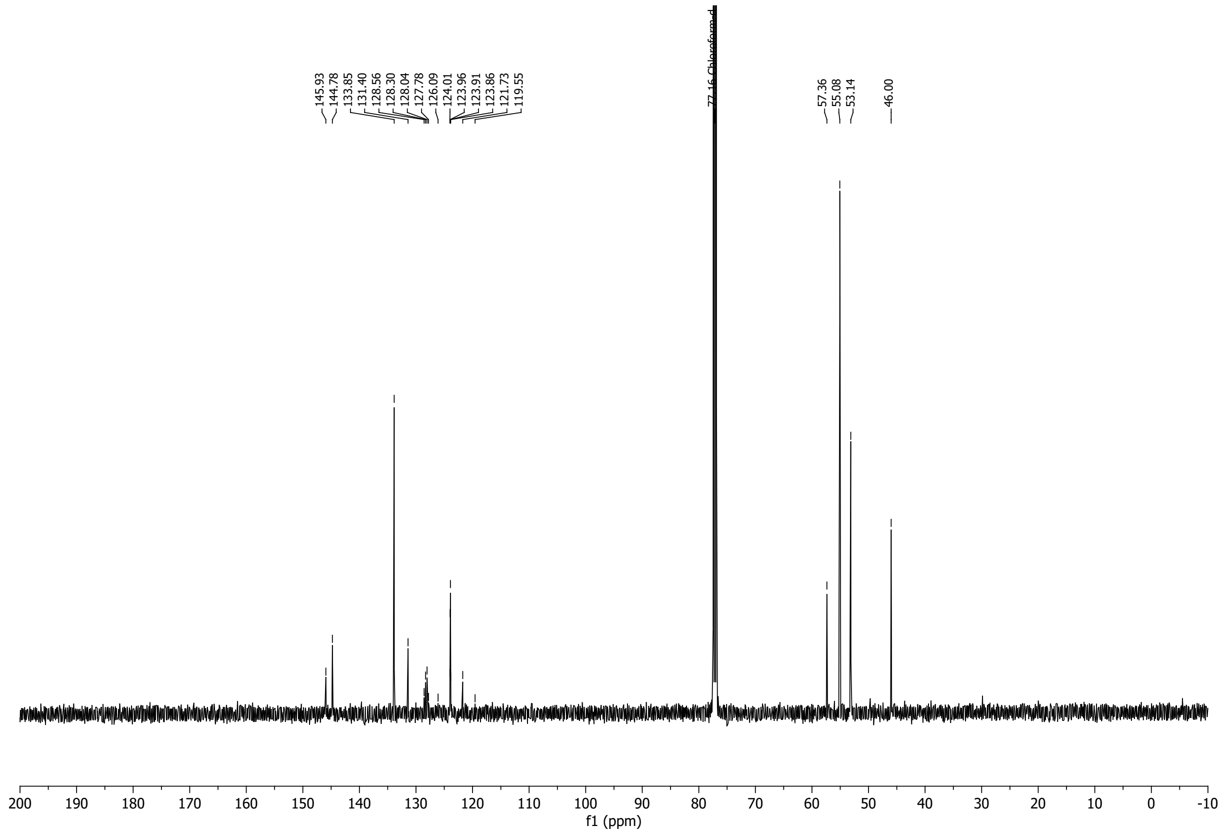

1-(5-(*tert*-Butylthio)-4-nitro-2-(trifluoromethyl)benzyl)-4-methylpiperazine (10)

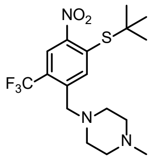

^{1}H NMR (500 MHz, $CDCl_3$):

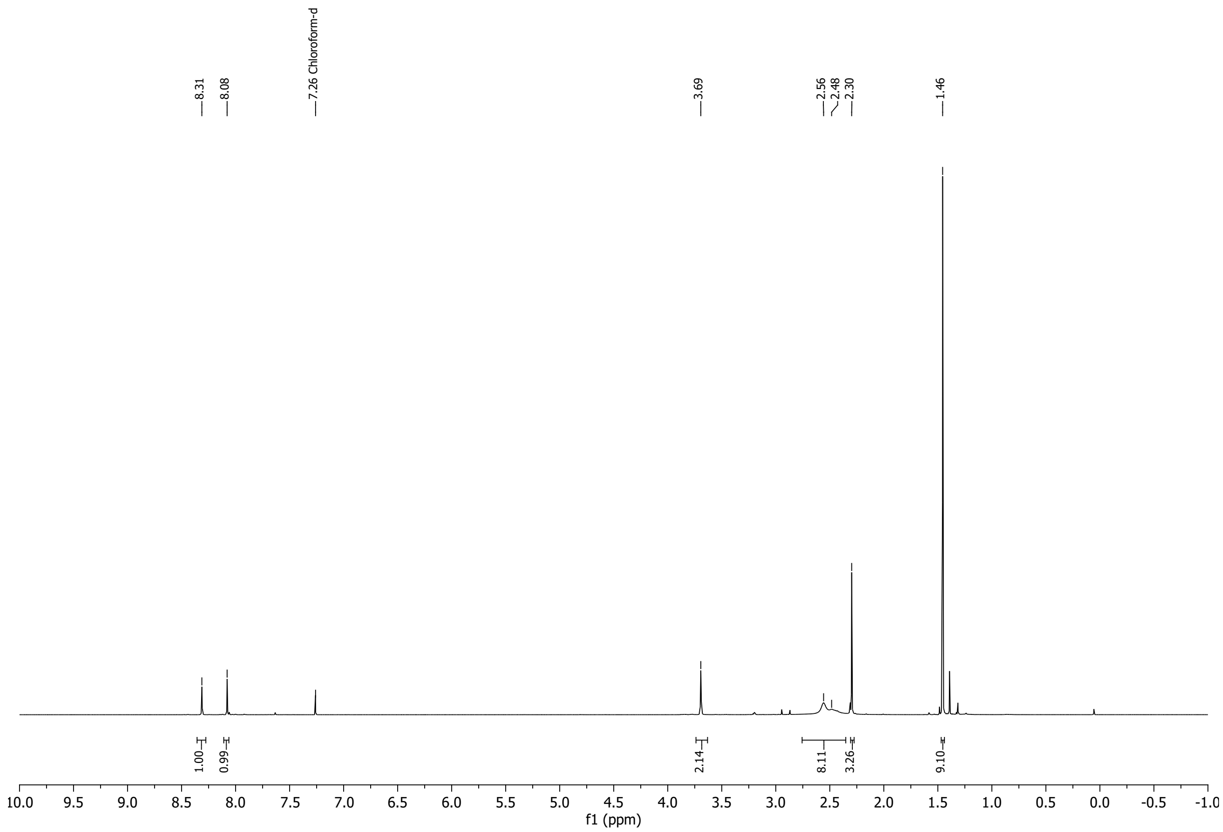

^{13}C NMR (126 MHz, $CDCl_3$):

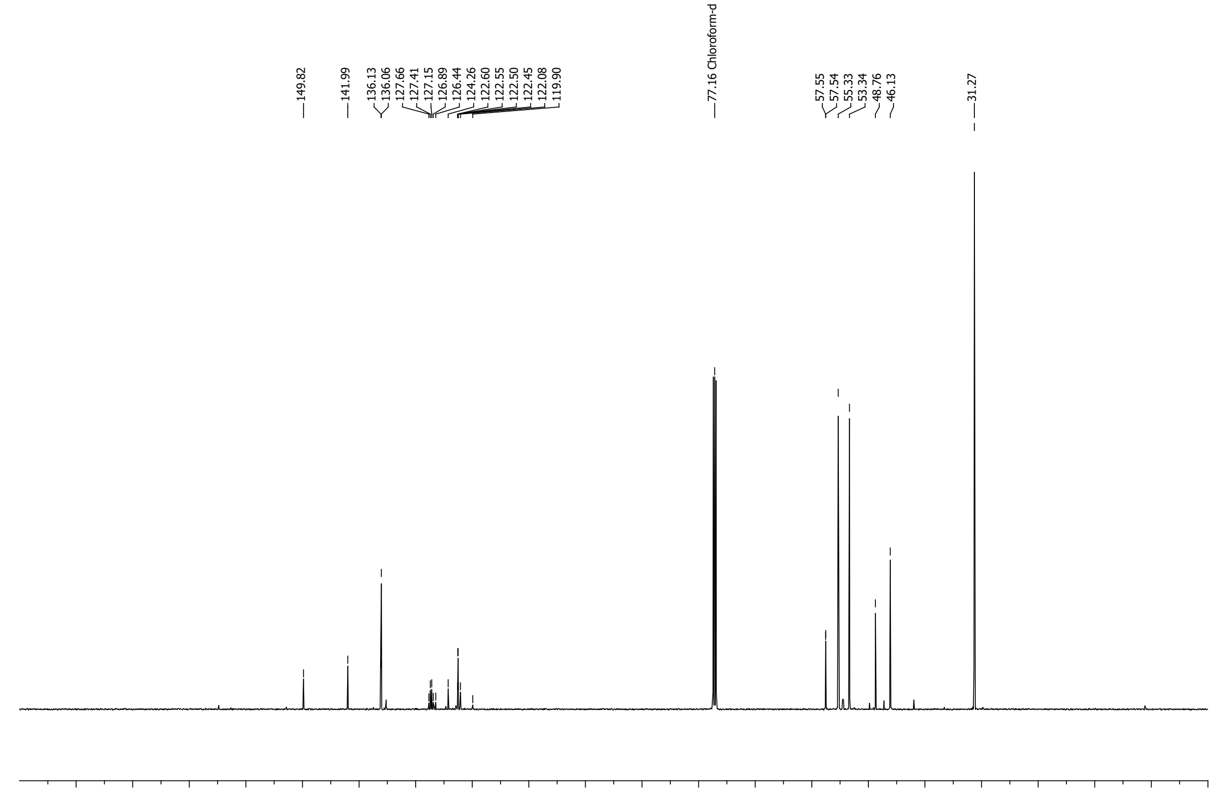

1-(4-Amino-5-(*tert*-butylthio)-2-(trifluoromethyl)benzyl)-4-methylpiperazine (11)

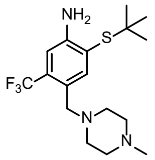

^{1}H NMR (500 MHz, $CDCl_3$):

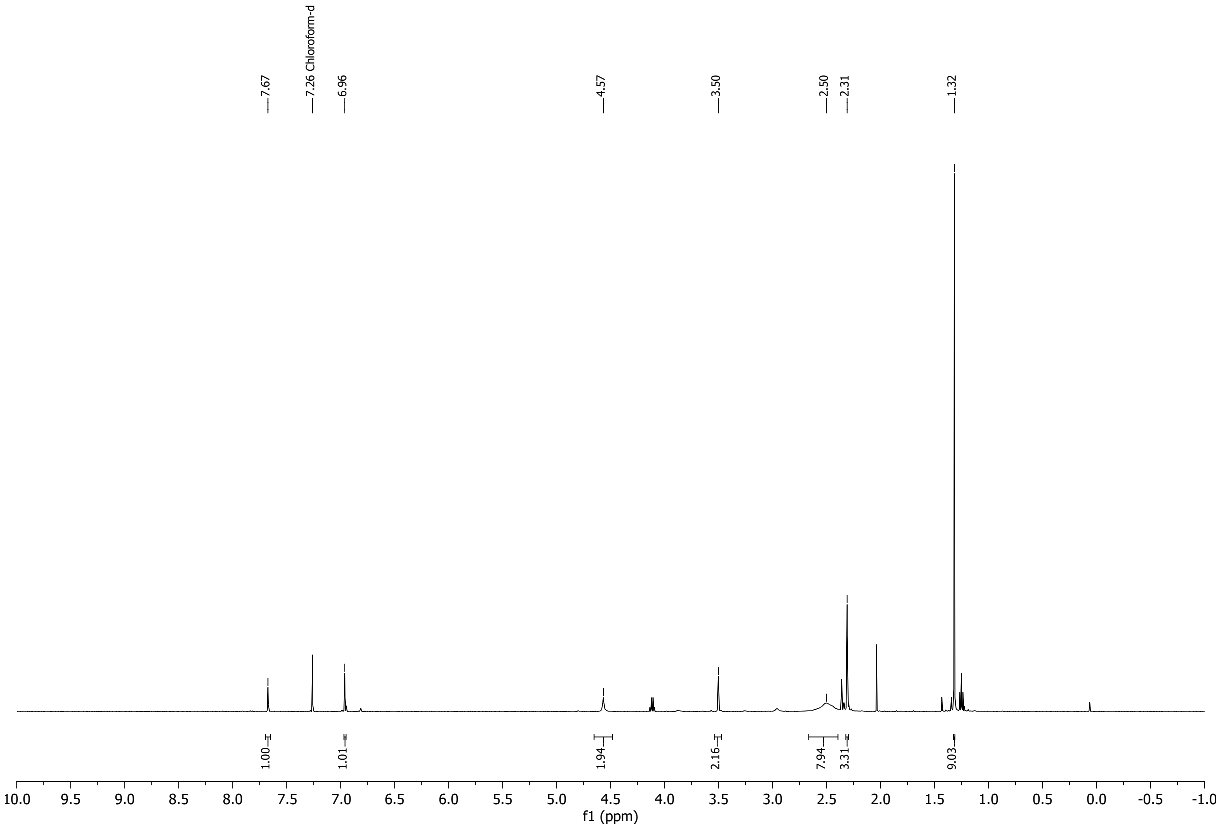

^{13}C NMR (126 MHz, $CDCl_3$):

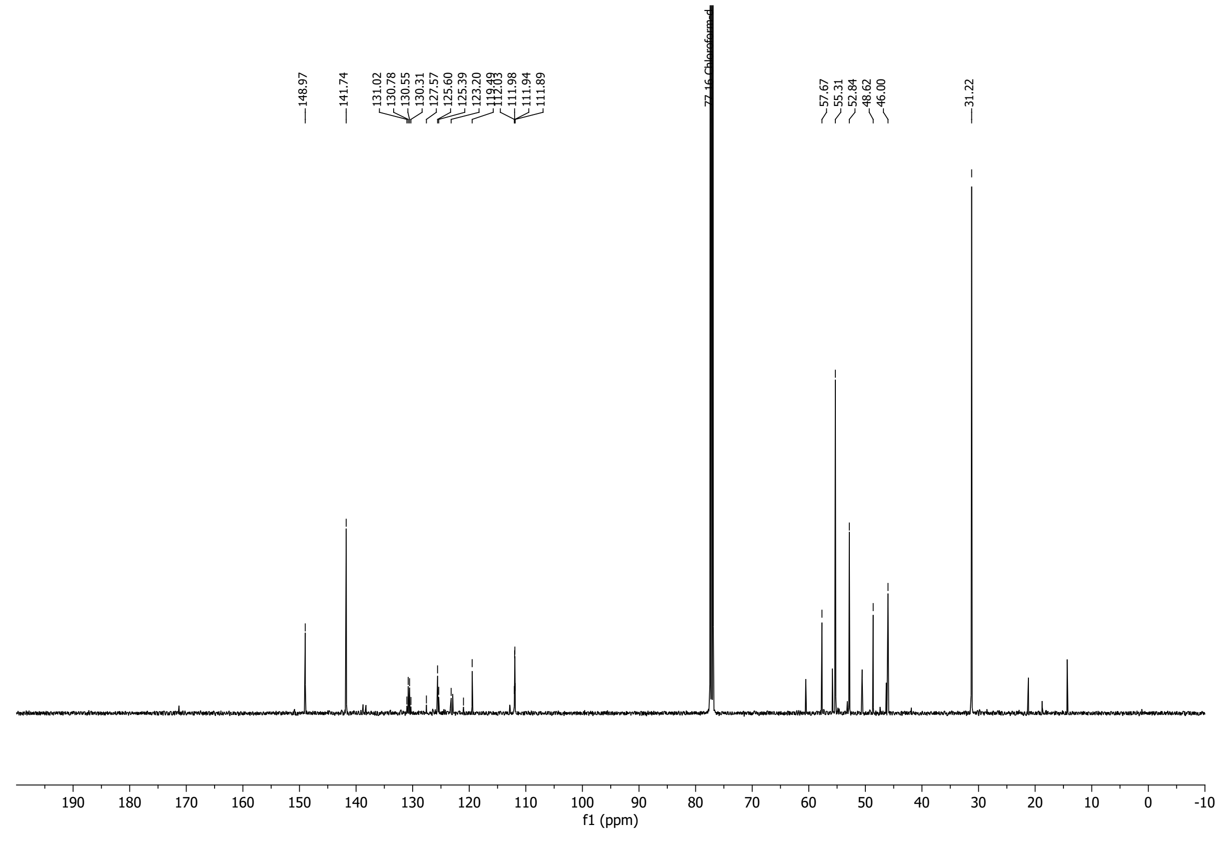

2-Amino-5-((4-methylpiperazin-1-yl)methyl)-4-(trifluoromethyl)benzenethiol (12)

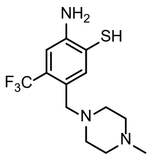

^{1}H NMR (500 MHz, DMSO-d_6):

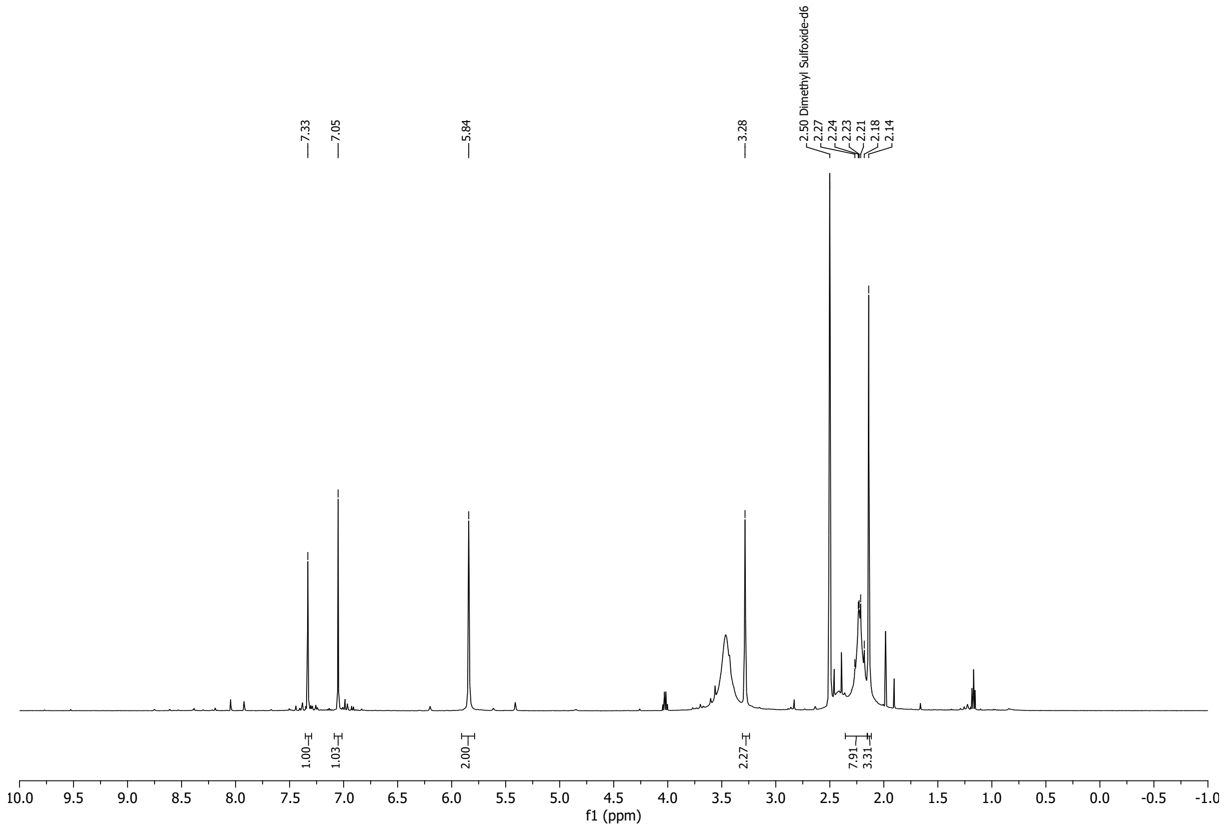

^{13}C NMR (126 MHz, DMSO-d_6):

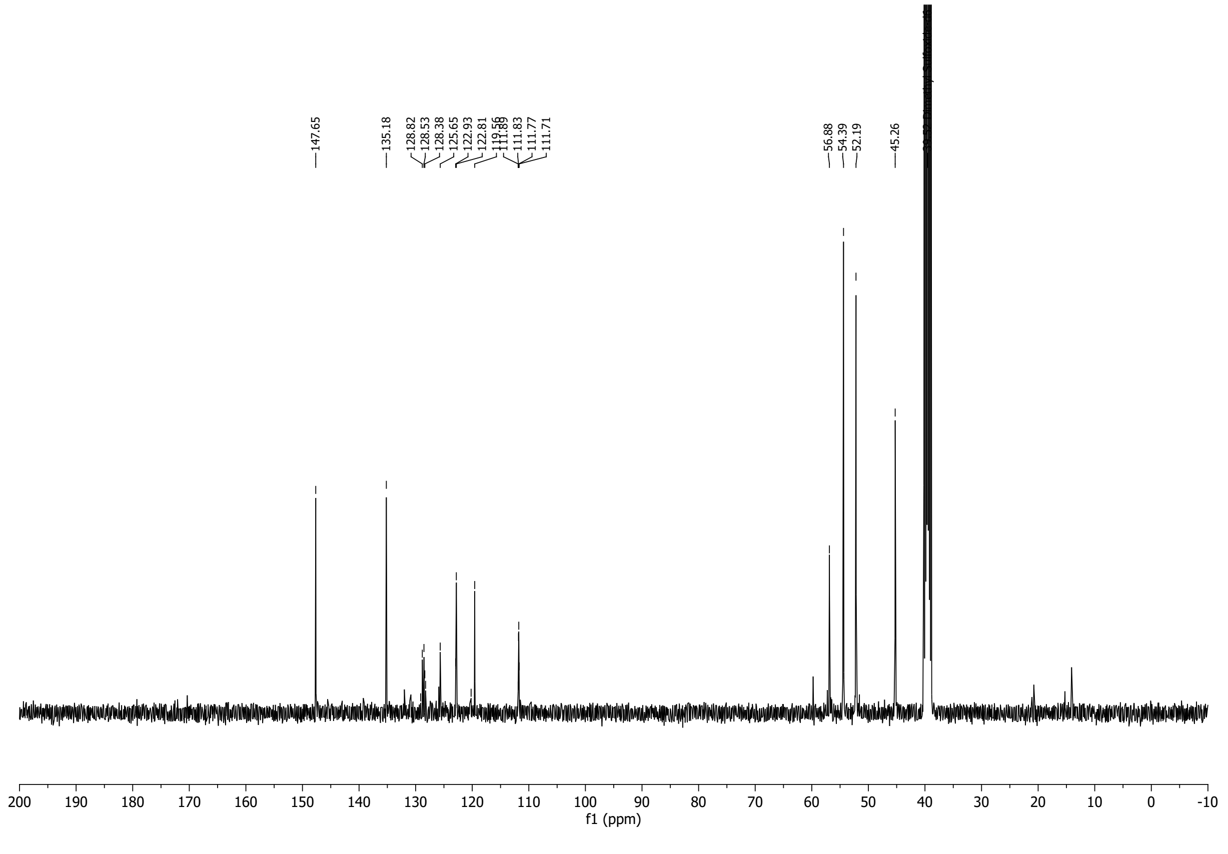

Methyl 3-ethynyl-4-methylbenzoate (14)

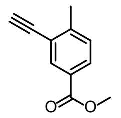

^{1}H NMR (500 MHz, $CDCl_3$):

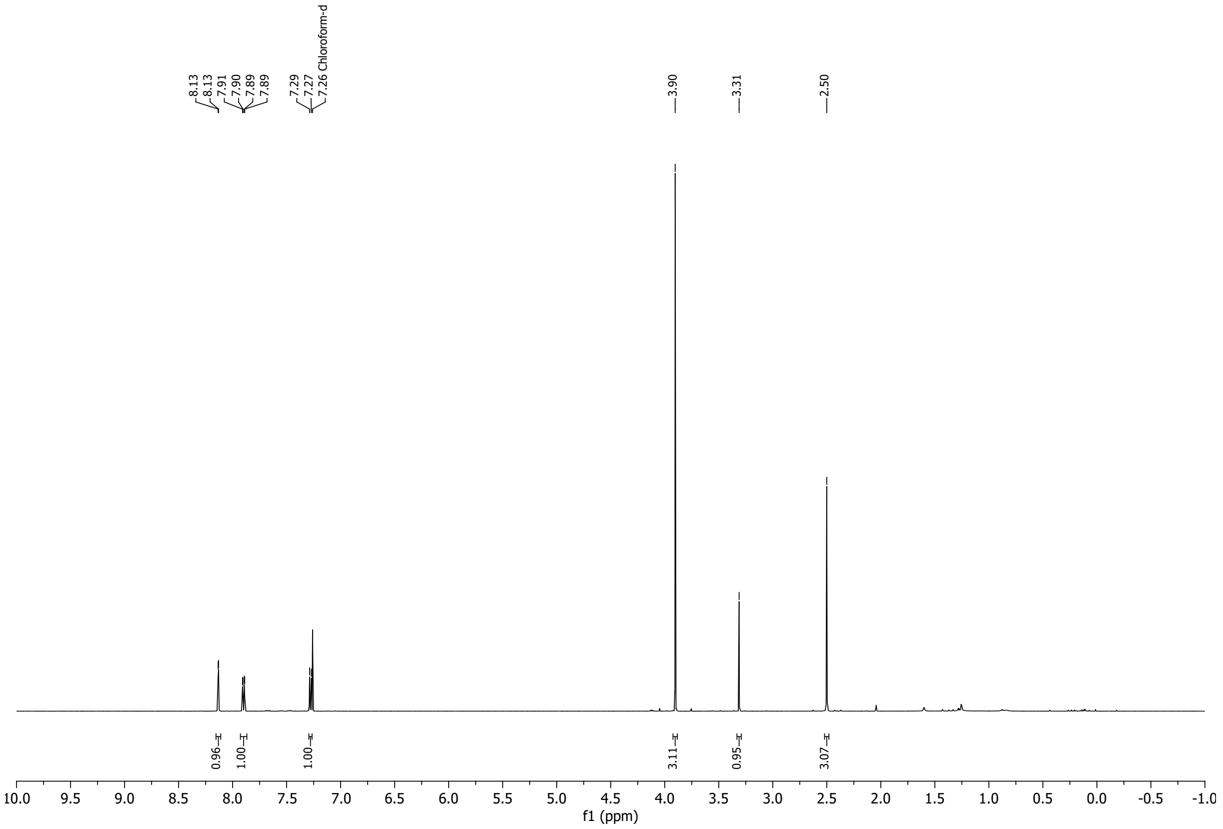

^{13}C NMR (126 MHz, $CDCl_3$):

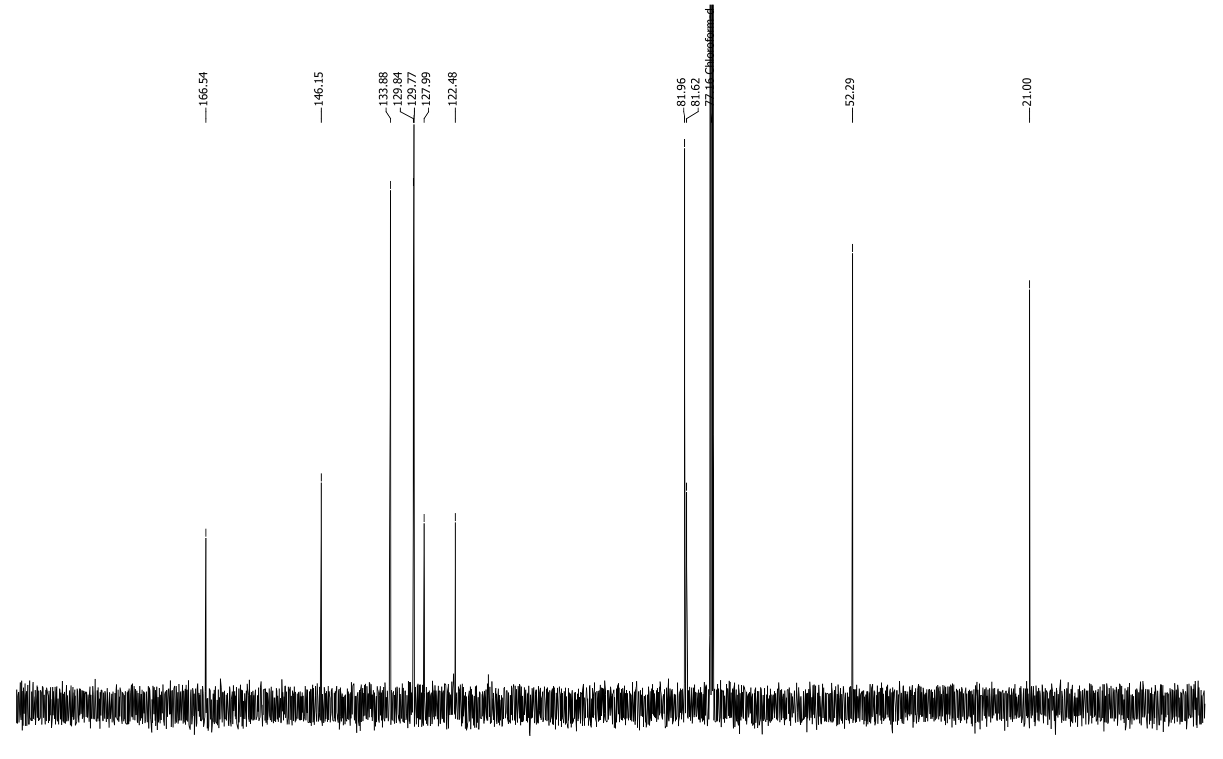

Methyl 3-((imidazo[1,2-*b*]pyridazin-3-yl)ethynyl)-4-methylbenzoate (15)

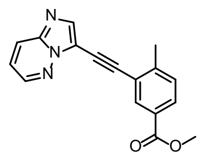

^{1}H NMR (400 MHz, DMSO-d_6):

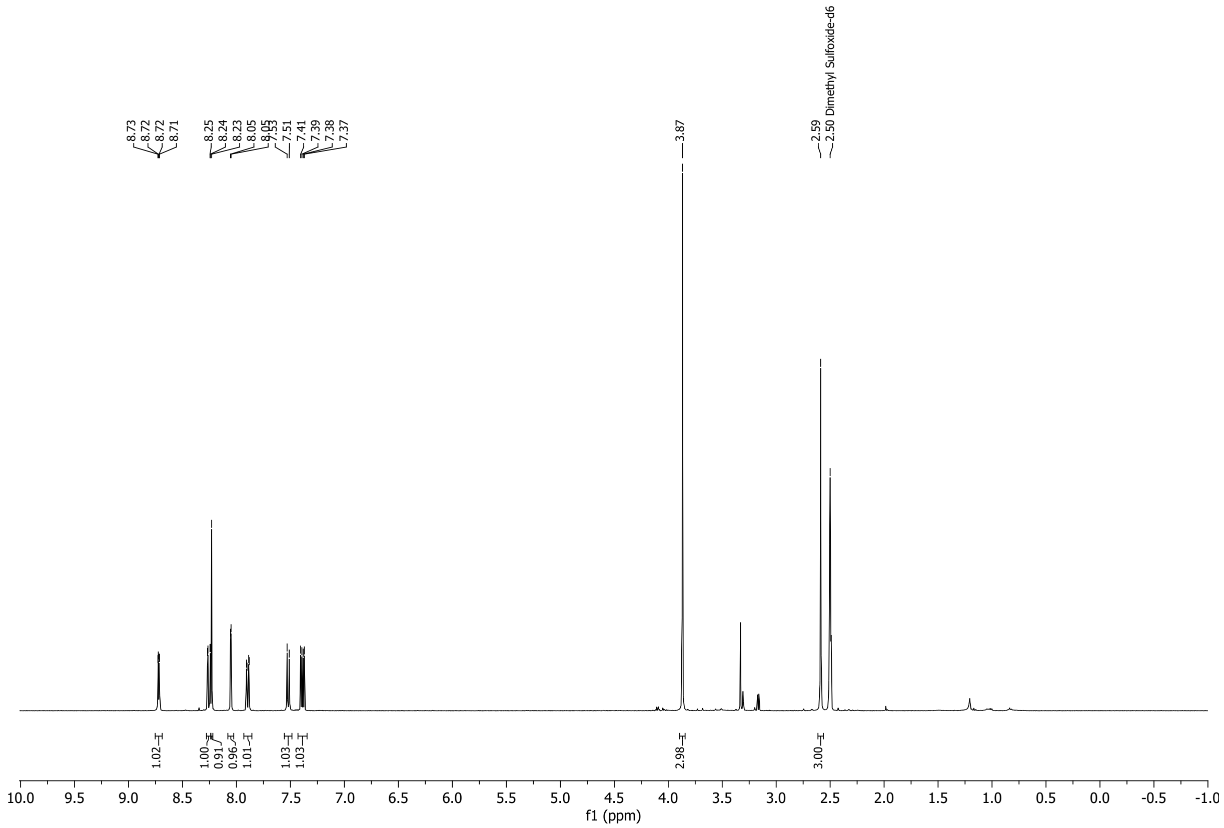

^{13}C NMR (101 MHz, DMSO-d_6):

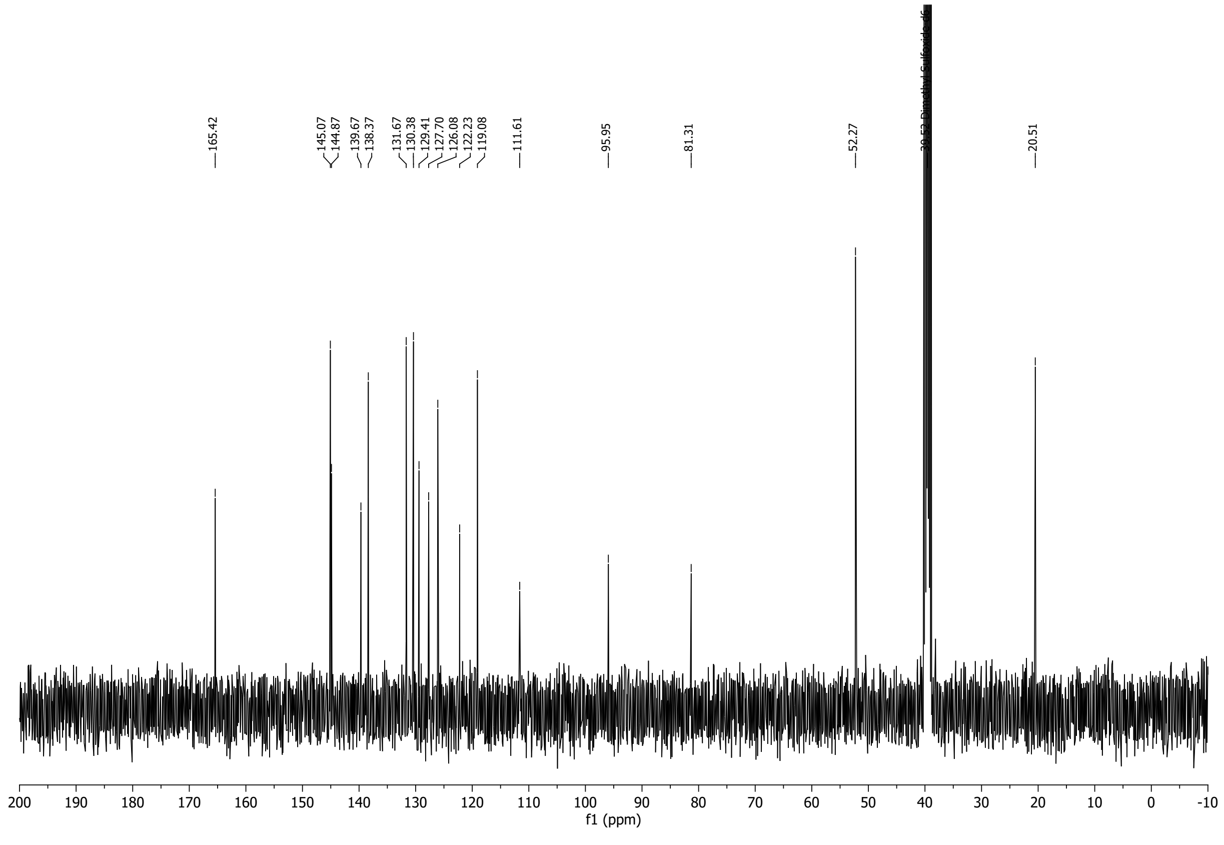

3-((Imidazo[1,2-*b*]pyridazin-3-yl)ethynyl)-4-methylbenzoic acid (17)

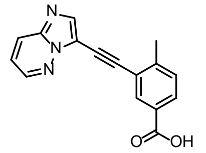

^{1}H NMR (400 MHz, DMSO-d_6):

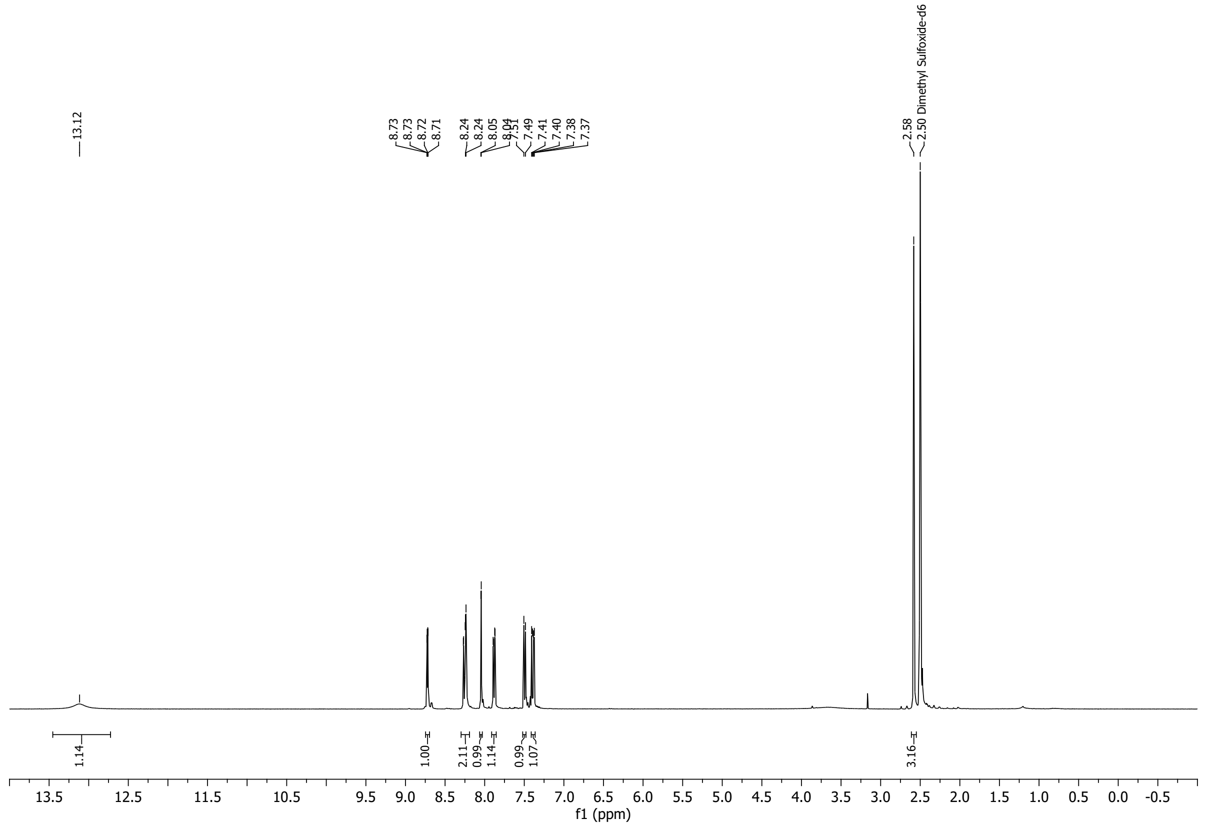

^{13}C NMR (101 MHz, DMSO-d_6):

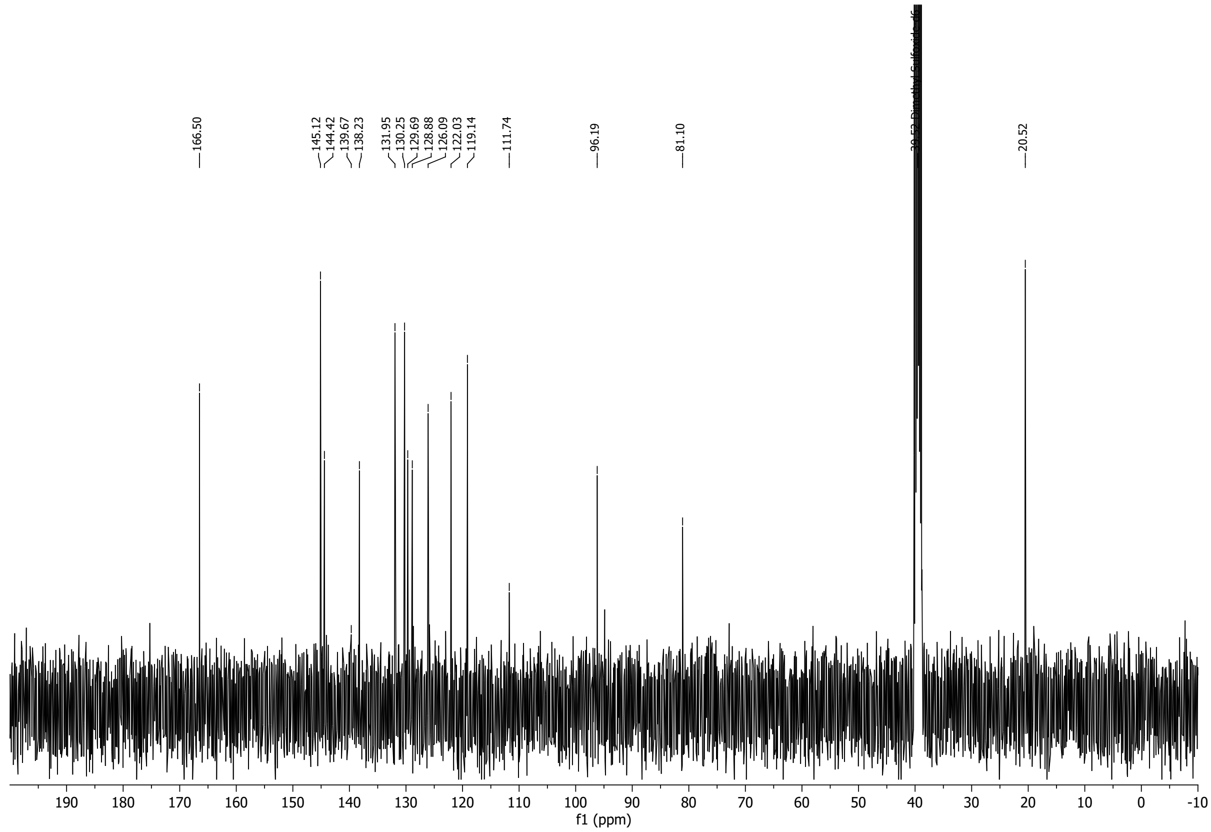

Phenyl-derived thioester 19

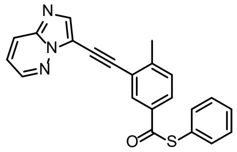

^{1}H NMR (500 MHz, $CDCl_3$):

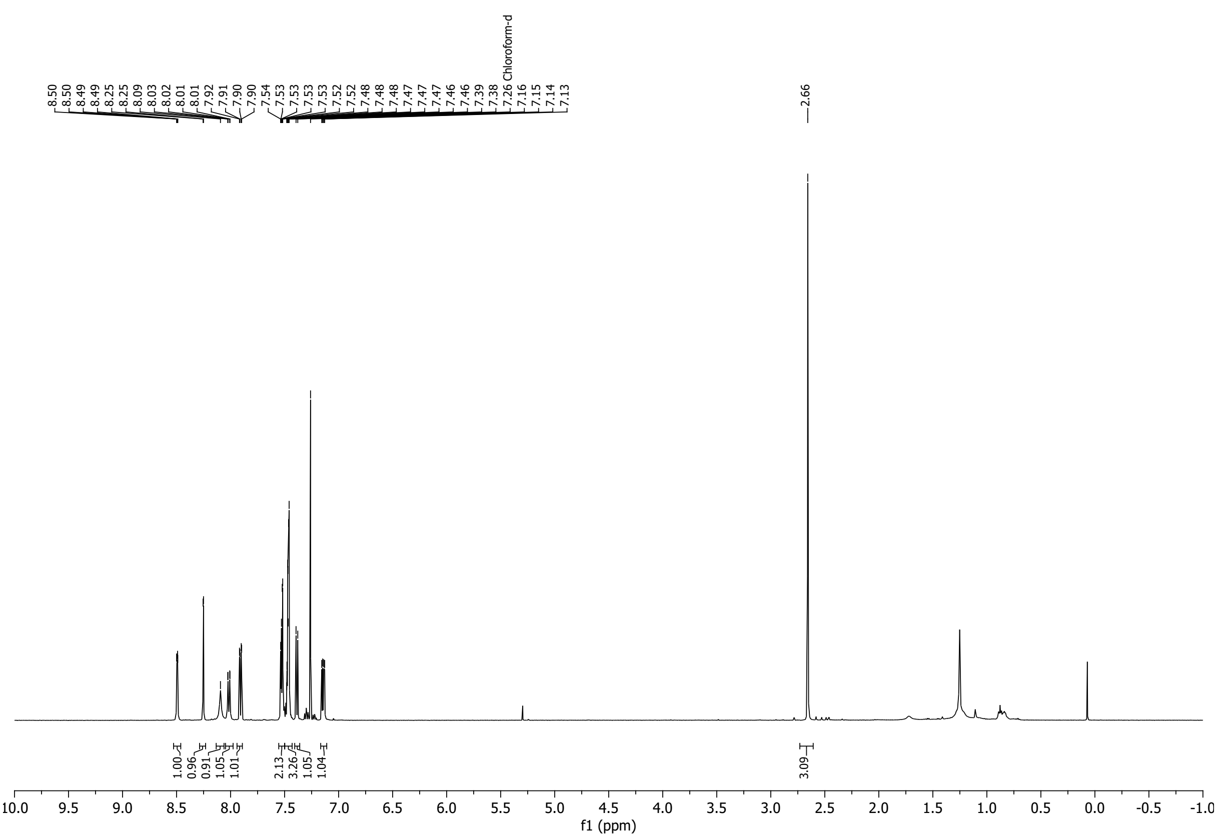

^{13}C NMR (126 MHz, $CDCl_3$):

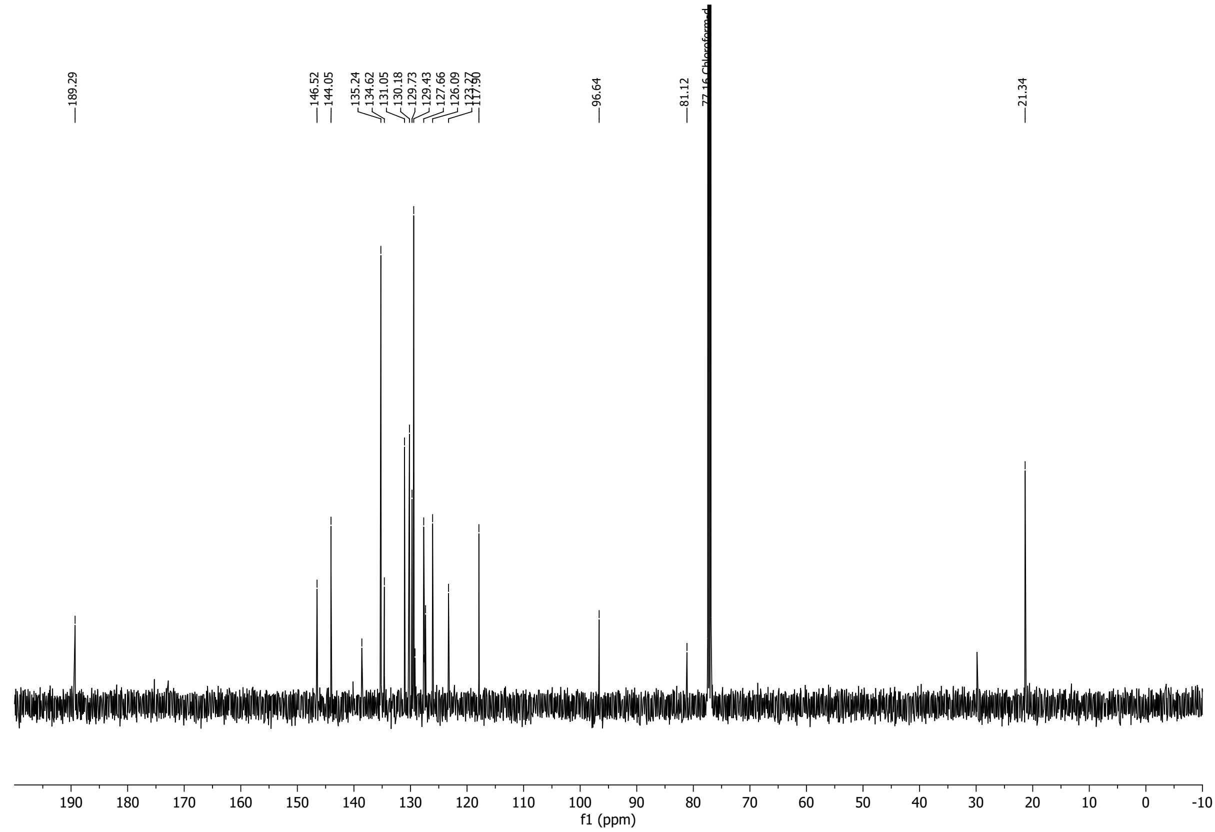

Ponatinib-S(*t*Bu) (38)

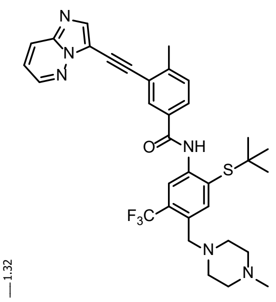

^{1}H NMR (500 MHz, methanol-d_4):

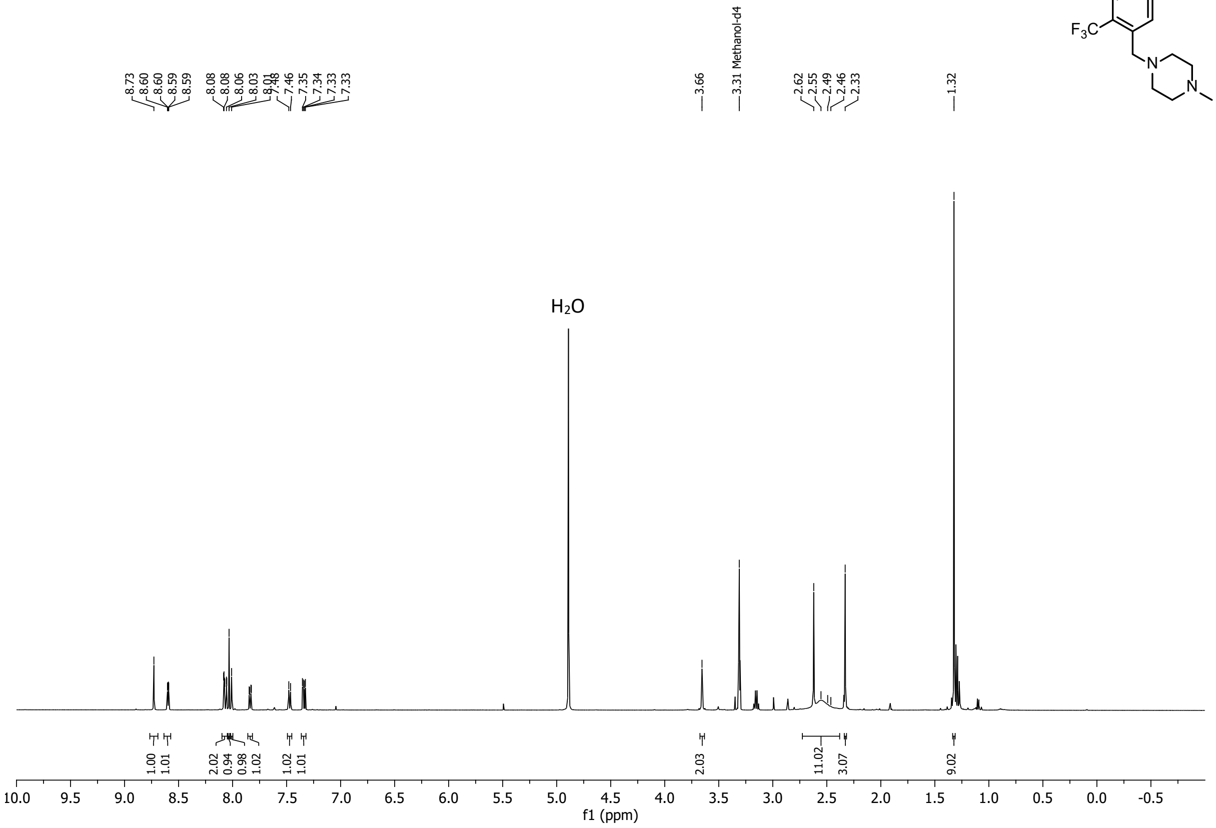

^{13}C NMR (126 MHz, methanol-d_4):

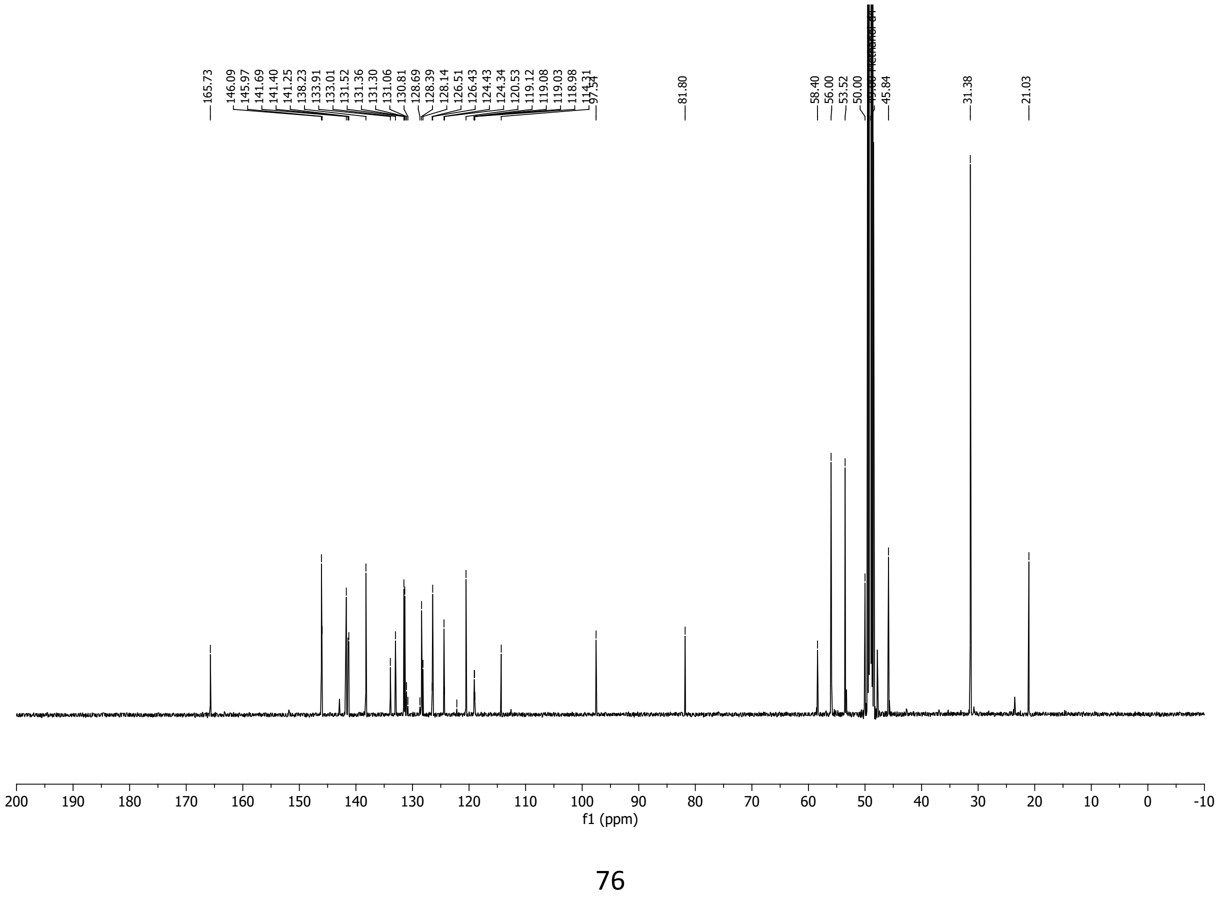

Ponatinib-BT (2b)

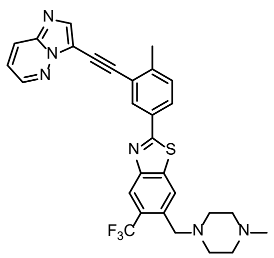

^{1}H NMR (500 MHz, DMSO-d_6):

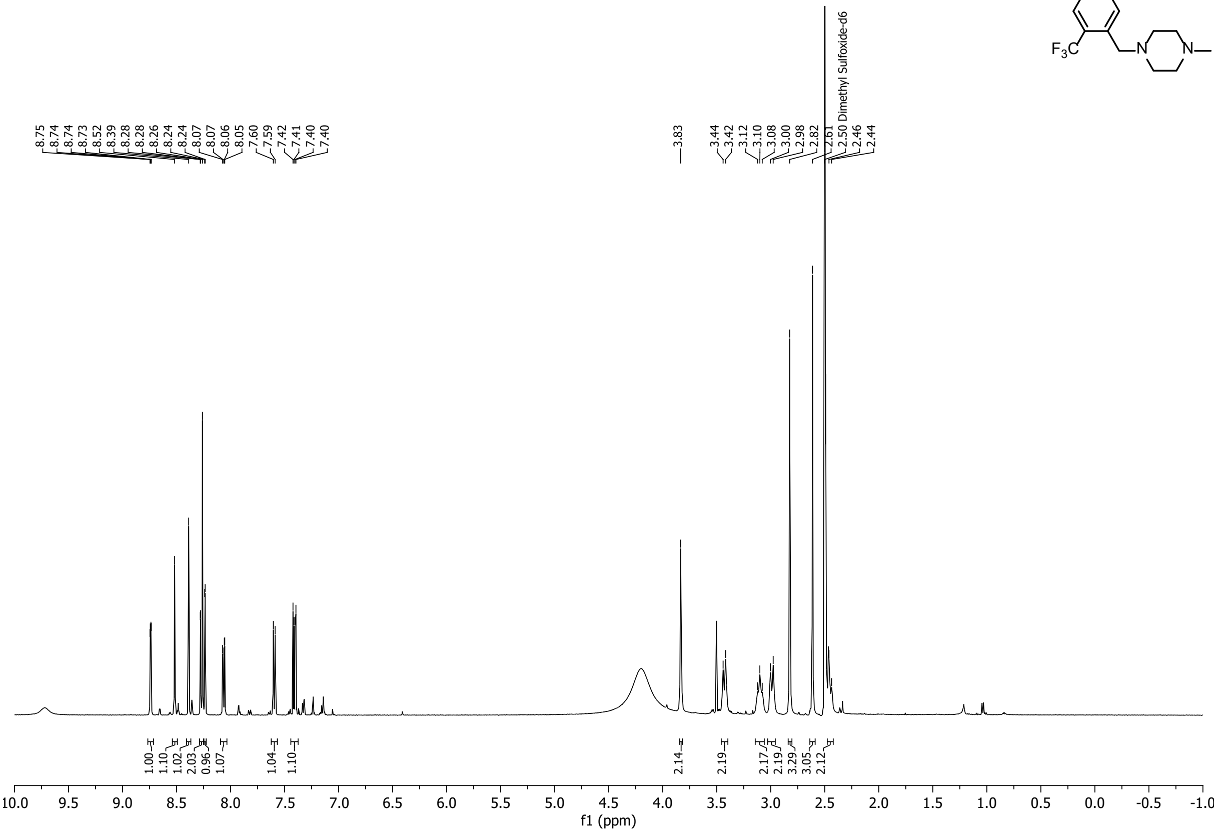

^{13}C NMR (126 MHz, DMSO-d_6):

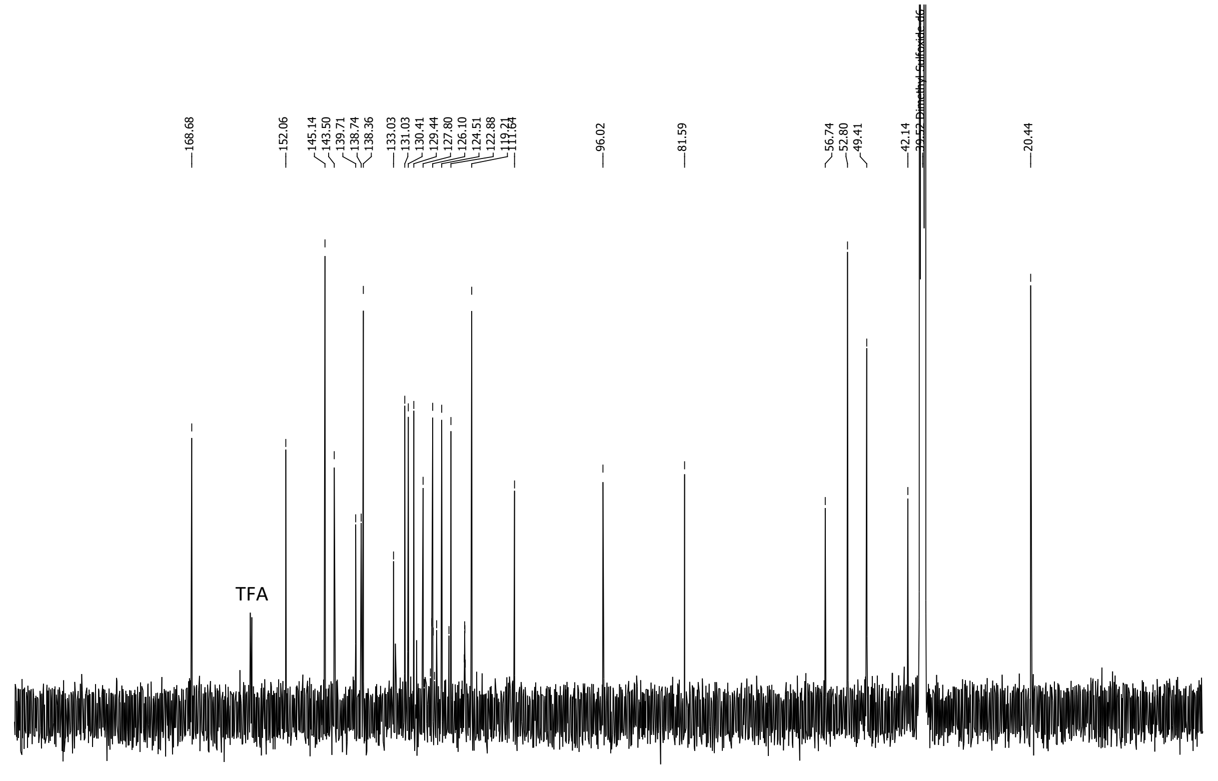

Methyl 3-((1*H*-pyrazolo[3,4-*b*]pyridin-5-yl)ethynyl)-4-methylbenzoate (16)

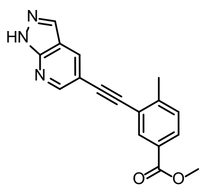

^{1}H NMR (500 MHz, DMSO-d_6):

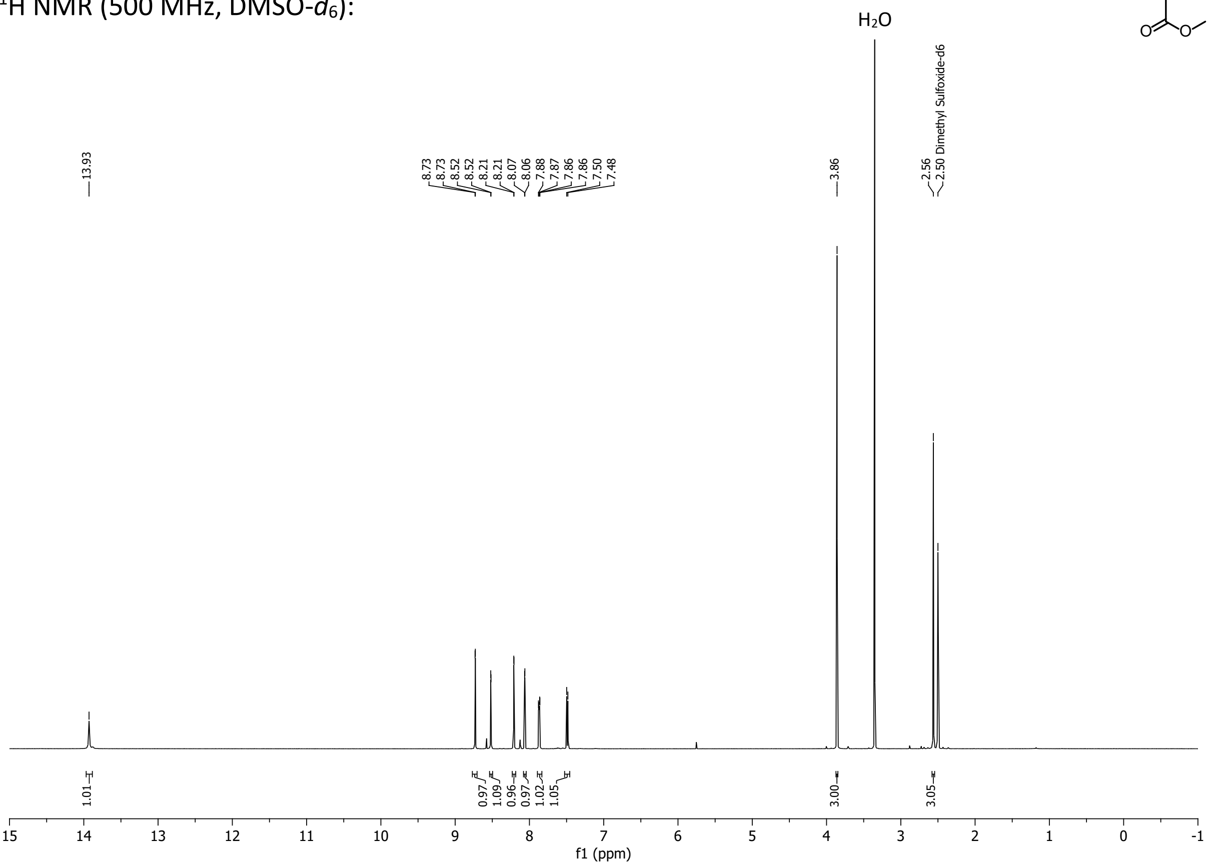

^{13}C NMR (126 MHz, DMSO-d_6):

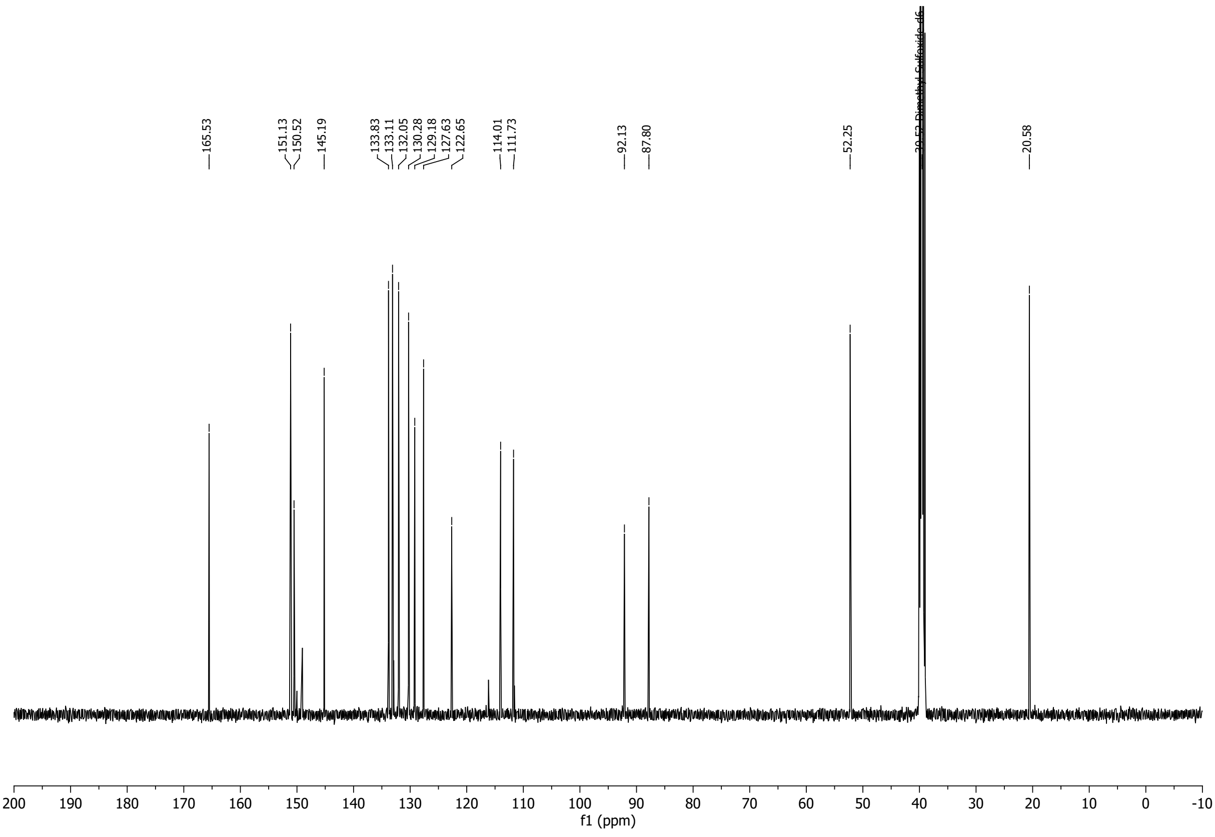

3-((1*H*-pyrazolo[3,4-*b*]pyridin-5-yl)ethynyl)-4-methylbenzoic acid (18)

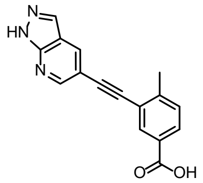

^{1}H NMR (400 MHz, DMSO-d_6):

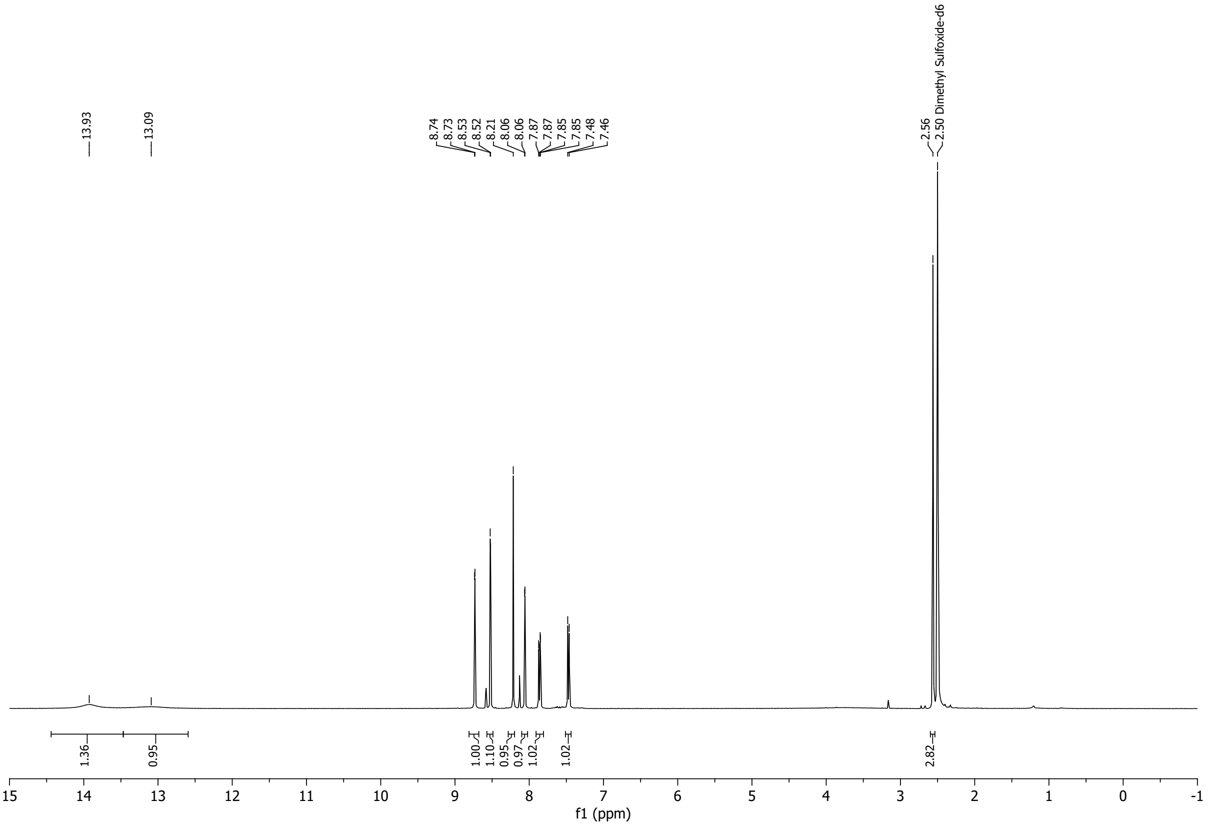

^{13}C NMR (101 MHz, DMSO-d_6):

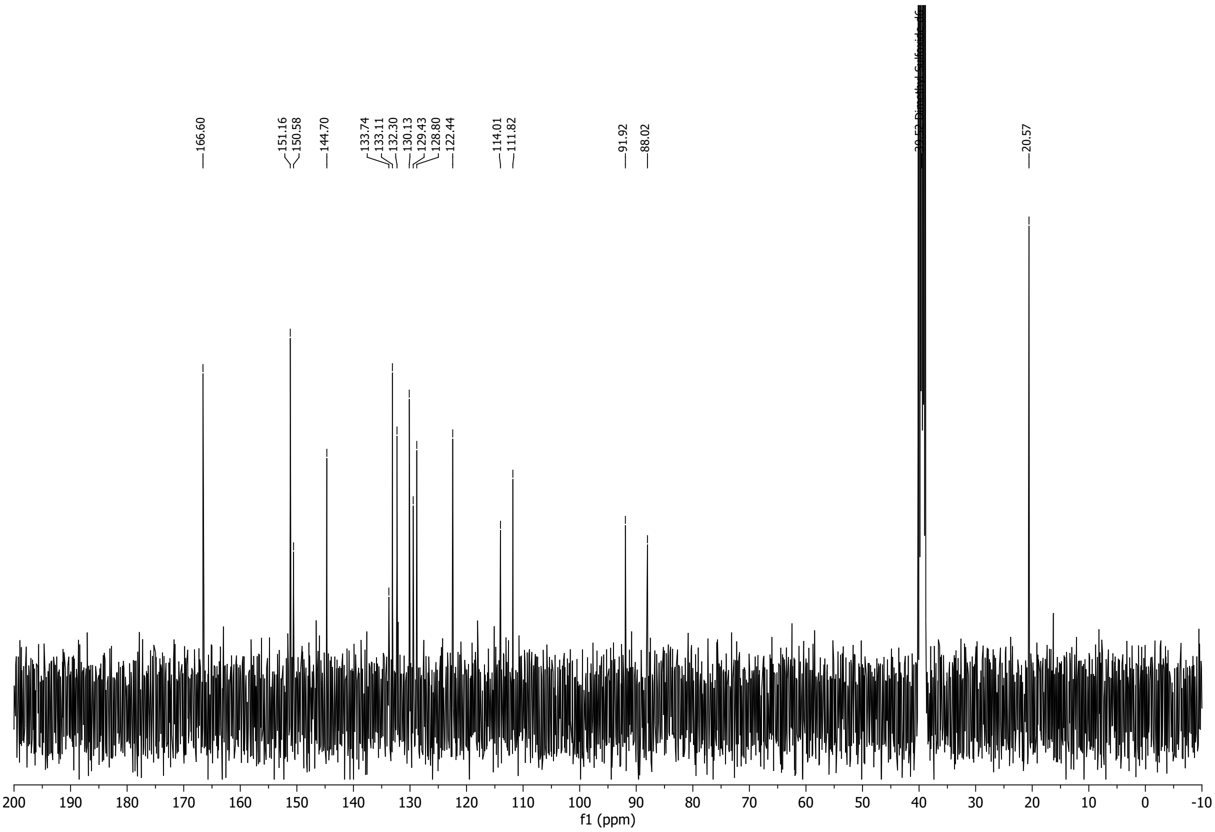

Phenyl-derived thioester 20

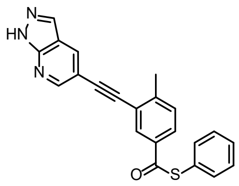

^{1}H NMR (500 MHz, DMSO-d_6):

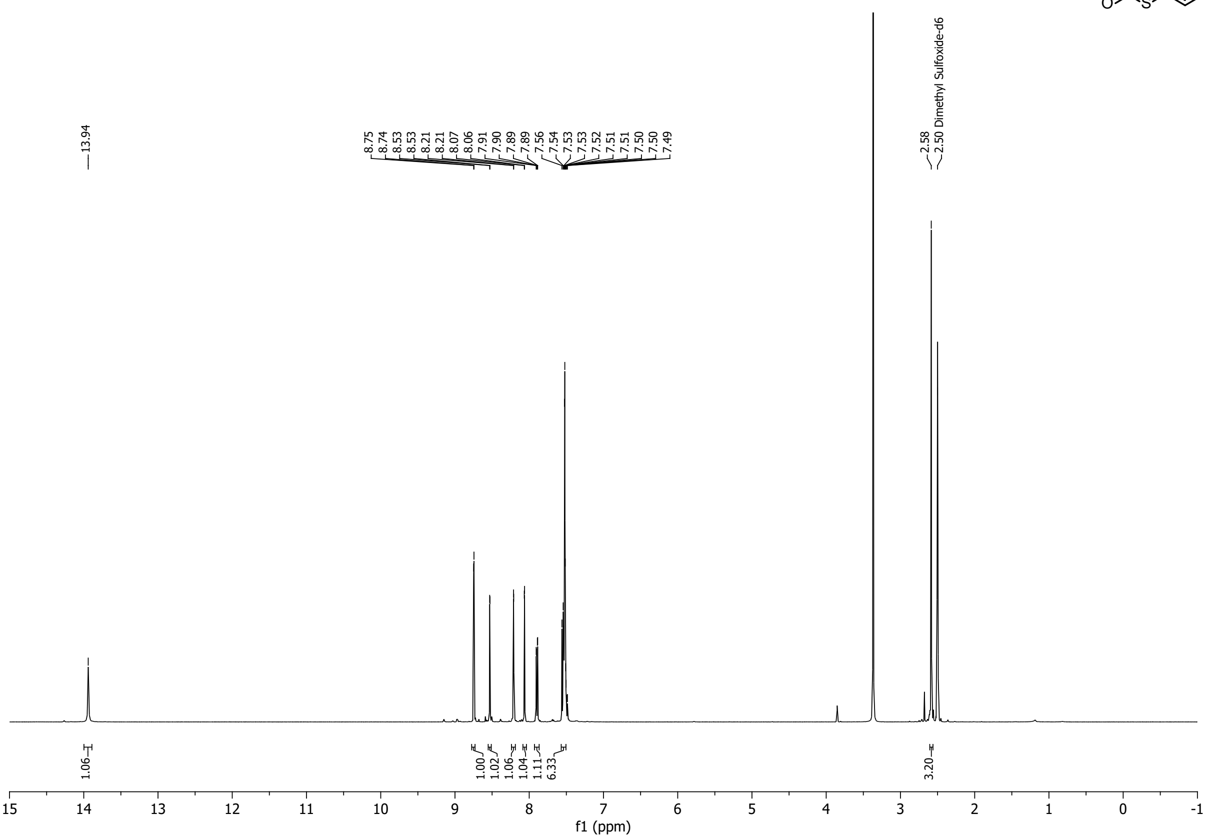

^{13}C NMR (126 MHz, DMSO-d_6):

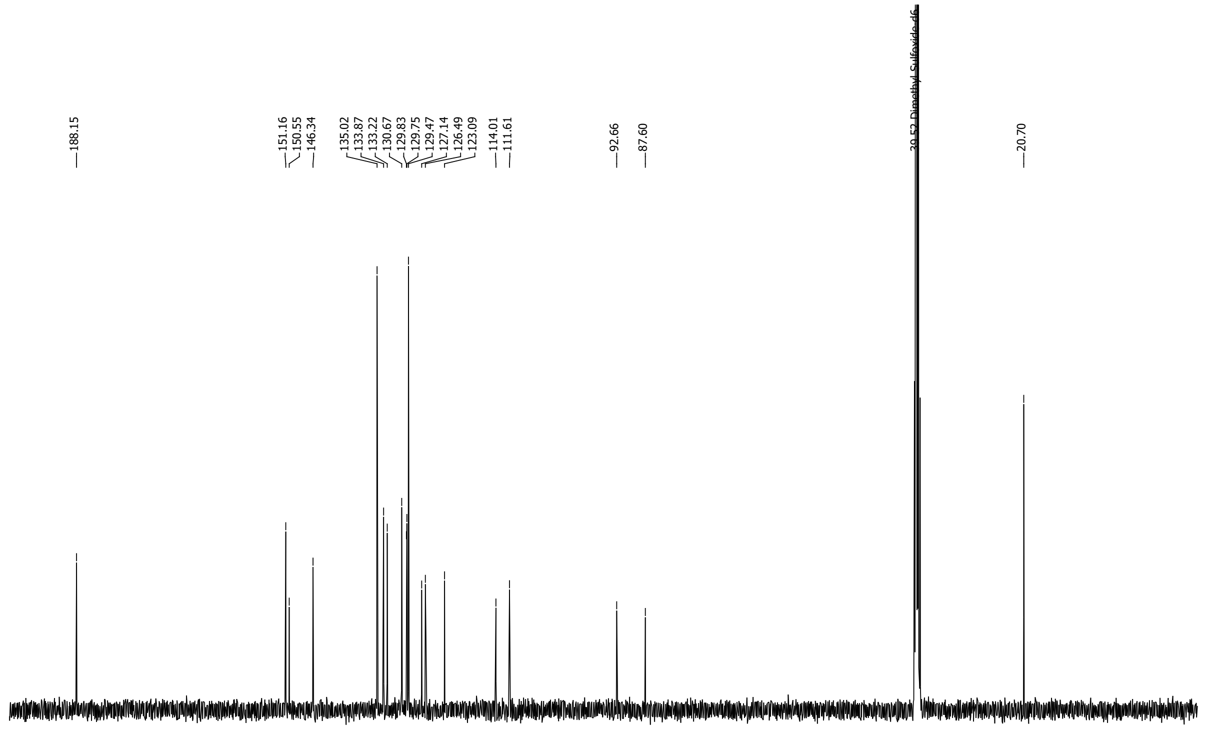

GZD824-S(*t*Bu) (39)

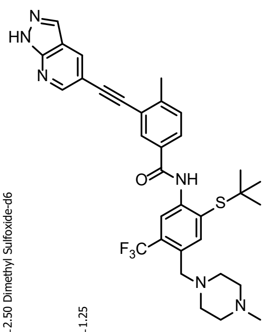

^{1}H NMR (400 MHz, DMSO-d_6):

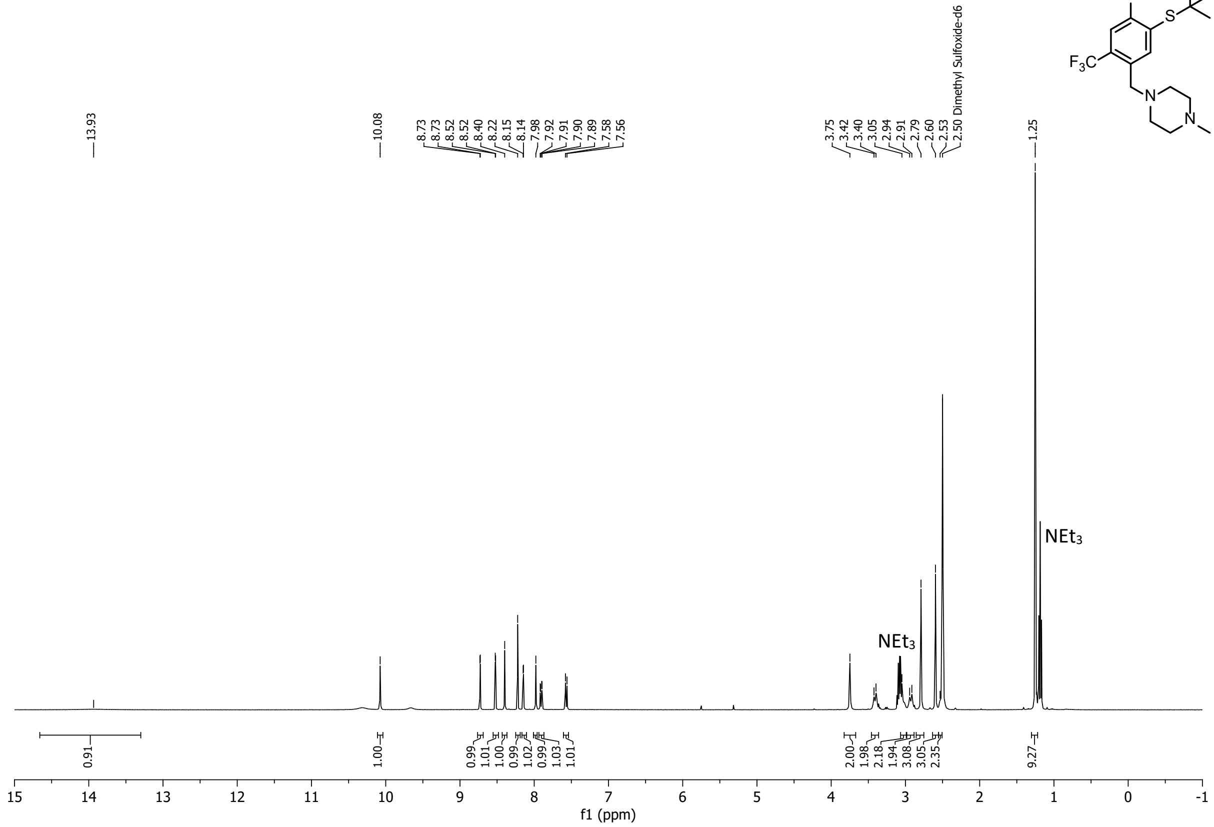

^{13}C NMR (126 MHz, DMSO-d_6):

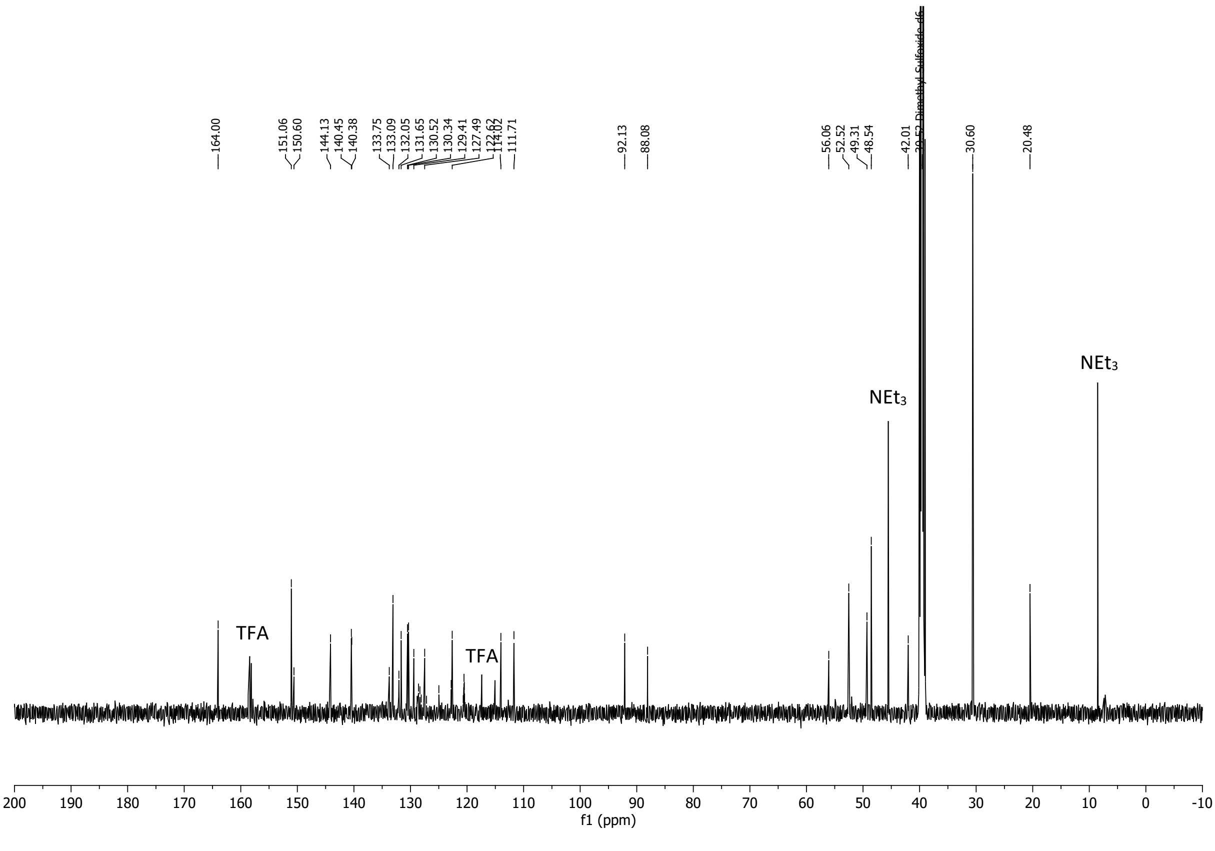

GZD824-BT (3b)

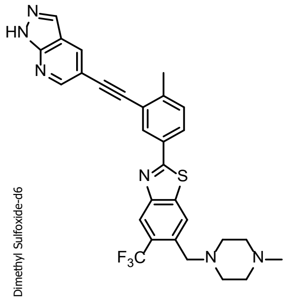

^{1}H NMR (500 MHz, DMSO-d_6):

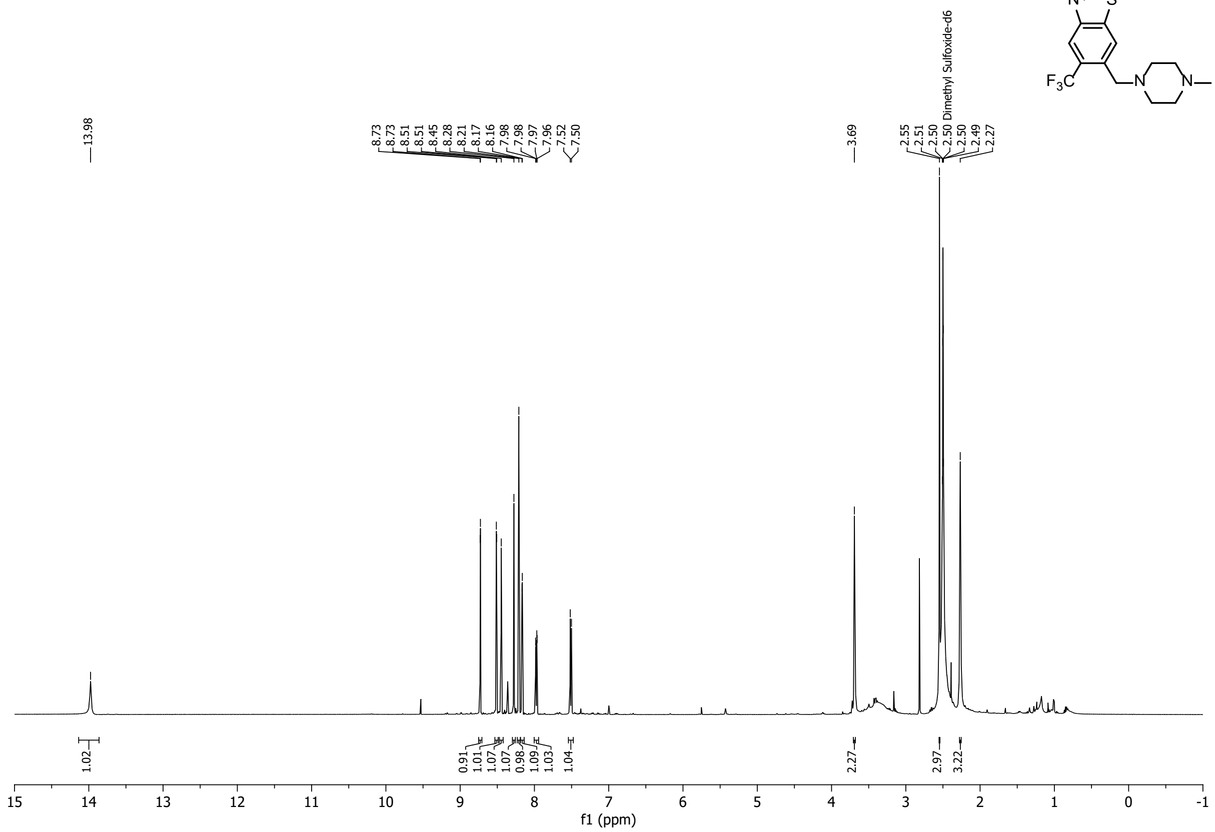

^{13}C NMR (126 MHz, DMSO-d_6):

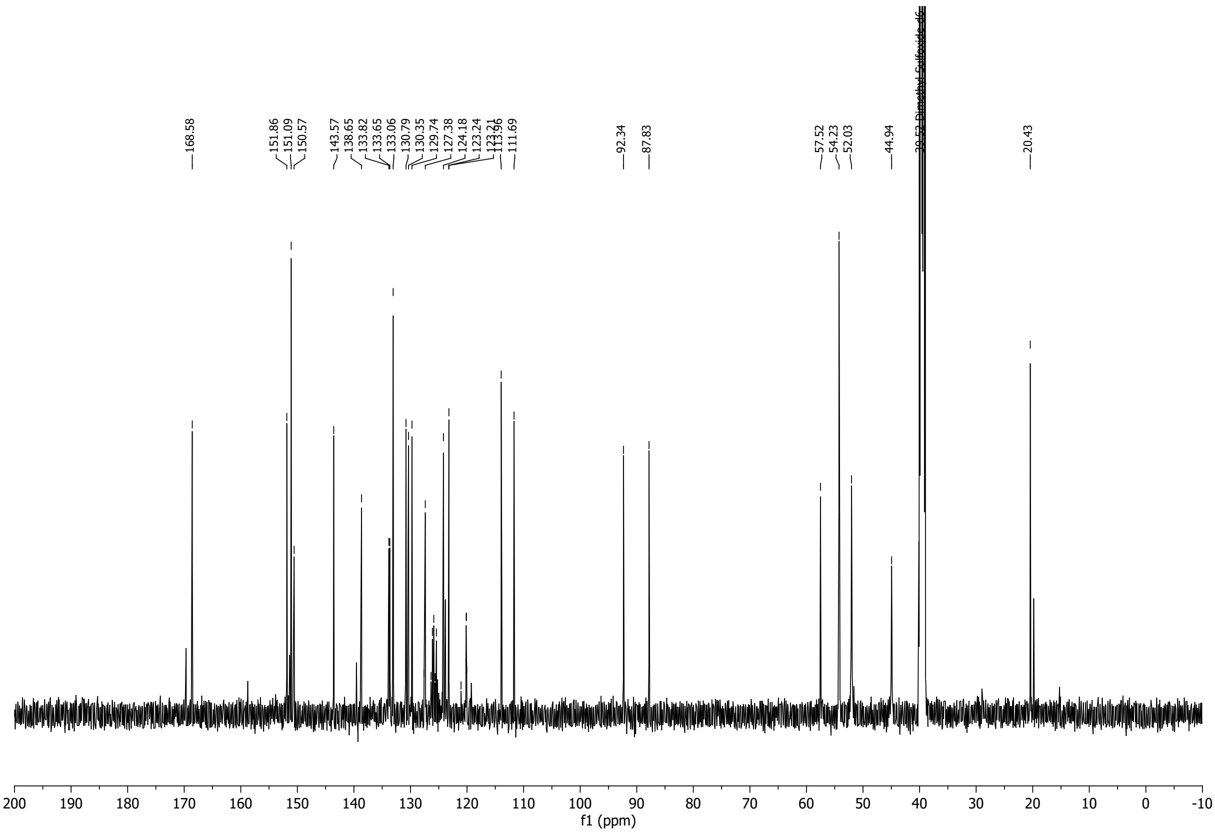

Ponatinib-SH (2a)

^{1}H NMR (500 MHz, DMSO-d_6):

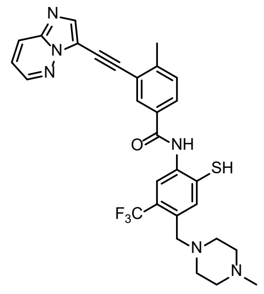

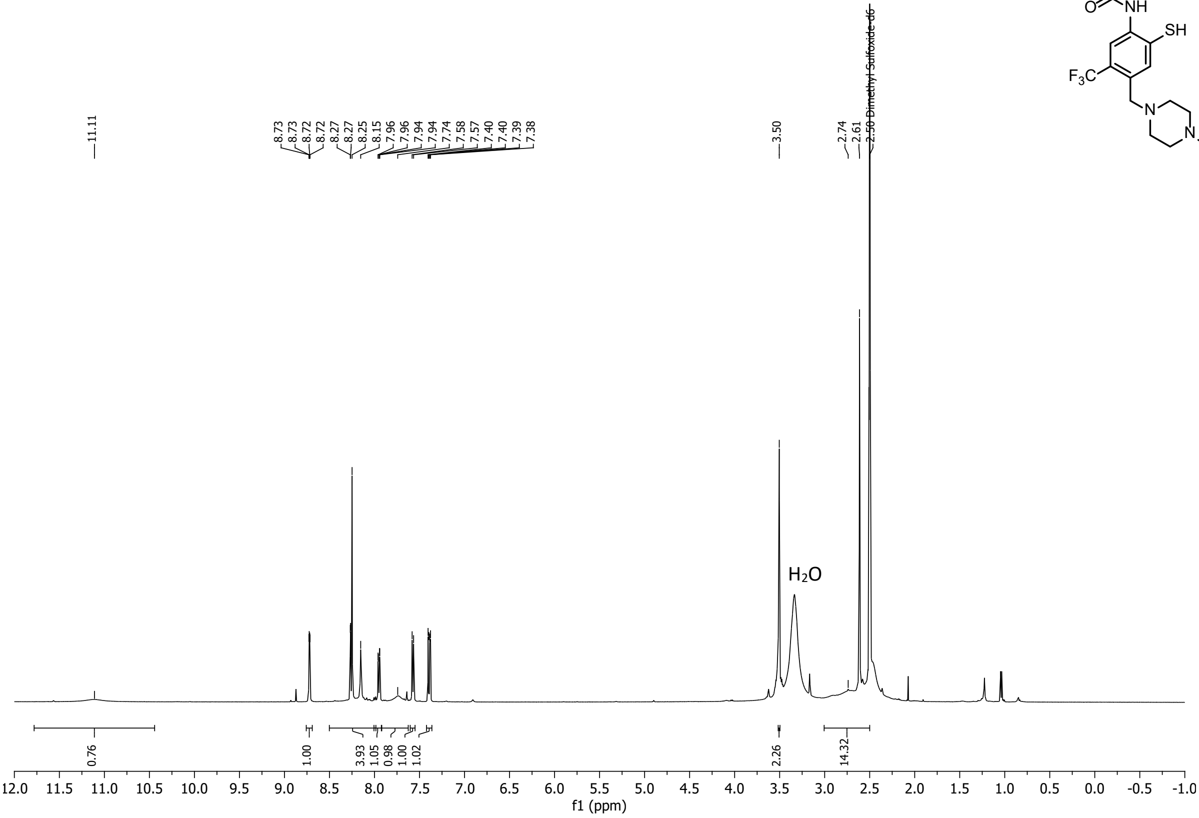

GZD824-SH (3a)

^{1}H NMR (500 MHz, DMSO-d_6):

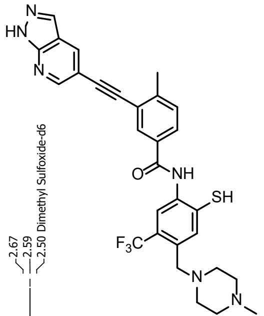

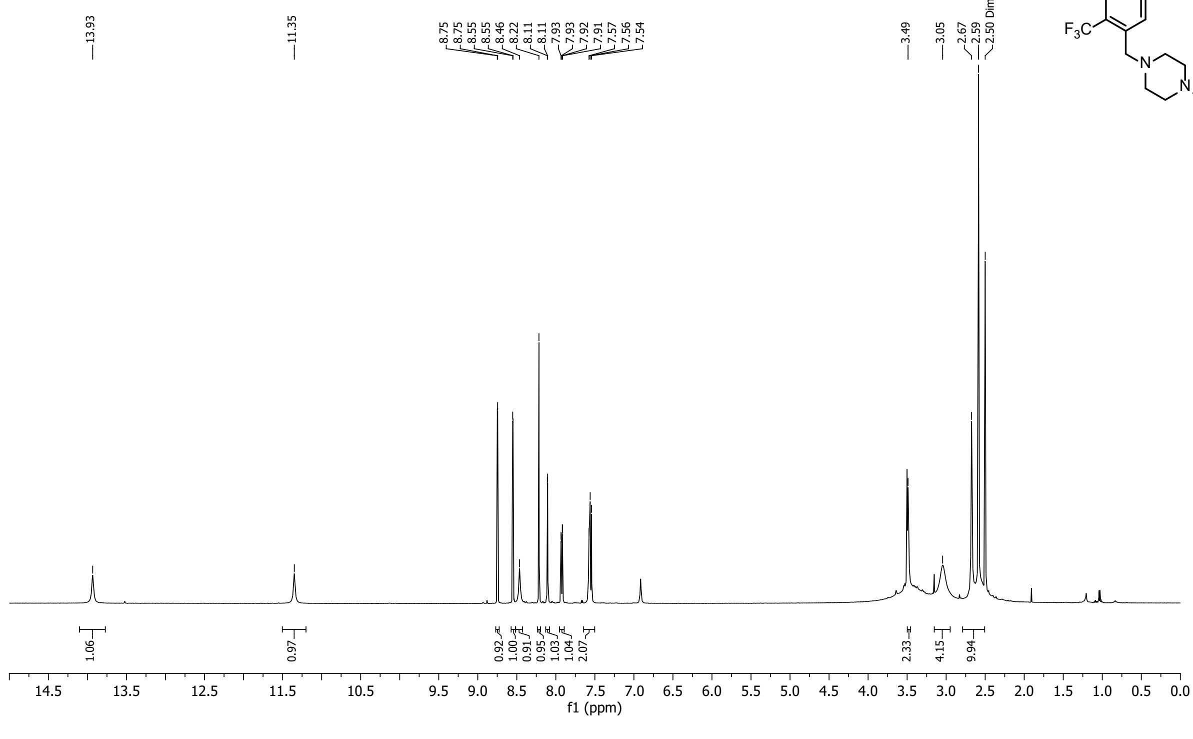

Ethyl 2-(4-(5-chloro-4-nitro-2-(trifluoromethyl)benzyl)piperazin-1-yl)-acetate (40)

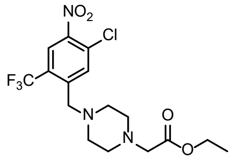

^1H NMR (400 MHz, $CDCl_3$):

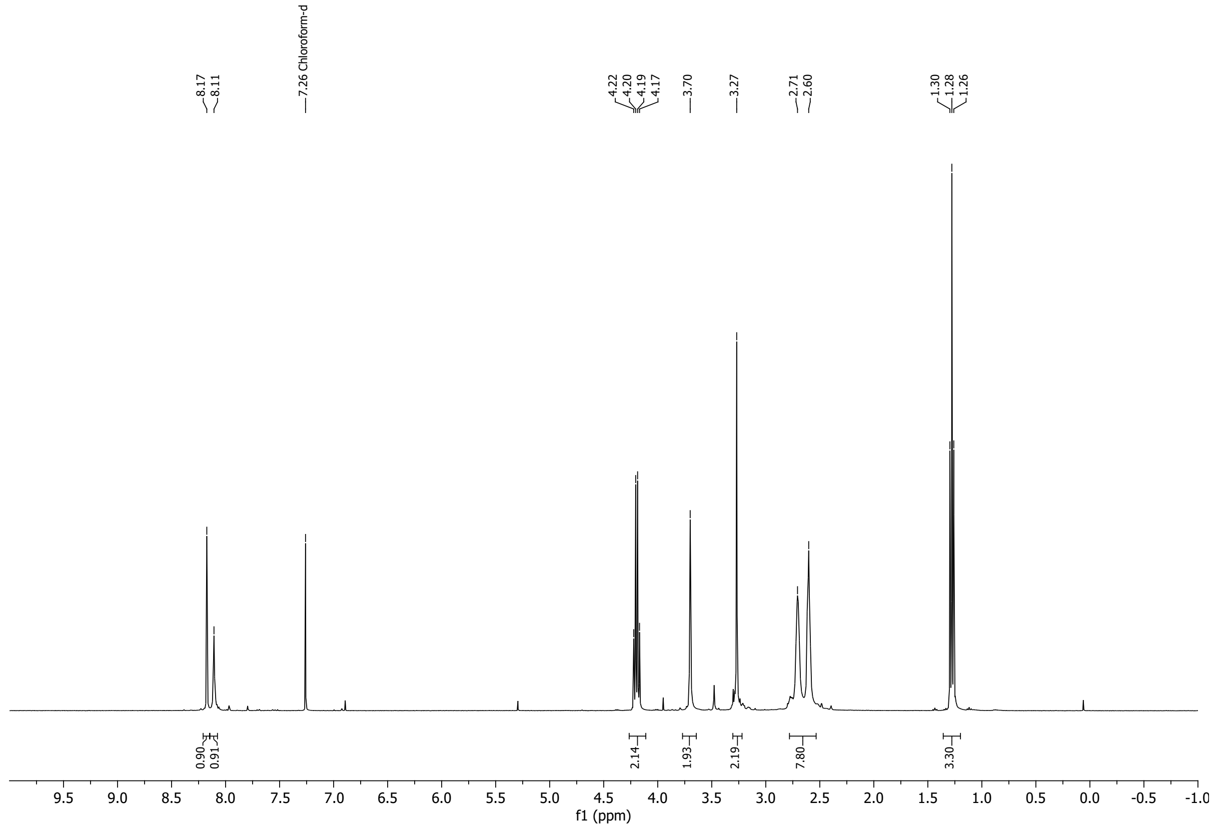

^{13}C NMR (101 MHz, $CDCl_3$):

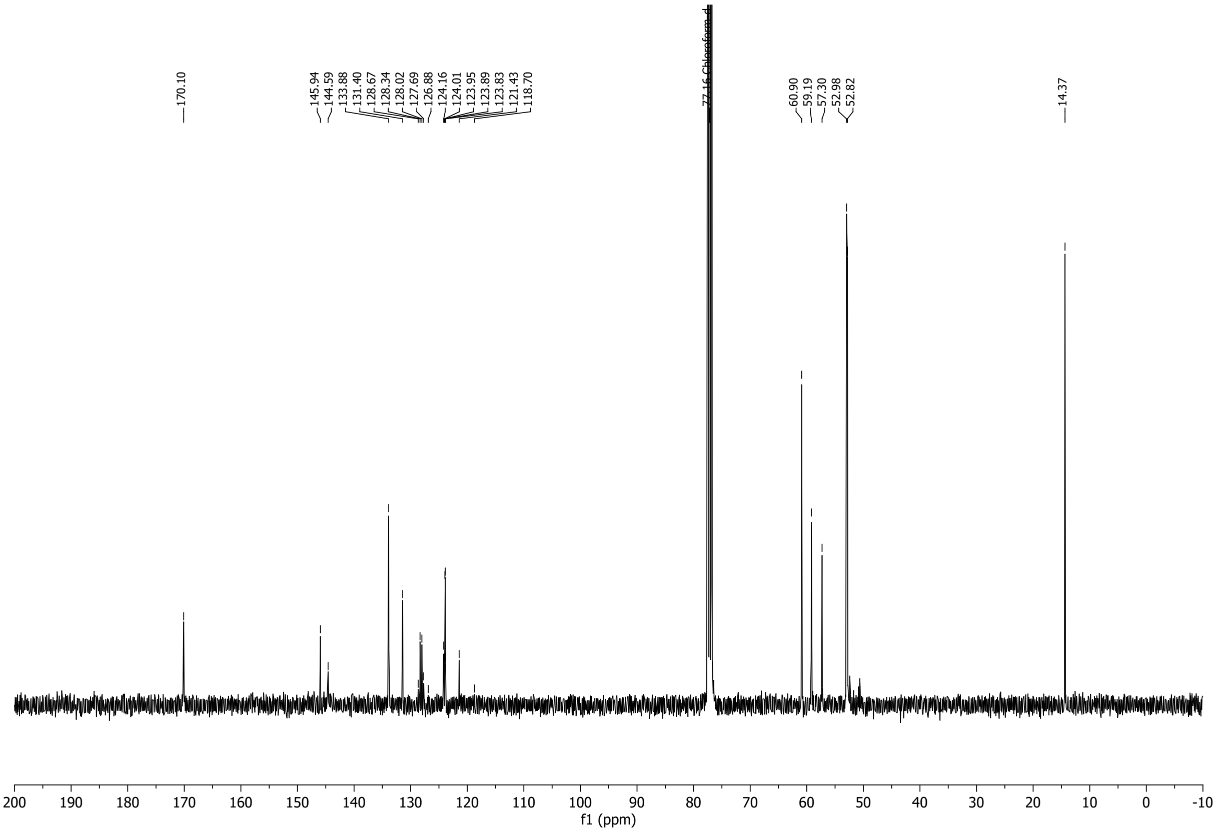

Ethyl 2-(4-(5-(*tert*-butylthio)-4-nitro-2-(trifluoromethyl)benzyl)-piperazin-1-yl)acetate (41)

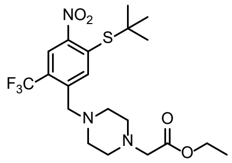

^{1}H NMR (400 MHz, $CDCl_3$):

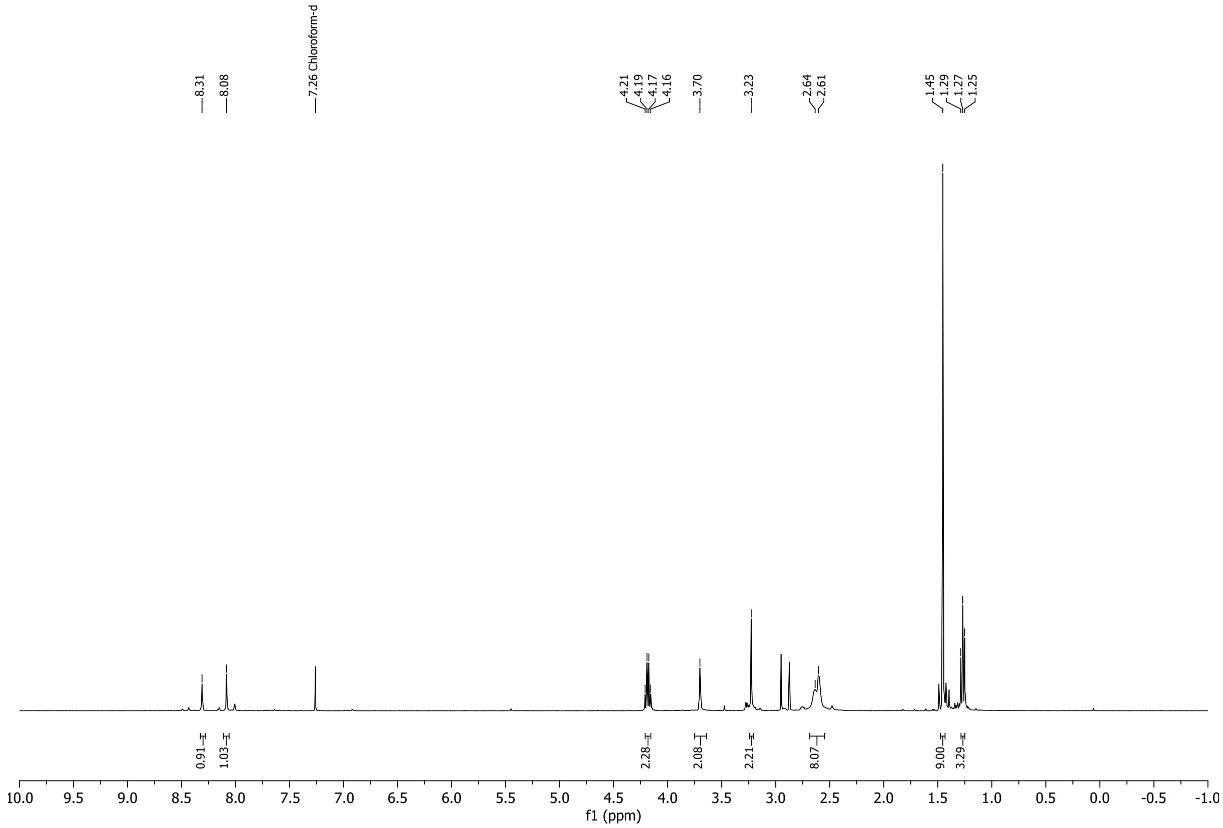

^{13}C NMR (101 MHz, $CDCl_3$):

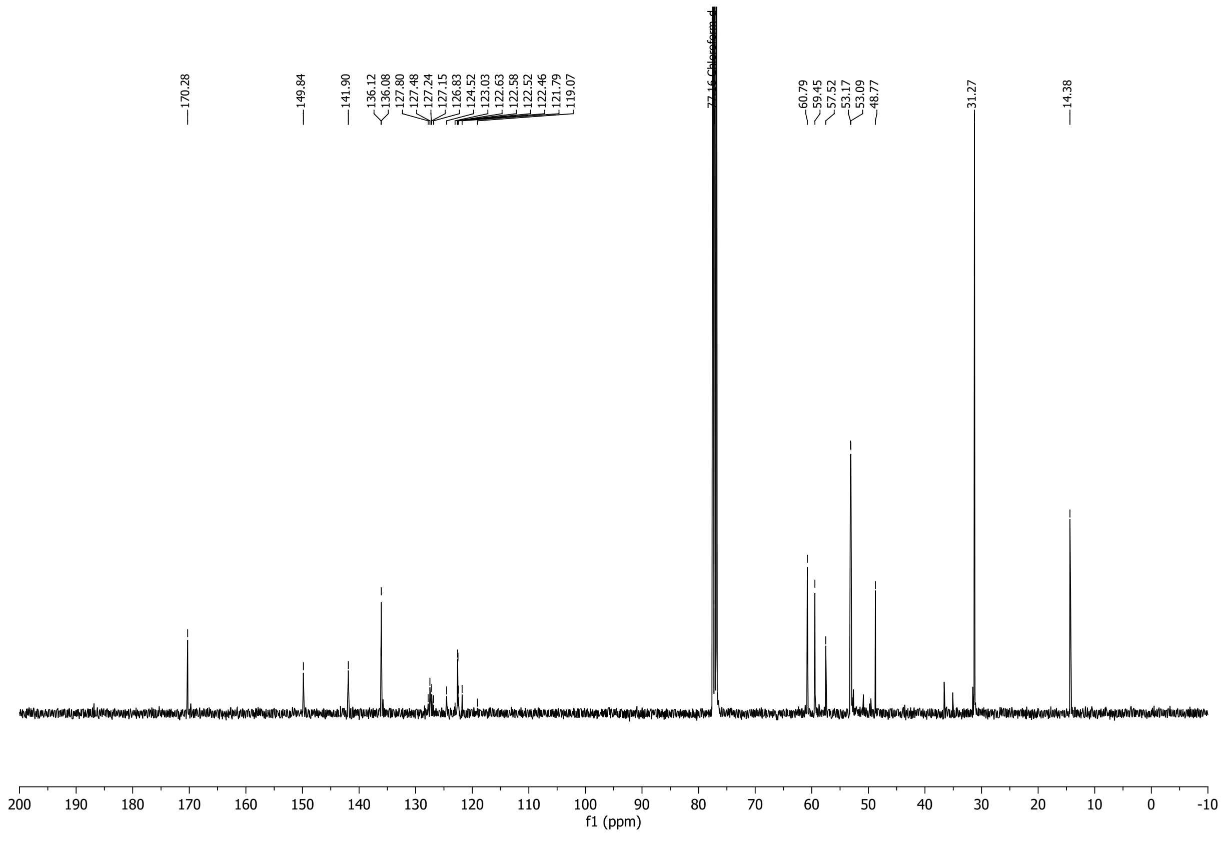

Ethyl 2-(4-(4-amino-5-(*tert*-butylthio)-2-(trifluoromethyl)benzyl)-piperazin-1-yl)acetate (42)

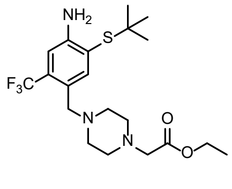

^{1}H NMR (400 MHz, $CDCl_3$):

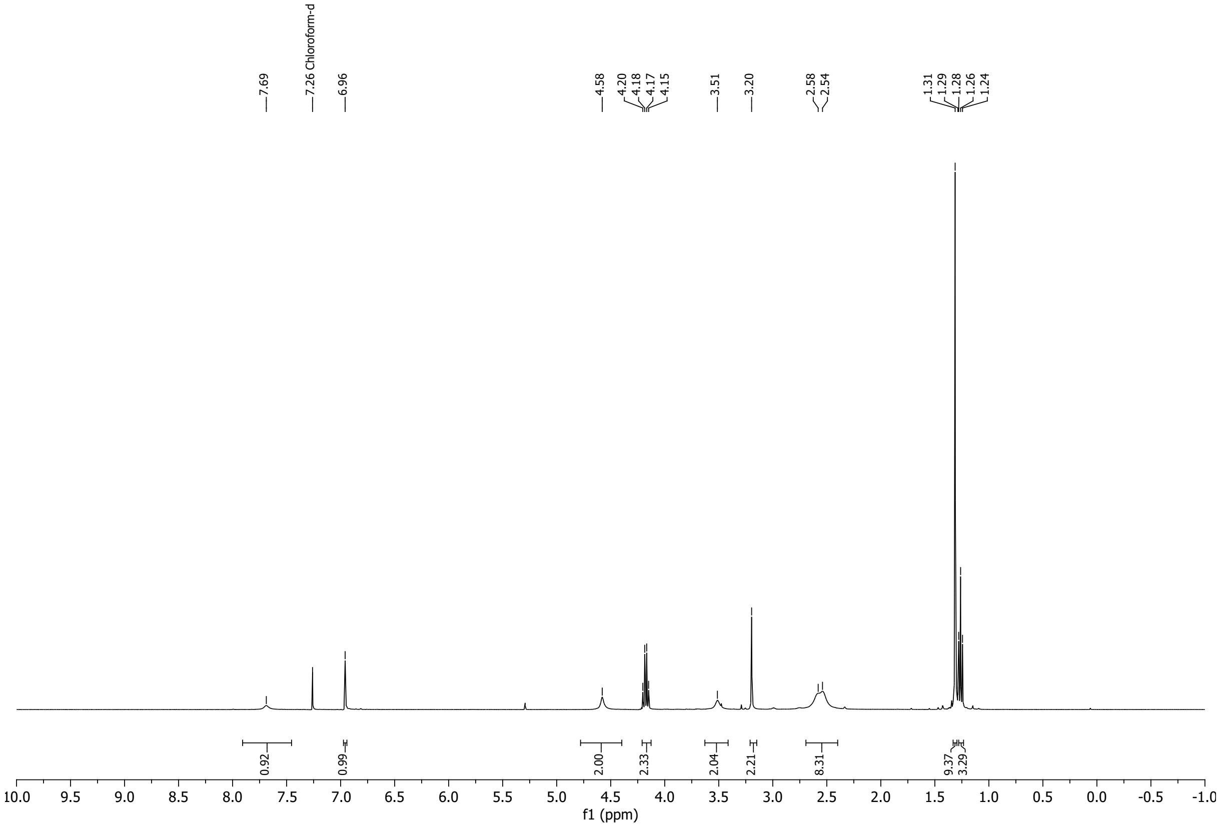

^{13}C NMR (101 MHz, $CDCl_3$):

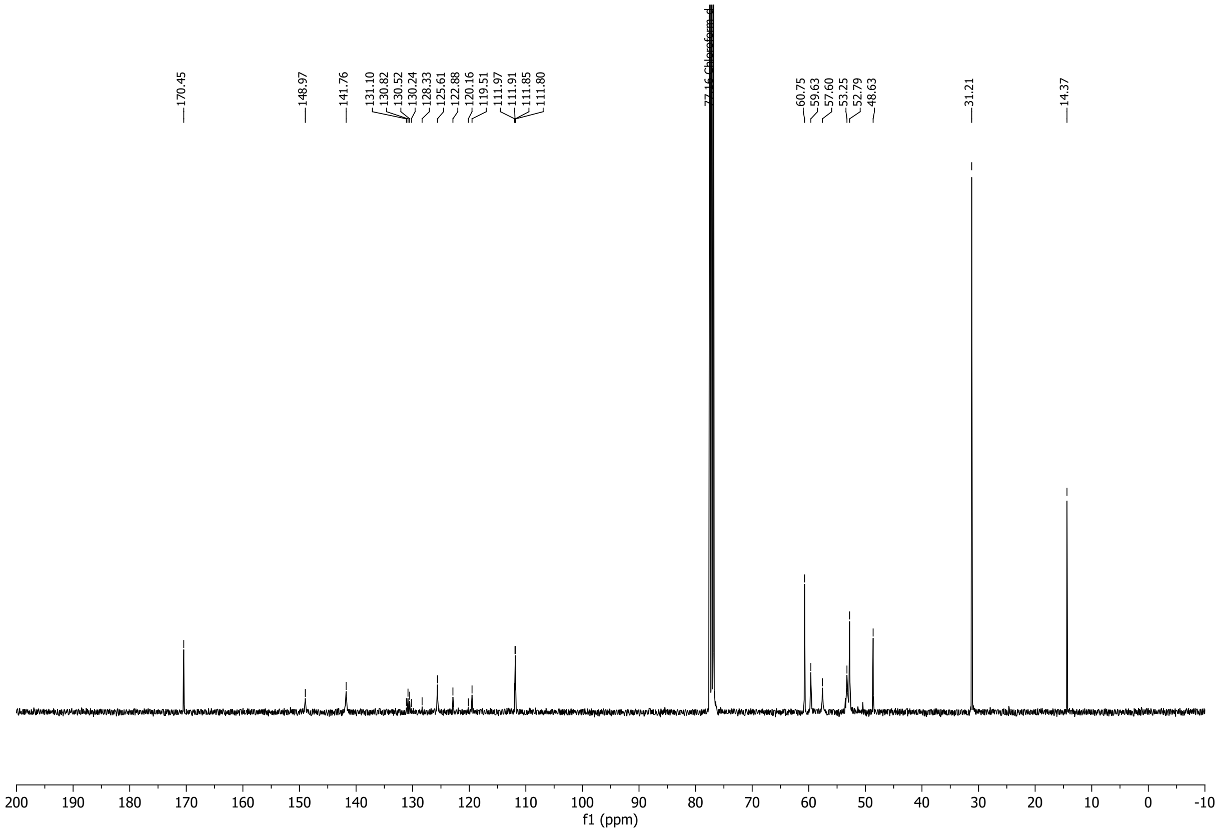

Ethyl 2-(4-(4-(di(*tert*-butyloxycarbonyl)amino)-5-(*tert*-butylthio)-2-(trifluoromethyl)benzyl)piperazin-1-yl)acetate (43)

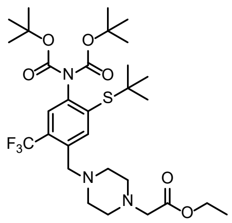

^{1}H NMR (500 MHz, $CDCl_3$):

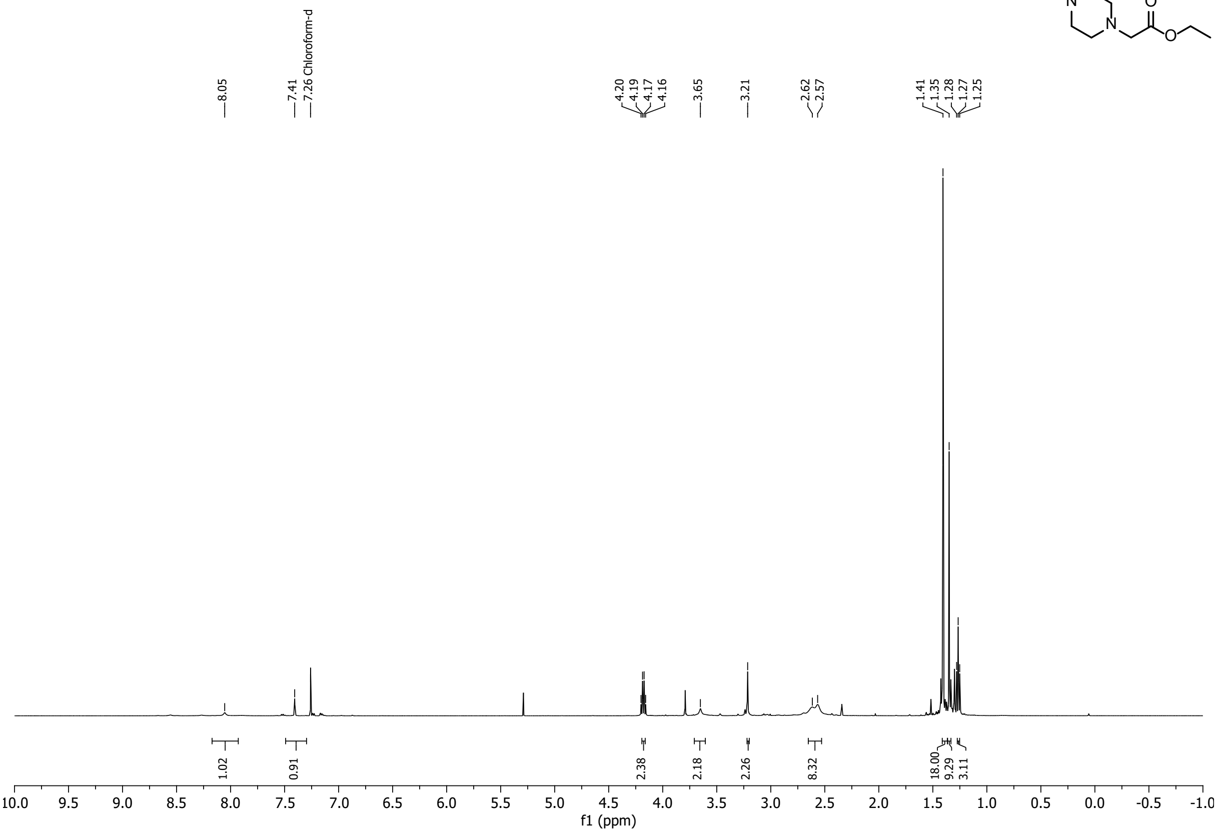

^{13}C NMR (126 MHz, $CDCl_3$):

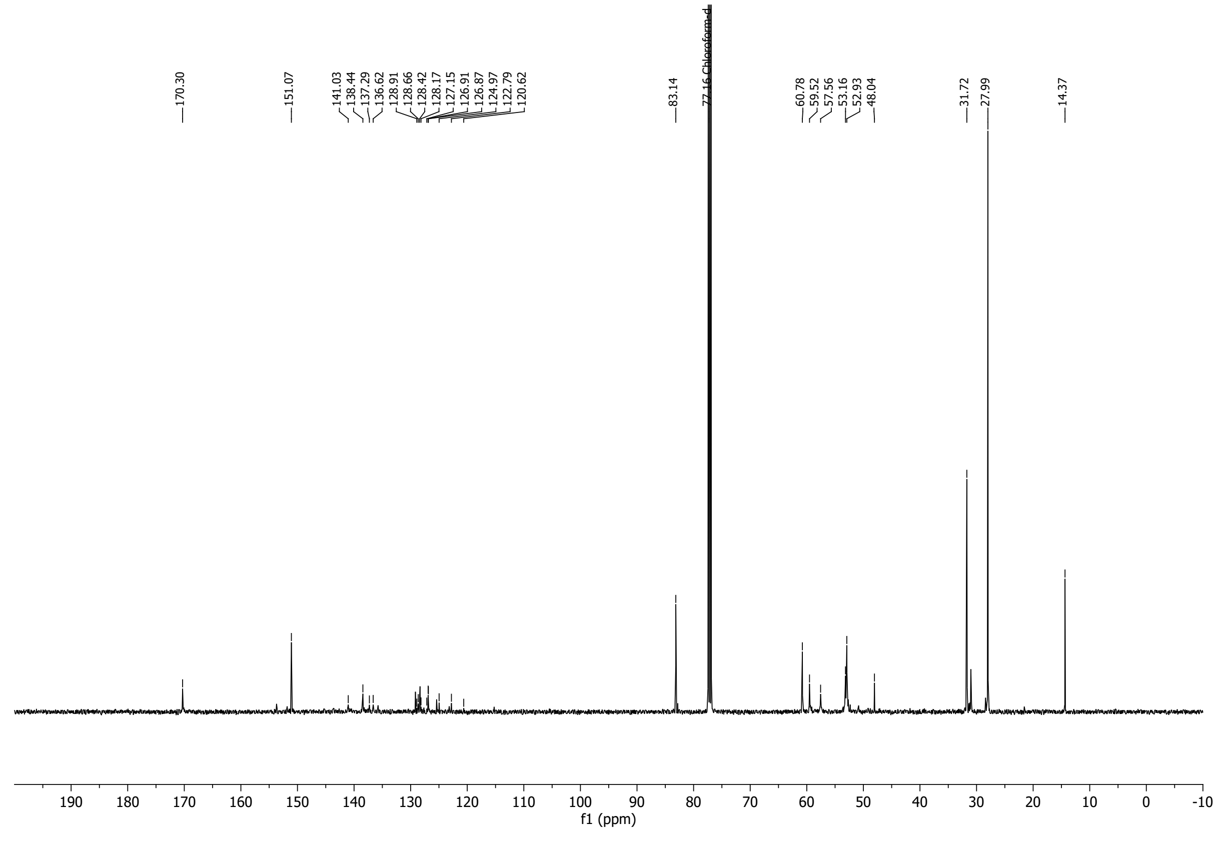

2-(4-(4-(Di(*tert*-butyloxycarbonyl)amino)-5-(*tert*-butylthio)-2-(trifluoromethyl)benzyl)piperazin-1-yl)acetic acid (21)

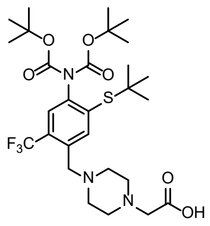

^{1}H NMR (500 MHz, DMSO-d_6):

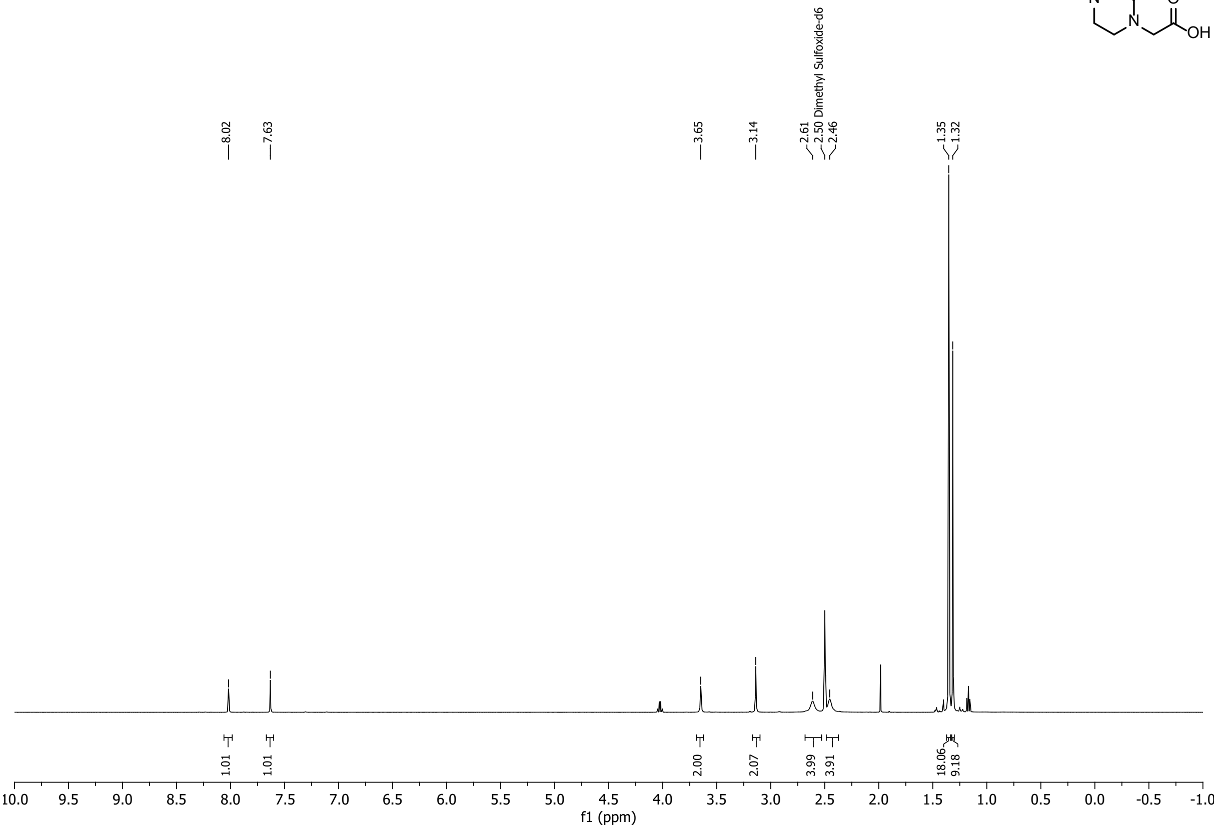

^{13}C NMR (126 MHz, DMSO-d_6):

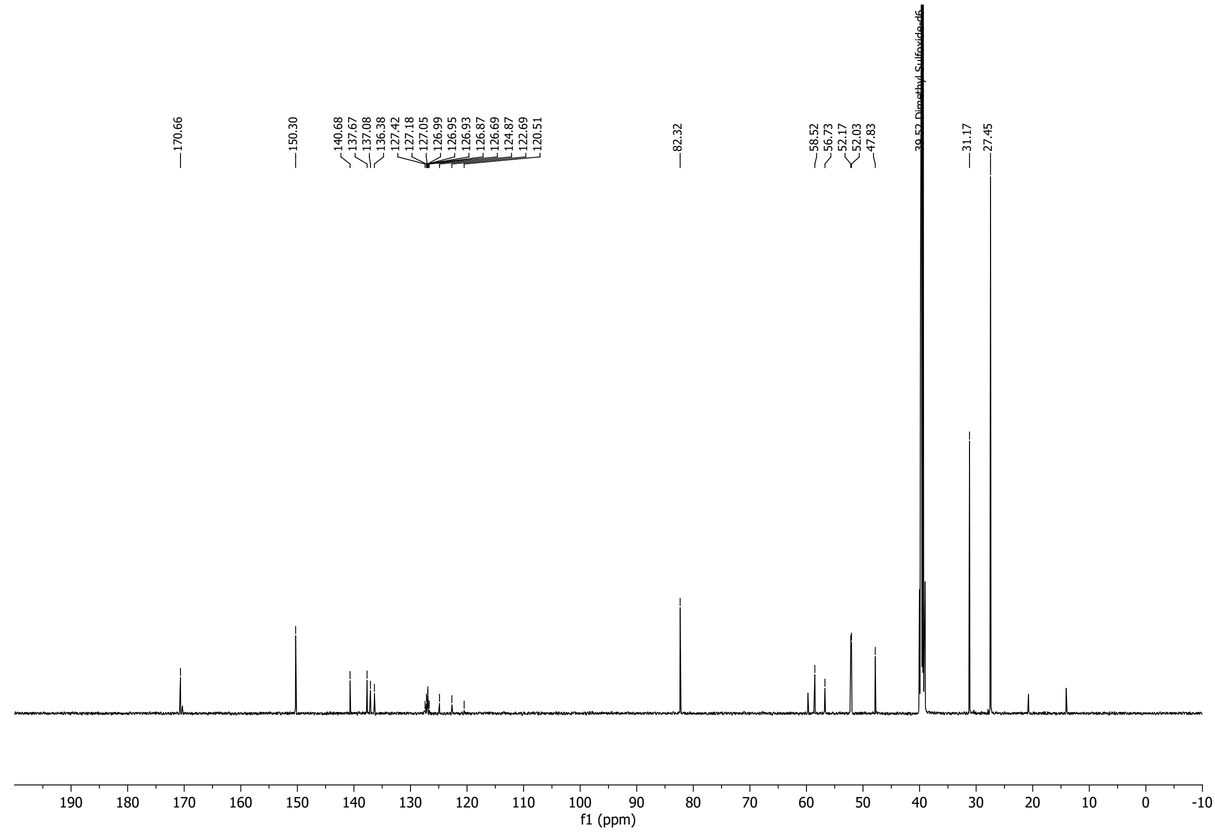

Disulfide 44

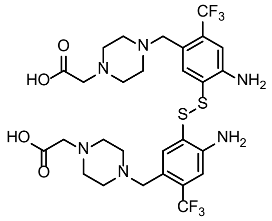

^{1}H NMR (500 MHz, DMSO-d_6):

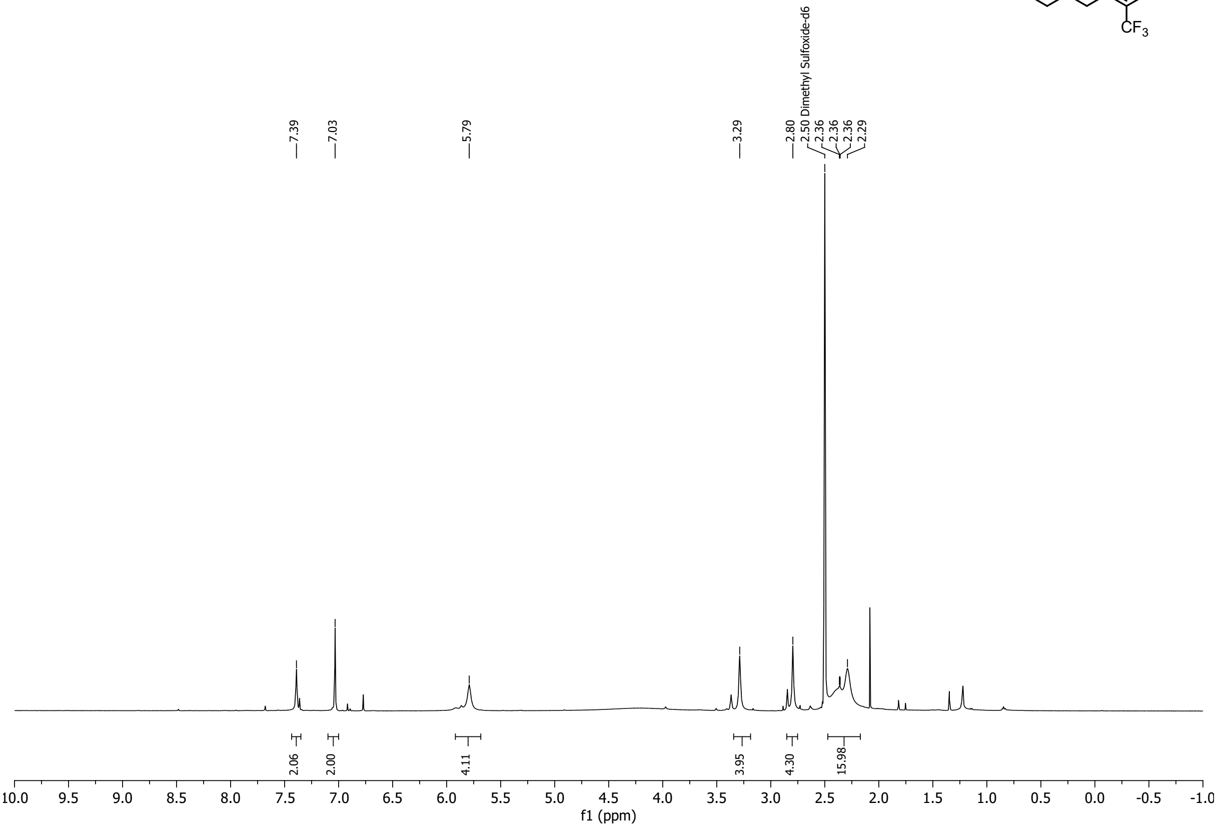

^{13}C NMR (126 MHz, DMSO-d_6):

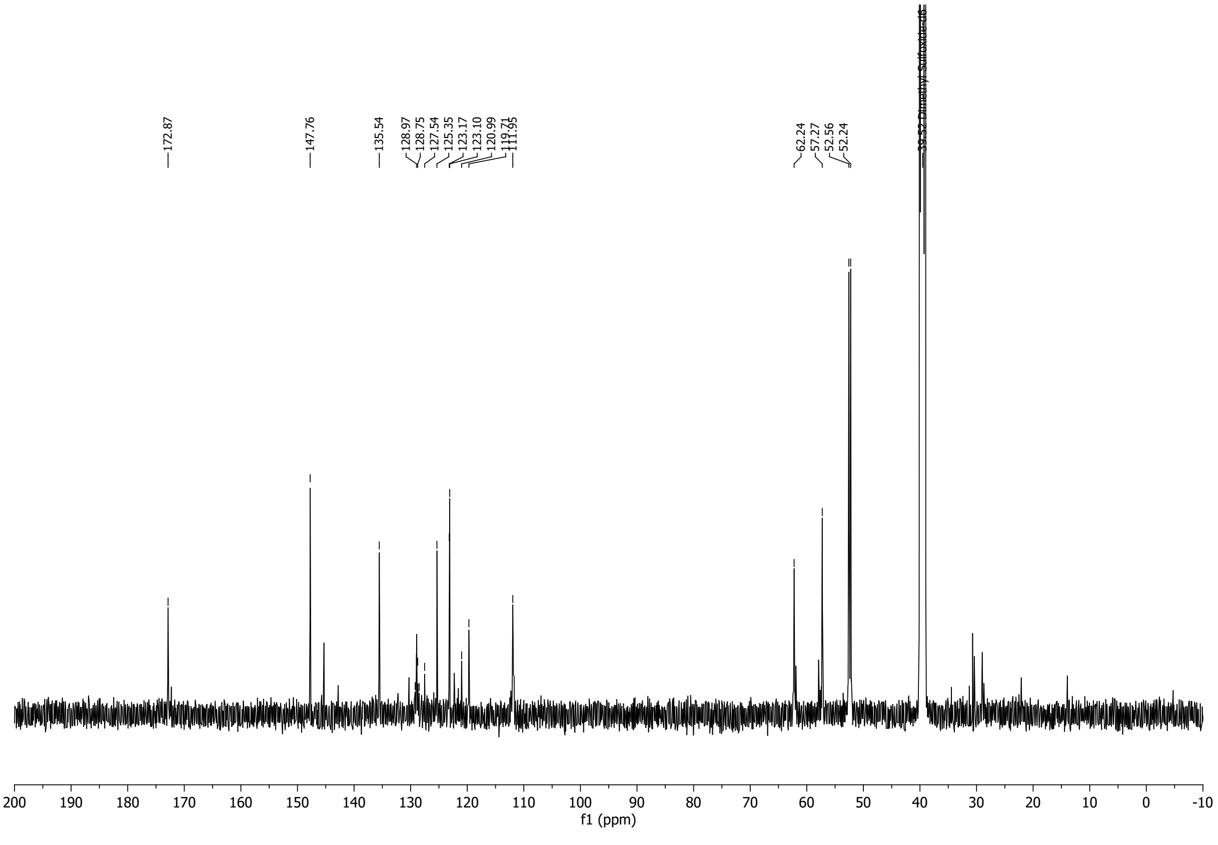

Diazide 28

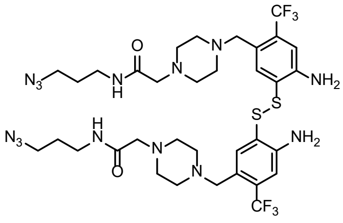

^{1}H NMR (500 MHz, $CDCl_3$):

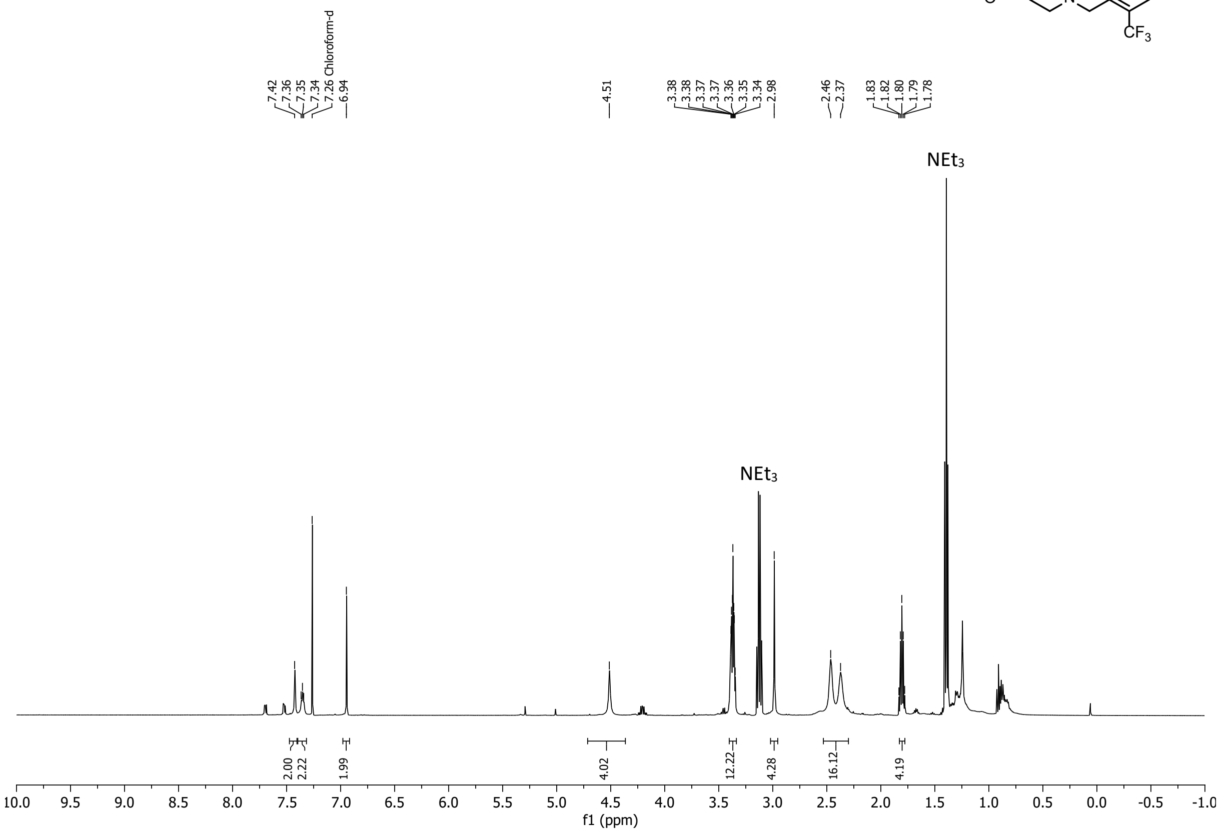

^{13}C NMR (126 MHz, $CDCl_3$):

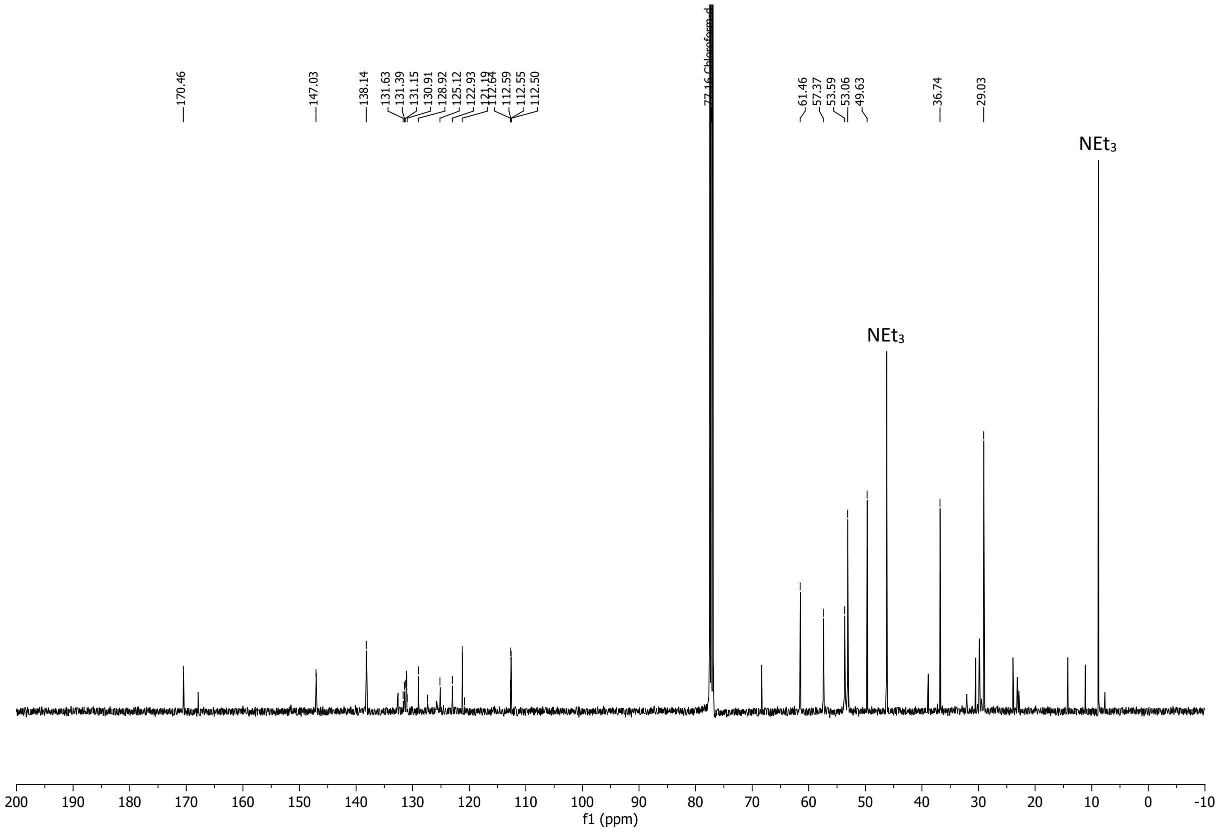

MPA-derived thioester 24

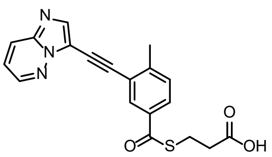

^{1}H NMR (400 MHz, DMSO-d_6):

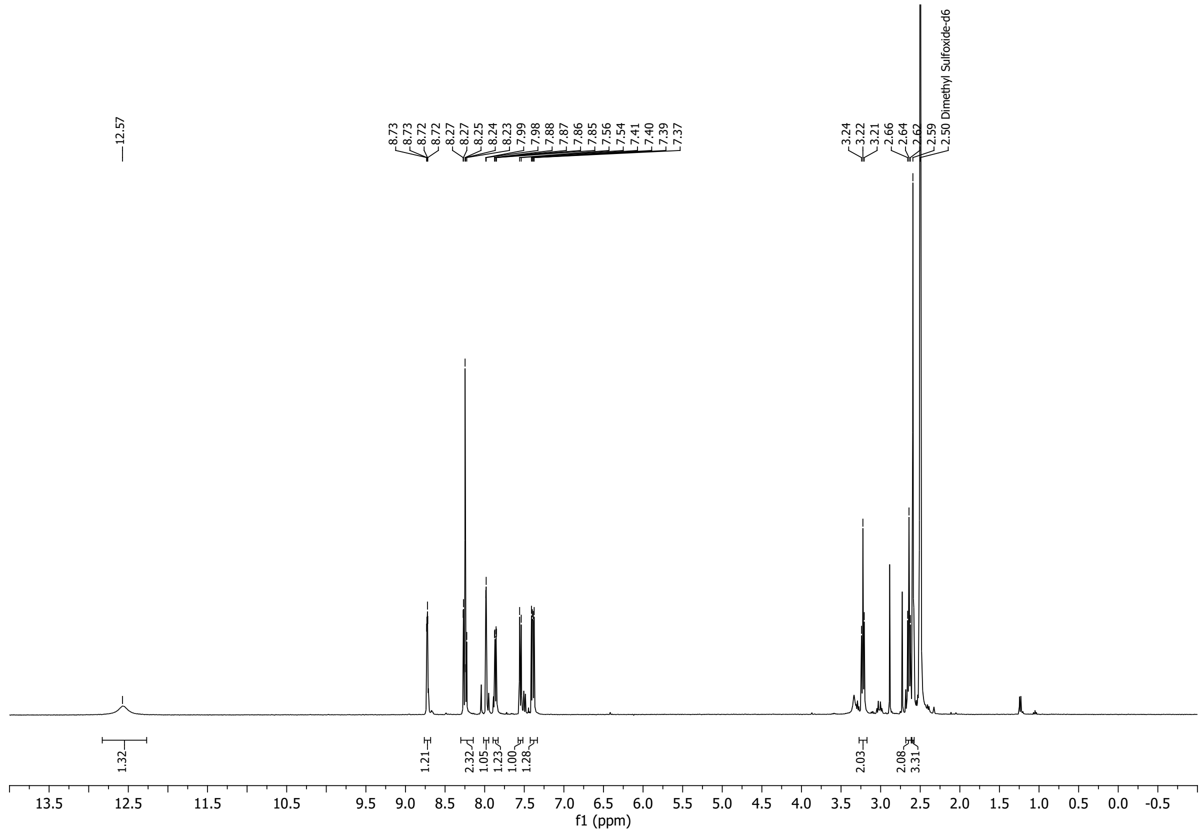

^{13}C NMR (101 MHz, DMSO-d_6):

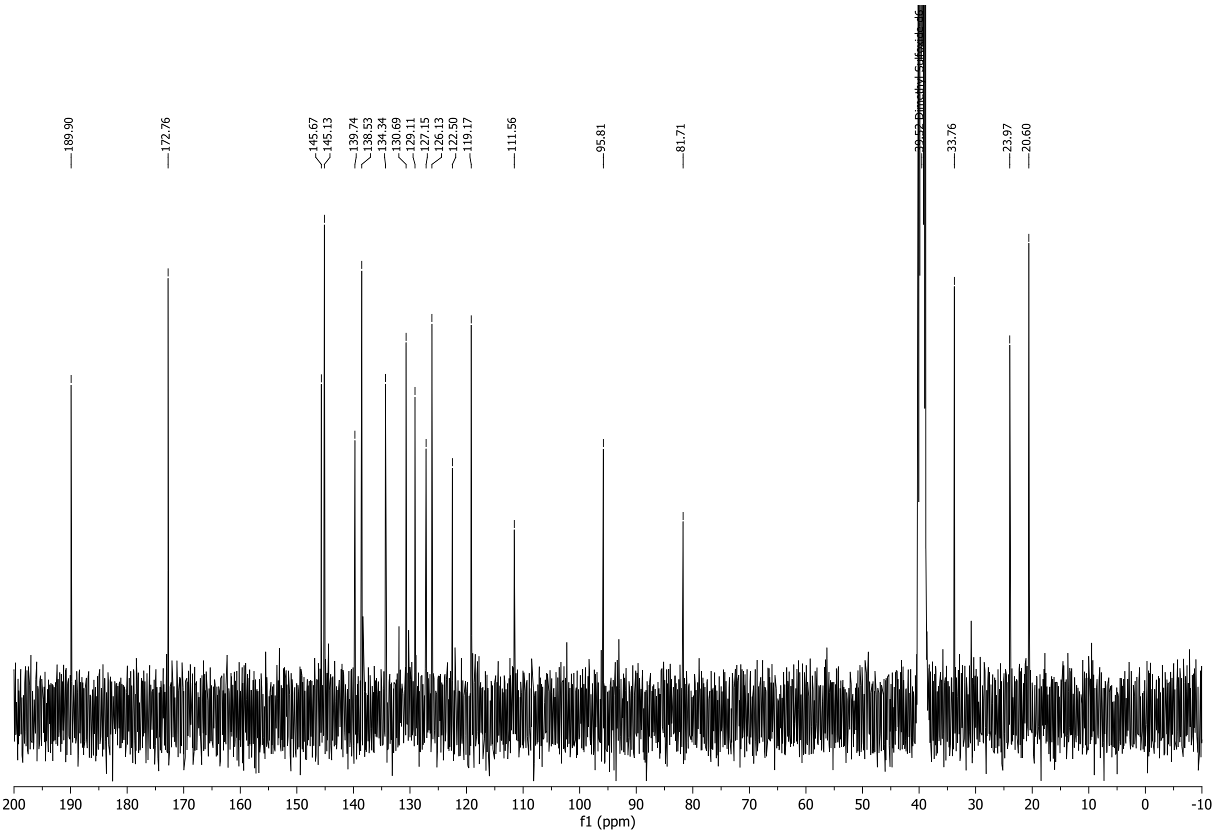

MPAA-derived thioester 25

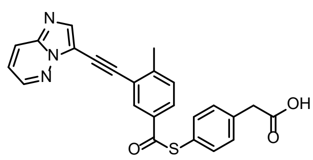

^{1}H NMR (400 MHz, DMSO-d_6):

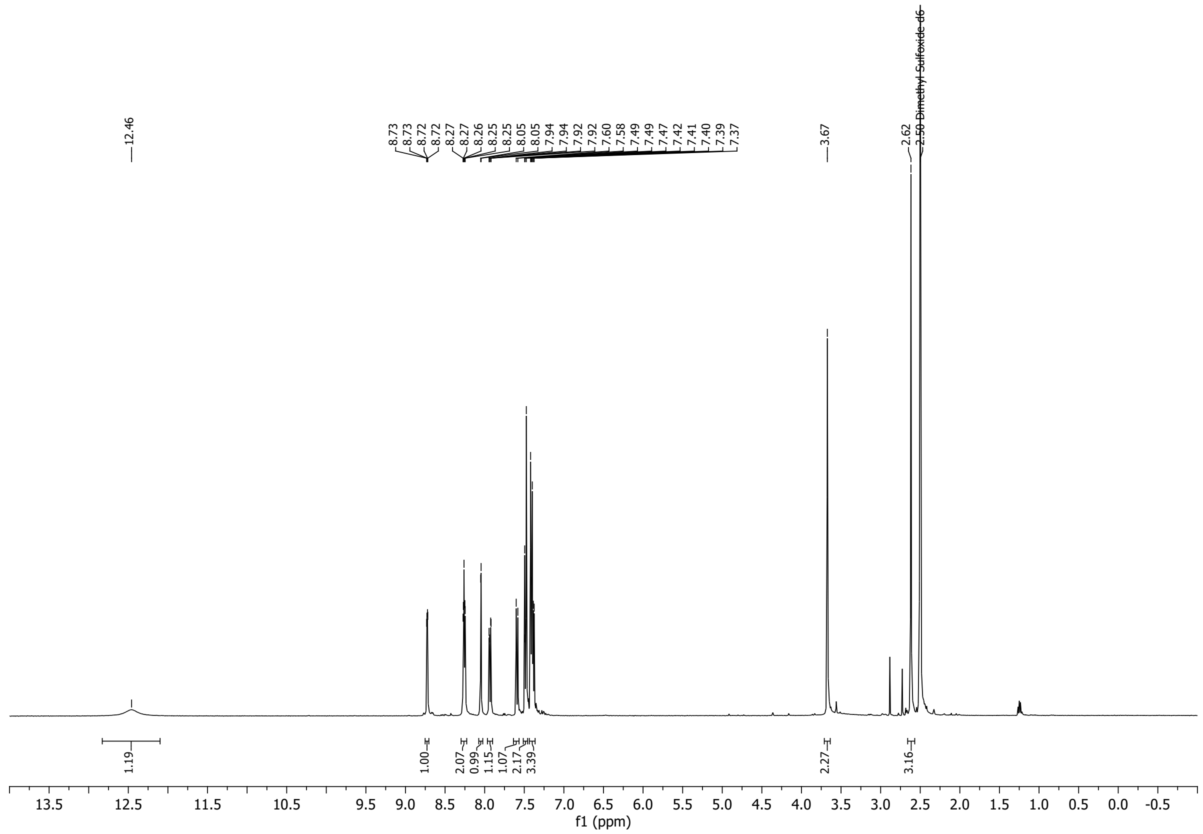

^{13}C NMR (101 MHz, DMSO-d_6):

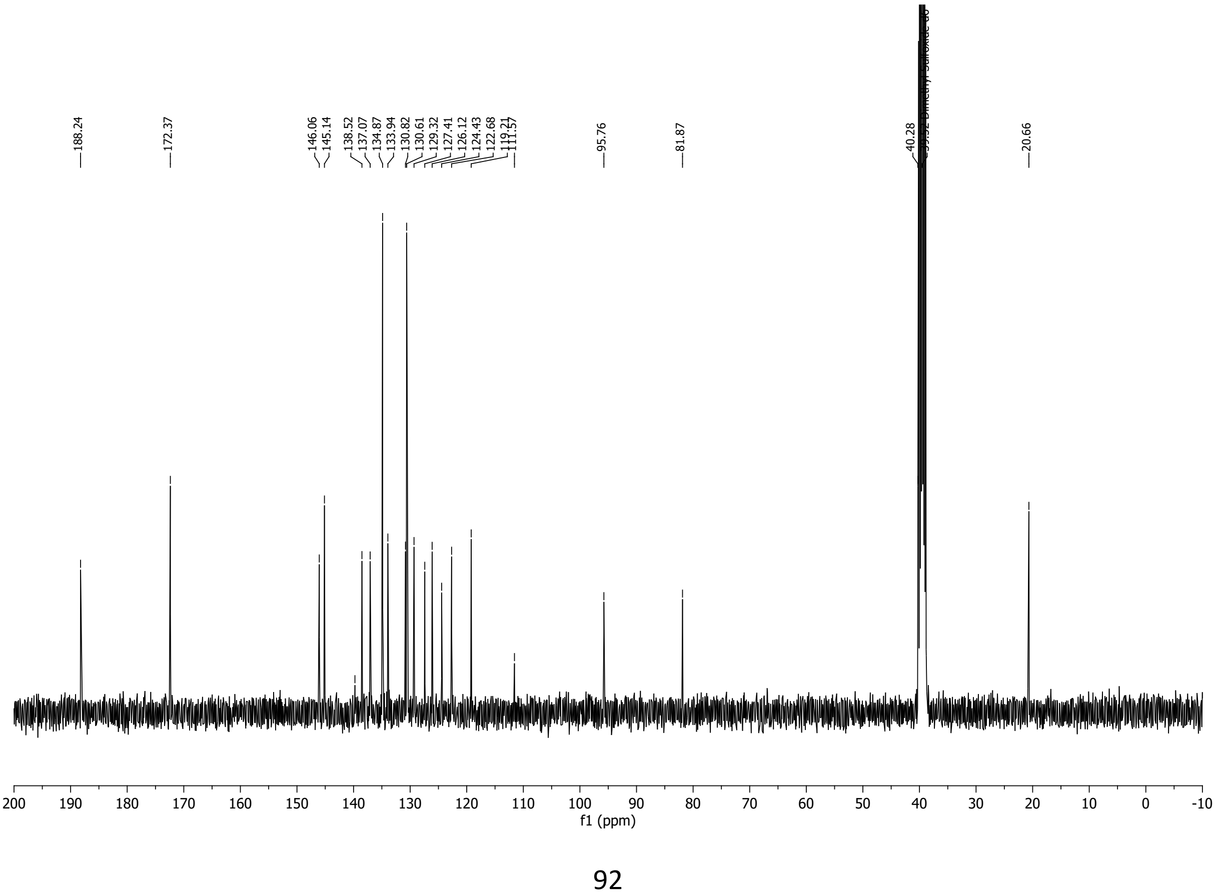

Azide-modified MPAA-derived thioester 29

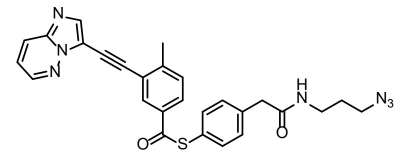

^{1}H NMR (500 MHz, $CDCl_3$):

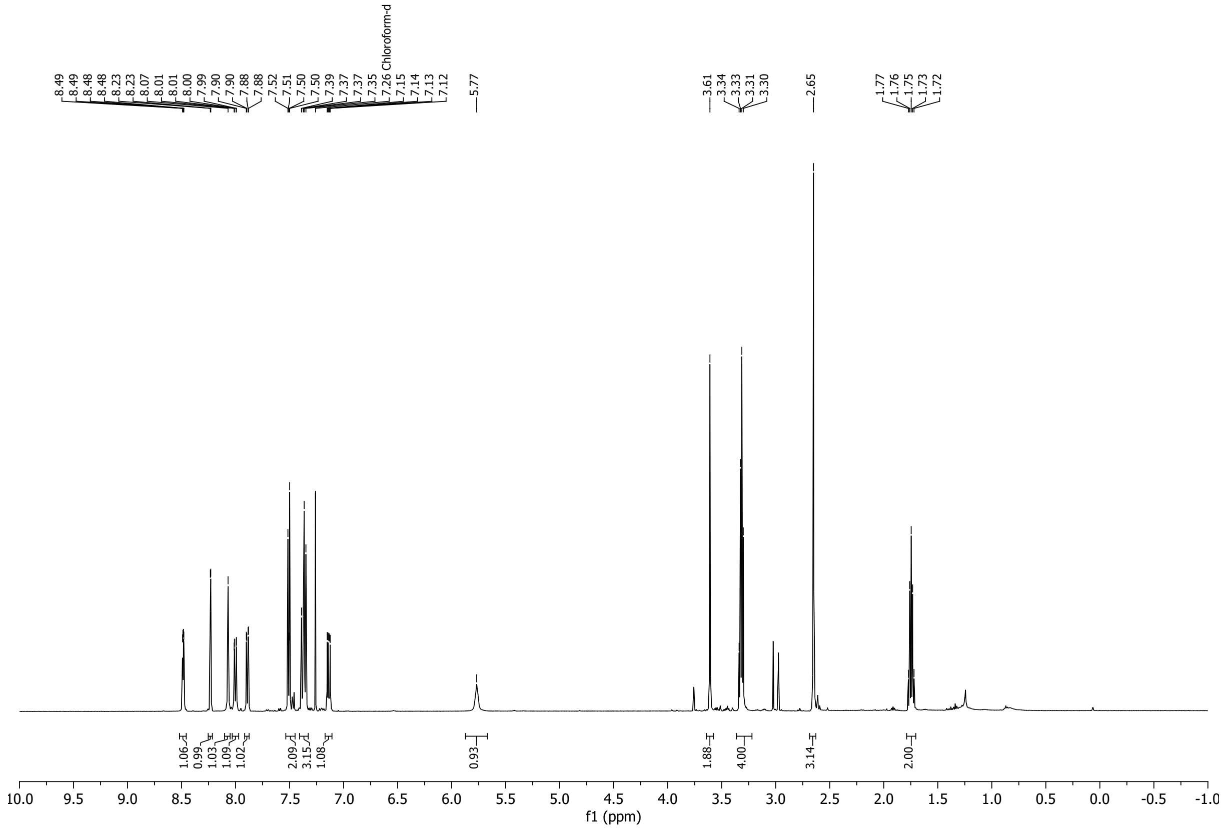

^{13}C NMR (126 MHz, $CDCl_3$):

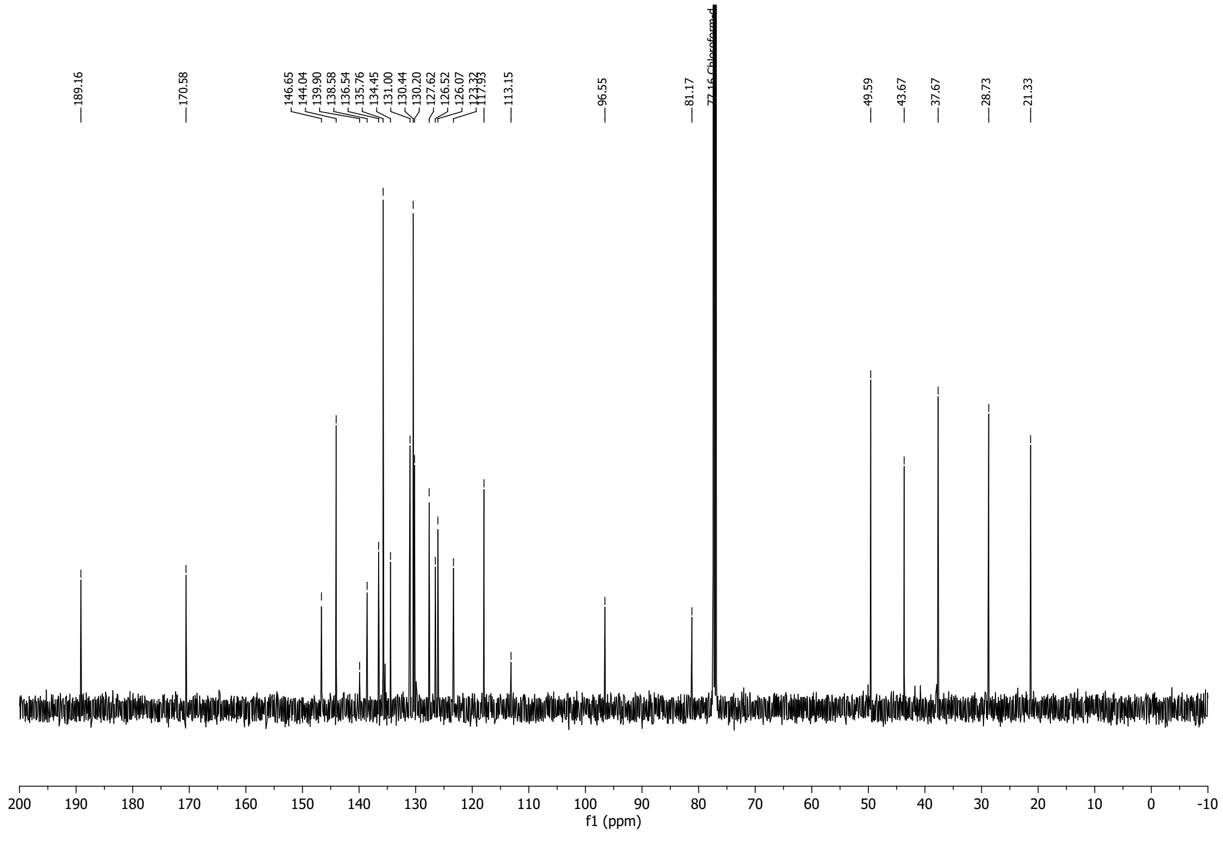

1-(Bromomethyl)-4-nitro-2-(trifluoromethyl)benzene (49)

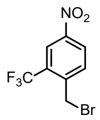

^{1}H NMR (500 MHz, $CDCl_3$):

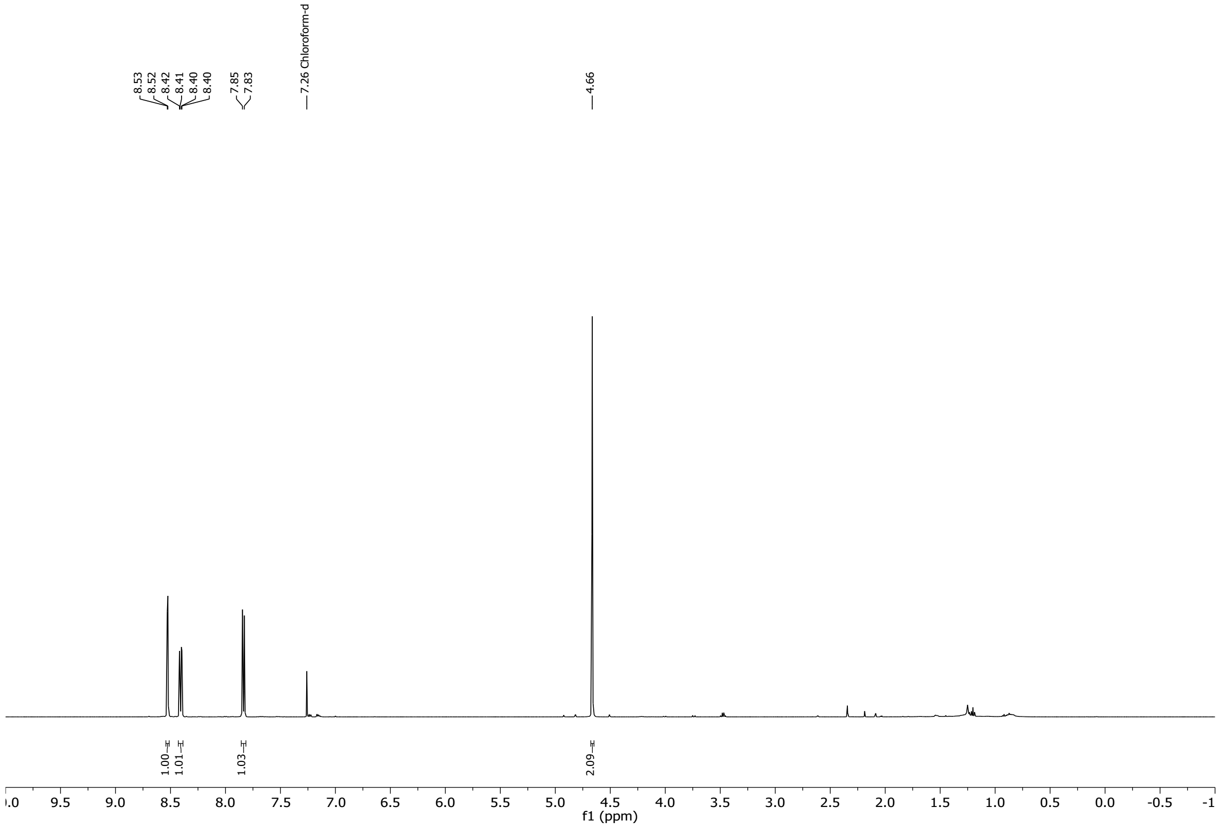

^{13}C NMR (126 MHz, $CDCl_3$):

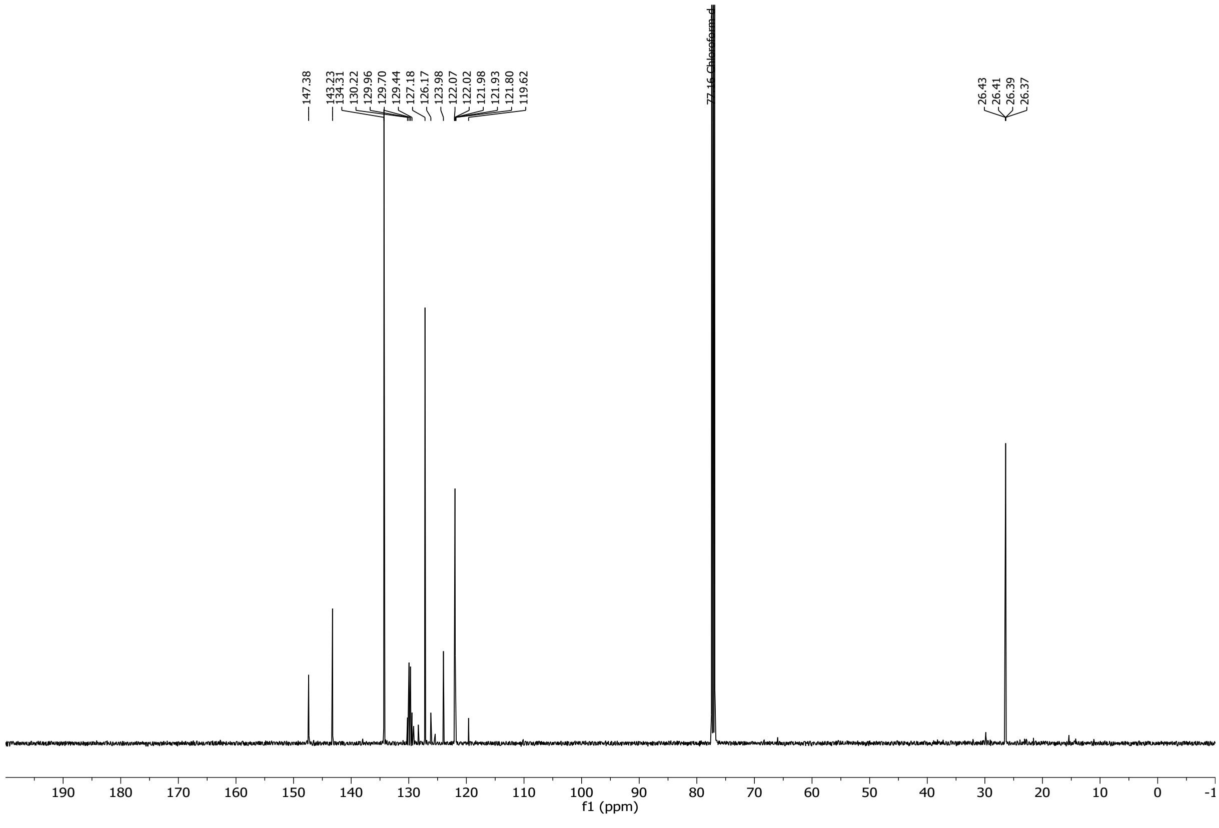

Ethyl 2-(4-(4-nitro-2-(trifluoromethyl)benzyl)piperazin-1-yl)acetate (50)

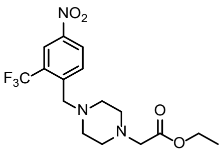

^{1}H NMR (500 MHz, $CDCl_3$):

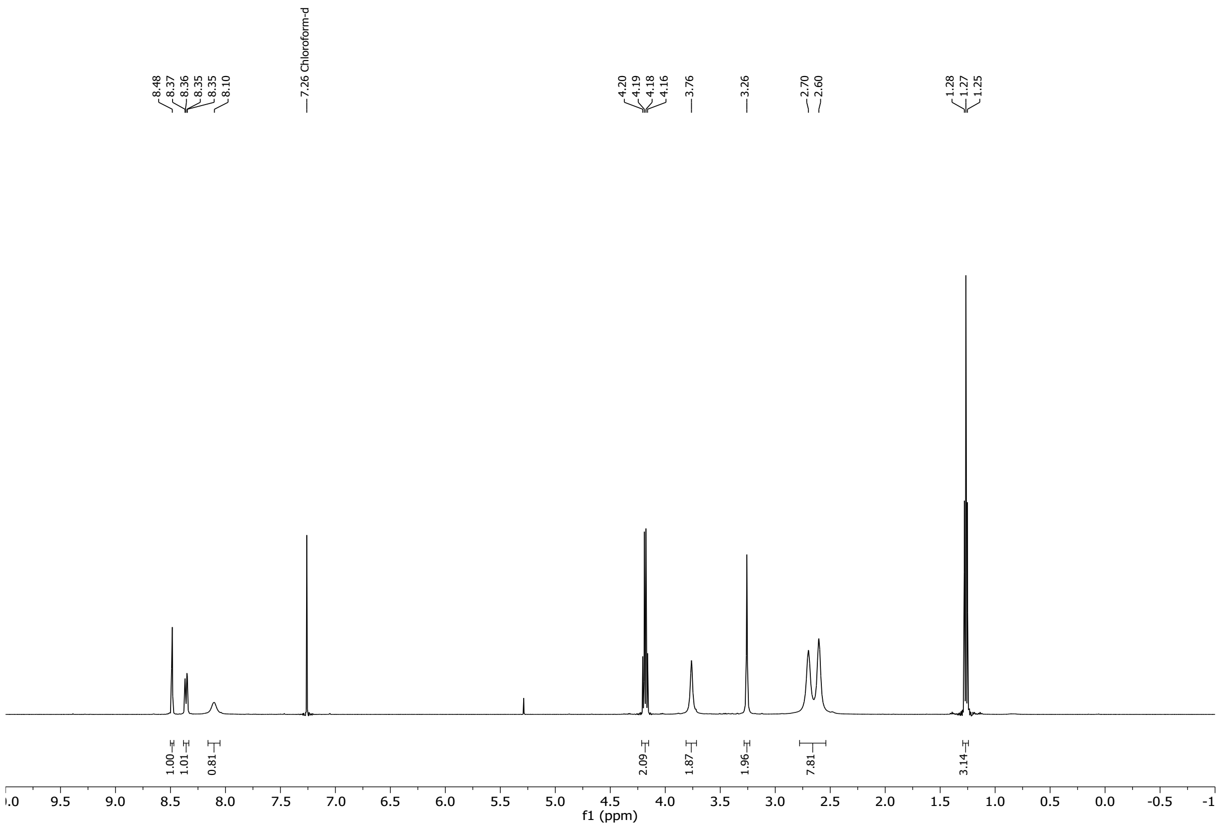

^{13}C NMR (126 MHz, $CDCl_3$):

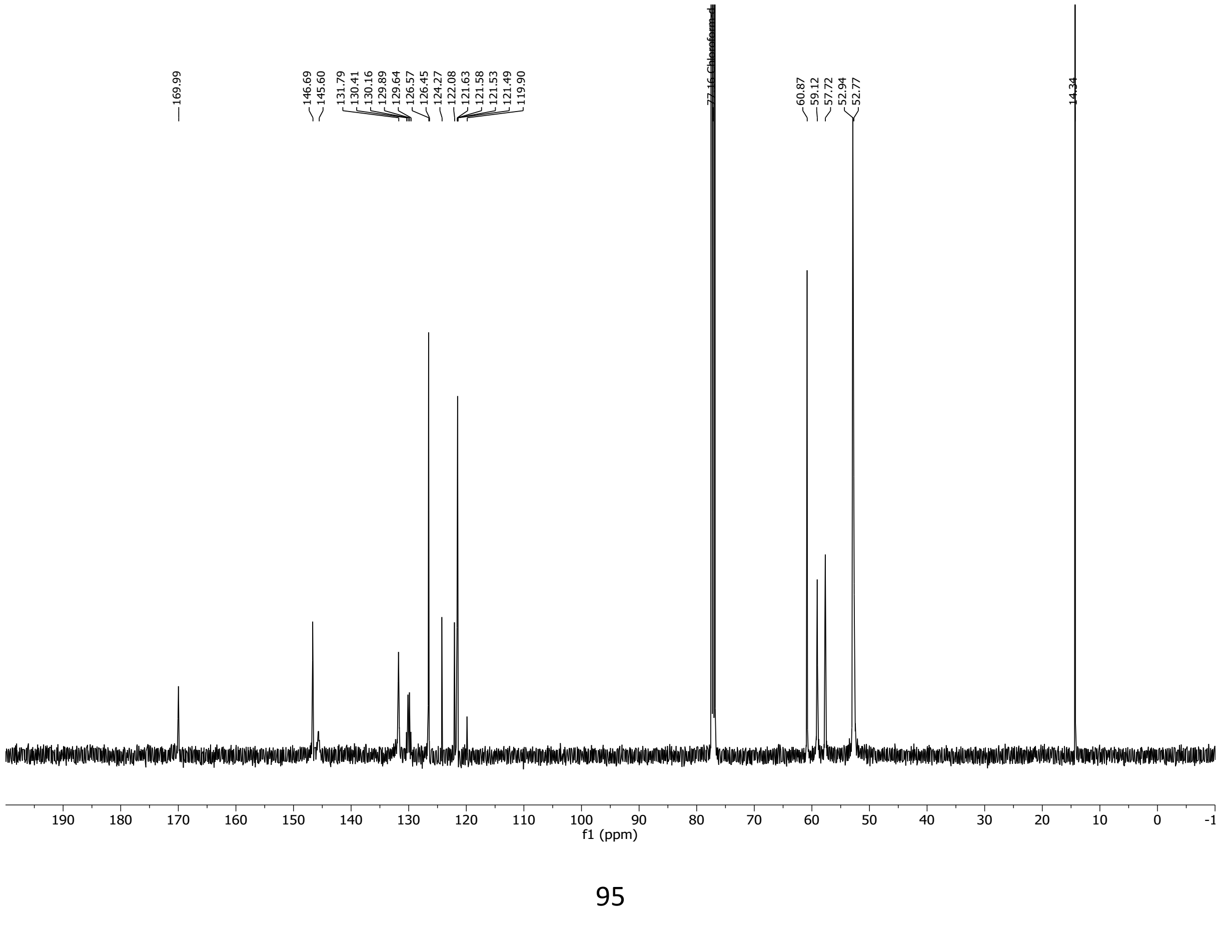

Ethyl 2-(4-(4-amino-2-(trifluoromethyl)benzyl)piperazin-1-yl)acetate (51)

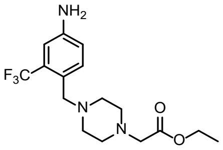

^{1}H NMR (500 MHz, $CDCl_3$):

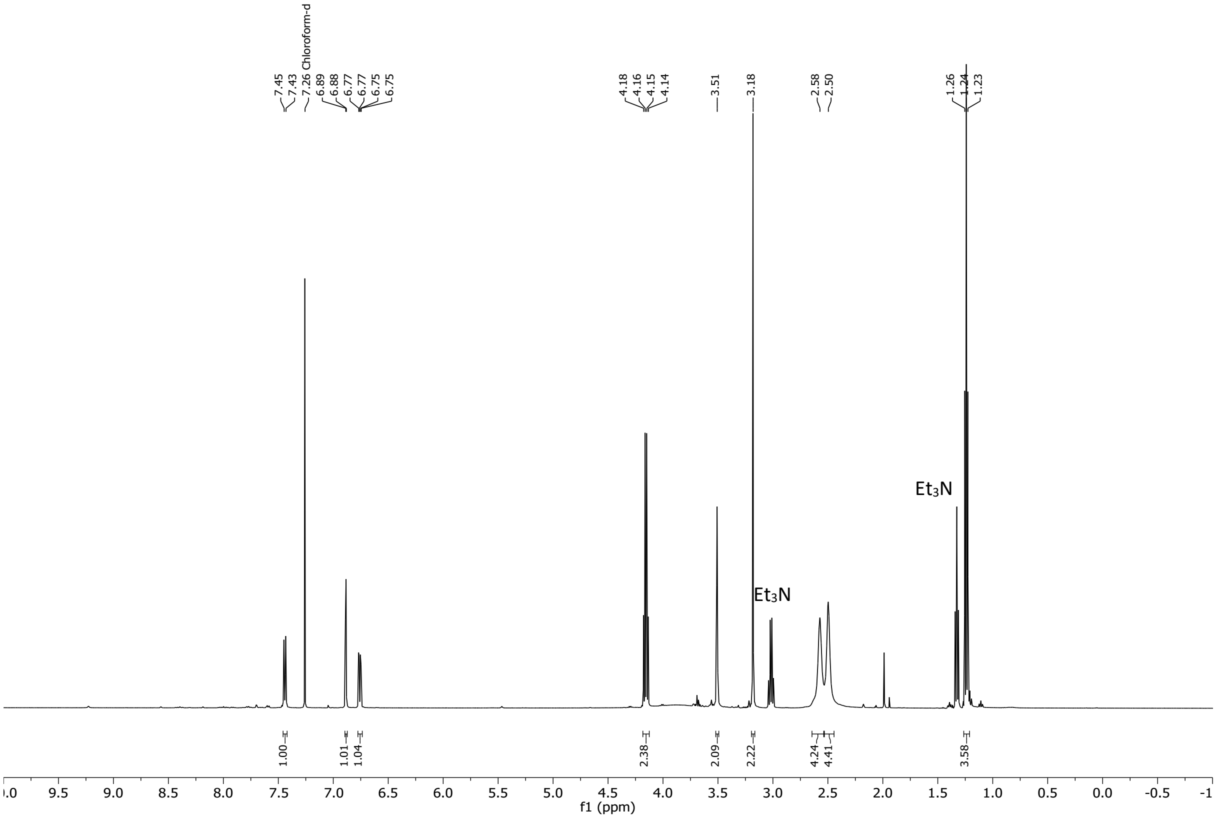

^{13}C NMR (126 MHz, $CDCl_3$):

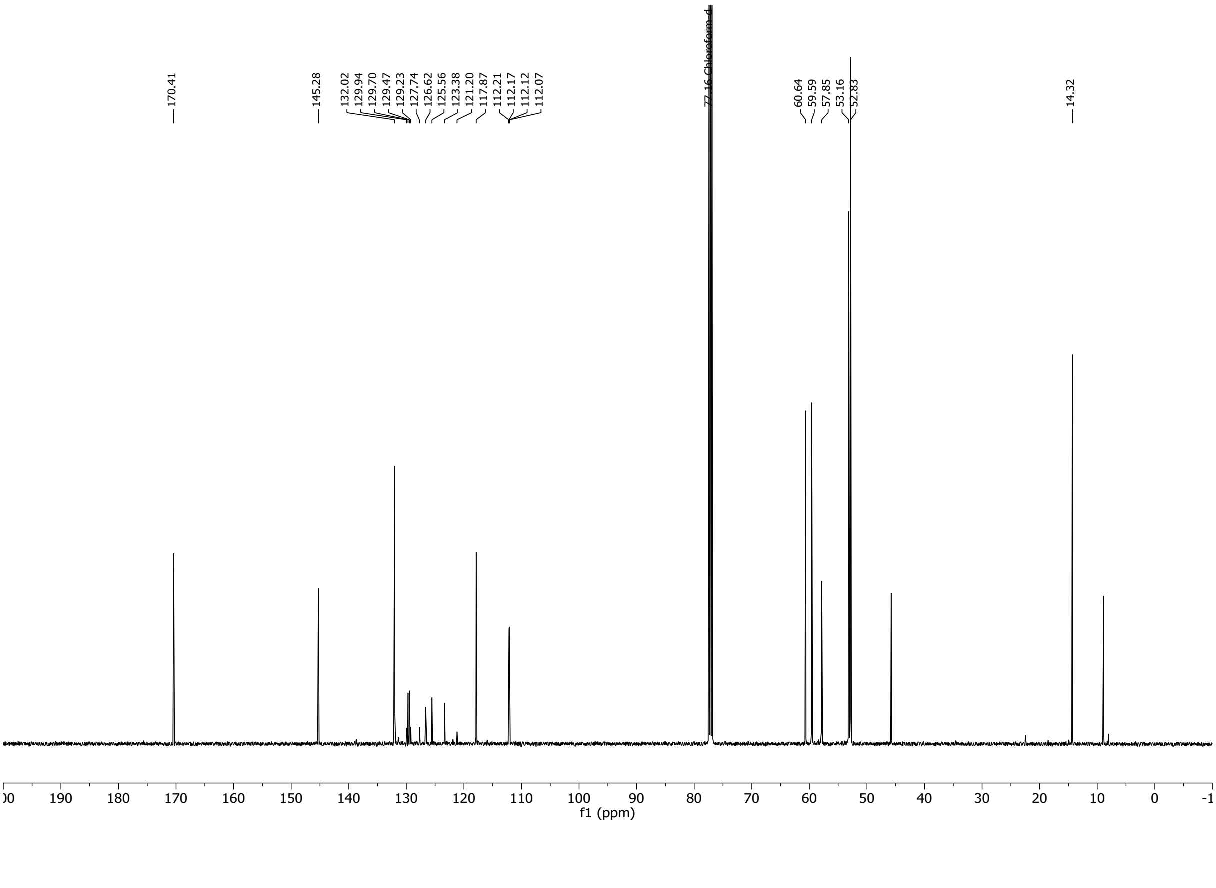

2-(4-(4-((*tert*-butoxycarbonyl)amino)-2-(trifluoromethyl)benzyl)piperazin-1-yl)acetic acid (53)

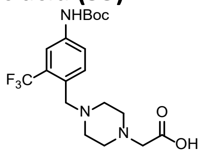

^{1}H NMR (400 MHz, CD_3CN/CD_3OD):

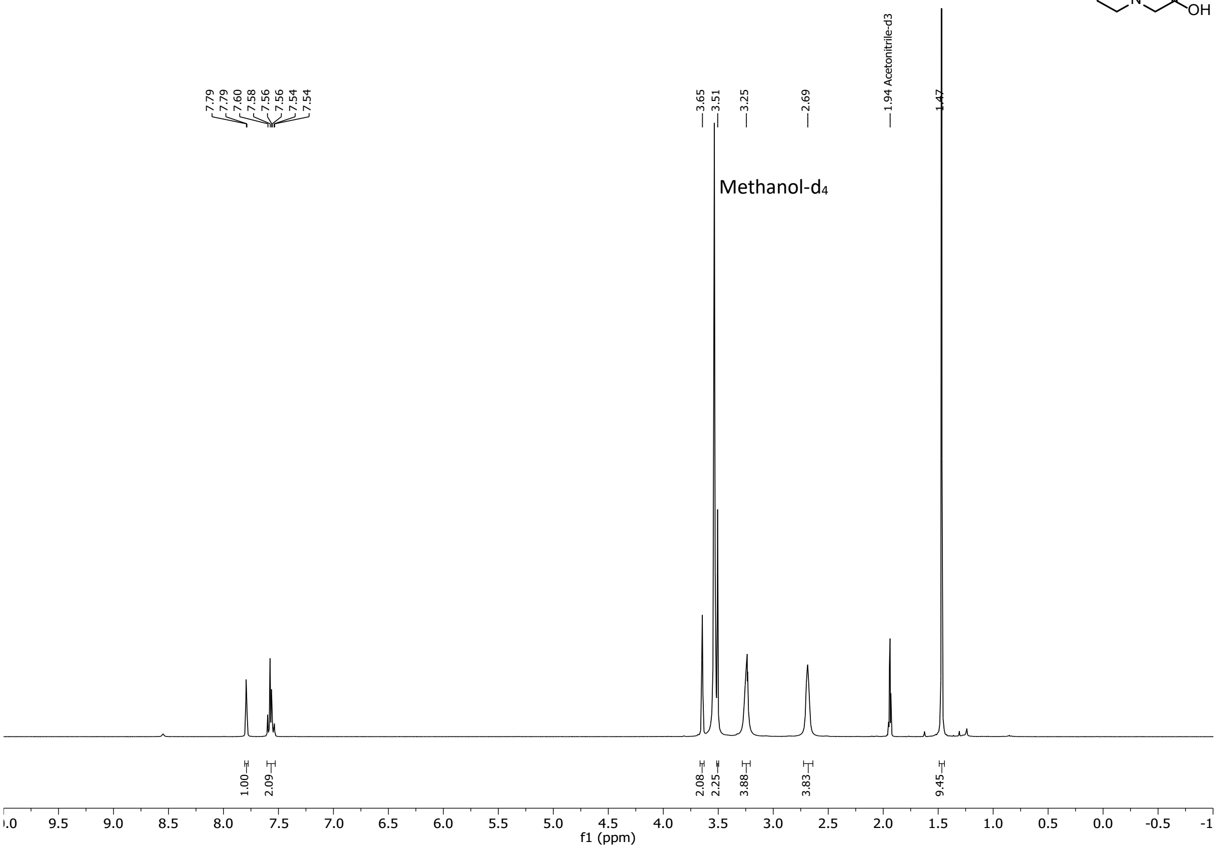

^{13}C NMR (101 MHz, CD_3CN/CD_3OD):

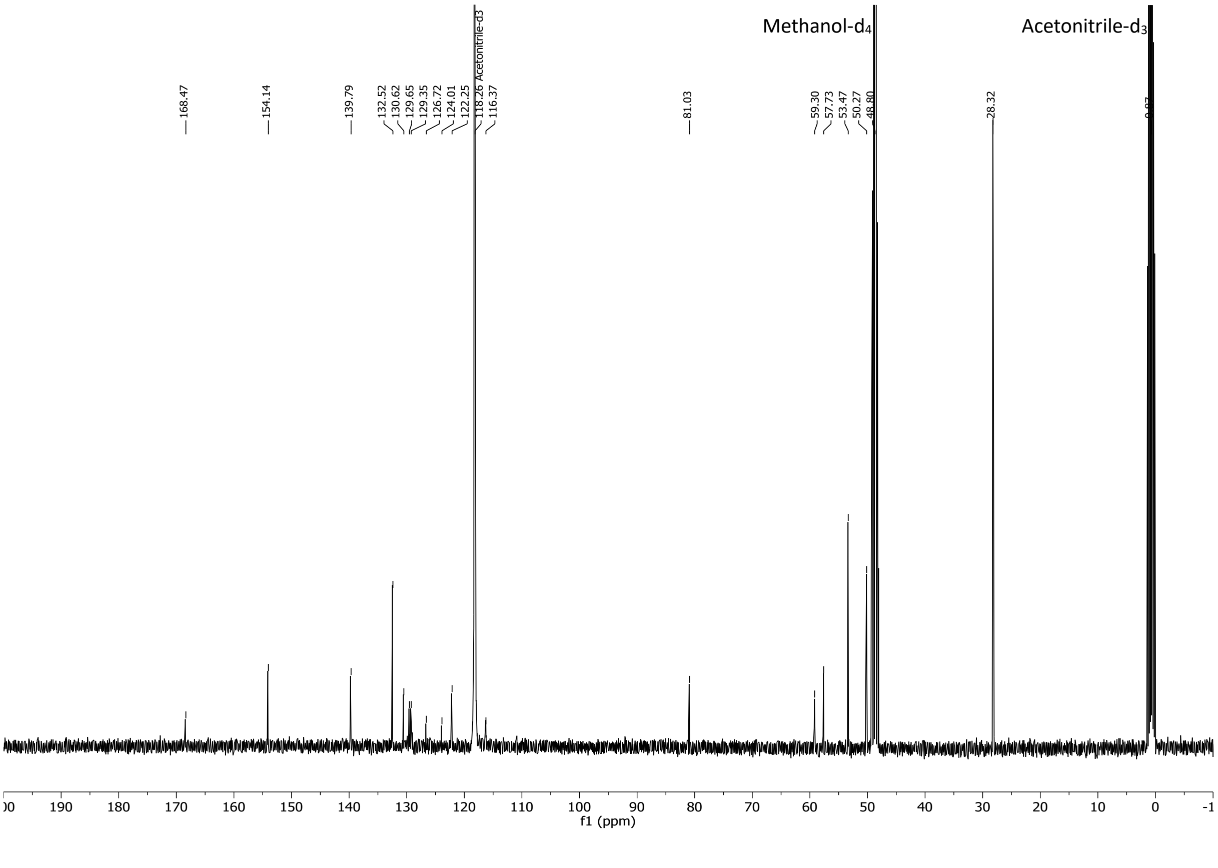

Abbreviations

AIBN, azobisisobutyronitrile; Ar, argon; Bhoc, benzhydryloxycarbonyl; Boc, *tert*-butyloxycarbonyl; CHAPS, 3-[(3-cholamidopropyl)dimethylammonio]-1-propanesulfonate; CML, chronic myeloid leukemia; conc., concentrated; dba, dibenzylideneacetone; DBCO, azadibenzocyclooctyne; DIPEA, *N*,*N*-diisopropylethylamine; DMAP, 4-dimethylaminopyridine; DMEM, Dulbecco's Modified Eagle Medium; EDT, 1,2-ethanedithiol; eq, equivalents; FCS, fetal calve serum; Fmoc, fluorenylmethoxycarbonyl; GnHCl, guanidinium chloride; HATU, 1-[bis(dimethylamino)methylene]-1*H*-1,2,3-triazolo-[4,5-*b*]pyridinium 3-oxide hexafluorophosphate; HCTU, *O*-(1*H*-6-chlorobenzotriazole-1-yl)-1,1,3,3-tetramethyluronium hexafluorophosphate; HOBt, *N*-hydroxybenzotriazole; Mmt, monomethoxytrityl; MOPS, 3-(*N*-morpholino)propanesulfonic acid; MPA, 3-mercaptopropionic acid; MPAA, 4-mercaptophenylacetic acid; NBS, *N*-bromosuccinimide; NMM, *N*-methylmorpholine; NMP, *N*-methyl-2-pyrrolidone; nt, nucleotide(s); PNA, peptide nucleic acid; rt, room temperature; SPS, solid-phase synthesis; TCEP, tris(2-carboxyethyl)phosphine; TFA, trifluoroacetic acid; TFMSA, triflic acid; THPTA, tris-(3-hydroxypropyltriazolylmethyl)amine; TIS, triisopropylsilane.

References

1. M. A. Fabian, W. H. Biggs, D. K. Treiber, C. E. Atteridge, M. D. Azimioara, M. G. Benedetti, T. A. Carter, P. Ciceri, P. T. Edeen, M. Floyd, J. M. Ford, M. Galvin, J. L. Gerlach, R. M. Grotzfeld, S. Herrgard, D. E. Insko, M. A. Insko, A. G. Lai, J.-M. Lelias, S. A. Mehta, Z. V. Milanov, A. M. Velasco, L. M. Wodicka, H. K. Patel, P. P. Zarrinkar and D. J. Lockhart, *Nat. Biotechnol.*, 2005, **23**, 329-336.
2. K. M. Korch, C. Eidamshaus, D. C. Behenna, S. Nam, D. Horne and B. M. Stoltz, *Angew. Chem. Int. Ed.*, 2015, **54**, 179-183.
3. S. Ueda, M. Su and S. L. Buchwald, *J. Am. Chem. Soc.*, 2012, **134**, 700-706.
4. C.-H. Zhang, M.-W. Zheng, Y.-P. Li, X.-D. Lin, M. Huang, L. Zhong, G.-B. Li, R.-J. Zhang, W.-T. Lin, Y. Jiao, X.-A. Wu, J. Yang, R. Xiang, L.-J. Chen, Y.-L. Zhao, W. Cheng, Y.-Q. Wei and S.-Y. Yang, *J. Med. Chem.*, 2015, **58**, 3957-3974.
